# Integrated widely targeted metabolomics and flavoromics reveal processing-driven dynamic changes in functional metabolites of *Eucommia ulmoides* leaf tea

**DOI:** 10.1016/j.fochx.2025.102434

**Published:** 2025-04-15

**Authors:** Yiyun Lin, Hui Ouyang, Ruobing Li, Yunzhe Shao, Xiangrong Tian, Yongkang Zhang

**Affiliations:** a National & Local United Engineering Laboratory of Integrative Utilization Technology of Eucommia Ulmoides, College of Chemistry and Chemical Engineering, Jishou University, Jishou 416000, PR China; b Hunan Engineering Laboratory for Analyse and Drugs Development of Ethnomedicine in Wuling Mountains, Jishou University, Jishou 416000, PR China; c School of Pharmaceutical Science, Jishou University, Jishou 416000, PR China; d Key Labotatory of Hunan Forest and Chemical Industry Engineering, Jishou University, Jishou 416000, PR China; e College of Biology and Environmental Science, Jishou University, Jishou 416000, PR China

**Keywords:** *Eucommia ulmoides* leaves, Tea, processing method, Widely targeted metabolomics, Flavoromics evaluation, Functional metabolites

## Abstract

This study employed a widely targeted metabolomics approach combined with flavoromics evaluation to systematically analyze dynamic metabolic variations under four processing techniques: fresh EUL (XY), traditionally dried EUL (HG), green tea processed EUL (GT), and black tea processed EUL (BT). A total of 1839 non-volatile metabolites and 289 volatile metabolites were identified. Key affected metabolites included amino acids (e.g., essential amino acids upregulated 90 % in HG), lipids (oxidized fatty acids elevated in BT), phenolic acids (23 % increase in GT), flavonoids (120+ downregulated in BT), nucleotides, and terpenoids. Each processing method demonstrates unique advantages: HG preserves amino acids, GT enhances catechin content, and BT optimizes secondary metabolites. This study provides the first comprehensive metabolic map of EUL processing, offering a scientific foundation for standardizing product quality and developing functional applications.

## Highlights

• *Eucommia ulmoides* leaf tea from different processing methods were characterized by widely targeted metabolomics approach.
• A total of 2127 (non-volatile and volatile) metabolites were observed in differently treated *Eucommia ulmoides* leaf tea.
• The BT treatment helped release secondary metabolites (e.g. flavonoids and alkaloids, etc.), but it reduced amino acids.
• The GT treatment could enhance the content of flavonoids, phenolic acids and tannins.
• The HG treatment method effectively retained some of the original substances in XY.

## 1 Introduction

*Eucommia ulmoides leaf (EUL)*, a dual-purpose medicinal and edible resource from the Eucommiaceae family, has been documented in the Chinese Pharmacopoeia for its traditional therapeutic effects in nourishing the liver/kidney, strengthening musculoskeletal function, and regulating blood pressure. Contemporary pharmacological studies have further elucidated its multifunctional bioactivities, including antihypertensive (Guo et al., 2020), antidiabetic, antioxidant (Li et al., 2022), and anti-osteoporotic properties. With the rising popularity of EUL-derived tea products, comprehensive characterization of its bioactive composition under different processing conditions has become a research priority.

Existing studies predominantly focus on conventional chemical markers (e.g., total flavonoids, chlorogenic acid) in specific processing methods (Peng et al., 2021; Shi et al., 2022). However, these analytical approaches provide fragmented metabolite profiles, failing to capture the holistic metabolic reprogramming induced by processing. Such limitations substantially hinder our understanding of synergistic effects between processing parameters and functional components, thus constraining the value-added utilization of EUL resources.

Emerging advancements in metabolomics offer promising solutions. Widely targeted metabolomics analysis, a novel approach amalgamating the expansiveness inherent in nontargeted metabolomics with the precision of targeted metabolomics(Lozada et al., 2023), has demonstrated exceptional performance in tracking metabolic dynamics during food processing (Baky et al., 2022; Dossou et al., 2022; Gaffney et al., 2021; Nguyen et al., 2024; Y. Xiao et al., 2022). This study pioneers the application of this approach to EUL processing research. Through systematic analysis of non-volatile/volatile metabolomes of EUL from different processing, integrated with flavoromics evaluation, we aim to address three critical scientific questions: (1) processing-specific regulation patterns of characteristic metabolite networks in EUL; (2) directional transformation mechanisms of key functional components (amino acids, phenolic acids, flavonoids); (3) correlations between process optimization and product quality enhancement. The findings will establish a theoretical foundation for standardizing EUL tea production and developing functional food products.

## 2 Materials and methods

### 2.1 Materials and reagents

EUL was obtained from the plants grown at the Jishou University planting base in Jishou City (109°72′E; 28°32′),(Hunan Province, China). Species: *E. ulmoides* Oliv.; Month of harvest: June.

Methanol (MeOH), acetonitrile (ACN), and n-hexane (Hex) for liquid chromatography grade solvents (HPLC) were purchased from Merck (Darmstadt, Germany), and formic acid was purchased from Sigma Aldrich (St. Louis, Missouri). Analytically pure sodium chloride (NaCl) was obtained from Sinopharm Chemical Reagent Co Ltd. (Shanghai, China).

### 2.2 Processing of EUL prepared by different processes

#### 2.2.1 Fresh leaves (XY)

Fresh EUL, with a consistent size, were carefully harvested and subsequently washed twice using distilled water.

#### 2.2.2 Low temperature drying (HG)

HG Preparation Process: fresh EUL (1.67 kg) → low temperature drying(75 °C, 3 h)(selected per Chinese Pharmacopoeia guidelines to protect heat-sensitive compounds).

Following the guidelines outlined in the current Chinese Pharmacopoeia.

#### 2.2.3 Processing in operating procedure of black tea (BT)

BT operating procedure: fresh EUL (1.95 kg) → spreading(11*h*) → withering(28 °C, 8 h) → rolling(25 min) → fermentation(28 °C, 5 h) (optimized based on preliminary trials to balance enzymatic oxidation and metabolite preservation) → low temperature drying(75 °C, 2.5 h) (selected per Chinese Pharmacopoeia guidelines to protect heat-sensitive compounds).

#### 2.2.4 Processing in operating procedure of green tea (GT)

GT Preparation Process: fresh EUL (1.26 kg) → spreading(1 h) → removing(100–120 °C, 4.5 min) → rolling(10 min) → low temperature drying(75 °C, 3 h) (selected per Chinese Pharmacopoeia guidelines to protect heat-sensitive compounds).

Samples were weighed, frozen in liquid nitrogen, and stored at −80 °C for further study.

### 2.3 Sensory evaluation

The sensory evaluation was approved by the Ethics Committee of Jishou University (Approval No.JSDX-2024-0131), and written informed consent was obtained from all participants.The sensory evaluation was conducted by a team of five trained panelists (three males and two females, aged 20–30 years) in accordance with the Chinese National Standard GB/T 23776–2018, which aligns with ISO 8586:2012 guidelines for sensory assessor training. All participants were informed about the experiment and its details and volunteered to join. The specific details of the assessment were conducted according to the Chinese National Sensory Evaluation Method for Tea (GB/T 23776–2018), and the samples were randomly presented to all assessors. Firstly, 200 g of each tea sample was placed in a white square tray to evaluate the appearance of the samples. Subsequently, 3 g of each sample was steeped in 150 mL of boiling water for 5 min, and the tea broth was filtered and placed in a white porcelain cup. In this cup, the panelists assessed the quality and gave scores for color, aroma, and taste accordingly. Finally, a score was given for the appearance of the steeped leaves based on the observation of the brewed samples. The overall quality score is calculated on a 100-point scale: The total quality score is the sum of the values of each component, with a maximum of 100 points. The components and their respective weights are as follows: dry tea contour (25 %), soup color (10 %), aroma (25 %), taste (30 %), and appearance of infused leaves (10 %).

### 2.4 Non-volatile metabolites

#### 2.4.1 Preparation process of different samples for UPLC-MS/MS analysis

Using vacuum freeze-drying technology, place the biological samples in a lyophilizer (Scientz-100F), then grinding (30 Hz, 1.5 min) the samples to powder form using a grinder (MM 400, Retsch). Next, weigh 50 mg of sample powder using an electronic balance (MS105DΜ) and add 1200 μL of −20 °C pre-cooled 70 % methanolic aqueous internal standard extract (less than 50 mg added at the rate of 1200 μL extractant per 50 mg sample). Vortex once every 30 min for 30 s, for a total of 6 times. After centrifugation (rotation speed 12,000 rpm, 3 min), the supernatant was aspirated, and the sample was filtered through a microporous membrane (0.22 μm pore size) and stored in the injection vial for UPLC-MS/MS analysis.

#### 2.4.2 UPLC and ESI-QTRAP-MS/MS conditions

The sample extracts were analyzed using an ultra-performance liquid chromatography-electrospray ionization-triple quadrupole-linear ion trap mass spectrometry (UPLC-ESI-QTRAP-MS/MS) system. The UPLC system (ExionLC™ AD, SCIEX) was coupled with a hybrid triple quadrupole-linear ion trap mass spectrometer (6500 Q TRAP, SCIEX).The analytical conditions were as follows, UPLC: column, Agilent™ SB-C18 (1.8 μm, 2.1 mm * 100 mm). The mobile phase was consisted of solvent A, pure water with 0.1 % formic acid, and solvent B, acetonitrile with 0.1 % formic acid. Sample measurements were performed with a gradient program that employed the starting conditions of 95 % A, 5 % B. Within 9 min, a linear gradient to 5 % A, 95 % B was programmed, and a composition of 5 % A, 95 % B was kept for 1 min. Subsequently, a composition of 95 % A, 5.0 % B was adjusted within 1.1 min and kept for 2.9 min. The flow velocity was set as 0.35 mL per minute. The column oven was set to 40 °C. The injection volume was 2 μL. The effluent was alternatively connected to an ESI-triple quadrupole-linear ion trap (QTRAP)-MS. Data acquisition and processing were performed using Analyst TF 1.6.3 software (SCIEX).

The ESI source operation parameters were as follows: source temperature 500 °C; ion spray voltage (IS) 5500 V (positive ion mode)/−4500 V (negative ion mode); ion source gas I (GSI), gas II(GSII), curtain gas (CUR) was set at 50, 60, and 25 psi, respectively; the collision-activated dissociation (CAD) was high. QQQ scans were acquired as MRM experiments with collision gas (nitrogen) set to medium. DP (declustering potential) and CE (collision energy) for individual MRM transitions was done with further DP and CE optimization. A specific set of MRM transitions were monitored for each period according to the metabolites eluted within this period.

#### 2.4.3 Qualification and quantification of non-volatile metabolites

The data obtained from UPLC-TripleTOF6600 were characterized for nonvolatiles by more accurate mass, retention time and isotope ratios with a commercial database from Metware (Hubei, China). After obtaining the metabolite profiling data of different samples, peak area integration was performed for all the substance chromatographic peaks and the integration was corrected for the mass spectrometry peaks of the same metabolite in different samples among them (Fraga et al., 2010).

### 2.5 Analysis of volatile metabolites

#### 2.5.1 Preparation process of different samples for GC–MS analysis

Materials were harvested, weighted, immediately frozen in liquid nitrogen, and stored at −80 °C until needed. Samples were ground to a powder in liquid nitrogen.

500 mg (1 mL) of the powder was transferred immediately to a 20 mL head-space vial (Agilent, Palo Alto, CA, USA), containing NaCl saturated solution, to inhibit any enzyme reaction. The vials were sealed using crimp-top caps with TFE‑silicone headspace septa (Agilent). At the time of SPME analysis, each vial was placed in 60 °C for 5 min, then a 120 μm DVB/CWR/PDMS fibre (Agilent) was exposed to the headspace of the sample for 15 min at 60 °C.

#### 2.5.2 Acquisition conditions for GC–MS analysis

After sampling, desorption of the VOCs from the fibre coating was carried out in the injection port of the GC apparatus (Model 8890; Agilent) at 250 °C for 5 min in the splitless mode. The identification and quantification of VOCs was carried out using an Agilent Model 8890 GC and a 7000D mass spectrometer (Agilent), equipped with a 30 m × 0.25 mm × 0.25 μm DB-5MS (5 % phenyl-polymethylsiloxane, Agilent J&W Scientific, Folsom, CA, USA) capillary column. Helium was used as the carrier gas at a linear velocity of 1.2 mL/min. The injector temperature was kept at 250 °C and the detector at 280 °C. The oven temperature was programmed from 40 °C (3.5 min), increasing at 10 °C /min to 100 °C, at 7 °C /min to 180 °C, at 25 °C /min to 280 °C, hold for 5 min. Mass spectra was recorded in electron impact (EI) ionisation mode at 70 eV. The quadrupole mass detector, ion source and transfer line temperatures were set, respectively, at 150, 230 and 280 °C. The MS was selected ion monitoring (SIM) mode was used for the identification and quantification of analytes.Data analysis were performed using MassHunter Qualitative Analysis B.06.00 (Agilent Technologies).

LC internal standards: Specified the use of 2-Chlorophenylalanine (98 % purity, J&K Scientific, Lot LBCOR15, CAS:14091–11-3), with QC samples for system monitoring.

#### 2.5.3 Qualification and quantification of volatile metabolites

Based on multi-species, literature, some specimens, and retention index, the database is established independently. Then Selected Ion Monitoring (SIM) mode for accurate scanning, 1 quantitative ion and 2–3 qualitative ions for each compound respectively. All the ions to be detected in each group were detected separately in time periods according to the order of peak appearance; if the retention time of the detected peaks was consistent with the standard reference and the selected ions all appeared in the mass spectra of the samples after deduction of the background, the substance was judged to be the substance; the quantitative ions were selected for the integration of the chromatographic peaks and the calibration work, to enhance the accuracy of the quantification.

GC internal standards: Specified the use of deuterated 3-Hexanone-2,2,4,4-d4 (CDN Isotopes, Canada, CAS:24588–54-3), with QC samples for system monitoring.

#### 2.5.4 rOAV metabolite calculations

Relative odor activity value (rOAV) is a method for determining the key flavor compounds of a food established in combination with the sensory thresholds of the compounds, and is used to clarify the contribution of each aroma compound to the overall aroma profile of the sample. The application of this method can identify the causes of the same or different flavors from EUL of different processing processes. Generally rOAV ≥1 suggests that the compound has a direct contribution to the flavor of the sample. According to literature(Huang et al., 2022) (Xue et al., 2022), an rOAV analysis was performed with the following formula:

$$\mathrm{rOA}V_{i}=\frac{C_{i}}{T_{i}}$$

In the formula, $\mathrm{rOA}V_{i}$ is the relative odor activity value of compound i, and $C_{i}$ is the relative content of the compound (μg/g or μg/mL); $T_{i}$ is the threshold of the compound (Threshold, μg/g or μg/mL).

The threshold values, $T_{i}$ were obtained from reputable databases: The Good Scents Company (http://www.thegoodscentscompany.com), Perflavory Information System (http://perflavory.com), Flavor Ingredient Library (https://www.femaflavor.org/flavor-library), LRI & Odor (http://www.odour.org.uk/odour/index.html), and Food Flavor Lab (http://foodflavorlab.cn/#/home).

#### 2.5.5 Metabolomics analysis and network visualisation

PCA: Unsupervised PCA (principal component analysis) was performed using the prcomp function in *R. prior* to analysis, missing values in the metabolite data were imputed with zeros, and the data matrix was normalized by unit variance scaling to reduce technical variability. The data was unit variance scaled before unsupervised PCA.

Hierarchical Cluster Analysis and Pearson Correlation Coefficients: The HCA (hierarchical cluster analysis) results of samples and metabolites were presented as heatmaps with dendrograms, while Pearson Correlation Coefficients (PCC) between samples were calculated by the cor function in R and presented as only heatmaps. Both HCA and PCC were carried out by R package ComplexHeatmap. For HCA, normalized signal intensities of metabolites (unit variance scaling) are visualized as a color spectrum.

Differential metabolites selected: For two-group analysis, differential metabolites were determined by VIP (VIP > 1) and absolute Log_2_FC (|Log_2_FC| ≥ 1.0). VIP values were extracted from OPLS-DA result, which also contain score plots and permutation plots, was generated using R package MetaboAnalystR. The data was logging transform (log_2_) and mean centering before OPLS-DA. In order to avoid overfitting, a permutation test (200 permutations) was performed.

KEGG annotation and enrichment analysis: Identified metabolites were annotated using KEGG Compound database (http://www.kegg.jp/kegg/compound/), annotated metabolites were then mapped to KEGG Pathway database (http://www.kegg.jp/kegg/pathway.html). Pathways with significantly regulated metabolites mapped to were then fed into MSEA (metabolite sets enrichment analysis), their significance was determined by hypergeometric test's *p*-values.

## 3 Results and discussion

### 3.1 Sensory evaluation analysis of EUL with different processing techniques

**Fig.1:**
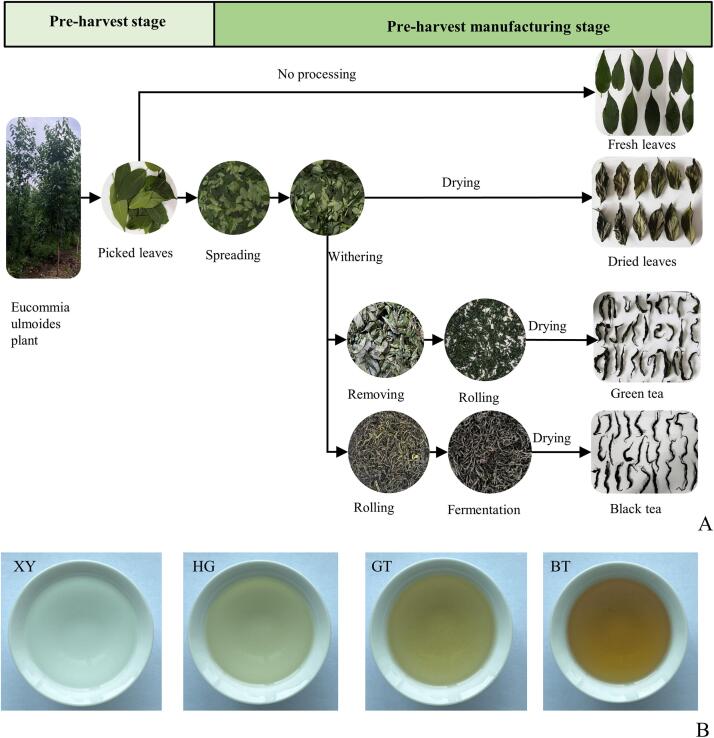
A Flow chart of different process preparation of EUL; B Brewing soup color of different samples.

The operating procedure and different product of EUL is shown in Fig.1A. The brewing soup color of each sample is shown in Fig.1B. The corresponding scoring is detailed in Table 1. The investigation revealed that BT processing had the most pronounced impact on the color and flavor profile of EUL tea. The soup brewed directly from fresh EUL exhibited a shiny of color and a refreshing taste. In contrast, the HG process yielded a tea of light-yellow hue with an astringent taste. Meanwhile, the GT processing resulted in a tea of apricot color and a noticeable bitter taste.

**Table 1:** Total score table of different processed *Eucommia ulmoides* leaves (EUL), (including Contour, Liquor color, Aroma, Taste, Infused leaf, Total points, Ranking).

Item	Contour	####	Liquor colo	####	Aroma	####	Taste	-30 %	Infused leaf	-10 %	Total points	Ranking
	Evaluation	Score	Evaluation	Score	Evaluation	Score	Evaluation	Score	Evaluation	Score		
XY	Sturdy、bloom	21.1	Shiny	8.46	Slightly grass odour	21.1	Grass taste、pale and watery	25.9	Far and tender	8.2	85.4	1
HG	Flat、dry	20.8	Light yellow	7.7	Grass odour	20.5	Grassy and astringent	24.6	Even	7.23	80.9	2
GT	Slightly tight、dull green	22.3	Apricot	7.93	Grass odour	19.9	Grassy taste 、slightly bitter	22	Open	7.4	79.6	3
BT	Tight curled	21.3	red and clear	8.7	Strong grass odour	18.9	Strong grassy taste、slightly pungent taste	22.4	Slightly open	7.67	78.9	4

### 3.2 Non-volatile metabolite profiles and dynamics of XY, HG, BT, GT

**Fig. 2:**
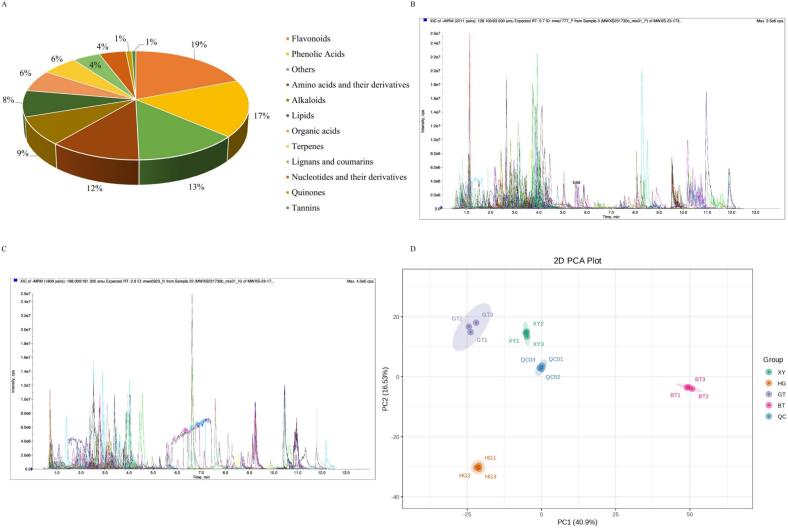
A show a pie chart of the distribution of nonvolatile metabolites; B and C show the superimposed analysis of the total ion current maps of the QC samples in positive and negative ion modes; D shows the PCA scores of the nonvolatile.

In this study, a wide range of targeted metabolomics methods was employed to monitor the XY, HG, BT, and GT samples. In total, 1839 nonvolatile metabolites (12 subclasses) were structurally detected or annotated, and identified using the Metware database. (Fig.2 A, Table 2) Among them are 352 flavonoids (19 %), 313 phenolic acids (17 %), 216 amino acids (12 %) and their derivatives, 157 alkaloids (9 %), 144 lipids (8 %), 117 organic acids (6 %), 105 terpenoids (6 %), 81 lignans and coumarins, 78 nucleotides and their derivatives, 17 quinones, 12 tannin and 247 other metabolites (including alcohols, lactones, aldehydes, chromone, sugars, ketones, vitamins, stilbenes and others). As shown in Fig.2B and 2C the superposition analysis of the total ion current maps of Quality Control Sample(*QC*)samples in positive and negative ion modes showed good reproducibility.

**Table 2:** Summary table of non-volatile metabolite compositions, classifications and relative content of substances in different processed EUL.

Index	Compounds	Class
s	1,4-Dihydro-1-Methyl-4-oxo-3-pyridinecarboxamide	Alkaloids
MWS0687	1-Methyl-6-Oxo-1,6-Dihydropyridine-3-Carboxamide	Alkaloids
pmf0290	2-Hydroxypyridine	Alkaloids
MWS1787	2-Picoline; 2-Methylpyridine	Alkaloids
pme1738	3-Carbamyl-1-methylpyridinium;(1-Methylnicotinamide)	Alkaloids
pma6298	3-Hydroxypyridine	Alkaloids
Zblp001009	3-pyridine-methanol-O-β-d-glucopyranosyl	Alkaloids
Wayp004564	4-hydroxy-4-(3-pyridyl)-butanoic acid	Alkaloids
pmf0291	4-Hydroxypyridine	Alkaloids
MWS2032	6-Methylnicotinamide	Alkaloids
MWSmce016	Nicotinic Acid Methyl Ester(Methyl Nicotinate)	Alkaloids
MWSslk076	Nicotinic acid N-oxide	Alkaloids
MWS3270	Quinolinic Acid	Alkaloids
Yacp000453	3-hydroxy-1-methylpyrrolidin-2-one*	Alkaloids
Zmsp000878	4-Hydroxy-5-(2-oxo-1-pyrrolidinyl)benzoic acid*	Alkaloids
MWStz073	5-Hydroxy-2-pyrrolidinone	Alkaloids
MWStz081	Piperlotine C; 1-(3,4,5-Trimethoxycinnamoyl)pyrrolidine	Alkaloids
MWS1860	Pyrrolidin	Alkaloids
MWSmce157	Stachydrine	Alkaloids
MWSslk106	2-Phenylethylamine	Alkaloids
MWS2076	2-Aminophenol	Alkaloids
MWSmce462	4-Hydroxybenzylamine	Alkaloids
Lmgp000796	4-Hydroxymandelonitrile	Alkaloids
MWS0435	Acetaminophen	Alkaloids
pma0101	Caffeoylagmatine	Alkaloids
pmp001244	Caffeoylcholine	Alkaloids
Hmcp009963	Candicine	Alkaloids
pmp001176	Dihydrocaffeoyltyramine	Alkaloids
MWSmce521	Dobutamine	Alkaloids
mws4002	Dopamine	Alkaloids
MWStz070	N-(2-Hydroxy-4-methoxyphenyl)acetamide	Alkaloids
Wagp001741	N-(gamma-L-glutamyl)tyramine O-glucoside	Alkaloids
pmb0492	N′,N″,N″‘-p-Coumaroyl-cinnamoyl-caffeoyl spermidine	Alkaloids
pma0702	N1,N8-Bis(sinapoyl)spermidine	Alkaloids
Zdcp003644	N-Caffeoylputrescine	Alkaloids
pmb0496	N-Feruloylagmatine	Alkaloids
Lmhp003013	N-Feruloyl-Cadaverine	Alkaloids
MWSmce098	Nonivamide	Alkaloids
pmb0490	p-Coumaroylputrescine	Alkaloids
Lmqp002784	Salicylamide	Alkaloids
pmp001214	Sinapine	Alkaloids
NK10246260	2,4-Dihydroxyquinoline	Alkaloids
Lahp002608	3,5-Dihydro-2H-Furo[3,2-*C*]Quinolin-4-One*	Alkaloids
Ladp002935	3-quinolinecarboxylic acid	Alkaloids
Lmgp001898	4,6-Dihydroxyquinoline	Alkaloids
Ladp002110	4-hydroxy-2-oxo-1,2-dihydroquinoline-3-carboxylic acid	Alkaloids
Lmlp002044	8-Hydro-4,7-dimethoxyfuranaquinoline	Alkaloids
Wcjp002598	8-hydroxyquinoline	Alkaloids
MWSslk057	9-Aminoacridine	Alkaloids
mws0393	Quinine	Alkaloids
Wasn002329	Xanthurenic Acid 8-O-Glucoside	Alkaloids
MWStz063	2-Ethyl-2,6,6-trimethylpiperidin-4-one	Alkaloids
MWSmce460	2-Piperidone	Alkaloids
pmp001198	6-Deoxyfagomine	Alkaloids
MWSmce338	N-Hydroxypipecolic acid	Alkaloids
pmb0782	Piperidine	Alkaloids
pmb1912	10-Formyltetrahydrofolic Acid	Alkaloids
MWS2050	1-Aminopropan-2-ol*	Alkaloids
MWS4401	1-Methylguanidine	Alkaloids
mws0982	1-Methylhistamine	Alkaloids
Lmxn006423	2(3H)-Benzothiazolone	Alkaloids
MWStz152	2-(Acetylamino)-3-phenyl-2-propenoic acid*	Alkaloids
MWSmce645	2-(Aminooxy)Acetic Acid	Alkaloids
MWSslk118	2,2’-Cyclouridine	Alkaloids
MWS1855	2,5-Dimethyl pyrazine	Alkaloids
MWS2063	2,6-Dimethylaniline	Alkaloids
MWSmce557	2-Acetyl-3-ethylpyrazine	Alkaloids
MWS1936	2-Methyl-5-nitroimidazole-1-ethanol	Alkaloids
Hmgp002327	3-amino-2-naphthoic acid*	Alkaloids
MWS1777	3-Chloroaniline	Alkaloids
MWSmce128	4(3H)-Quinazolinone	Alkaloids
Hmtp000776	4,5,6-Trihydroxy-2-cyclohexen-1-ylideneacetonitrile	Alkaloids
Wccp001401	4-[2-formyl-5-(hydroxymethyl)pyrrol-1-yl]butanoic acid	Alkaloids
Lamp000484	4-Methylazetidine-2-Carboxylic acid*	Alkaloids
pma3649	5-Aminolevulinic Acid*	Alkaloids
MWS2072	5-Nitrobenzimidazole	Alkaloids
Wbjp004368	8-Hydroxy-harmine	Alkaloids
MWSmce207	Acetylpyrazine	Alkaloids
pmb0501	Agmatine	Alkaloids
MWS3105	Aniline	Alkaloids
MWSmce331	Azetidine-2-carboxylic acid*	Alkaloids
pmb0069	Benzamide	Alkaloids
MWSmce548	Betaine	Alkaloids
MWS1792	Butylamine	Alkaloids
pme1841	Cadaverine	Alkaloids
mws2218	Caffeine	Alkaloids
pmb0484	Choline	Alkaloids
MWS1784	Cyclohexylamine	Alkaloids
MWSmce526	Daminozide	Alkaloids
MWSslk100	Diphenylamine	Alkaloids
mws1346	DL-2-Aminoadipic acid*	Alkaloids
pme0066	Guanidinoacetate	Alkaloids
Wcdp010864	Hexadecanamide	Alkaloids
pmp001269	Hexadecyl ethanolamine	Alkaloids
pma2987	Histidinol	Alkaloids
MWSmce448	Imidazol-1-yl-acetic acid*	Alkaloids
MWSmce118	Imidazole-4-Acetic Acid*	Alkaloids
MWSmce461	L-Azetidine-2-carboxylic acid*	Alkaloids
pme1002	L-Tyramine	Alkaloids
mws1383	Lumichrome	Alkaloids
Zasp102439	*m*-Aminophenylacetylene	Alkaloids
Zahp014087	N-(12-methyltetradecyl)propionamide*	Alkaloids
Lmmp002080	N-(4-Aminobutyl)benzamide	Alkaloids
Zajp000573	N-(4-oxopentyl)-acetamide*	Alkaloids
Wbjp002336	N,N-diethyl-5-hydroxytryptamineoxalate	Alkaloids
MWS3020	N-Acetylcadaverine	Alkaloids
pme2693	N-Acetylputrescine	Alkaloids
Smcp000882	N-benzoyl-2-aminoethyl-β-D-glucopyranoside	Alkaloids
HJKP000649	N-benzylformamide	Alkaloids
pmp001287	*N*-Benzylmethylene isomethylamine	Alkaloids
MWS0700	Neopterin	Alkaloids
pmp000476	N-Isobutyl-4,5-epoxy-2E-decaenamide	Alkaloids
MWSmce571	N-Methylbenzylamine	Alkaloids
Qmdp090606	N-Methyltetrahydropalmatine	Alkaloids
mws0983	N-Oleoylethanolamine	Alkaloids
Zmpp000906	Norepinephrine	Alkaloids
MWSslk108	O-Acetyl-l-carnitine	Alkaloids
Wbjp001169	o-Carboxy-5-hydroxytryptamine	Alkaloids
Zahp013600	Octadec-2-enamide*	Alkaloids
Wccp011838	Octadec-8-enamide*	Alkaloids
Zahp012577	Octadecadienamide	Alkaloids
pmb1754	O-Phosphocholine	Alkaloids
pma0948	Phenylethanolamine	Alkaloids
pme2292	Putrescine	Alkaloids
mws0017	Spermidine	Alkaloids
mws0018	Spermine	Alkaloids
Hamp012107	Stearamide*	Alkaloids
Wcfp007836	Tetradecyldiethanolamine	Alkaloids
MWS1919	Thiazole	Alkaloids
MWS0812	Trimethylamine N-Oxide*	Alkaloids
Wbmp002283	Α-hydroxyquinoline	Alkaloids
Cmyp003522	3’-Hydroxy-*N*-methylcoclaurine	Alkaloids
MWSmce709	Isoquinoline	Alkaloids
MWStz108	1-Ethoxycarbonyl-β-Carboline	Alkaloids
Zmbp002538	1-Methoxy-indole-3-acetamide*	Alkaloids
MWStz282	3-Hydroxy-3-acetonyloxindole*	Alkaloids
pmb0819	3-Indoleacetonitrile	Alkaloids
Hmmp001310	3-Indoleacrylic acid*	Alkaloids
pme2244	3-Indolepropionic acid	Alkaloids
pmc0682	4-Aminoindole	Alkaloids
mws0597	5-Hydroxyindole-3-acetic acid	Alkaloids
pme2836	5-Hydroxytryptophol	Alkaloids
mws0333	5-Methoxytryptamine	Alkaloids
Lmyn002540	Dioxindole-3-acetyl-3-O-glucoside	Alkaloids
pmb1096	Indole	Alkaloids
pme1651	Indole-3-acetic acid (IAA)	Alkaloids
mws0103	Indole-3-carboxaldehyde	Alkaloids
mws1417	Indole-3-carboxylic acid*	Alkaloids
mws0102	Indole-5-carboxylic acid*	Alkaloids
pmb0818	Methoxyindoleacetic acid	Alkaloids
Hmyp002656	Methyl dioxindole-3-acetate	Alkaloids
pmb0769	N-(p-Coumaroyl)serotonin Glucoside	Alkaloids
mws0677	N-Acetyl-5-hydroxytryptamine	Alkaloids
mws0620	N-Methyltryptamine	Alkaloids
MWSmce314	Oxindole	Alkaloids
mws0005	Tryptamine	Alkaloids
MWStz201	β-Carboline-1-propanoic acid	Alkaloids
Zbqn002320	(4-(2-oxopropoxy)phenyl)-L-alanine	Amino acids and derivatives
MWS00330g	1-Amino-1-cyclobutane-carboxylic-acid*	Amino acids and derivatives
MWS0933	1-Methylhistidine*	Amino acids and derivatives
Zmmp001941	2-Amino-3,4-dihydroxybutanoic acid-3-O-arabinoside	Amino acids and derivatives
MWS04528	2-Amino-4-sulfinobutanoic acid	Amino acids and derivatives
Zmdp000441	3-(Allylsulphinyl)-L-alanine	Amino acids and derivatives
MWS04491	3,4-Dehydro-DL-proline	Amino acids and derivatives
MWS1039a	3-Amino-2-methylpropanoic acid	Amino acids and derivatives
pme2914	3-Hydroxy-3-methylpentane-1,5-dioic acid	Amino acids and derivatives
MWS04559g	3-Hydroxy-*L*-phenylalanine*	Amino acids and derivatives
pme0181	3-Methyl-L-Histidine*	Amino acids and derivatives
MWS2010	3-nitro-L-tyrosine	Amino acids and derivatives
Zbqp003444	4-amino-5-oxo-5-(pentylamino)pentanoic acid	Amino acids and derivatives
pme2758	4-Hydroxy-L-glutamic acid	Amino acids and derivatives
MWSmce190	4-Hydroxy-L-Isoleucine	Amino acids and derivatives
mws3109	4-Hydroxy-*L*-phenylglycine	Amino acids and derivatives
pme2566	5-L-Glutamyl-L-amino acid	Amino acids and derivatives
Zbqp000949	5-methyl 1-propyl L-glutamate	Amino acids and derivatives
mws0263	5-Oxo-L-Proline*	Amino acids and derivatives
MWS0813	5-Oxoproline*	Amino acids and derivatives
MWS201401	Ala-Asn	Amino acids and derivatives
pme3351	Allysine(6-Oxo DL-Norleucine)	Amino acids and derivatives
MWS201384	Arg-Gly	Amino acids and derivatives
Zmdp000292	Arginine methyl ester*	Amino acids and derivatives
MWS201387	Asn-Arg	Amino acids and derivatives
MWS201389	Asn-Gln	Amino acids and derivatives
MWS201388	Asn-Hyp	Amino acids and derivatives
MWS201391	Asn-Ile	Amino acids and derivatives
MWS201449	Asn-Leu	Amino acids and derivatives
MWS201408	Asp-Glu	Amino acids and derivatives
MWS201455	Asp-Lys*	Amino acids and derivatives
pmf0470	cis-4-Hydroxy-D-proline*	Amino acids and derivatives
MWStz261	Cyclo(D-Leu-L-Pro)*	Amino acids and derivatives
MWStz205	Cyclo(D-Phe-L-Pro)*	Amino acids and derivatives
MWStz083	Cyclo(D-Val-L-Pro)	Amino acids and derivatives
Zazp002547	cyclo-(Gly-Phe)	Amino acids and derivatives
MWStz091	Cyclo(L-Ala-L-Pro)	Amino acids and derivatives
MWStz170	Cyclo(L-Leu-trans-4-hydroxy-L-Pro)	Amino acids and derivatives
MWStz211	Cyclo(L-Phe-trans-4-hydroxy-L-Pro)	Amino acids and derivatives
Lmrj002793	Cyclo(Phe-Glu)	Amino acids and derivatives
Lmhp001430	Cyclo(Pro-Glu)	Amino acids and derivatives
Lmhp002764	Cyclo(Pro-Leu)*	Amino acids and derivatives
MWStz255	Cyclo(Pro-Phe)*	Amino acids and derivatives
Lmrj002244	Cyclo(Pro-Pro)	Amino acids and derivatives
Lmrj002698	Cyclo(Pro-Val)	Amino acids and derivatives
Lmrj001341	Cyclo(Ser-Pro)	Amino acids and derivatives
ML10181668	Cycloleucine	Amino acids and derivatives
MWS0611	D-Alanyl-D-Alanine*	Amino acids and derivatives
MWS04555g	D-Allo-Isoleucine*	Amino acids and derivatives
MWS1926	DL-Leucine*	Amino acids and derivatives
mws0224	DL-Methionine	Amino acids and derivatives
MWS00275g	DL-O-tyrosine	Amino acids and derivatives
MWSmce056	DL-Tryptophan*	Amino acids and derivatives
mws4532g	D-Ornithine	Amino acids and derivatives
MWS201444	Gln-Gly	Amino acids and derivatives
MWS201469	Glu-Arg	Amino acids and derivatives
MWS201465	Glu-Phe	Amino acids and derivatives
MWS20672	Glu-Phe-Ala	Amino acids and derivatives
MWS4296	Glycylphenylalanine*	Amino acids and derivatives
MWS4309	Glycyl-tryptophan	Amino acids and derivatives
MWS201428	Gly-Glu	Amino acids and derivatives
MWS20645	Gly-Tyr*	Amino acids and derivatives
pme2853	Hexanoyl-L-glycine	Amino acids and derivatives
Zbqp002414	h-gamma-Glu-leu-oh	Amino acids and derivatives
MWS201405	His-Tyr	Amino acids and derivatives
MWS04447	Homoproline	Amino acids and derivatives
MWS201398	Hyp-Ser	Amino acids and derivatives
MWS201397	Hyp-Val	Amino acids and derivatives
MWS201424	Ile-Asn	Amino acids and derivatives
pme2074	Jasmonoyl-L-Isoleucine	Amino acids and derivatives
MWS00411g	L-2-Aminoadipate*	Amino acids and derivatives
pme0128	L-Alanyl-L-Alanine*	Amino acids and derivatives
mws4176	L-Alanyl-L-Phenylalanine	Amino acids and derivatives
pme2679	L-Allo-isoleucine	Amino acids and derivatives
mws0260	L-Arginine	Amino acids and derivatives
mws0001	L-Asparagine	Amino acids and derivatives
mws0219	L-Aspartic Acid*	Amino acids and derivatives
mws0629	L-Aspartyl-L-Phenylalanine	Amino acids and derivatives
pme0116	L-Carnosine	Amino acids and derivatives
pme0008	L-Citrulline	Amino acids and derivatives
Hmsp000364	L-Cyclopentylglycine	Amino acids and derivatives
pme2773	L-Cystathionine	Amino acids and derivatives
mws0875	L-Cysteinyl-L-glycine	Amino acids and derivatives
mws0221	L-Cystine	Amino acids and derivatives
Lmbp001216	L-Dihomomethionine	Amino acids and derivatives
MWS201461	Leu-Arg	Amino acids and derivatives
MWS201437	Leu-Asp	Amino acids and derivatives
pme0014	L-Glutamic acid	Amino acids and derivatives
pme0193	l-Glutamine	Amino acids and derivatives
mws0217	L-Glycine	Amino acids and derivatives
mws5041	L-Glycyl-L-isoleucine*	Amino acids and derivatives
mws5042	L-Glycyl-*L*-phenylalanine*	Amino acids and derivatives
pme0124	L-Glycyl-L-proline	Amino acids and derivatives
mws0254	L-Histidine	Amino acids and derivatives
mws0193	L-Homocitrulline	Amino acids and derivatives
pme0057	L-Homocysteine	Amino acids and derivatives
pme2890	L-Homocystine	Amino acids and derivatives
Lmbp000123	L-Homomethionine	Amino acids and derivatives
MWS00123g	*L*-Homophenylalanine	Amino acids and derivatives
mws0671	L-Homoserine*	Amino acids and derivatives
mws0258	L-Isoleucine*	Amino acids and derivatives
Lmrj002087	L-Isoleucyl-l-Aspartate	Amino acids and derivatives
MWS00327g	L-Isoserine	Amino acids and derivatives
mws0227	L-Leucine*	Amino acids and derivatives
Lmhp002031	L-Leucyl-L-Leucine	Amino acids and derivatives
mws5035	L-Leucyl-*L*-phenylalanine	Amino acids and derivatives
pme0026	l-Lysine	Amino acids and derivatives
pmb0962	l-Lysine-Butanoic Acid	Amino acids and derivatives
pme1210	L-Methionine	Amino acids and derivatives
pme1419	L-Methionine methyl ester	Amino acids and derivatives
pme2617	L-Methionine Sulfoxide	Amino acids and derivatives
mws1587	L-Norleucine*	Amino acids and derivatives
pme0021	L-Phenylalanine	Amino acids and derivatives
mws0636	L-Phenylalanyl-*L*-phenylalanine	Amino acids and derivatives
pme0006	L-Proline*	Amino acids and derivatives
Lmhp001461	L-Prolyl-L-Leucine	Amino acids and derivatives
Lmhp001732	L-Prolyl-L-Phenylalanine	Amino acids and derivatives
Zmzn000113	L-threo-3-Methylaspartate	Amino acids and derivatives
mws0230	L-Threonine*	Amino acids and derivatives
mws0282	L-Tryptophan	Amino acids and derivatives
MWS20627g	L-Tyrosinamide	Amino acids and derivatives
mws0250	L-Tyrosine*	Amino acids and derivatives
MWS1771	L-Tyrosine methyl ester	Amino acids and derivatives
mws0256	L-Valine	Amino acids and derivatives
Wcdp000380	L-Valinol	Amino acids and derivatives
Lmhp001670	L-Valyl-L-Leucine	Amino acids and derivatives
Lmhp002001	L-Valyl-L-Phenylalanine	Amino acids and derivatives
MWS201403	Lys-Ala	Amino acids and derivatives
MWS201411	Lys-Asn	Amino acids and derivatives
MWS201412	Lys-Asp*	Amino acids and derivatives
MWS201419	Lys-Gly	Amino acids and derivatives
MWS201464	Lys-Phe	Amino acids and derivatives
MWS201436	Lys-Thr	Amino acids and derivatives
MWS201471	Lys-Tyr	Amino acids and derivatives
pmb0034	L-α-Glutamyl-L-Glutamic Acid	Amino acids and derivatives
Zmdp002216	L-γ-Glutamyl-L-leucine	Amino acids and derivatives
MWS201470	Met-Arg	Amino acids and derivatives
MWS201399	Met-Asn	Amino acids and derivatives
MWSmce585	Methyl 3-aminopropanoate	Amino acids and derivatives
pme0109	Methyldopa	Amino acids and derivatives
MWS201421	Met-Phe	Amino acids and derivatives
MWS201429	Met-Ser	Amino acids and derivatives
MWS201430	Met-Thr	Amino acids and derivatives
Wayn001257	N-(1-Deoxy-1-fructosyl)Leucine	Amino acids and derivatives
Wayp001024	N-(1-Deoxy-1-fructosyl)Valine	Amino acids and derivatives
MWS1933	N-(2-Methylbenzoyl)glycine	Amino acids and derivatives
mws0124	N-(3-Indolylacetyl)-L-alanine	Amino acids and derivatives
MWS04412	N(6),N(6)-Dimethyl-l-lysine	Amino acids and derivatives
Zbqn004590	N-(acetyl)phenylalanine	Amino acids and derivatives
MWS5164	*N*,*N*′-Dimethylarginine;SDMA*	Amino acids and derivatives
pme3033	*N*,*N*-Dimethylglycine*	Amino acids and derivatives
Smjp002269	N-[3-(4-Hydroxyphenyl)acryloyl]-L-tyrosine	Amino acids and derivatives
MWS20987	N5-(1-Iminoethyl)-L-ornithine	Amino acids and derivatives
pme0122	N6-Acetyl-l-lysine	Amino acids and derivatives
MWSslk120	N-Acetyl-DL-phenylalanine	Amino acids and derivatives
pme0170	N-Acetyl-L-Arginine	Amino acids and derivatives
pme0075	N-Acetyl-L-glutamic acid	Amino acids and derivatives
pme0137	N-Acetyl-l-Glutamine	Amino acids and derivatives
pme0253	N-Acetyl-L-leucine	Amino acids and derivatives
MWSslk140	N-Acetyl-L-Methionine	Amino acids and derivatives
Zmgn002106	N-Acetyl-*L*-phenylalanine	Amino acids and derivatives
pme3382	N-Acetyl-L-threonine	Amino acids and derivatives
pmb2591	N-Acetyl-L-Tryptophan	Amino acids and derivatives
mws0520	N-Acetyl-L-tyrosine	Amino acids and derivatives
MWS4471	N-Alpha-Acetyl-L-Asparagine	Amino acids and derivatives
Zbqn003921	N-carboxy-N-(2-oxo-2-phenylethyl)-L-alanine	Amino acids and derivatives
MWS00204g	N-Ethylglycine*	Amino acids and derivatives
NK10251888	NG,NG-Dimethyl-L-arginine*	Amino acids and derivatives
mws0736	N-Glycyl-L-leucine*	Amino acids and derivatives
Lmqp000427	N-Methyl-Trans-4-Hydroxy-L-Proline	Amino acids and derivatives
MWS5209	N-Methyl-α-aminoisobutyric acid	Amino acids and derivatives
Zmjp000182	*N*-Monomethyl-L-arginine*	Amino acids and derivatives
MWS3166	N-Palmitoylglycine	Amino acids and derivatives
Zmyn000155	N-α-Acetyl-L-ornithine	Amino acids and derivatives
MWS201054	O-Acetyl-L-homoserine	Amino acids and derivatives
mws1050	O-Acetylserine	Amino acids and derivatives
MWS20633g	O-phosphate-L-tyrosine	Amino acids and derivatives
mws3133	Oxaceprol	Amino acids and derivatives
mws4134	Oxiglutatione	Amino acids and derivatives
MWS201439	Phe-His	Amino acids and derivatives
MWS201458	Phe-Ile	Amino acids and derivatives
mws0715	Phenylacetyl-l-glutamine	Amino acids and derivatives
MWS201478	Phe-Ser	Amino acids and derivatives
MWS201442	Phe-Thr	Amino acids and derivatives
MWS201414	Pro-Asn	Amino acids and derivatives
MWS201415	Pro-Asp	Amino acids and derivatives
Zbqn001255	propyl-L-alanine	Amino acids and derivatives
MWS201466	Pro-Trp	Amino acids and derivatives
mws4516	Pyroglutamic acid	Amino acids and derivatives
Zmdp000976	S-(2-Carboxypropyl)cysteine	Amino acids and derivatives
pme1286	S-(5′-Adenosy)-L-homocysteine	Amino acids and derivatives
MWStz103	S-(5′-Adenosyl)-L-methionine	Amino acids and derivatives
mws0582	S-(Methyl)glutathione	Amino acids and derivatives
MWS201440	Ser-Glu	Amino acids and derivatives
MWS201443	Ser-Lys	Amino acids and derivatives
MWS201381	Ser-Trp	Amino acids and derivatives
Lcsp002417	Ser-Val-Leu	Amino acids and derivatives
Zmdp000972	S-Methyl-L-cysteine	Amino acids and derivatives
MWS20966	*S*-methyl-L-thiocitrulline	Amino acids and derivatives
MWS201477	Thr-Thr	Amino acids and derivatives
mws0216	Trans-4-Hydroxy-L-proline*	Amino acids and derivatives
pmp001257	Tridecanoylglycine	Amino acids and derivatives
Zmzp000145	Trimethyllysine	Amino acids and derivatives
MWS201187	Trp-His	Amino acids and derivatives
MWS201480	Tyr-Ala	Amino acids and derivatives
MWS201479	Tyr-Gly*	Amino acids and derivatives
MWS201451	Val-His	Amino acids and derivatives
MWS201400	Val-Trp	Amino acids and derivatives
Zbqp003189	Val-Val	Amino acids and derivatives
Zmdp001647	γ-Glutamyl-L-valine	Amino acids and derivatives
Zmdp001663	γ-glutamylmethionine	Amino acids and derivatives
Zmdn001564	γ-Glutamylphenylalanine	Amino acids and derivatives
Zmdp001857	γ-Glutamyltyrosine	Amino acids and derivatives
Zmmp003443	γ-Glu-Trp	Amino acids and derivatives
Zmdp001718	γ-L-Glutamyl-S-allyl-L-cysteine	Amino acids and derivatives
Zmdp000446	γ-L-Glutamyl-*S*-methyl-L-cysteine	Amino acids and derivatives
mws1068	Kaempferol (3,5,7,4’-Tetrahydroxyflavone)	Flavonoids
MWSHY0136	Kaempferol-3-O-glucoside (Astragalin)*	Flavonoids
pme2954	Quercetin	Flavonoids
mws0091	Quercetin-3-O-glucoside (Isoquercitrin)	Flavonoids
mws0045	Quercetin-3-O-rhamnoside(Quercitrin)	Flavonoids
MWSHY0067	Quercetin-3-O-rutinoside (Rutin)*	Flavonoids
MWSHY0065	Catechin*	Flavonoids
Lajp002810	Procyanidin A4	Flavonoids
Lmsp004137	3,4,2′,4′,6’-Pentahydroxychalcone	Flavonoids
Wcfp003437	3-Prenyl-4,2′,4′-Trihydroxychalcone	Flavonoids
pmn001716	Carthamone*	Flavonoids
HJN055	Dihydrocharcone-4’-O-glucoside*	Flavonoids
Labn004865	Hydroxy isoliquiritigenin glucoside*	Flavonoids
Lasp002993	Isobavachalcone glucoside	Flavonoids
Lmlp006175	Isosalipurposide (Phlorizin Chalcone)	Flavonoids
pme2960	Naringenin chalcone; 2′,4,4′,6’-Tetrahydroxychalcone*	Flavonoids
Cmxp005429	Okanin	Flavonoids
zjgp122004	Okanin-3’-O-β-*D*-glucoside*	Flavonoids
Cmxp003975	Okanin-4’-O-glucoside(Marein)*	Flavonoids
pme1201	Phloretin	Flavonoids
mws2118	Phloretin-2’-O-glucoside (Phlorizin)	Flavonoids
Zbpn005555	Phloretin-4’-O-glucoside (Trilobatin)*	Flavonoids
Hmmn003343	Sappanchalcone	Flavonoids
Hmpn005101	Sieboldin	Flavonoids
Zbbp005255	Aureusidin	Flavonoids
Cmxn006627	Maritimetin	Flavonoids
Jmgn005927	2-hydroxynaringenin	Flavonoids
Lmsp004301	3′,5,5′,7-Tetrahydroxyflavanone-7-O-glucoside*	Flavonoids
Hmqn003268	5,7,3′,4′,5’-Pentahydroxydihydroflavone	Flavonoids
Jmgn004021	6-C-Glucosyl-2-Hydroxynaringenin	Flavonoids
Zbsp007084	Butin; 7,3′,4′-Trihydroxyflavanone*	Flavonoids
HJN090	Butin-7-O-glucoside*	Flavonoids
MWSslk252	Didymin (Isosakuranetin-7-O-rutinoside)*	Flavonoids
Hmhp005335	Dihydrobaicalein	Flavonoids
mws0064	Eriodictyol (5,7,3′,4’-Tetrahydroxyflavanone)*	Flavonoids
HJN086	Eriodictyol-3’-O-glucoside*	Flavonoids
MWS20145	Eriodictyol-7-O-glucoside*	Flavonoids
MWSHY0092	Eriodictyol-7-O-Rutinoside (Eriocitrin)	Flavonoids
pmb3023	Eriodictyol-8-C-glucoside*	Flavonoids
pmb0628	Eriodictyol-8-C-glucoside-4’-O-glucoside	Flavonoids
Lmqn009304	Eucalyptin (5-Hydroxy-7,4′-dimethoxy-6,8-dimethylflavone)	Flavonoids
mws0463	Hesperetin	Flavonoids
pmb2970	Hesperetin-5,7-di-O-glucoside	Flavonoids
pme1598	Hesperetin-5-O-glucoside	Flavonoids
Lmzp002365	Hesperetin-7-O-glucoside	Flavonoids
MWSHY0019	Hesperetin-7-O-neohesperidoside(Neohesperidin)*	Flavonoids
MWSHY0116	Hesperetin-7-O-rutinoside (Hesperidin)*	Flavonoids
Lahp003599	Homoeriodictyol-7,4′-di-O-β-D-glucopyranoside	Flavonoids
pme1611	Isohemiphloin	Flavonoids
Labp005025	Malonyl isoSakuranin	Flavonoids
MWSHY0017	Naringenin (5,7,4′-Trihydroxyflavanone)*	Flavonoids
HJN087	Naringenin-4’-O-glucoside*	Flavonoids
pma0724	Naringenin-6-C-Glucoside	Flavonoids
pmp000394	Naringenin-7-O-(2”-O-apiosyl)glucoside	Flavonoids
pma0791	Naringenin-7-O-(6″-malonyl)glucoside	Flavonoids
mws1179	Naringenin-7-O-glucoside (Prunin)*	Flavonoids
mws0046	Naringenin-7-O-Neohesperidoside(Naringin)*	Flavonoids
mws1066	Naringenin-7-O-Rutinoside(Narirutin)*	Flavonoids
Lmzn001875	Naringenin-7-O-Rutinoside-4’-O-glucoside	Flavonoids
mws1454	Persicoside	Flavonoids
mws0789	Pinocembrin (Dihydrochrysin)	Flavonoids
HJN078	Pinocembrin-7-O-(2”-O-arabinosyl)glucoside	Flavonoids
HJAP135	Pinocembrin-7-O-(6”-O-malonyl)glucoside	Flavonoids
Lmyp004617	Pinocembrin-7-O-glucoside (Pinocembroside)*	Flavonoids
HJN076	Pinocembrin-7-O-neohesperidoside	Flavonoids
mws0791	Poncirin (Isosakuranetin-7-O-neohesperidoside)*	Flavonoids
mws0914	3,5,7-Trihydroxyflavanone (Pinobanksin)	Flavonoids
mws1174	3-O-Acetylpinobanksin	Flavonoids
mws1094	Aromadendrin (Dihydrokaempferol)	Flavonoids
Lmtn002796	Aromadendrin-7-O-glucoside*	Flavonoids
Cmsp004086	Dihydrokaempferide	Flavonoids
Lmmn004625	Dihydrokaempferol-7-O-glucoside*	Flavonoids
HJN104	Dihydromyricetin-3-O-glucoside	Flavonoids
mws0044	Taxifolin(Dihydroquercetin)	Flavonoids
Zbxn003562	Taxifolin-2-O-glucoside	Flavonoids
Xmsn002700	Taxifolin-3’-O-glucoside	Flavonoids
mws1361	Taxifolin-3-O-rhamnoside (Astilbin)	Flavonoids
MWSHY0130	Taxifolin-7-O-rhamnoside	Flavonoids
Lmdp003110	2,6,7,4’-Tetrahydroxyisoflavanone	Flavonoids
Lmqn006260	2′,3′,4′,5,7-Pentahydroxyflavone*	Flavonoids
Zajp007311	3′,4′,5′,5,7-Pentamethoxyflavone*	Flavonoids
Lmtp003948	3′,5′,5,7-Tetrahydroxy-4′-methoxyflavanone-3’-O-glucoside	Flavonoids
Wagp009079	3,5,6,7,8,4’-Hexamethoxyflavone	Flavonoids
Zbqn006065	3,5,7,2’-Tetrahydroxyflavone; Datiscetin*	Flavonoids
MWSslk146	4’-O-Glucosylvitexin	Flavonoids
Zmjp004875	5,6,3′,4’-Tetrahydroxy-3,7-dimethoxyflavone	Flavonoids
Zmjp004852	5,6,3′,4’-Tetrahydroxy-3,7-dimethoxyflavone-6-O-glucoside*	Flavonoids
Zmhn003257	5,7,2′-Trihydroxy-8-methoxyflavone*	Flavonoids
Hmmp007513	5,7-Dihydroxy-6,3′,4′,5′-tetramethoxyflavone (Arteanoflavone)*	Flavonoids
Zmhp003514	6,7,8-Tetrahydroxy-5-methoxyflavone	Flavonoids
Zmyn003693	6-Hydroxy-2′-methoxyflavone	Flavonoids
Hmcn002875	6-Hydroxyluteolin	Flavonoids
Hmcn001884	6-Hydroxyluteolin 5-glucoside*	Flavonoids
MWS20151	Apigenin; 4′,5,7-Trihydroxyflavone	Flavonoids
MWS20148	Apigenin-4’-O-glucoside*	Flavonoids
Lmtp002942	Apigenin-6,8-di-C-arabinoside*	Flavonoids
mws1073	Apigenin-6,8-di-C-glucoside (Vicenin-2)	Flavonoids
Lmtp002474	Apigenin-6-C-(2″-glucosyl)arabinoside	Flavonoids
pmp000237	Apigenin-6-C-(2″-glucuronyl)xyloside	Flavonoids
Lmnp102580	Apigenin-6-C-(2″-xylosyl)glucoside*	Flavonoids
Lmtp002822	Apigenin-6-C-arabinoside-8-C-xyloside*	Flavonoids
MWSHY0008	Apigenin-6-C-glucoside (Isovitexin)*	Flavonoids
Hmgp003664	Apigenin-6-C-rhamnoside	Flavonoids
MWSHY0021	Apigenin-7,4′-dimethyl ether	Flavonoids
pmb0571	Apigenin-7-O-(2″-glucosyl)arabinoside	Flavonoids
pmp000585	Apigenin-7-O-(6″-malonyl)glucoside	Flavonoids
Lmpp003930	Apigenin-7-O-(6″-p-Coumaryl)glucoside	Flavonoids
MWSHY0189	Apigenin-7-O-glucoside(Cosmosiin)*	Flavonoids
pmb2991	Apigenin-7-O-glucoside-4’-O-rutinoside	Flavonoids
pme0368	Apigenin-7-O-rutinoside (Isorhoifolin)	Flavonoids
Lmtp002642	Apigenin-8-C-(2″-glucosyl)arabinoside	Flavonoids
Lmnp202580	Apigenin-8-C-(2″-xylosyl)glucoside*	Flavonoids
pmb0681	Apigenin-8-C-Arabinoside	Flavonoids
MWSHY0181	Apigenin-8-C-Glucoside (Vitexin)*	Flavonoids
Lakn002234	Catechin glucosyl glucoside	Flavonoids
Lmyp005841	Chrysin-7-O-glucoside	Flavonoids
Lazp002140	Chrysoeriol glucosyl xylosyl glucoside	Flavonoids
Zmxp002867	Chrysoeriol-5,7-di-O-glucoside	Flavonoids
pmb2999	Chrysoeriol-5-O-glucoside	Flavonoids
pmb0587	Chrysoeriol-7-O-(2”-O-glucuronyl)glucoside	Flavonoids
pmp001289	Chrysoeriol-7-O-(2”-O-glucuronyl)glucuronide	Flavonoids
Zbsn005918	Chrysoeriol-7-O-(6″-acetyl)glucoside*	Flavonoids
pmb3012	Chrysoeriol-7-O-glucoside	Flavonoids
pmb3002	Chrysoeriol-7-O-rutinoside	Flavonoids
pmb2997	Chrysoeriol-7-O-Sophoroside-5-O-glucuronide	Flavonoids
Zmjp009119	Chrysosplenetin (5,4’-Dihydroxy-3,6,7,3′-tetramethoxyflavone)*	Flavonoids
mws0058	Diosmetin (5,7,3′-Trihydroxy-4′-methoxyflavone)*	Flavonoids
pmp000579	Diosmetin-7-O-galactoside*	Flavonoids
Hmmp004965	Diosmetin-7-O-glucoside*	Flavonoids
MWSHY0190	Diosmetin-7-O-rutinoside (Diosmin)	Flavonoids
Lmyn006227	Galangin (3,5,7-Trihydroxyflavone)	Flavonoids
Lmlp005572	Galangin-7-O-glucoside	Flavonoids
MWSslk237	Hispidulin-7-O-glucoside(Homoplantaginin)*	Flavonoids
Zbzp003056	Isoorientin-7-O-glucoside*	Flavonoids
Lmlp002990	Isosaponarin(Isovitexin-4’-O-glucoside)	Flavonoids
mws1292	Isoschaftoside	Flavonoids
Smhp004476	Isovitexin-2”-O-xyloside*	Flavonoids
Hmmp003156	Isovitexin-7-O-glucoside(Saponarin)	Flavonoids
Lmyp003837	Isovitexin-7-O-glucoside-2”-O-rhamnoside	Flavonoids
pmp000236	Isovitexin-8-O-xyloside	Flavonoids
Lmqp003887	Kaempferol-3-O-xylosyl(1 → 2)glucoside	Flavonoids
Lmfn001893	Leucocyanidin	Flavonoids
pme0088	Luteolin (5,7,3′,4’-Tetrahydroxyflavone)	Flavonoids
MWS20147	Luteolin-3’-O-glucoside	Flavonoids
Hmpp003270	Luteolin-4’-O-glucoside*	Flavonoids
HJAP010	Luteolin-6,8-di-C-arabinoside	Flavonoids
MWSHY0016	Luteolin-6-C-glucoside (Isoorientin)	Flavonoids
Lmnp002413	Luteolin-6-C-glucoside-7-O-rhamnoside	Flavonoids
Zmxp003107	Luteolin-7,3′-di-O-glucoside	Flavonoids
Zmlp003063	Luteolin-7-O-(2”-O-rhamnosyl)rutinoside*	Flavonoids
Hmjn004446	Luteolin-7-O-(6″-caffeoyl)rhamnoside	Flavonoids
pmp000587	Luteolin-7-O-(6″-malonyl)glucoside*	Flavonoids
Hmqp003184	Luteolin-7-O-(6″-malonyl)glucoside-5-O-arabinoside	Flavonoids
Zbsp004156	Luteolin-7-O-(6″-malonyl)glucoside-5-O-rhamnoside	Flavonoids
Hmpp002612	Luteolin-7-O-gentiobioside	Flavonoids
MWSHY0104	Luteolin-7-O-glucoside (Cynaroside)*	Flavonoids
MWSHY0121	Luteolin-7-O-glucuronide	Flavonoids
Lmmp003487	Luteolin-7-O-glucuronide-(2 → 1)-glucuronide	Flavonoids
MWSHY0080	Luteolin-7-O-neohesperidoside (Lonicerin)*	Flavonoids
Hmqp002870	Luteolin-7-O-Sophoroside-5-O-arabinoside	Flavonoids
HJAP012	Luteolin-8-C-arabinoside	Flavonoids
Hmlp003068	Meratin*	Flavonoids
Hmmn004152	Neoeriocitrin	Flavonoids
Hmgp002148	Nepetin-7-O-alloside*	Flavonoids
Hmgp002036	Nepetin-7-O-glucoside(Nepitrin)*	Flavonoids
mws0043	Nobiletin (5,6,7,8,3′,4’-Hexamethoxyflavone)	Flavonoids
Ybnn007052	Norartocarpetin	Flavonoids
Zmjp003031	Orientin-2”-O-galactoside*	Flavonoids
pmb0636	Orientin-7-O-arabinoside	Flavonoids
Zbjp003056	Orientin-7-O-glucoside*	Flavonoids
Lmsp004632	Scutellarein (5,6,7,4’-Tetrahydroxyflavone)	Flavonoids
mws0055	Tangeretin (4′,5,6,7,8-Pentamethoxyflavone)*	Flavonoids
mws0920	Tricetin (5,7,3′,4′,5’-Pentahydroxyflavone)	Flavonoids
Lmzp004885	Tricin (5,7,4′-Trihydroxy-3′,5′-dimethoxyflavone)	Flavonoids
Hmmp002324	Tricin-4’-O-rutinoside-7-O-rutinoside	Flavonoids
pmb3042	Tricin-5-O-Glucoside	Flavonoids
pmb0713	Tricin-7-O-(2”-O-glucosyl)glucoside	Flavonoids
pmb3045	Tricin-7-O-Glucuronide	Flavonoids
Lmgp004959	Tricin-7-O-neohesperidoside*	Flavonoids
Hmgp003086	Tricin-7-O-rutinoside*	Flavonoids
Lcyp000688	Verecundin(Pinocembrin-5-glucoside)*	Flavonoids
Zbsn003878	Vicenin-3	Flavonoids
pmp001111	Violanthin	Flavonoids
Zmjp003291	Vitexin-2”-O-galactoside	Flavonoids
pme3227	Vitexin-2”-O-rhamnoside	Flavonoids
Zmjp003463	Vitexin-2”-O-xyloside	Flavonoids
Lmcp004624	3-Methylkaempferol	Flavonoids
Lmjp003655	6-C-MethylKaempferol-3-glucoside*	Flavonoids
pma0214	6-C-Methylquercetin-3-O-glucoside*	Flavonoids
Lmmp002963	6-C-Methylquercetin-3-O-rutinoside*	Flavonoids
Zmhp102730	6-Hydroxykaempferol-3,6-O-Diglucoside	Flavonoids
pmp001312	6-Hydroxykaempferol-3,7,6-O-triglycoside	Flavonoids
Zmhp002640	6-Hydroxykaempferol-3-O-Rutinoside-6-O-glucoside*	Flavonoids
pmp001311	6-Hydroxykaempferol-6,7-O-Diglucoside	Flavonoids
pmp001309	6-Hydroxykaempferol-7-O-glucoside	Flavonoids
Lmjp003295	6-Methoxykaempferol-3-O-glucoside*	Flavonoids
mws2186	Avicularin(Quercetin-3-O-α-L-arabinofuranoside)*	Flavonoids
MWSHY0077	Fisetin	Flavonoids
Lmmp002143	Gossypetin-3-O-(6″-malonyl)glucoside*	Flavonoids
Lmmp001947	Gossypetin-3-O-glucoside*	Flavonoids
Lmmp002904	Gossypetin-3-O-glucoside-8-O-xyloside	Flavonoids
Zmhp003784	Gossypetin-7-O-(3″-glucosyl)rhamnoside; Rhodioflavonoside	Flavonoids
Zmhp004034	Gossypetin-7-O-rhamnoside; Rhodiolgin	Flavonoids
Zmhp003716	Herbacetin-7-O-rhamnoside-8-O-glucoside; Rhodionidin*	Flavonoids
pmb0645	Hesperetin-6-C-glucoside-7-O-glucoside	Flavonoids
pmb0618	Hesperetin-8-C-glucoside-3’-O-glucoside	Flavonoids
mws0066	Isorhamnetin; 3′-Methoxy-3,4′,5,7-Tetrahydroxyflavone	Flavonoids
Hmcp001578	Isorhamnetin-3,7-O-diglucoside	Flavonoids
Li512111	Isorhamnetin-3-O-(6″-acetylglucoside)	Flavonoids
Zbpp002027	Isorhamnetin-3-O-(6″-malonyl)glucoside*	Flavonoids
Hmcp001658	Isorhamnetin-3-O-(6″-malonyl)glucoside-7-O-glucoside	Flavonoids
Hmcp002316	Isorhamnetin-3-O-arabinoside	Flavonoids
Lmgp005640	Isorhamnetin-3-O-Galactoside; Cacticin	Flavonoids
Zbpp001992	Isorhamnetin-3-O-Glucoside*	Flavonoids
MWSHY0064	Isorhamnetin-3-O-neohesperidoside*	Flavonoids
Zbpp001877	Isorhamnetin-3-O-rutinoside*	Flavonoids
MWSHY0135	Isorhamnetin-3-O-rutinoside (Narcissin)*	Flavonoids
Hmmp002240	Isorhamnetin-3-O-rutinoside-7-O-(2”-O-glucosyl)glucuronate	Flavonoids
HJAP128	Isorhamnetin-3-O-rutinoside-7-O-rhamnoside	Flavonoids
Hmcp002207	Isorhamnetin-7-O-glucoside (Brassicin)*	Flavonoids
Zbjn005825	Kaempferide-3-O-(6’-*O*-acetyl)glucoside*	Flavonoids
Zmcn003728	Kaempferol-3,7-O-diglucoside	Flavonoids
MWSHY0201	Kaempferol-3,7-O-dirhamnoside (Kaempferitrin)	Flavonoids
Lmyp003500	Kaempferol-3-O-(2″-galloyl)galactoside*	Flavonoids
Lmdp004819	Kaempferol-3-O-(2″-sinapoyl)glucosyl-(1 → 2)-(6″-malonyl)glucoside	Flavonoids
Hmln001836	Kaempferol-3-O-(6”-Acetyl)glucosyl-(1 → 3)-Galactoside	Flavonoids
Lmyp003599	Kaempferol-3-O-(6″-galloyl)glucoside*	Flavonoids
Lmdp004892	Kaempferol-3-O-(6″-malonyl)galactoside*	Flavonoids
Lmmp003817	Kaempferol-3-O-(6″-malonyl)glucoside*	Flavonoids
Hmcp001629	Kaempferol-3-O-(6″-Malonyl)glucoside-7-O-Glucoside	Flavonoids
Lmmn003398	Kaempferol-3-O-(6”-*O*-acetyl)glucoside	Flavonoids
Xmyp004945	Kaempferol-3-O-(6”-Rhamnosyl-2″-Glucosyl)Glucoside (Camelliaside A)	Flavonoids
pmn001637	Kaempferol-3-O-arabinoside (Juglanin)	Flavonoids
Hmcp001858	Kaempferol-3-O-arabinoside-7-O-rhamnoside	Flavonoids
mws0913	Kaempferol-3-O-galactoside (Trifolin)*	Flavonoids
Lmsp004670	Kaempferol-3-O-glucoside-7-O-rhamnoside*	Flavonoids
Lmzn001894	Kaempferol-3-O-glucuronide	Flavonoids
Lmjp002867	Kaempferol-3-O-neohesperidoside*	Flavonoids
pmp001105	Kaempferol-3-O-neohesperidoside-7-O-glucoside*	Flavonoids
mws0919	Kaempferol-3-O-rhamnoside (Afzelin)(Kaempferin)	Flavonoids
pme1605	Kaempferol-3-O-robinobioside(Biorobin)	Flavonoids
mws1035	Kaempferol-3-O-robinoside-7-O-rhamnoside (Robinin)*	Flavonoids
MWSHY0050	Kaempferol-3-O-rutinoside(Nicotiflorin)*	Flavonoids
Lmsp003161	Kaempferol-3-O-sophoroside-7-O-rhamnoside*	Flavonoids
Lmqp002170	Kaempferol-3-O-sophorotrioside	Flavonoids
Xmyp005654	Kaempferol-4’-O-glucoside*	Flavonoids
HJAP023	Kaempferol-6,8-di-C-glucoside-7-O-glucoside	Flavonoids
mws0089	Kaempferol-7-O-glucoside*	Flavonoids
pme0321	Kaempferol-7-O-rhamnoside	Flavonoids
HJAP005	Laricitrin-3-O-glucoside*	Flavonoids
Hmcp002187	Limocitrin-3-O-galactoside*	Flavonoids
pme3514	Morin*	Flavonoids
Lmfn004065	Morin-3-O-arabinoside*	Flavonoids
Lmfn004036	Morin-3-O-lyxoside*	Flavonoids
Lmfp004055	Morin-3-O-xyloside*	Flavonoids
mws0032	Myricetin	Flavonoids
Lmtp004126	Myricetin-3-O-(6″-malony)glucoside*	Flavonoids
pmn001640	Myricetin-3-O-arabinoside	Flavonoids
Lmdp002969	Myricetin-3-O-galactoside*	Flavonoids
mws0056	Myricetin-3-O-rhamnoside (Myricitrin)	Flavonoids
mws1045	Myricetin-3-O-rutinoside	Flavonoids
Lmpp003465	Myricetin-3-O-β-D-glucoside*	Flavonoids
Zahp003364	myricetin-3-O-β-d-xylopyranosyl-(1 → 2)-β-D-glucopyranoside	Flavonoids
Lakp003108	Myricetindiglucoside	Flavonoids
Lmjp003231	Patuletin-3-O-glucoside*	Flavonoids
Hmgp001996	Quercetagetin; 3,3′,4′,5,6,7-Hexahydroxyflavone	Flavonoids
Lmfp003403	Quercetagetin-7-O-glucoside(Quercetagitrin)*	Flavonoids
Lmcp004369	Quercetin-3′,4′-dimethyl ether	Flavonoids
Zbsp004438	Quercetin-3,4’-*O*-di-glucoside	Flavonoids
Zmcp002666	Quercetin-3,7-Di-O-glucoside*	Flavonoids
Zmcp003530	quercetin-3-hydroxyferuloyldiglucoside	Flavonoids
Lmyp003588	Quercetin-3-O-(2″,6”-O-digalloyl)-glucoside	Flavonoids
pmn001644	Quercetin-3-O-(2”-*O*-acetyl)glucuronide	Flavonoids
Lmdp004221	Quercetin-3-O-(2”-O-caffeoyl)glucoside-(1 → 2)-(6″-Malonyl)glucoside	Flavonoids
pmp000596	Quercetin-3-O-(2”-O-galactosyl)glucoside	Flavonoids
Lmfp005436	Quercetin-3-O-(2”-O-galloyl)Arabinoside	Flavonoids
MWSHY0142	Quercetin-3-O-(2”-O-galloyl)galactoside	Flavonoids
Lmmp003266	Quercetin-3-O-(2”-O-malonyl)glucoside-7-O-arabinoside	Flavonoids
Lmjp003360	Quercetin-3-O-(2”-O-malonyl)sophoroside-7-O-arabinoside	Flavonoids
Lmbp002336	Quercetin-3-O-(2”-O-rhamnosyl)galactoside*	Flavonoids
HJAP127	Quercetin-3-O-(2”-O-Rhamnosyl)rutinoside	Flavonoids
Hmcp001618	Quercetin-3-O-(2”-O-Xylosyl)rutinoside	Flavonoids
Hmln002199	Quercetin-3-O-(6”-*O*-acetyl)galactoside	Flavonoids
Zmsp004363	Quercetin-3-O-(6”-*O*-acetyl)glucoside	Flavonoids
Hmln001682	Quercetin-3-O-(6”-*O*-acetyl)glucosyl-(1 → 3)-Galactoside	Flavonoids
Zbsp004136	Quercetin-3-O-(6”-O-arabinosyl)glucoside*	Flavonoids
MWSHY0199	Quercetin-3-O-(6”-O-galloyl)galactoside	Flavonoids
pmb0706	Quercetin-3-O-(6”-O-malonyl)glucosyl-5-O-glucoside	Flavonoids
Lmdp004426	Quercetin-3-O-[2”-O-(6″‘-sinapoyl)glucosyl]glucoside	Flavonoids
Lmfp002421	Quercetin-3-O-[rhamnosyl(1 → 2)glucosyl]-5-O-glucoside*	Flavonoids
Lmdp003286	Quercetin-3-O-alloside; Isohyperoside*	Flavonoids
mws4183	Quercetin-3-O-arabinoside	Flavonoids
MWSHY0113	Quercetin-3-O-galactoside (Hyperin)*	Flavonoids
Lmsp004166	Quercetin-3-O-glucoside-7-O-rhamnoside*	Flavonoids
Lmjp002461	Quercetin-3-O-neohesperidoside*	Flavonoids
Hmcp001769	Quercetin-3-O-rhamnosyl(1 → 2)arabinoside	Flavonoids
Lssp210058	Quercetin-3-O-robinobioside*	Flavonoids
Lmmp002334	Quercetin-3-O-rutinoside-7-O-glucoside*	Flavonoids
Lnrp102163	Quercetin-3-O-rutinoside-7-O-rhamnoside	Flavonoids
Lmjp002596	Quercetin-3-O-sambubioside*	Flavonoids
Lmjp003206	Quercetin-3-O-Sambubioside-5-O-Glucoside	Flavonoids
MWSHY0162	Quercetin-3-O-sophoroside (Baimaside)	Flavonoids
Lmsp002982	Quercetin-3-O-sophoroside-7-O-rhamnoside*	Flavonoids
Lmdp003509	Quercetin-3-O-xyloside (Reynoutrin)*	Flavonoids
Lmmp003306	Quercetin-3-O-xylosyl(1 → 2)arabinoside	Flavonoids
Zmgp002857	Quercetin-3-O-α-rhamnosyl (1 → 2)-[α-rhamnosyl (1 → 6)]-β-glucoside	Flavonoids
Lssp210052	Quercetin-3-rutinoside-7-galactoside	Flavonoids
mws0856	Quercetin-4’-O-glucoside (Spiraeoside)*	Flavonoids
Lmfn003760	Quercetin-4’-O-glucuronide	Flavonoids
Smgp004575	Quercetin-5-O-β-D-glucoside*	Flavonoids
Lmmp002995	Quercetin-7-O-(2″-malonyl)glucosyl-5-O-glucoside	Flavonoids
pmp000589	Quercetin-7-O-(6″-malonyl)glucoside	Flavonoids
pmb0709	Quercetin-7-O-(6″-malonyl)glucosyl-5-O-glucoside	Flavonoids
mws1329	Quercetin-7-O-glucoside	Flavonoids
Zbsp004301	Quercetin-7-O-rutinoside*	Flavonoids
Lmmp002755	Quercetin-7-O-rutinoside-4’-O-glucoside	Flavonoids
Lmjp002906	Rhamnetin-3-O-Glucoside*	Flavonoids
Zmpp002571	Robinetin	Flavonoids
pmb0565	Syringetin-3-O-glucoside	Flavonoids
mws2627	Tamarixetin (3,3′,5,7-Tetrahydroxy-4′-Methoxyflavone)	Flavonoids
Zmhp005139	Tamarixetin-3-O-(6″-malonyl)glucoside*	Flavonoids
Lahp003408	2-(3,4-dihydroxyphenyl)-4 h-chromene-3,5,7-triol-glucoside	Flavonoids
Lmmp003109	3’-O-Methyl-epicatechin	Flavonoids
pme3285	Afzelechin (3,5,7,4’-Tetrahydroxyflavan)	Flavonoids
mws0355	Catechin gallate*	Flavonoids
pmn001416	Catechin-(7,8-bc)-4α-(3,4-dihydroxyphenyl)-dihydro-2-(3H)-one	Flavonoids
pmn001415	Catechin-(7,8-bc)-4β-(3,4-dihydroxyphenyl)-dihydro-2-(3H)-one	Flavonoids
Zajn002491	catechin-4-β-D-galactopyranoside*	Flavonoids
pmb2947	Catechin-catechin-catechin	Flavonoids
mws1422	Epiafzelechin	Flavonoids
pme0460	Epicatechin*	Flavonoids
mws1397	Epicatechin gallate*	Flavonoids
HJN041	Epicatechin glucoside	Flavonoids
Zmdn002400	Epicatechin-3’-O-β-D-glucopyranoside*	Flavonoids
Zmdn002049	Epicatechin-4’-O-β-D-glucopyranoside*	Flavonoids
pmb3114	Epicatechin-epiafzelechin	Flavonoids
MWSHY0098	Epigallocatechin	Flavonoids
mws0034	Epigallocatechin-3-gallate*	Flavonoids
Lajp003377	Fisetinidol-(4α,6)-gallocatechin	Flavonoids
mws0049	Gallocatechin	Flavonoids
mws2220	Gallocatechin 3-O-gallate*	Flavonoids
Lajp002779	Gallocatechin-(4α- > 8)-Catechin-(4α- > 8)-Catechin	Flavonoids
pmb2586	Gallocatechin-(4α → 8)-catechin	Flavonoids
Lmmp000897	Gallocatechin-(4α → 8)-gallocatechin	Flavonoids
Wbtp004753	1,2,3,7,8-pentahydroxy-6-methylanthracene-9,10-dione	Flavonoids
Wbtn006715	1,2,4,5,8-pentahydroxy-6-methylanthracene-9,10-dione*	Flavonoids
Wbtp004961	1,2,4,5-tetrahydroxy-7-(hydroxymethyl)anthracene-9,10-dione	Flavonoids
Wbtp004863	4-(beta-d-Glucopyranosyl)-7-methoxy-1,3,6-trihydroxy-9H-xanthene-9-one	Flavonoids
pmn001477	4-C-Glucose-1,3,6-trihydroxy-7-methoxyxanthone	Flavonoids
Satp005088	5-hydroxy-7-(2-hydroxypropyl)-2-(3-hydroxy-2-(4-hydroxy-3,5-dimethoxybenzyl)propyl)-chromone	Flavonoids
Wbtn006810	6,7-dihydroxy-1,3-dimethoxyxanthen-9-one*	Flavonoids
Lcsn003955	Maesopsin	Flavonoids
pmn001370	Pinoresinol-4,4’-O-diglucoside	Lignans and Coumarins
pmn001369	1-Hydroxypineolin Diglucoside	Lignans and Coumarins
pmn001375	1-Hydroxypinoresinol-1-O-Glucoside	Lignans and Coumarins
Zmcn003729	1-Hydroxypinoresinol-4’-O-Glucoside	Lignans and Coumarins
Lmmn002260	5′-Methoxyisolariciresinol-9’-O-glucoside	Lignans and Coumarins
WaYn005928	Buddlenol B*	Lignans and Coumarins
WaYn006596	Buddlenol C	Lignans and Coumarins
WaYn006188	Buddlenol E*	Lignans and Coumarins
Lazn006616	Buddlenol F	Lignans and Coumarins
Zmdn003480	Cycloolivil-6-O-glucoside	Lignans and Coumarins
Lmsp004450	Dehydrodiconiferyl alcohol	Lignans and Coumarins
Cmsp003083	Dehydrodiconiferyl alcohol-4-O-glucoside*	Lignans and Coumarins
Lmsp003655	Dehydrodiconiferyl alcohol-gamma’-O-glucoside*	Lignans and Coumarins
Lmtn002596	Dihydrodehydrodiconiferyl alcohol-4-O-glucoside*	Lignans and Coumarins
Lskp211385	dihydrosesamin	Lignans and Coumarins
Lskp211249	epieudesmin	Lignans and Coumarins
MWSHC20189	Epipinoresinol*	Lignans and Coumarins
Lazn003893	Erythro-Guaiacylglycerol-β-Coniferyl Ether	Lignans and Coumarins
Lazn006735	Erythro-Guaiacylglycerol-β-O-4′-dehydrodisinapyl Ether	Lignans and Coumarins
Lazn005262	Erythro-Guaiacylglycerol-β-Sinapyl Ether	Lignans and Coumarins
WaYn003112	Erythro-Guaiacylglycerol-β-threo-syringylglycerol Ether	Lignans and Coumarins
pmn001371	Eucommin A	Lignans and Coumarins
Lhhp102922	Fargesin	Lignans and Coumarins
Lazn002951	Guaiacylglycerol-β-Guaiacyl Ether	Lignans and Coumarins
Lazn002030	Guaiacylglycerol-β-Guaiacyl Ether glucoside	Lignans and Coumarins
Hmgn004139	Isohydroxymatairesinol	Lignans and Coumarins
Lmmn003748	Isolariciresinol	Lignans and Coumarins
Lmmn002274	Isolariciresinol-9’-O-glucoside*	Lignans and Coumarins
Cmsp005051	Isolariciresinol-9-O-glucoside	Lignans and Coumarins
Zmdn003762	Isosyringinoside	Lignans and Coumarins
HJN083	Lariciresinol-4’-O-glucoside	Lignans and Coumarins
Hmcn002743	Lirioresinol A	Lignans and Coumarins
Lmmn003020	Lyoniresinol	Lignans and Coumarins
Cmmn005231	Massoniresinol; Vladinol A	Lignans and Coumarins
Lssp210087	Matairesinol	Lignans and Coumarins
Lmmn003875	Medioresinol	Lignans and Coumarins
pmn001372	Medioresinol-4,4′-di-O-glucoside	Lignans and Coumarins
Rfmb056	Medioresinol-4’-O-(6″‘-acetyl)glucoside	Lignans and Coumarins
Hmln002100	Nortrachelogenin-4-O-glucoside	Lignans and Coumarins
pmn001376	Olivil	Lignans and Coumarins
Lhmp121010	Olivil Monoacetate	Lignans and Coumarins
Lmnn102709	Olivil-4’-O-glucoside	Lignans and Coumarins
mws0097	Pinoresinol*	Lignans and Coumarins
Rfmb25702	Pinoresinol-4-O-(6″-acetyl)glucoside	Lignans and Coumarins
pmn001378	Pinoresinol-4-O-glucoside	Lignans and Coumarins
Lskp211262	Secoisolariciresinol	Lignans and Coumarins
Lmtn003096	Secoisolariciresinol 4-O-glucoside	Lignans and Coumarins
MWSmce499	Secoisolariciresinol diglucoside	Lignans and Coumarins
Cmsn002480	Secoisolariciresinol-9’-O-glucoside	Lignans and Coumarins
MWS20152	Syringaresinol	Lignans and Coumarins
Rfmb26201	Syringaresinol-4’-O-(6″-acetyl)glucoside	Lignans and Coumarins
MWSHC2047	Syringaresinol-4’-O-glucoside; Acanthoside B	Lignans and Coumarins
MWSmce159	6,7-Dihydroxy-4-methylcoumarin	Lignans and Coumarins
Zbsp004940	6-Hydroxy-7-methoxycoumarin*	Lignans and Coumarins
zjgp122320	6-hydroxycoumarin	Lignans and Coumarins
mws0987	6-MethylCoumarin	Lignans and Coumarins
MWSslk135	7,8-Dihydroxy-4-methylcoumarin	Lignans and Coumarins
Walp004391	7-C-Glucosylcoumarin*	Lignans and Coumarins
Hmqn002118	7-Hydroxycoumarin;Umbelliferone	Lignans and Coumarins
mws1075	7-Methoxycoumarin	Lignans and Coumarins
Lhkp101525	Apiosylskimmin (Adicardin)	Lignans and Coumarins
pmn001492	Ayapin	Lignans and Coumarins
MWSmce301	Coumarin-3-carboxylic Acid	Lignans and Coumarins
mws1074	Daphnetin	Lignans and Coumarins
Cmyn001328	Daphnin*	Lignans and Coumarins
MWSmce232	Dicumarol	Lignans and Coumarins
mws1013	Esculetin (6,7-Dihydroxycoumarin)	Lignans and Coumarins
Lmbn001162	Esculetin-7-O-glucoside*	Lignans and Coumarins
pmb3093	Esculetin-7-O-quinic acid	Lignans and Coumarins
mws1015	Esculin (6,7-Dihydroxycoumarin-6-O-glucoside)*	Lignans and Coumarins
mws1014	Fraxetin (7,8-Dihydroxy-6-methoxycoumarin)	Lignans and Coumarins
MWSmce025	Fraxetin-8-O-glucoside (Fraxin)	Lignans and Coumarins
Lmjp003090	Isofraxidin-7-O-glucoside	Lignans and Coumarins
Lcyp000676	Isoscopoletin-β-D-glucoside*	Lignans and Coumarins
MWSslk171	Nodakenin	Lignans and Coumarins
Lmwp102713	Peucedanol*	Lignans and Coumarins
MWSCX014	Scopoletin (7-Hydroxy-6-methoxycoumarin)*	Lignans and Coumarins
Hmbp002498	Scopoletin-7-O-xylosyl(1 → 6)glucoside*	Lignans and Coumarins
Lhqp101805	Skimmin (7-Hydroxycoumarin-7-O-glucoside)	Lignans and Coumarins
Lmjp002764	Umckalin (7-hydroxy-5,6-dimethoxycoumarin)	Lignans and Coumarins
Qmzp101901	Zanthoxyloside*	Lignans and Coumarins
Wcdp010162	1-Monolinolenoyl-Rac-Glycerol*	Lipids
MWSmce549	1-Monomyristin	Lipids
pmb0296	1-Oleoyl-Sn-Glycerol	Lipids
Zbfn008434	1-O-Linoleoyl-3-O-galactopyranosyl-L-glycerol	Lipids
Wagn011658	1-Palmitoyl-Sn-Glycerol 3-O-Diglucoside	Lipids
pmb1562	1-Stearidonoyl-Glycerol	Lipids
Lmhp011562	1-α-Linolenoyl-glycerol*	Lipids
Lmhp009773	1-α-Linolenoyl-glycerol-3-O-glucoside*	Lipids
Wagn012030	2-Palmitoyl-Sn-Glycerol 3-O-Diglucoside	Lipids
Lmhp011388	2-α-Linolenoyl-glycerol*	Lipids
Lmhp008513	2-α-Linolenoyl-glycerol-1,3-di-O-glucoside	Lipids
Lmhp009526	2-α-Linolenoyl-glycerol-1-O-glucoside*	Lipids
Lmfp009701	Glycerol 9(E),11(Z),13(*E*)-octadecatrienoyl ester*	Lipids
Walp013004	Glycidyl Linoleate	Lipids
WaYn012231	LPG(18:2(9Z,12Z)/0:0)	Lipids
Hmyn007168	LysoPG 16:0	Lipids
Hmyn007087	LysoPI 16:0	Lipids
Sazp010264	Monogalactosyldiacylglycerol	Lipids
Lmsp010763	Monolinolenin*	Lipids
Hmyp007792	PE(oxo-11:0/16:0)	Lipids
Lmqn008024	PS(18:2)	Lipids
mws0120	Choline Alfoscerate	Lipids
pmb2221	4-Hydroxysphinganine; Phytosphingosine	Lipids
pmp001264	Hexadecylsphingosine	Lipids
pmd0130	LysoPC 14:0	Lipids
pmb2319	LysoPC 15:0*	Lipids
Lmhp009129	LysoPC 15:0(2n isomer)*	Lipids
pmb2260	LysoPC 15:1	Lipids
pmb0855	LysoPC 16:0	Lipids
pmd0132	LysoPC 16:0(2n isomer)	Lipids
pmp001270	LysoPC 16:1*	Lipids
Lmhp008833	LysoPC 16:1(2n isomer)*	Lipids
pmb0863	LysoPC 16:2(2n isomer)	Lipids
pmb2406	LysoPC 17:0*	Lipids
Lmhp010515	LysoPC 17:0(2n isomer)*	Lipids
Lmhp009590	LysoPC 17:1	Lipids
Lmhp008718	LysoPC 17:2	Lipids
mws0126	LysoPC 18:0	Lipids
pmd0136	LysoPC 18:0(2n isomer)	Lipids
Lmhp010190	LysoPC 18:1(2n isomer)	Lipids
pmp001251	LysoPC 18:2(2n isomer)	Lipids
pmb0865	LysoPC 18:3(2n isomer)	Lipids
Hmqp006235	LysoPC 18:4	Lipids
pmb2228	LysoPC 19:0	Lipids
Lmhp010908	LysoPC 19:1	Lipids
Lmhp007598	LysoPC 19:2(2n isomer)	Lipids
Lmhp011549	LysoPC 20:1	Lipids
pmd0147	LysoPC 20:2*	Lipids
pmd0146	LysoPC 20:2(2n isomer)*	Lipids
Lmhp009890	LysoPC 20:3	Lipids
pmb0864	LysoPE 14:0*	Lipids
Lmhp008337	LysoPE 14:0(2n isomer)*	Lipids
Lmhp009187	LysoPE 15:0	Lipids
Lmhp008885	LysoPE 15:0(2n isomer)	Lipids
Lmhp008440	LysoPE 15:1	Lipids
pmb0876	LysoPE 16:0	Lipids
pmd0160	LysoPE 16:0(2n isomer)	Lipids
Lmhp009034	LysoPE 16:1*	Lipids
Lmhp008763	LysoPE 16:1(2n isomer)*	Lipids
Lmhp010162	LysoPE 17:0	Lipids
Lmhp009769	LysoPE 17:1*	Lipids
Lmhp009464	LysoPE 17:1(2n isomer)*	Lipids
pmb0883	LysoPE 18:0	Lipids
mws0289	LysoPE 18:1*	Lipids
pmb0856	LysoPE 18:1(2n isomer)*	Lipids
pmb0881	LysoPE 18:2	Lipids
pmb0874	LysoPE 18:2(2n isomer)	Lipids
Lmhp008801	LysoPE 18:3	Lipids
Lmhp008589	LysoPE 18:3(2n isomer)	Lipids
Lmhp008233	LysoPE 18:4	Lipids
Lmhp010040	LysoPE 20:3*	Lipids
Lmhp009802	LysoPE 20:3(2n isomer)*	Lipids
Lmbn006152	(9Z,11E)-Octadecadienoic acid*	Lipids
MWSslk133	1,14-Tetradecanedioic Acid	Lipids
pmn001686	10,16-Dihydroxypalmitic acid	Lipids
Zmpn003698	11,14,17-Eicosatrienoic acid*	Lipids
mws2623	11-Octadecanoic acid(Vaccenic acid)*	Lipids
Lmbn005487	12,13-DHOME; (9*Z*)-12,13-Dihydroxyoctadec-9-enoic acid	Lipids
mws5045	12-Hydroxydodecanoic acid	Lipids
Lmbn009444	12-Hydroxyoctadec-9-Enoic Acid; Ricinoleic acid	Lipids
Zmyn004548	12-Oxo-phytodienoic acid	Lipids
Lmbn005369	13(*S*)-HODE;13(*S*)-Hydroxyoctadeca-9Z,11E-dienoic acid*	Lipids
Zmzn003953	13(s)-hydroperoxy-(9z,11e,15z)-octadecatrienoic acid	Lipids
MWS80007	13-Hydroperoxy-9Z,11E-octadecadienoic acid*	Lipids
Lmbn005443	13-KODE; (9Z,11E)-13-Oxooctadeca-9,11-dienoic acid*	Lipids
MWS2430	13-methylmyristic acid	Lipids
pmb2804	13S-Hydroperoxy-9Z,11E-octadecadienoic acid	Lipids
Zmyn005026	16-Methylheptadecanoic acid	Lipids
Zmyn004676	17-Hydroxylinolenic acid	Lipids
pmf0297	1-Eicosanol	Lipids
pmf0293	1-Octadecanol	Lipids
Zmyn005384	2R-Hydroxyoctadecanoic Acid*	Lipids
Zmyn005252	3-Hydroxy-palmitic acid methyl ester	Lipids
Wcdp006929	4-Oxo-9,11,13,15-Octadecatetraenoic Acid	Lipids
pmb0885	4-Oxo-9Z,11Z,13E,15E-Octadecatetraenoic Acid	Lipids
MWS2673	5,6-DiHETrE[(±)5,6-dihydroxy-8Z,11Z,14Z-eicosatrienoic acid]	Lipids
Lmbn005287	7S,8S-DiHODE; (9Z,12Z)-(7*S*,8*S*)-Dihydroxyoctadeca-9,12-dienoic acid*	Lipids
pmb2636	8,15-Dihydroxy-5,9,11,13-eicosatetraenoic acid	Lipids
Lmbn005662	9(10)-EpOME;(9*R*,10*S*)-(12Z)-9,10-Epoxyoctadecenoic acid	Lipids
pmn001694	9,10,13-Trihydroxy-11-Octadecenoic Acid	Lipids
Lmbn004240	9,10-Dihydroxy-12,13-epoxyoctadecanoic acid	Lipids
Lmbn003970	9,12,13-TriHOME; 9(*S*),12(*S*),13(*S*)-Trihydroxy-10(E)-octadecenoic acid	Lipids
pmn001691	9,12,13-Trihydroxy-10,15-octadecadienoic acid	Lipids
pmb2791	9-Hydroperoxy-10E,12,15Z-octadecatrienoic acid	Lipids
pmb2786	9-Hydroxy-10,12,15-octadecatrienoic acid*	Lipids
Zmyn004449	9-Hydroxy-12-oxo-10(E),15(Z)-octadecadienoic acid	Lipids
pmn001689	9-Hydroxy-12-oxo-15(Z)-octadecenoic acid*	Lipids
Rfmb087	9-Hydroxy-13-oxo-10-octadecenoic Acid	Lipids
WaYn010298	9-Hydroxyoctadeca-6,10,12,15-Tetraenoic Acid	Lipids
Wcdp007645	9-Oxo-10,12-Octadecadienoic Acid	Lipids
pmb2787	9-Oxo-10E,12Z-octadecadienoic acid	Lipids
Zmjn004133	9S-Hydroperoxy-10E,12Z-octadecadienoic acid	Lipids
Rfmb091	9S-Hydroxy-10E,12Z-octadecadienoic acid*	Lipids
Wafn011571	alpha-Hydroxylinoleic acid*	Lipids
pmf0397	Arachidic acid	Lipids
Zbfn013528	Beta-Hydroxypalmitic Acid*	Lipids
MWS0552	Cis-10-Pentadecenoic Acid(C15:1)	Lipids
Lmbn005923	Crepenynic acid	Lipids
Lmbn006210	Dihomo-gamma-linolenic acid; (8Z,11Z,14Z)-Icosatrienoic acid*	Lipids
MWS4295	DL-2-hydroxystearic acid*	Lipids
Lmhn012602	Docosenoic acid	Lipids
mws0474	Dodecanedioic aicd	Lipids
pmb2640	Dodecanoic acid (Lauric acid)	Lipids
pmn001606	Eicosenoic acid	Lipids
mws0396	Elaidic Acid*	Lipids
MWSslk132	Hexadecanedioic acid	Lipids
Zadn009075	Hydroperoxylinoleic acid*	Lipids
Wafn015217	Hydroxyicosanoic Acid	Lipids
mws1491	Linoleic acid*	Lipids
Yalp008047	Methyl 12-phenyldodecanoate	Lipids
pmb1650	Octadeca-11E,13E,15Z-trienoic acid	Lipids
Wcdp009119	Octadeca-9,12,15-trienoic acid	Lipids
YC512118	Oleamide (9-Octadecenamide)	Lipids
pmf0395	Oleic acid	Lipids
pme2827	Palmitaldehyde	Lipids
mws1488	Palmitic acid	Lipids
Lmyn012331	Petroselinic acid*	Lipids
pmb0889	Punicic acid (9Z,11E,13Z-octadecatrienoic acid)	Lipids
mws1489	Stearic Acid	Lipids
Lmsn004901	Tetracosanoic Acid (Lignoceric acid)	Lipids
MWS5231	Tridecanedioic acid	Lipids
MWS1900	Undecanedioic acid	Lipids
mws0367	α-Linolenic Acid*	Lipids
mws0366	γ-Linolenic Acid*	Lipids
mws0663	1,7-Dimethylxanthine	Nucleotides and derivatives
MWSmce295	1-beta-D-Arabinofuranosyluracil	Nucleotides and derivatives
mws0847	1-Methyladenine	Nucleotides and derivatives
pme0166	1-Methylxanthine	Nucleotides and derivatives
pme3967	2-(Dimethylamino)guanosine*	Nucleotides and derivatives
pmb0374	2-Aminopurine	Nucleotides and derivatives
pme3961	2’-Deoxyadenosine*	Nucleotides and derivatives
pme3184	2’-Deoxyadenosine-5′-monophosphate	Nucleotides and derivatives
pme1194	2’-Deoxycytidine	Nucleotides and derivatives
pme1184	2’-Deoxyguanosine	Nucleotides and derivatives
pmc0066	2’-Deoxyinosine-5′-monophosphate	Nucleotides and derivatives
pmb2507	2-Deoxyribose-1-phosphate	Nucleotides and derivatives
mws0863	2-Deoxyribose-5′-phosphate	Nucleotides and derivatives
Hmhp001812	2’-O-Methyladenosine	Nucleotides and derivatives
mws0874	3′-Adenylic Acid	Nucleotides and derivatives
pme1266	3-Methylxanthine	Nucleotides and derivatives
pme0152	5,6-Dihydro-5-methyluracil	Nucleotides and derivatives
pme0151	5,6-Dihydrouracil	Nucleotides and derivatives
Lmqp000329	5-Aminoimidazole ribonucleotide	Nucleotides and derivatives
pme1474	5’-Deoxy-5′-(methylthio)adenosine	Nucleotides and derivatives
MWSmce116	5’-Deoxyadenosine*	Nucleotides and derivatives
MWS2910	5-Methyl-2’-Deoxycytidine	Nucleotides and derivatives
mws0572	5-Methylcytosine	Nucleotides and derivatives
MWSslk102	6-Chloropurine	Nucleotides and derivatives
MWS4525	6-*O*-methylguanine	Nucleotides and derivatives
pme3968	7-Methylguanine	Nucleotides and derivatives
pme2801	7-Methylxanthine	Nucleotides and derivatives
MWS2984	8-Azaguanine	Nucleotides and derivatives
mws0724	8-Hydroxyguanosine	Nucleotides and derivatives
mws1060	9-(Arabinosyl)hypoxanthine	Nucleotides and derivatives
Wcjp001256	9-Alpha-Ribofuranosyladenine*	Nucleotides and derivatives
Zmsp001773	9-Arabinosyladenine*	Nucleotides and derivatives
MWSHC2054	Adenine	Nucleotides and derivatives
pme0230	Adenosine*	Nucleotides and derivatives
Zmjn001030	Adenosine 2’-Phosphate	Nucleotides and derivatives
pme2117	Adenosine 5′-diphosphate	Nucleotides and derivatives
pmb0981	Adenosine 5′-monophosphate	Nucleotides and derivatives
pme1173	Allopurinol	Nucleotides and derivatives
mws1715	Cordycepin (3’-Deoxyadenosine)*	Nucleotides and derivatives
MWSmce333	Crotonoside; 2-Hydroxyadenosine	Nucleotides and derivatives
mws0884	Cyclic 3′,5′-Adenylic acid	Nucleotides and derivatives
ML10180524	Cytarabine	Nucleotides and derivatives
pme3732	Cytidine	Nucleotides and derivatives
MWSslk257	Cytidine 5′-monophosphate(Cytidylic acid)	Nucleotides and derivatives
mws0255	Cytosine	Nucleotides and derivatives
MWS5083	Flavin Single Nucleotide(FMN)	Nucleotides and derivatives
pme1109	Guanine	Nucleotides and derivatives
pme1178	Guanosine	Nucleotides and derivatives
mws0609	Guanosine 3′,5′-cyclic monophosphate	Nucleotides and derivatives
pmb0998	Guanosine 5′-monophosphate	Nucleotides and derivatives
pme0033	Hypoxanthine	Nucleotides and derivatives
pme1119	Inosine	Nucleotides and derivatives
pmb0532	Inosine 5′-monophosphate	Nucleotides and derivatives
MWS5173	Isocytosine	Nucleotides and derivatives
pme0183	Isoguanine	Nucleotides and derivatives
pmb0964	Isopentenyladenine-7-N-glucoside	Nucleotides and derivatives
mws0984	L-Sepiapterin	Nucleotides and derivatives
Lmcp002302	N6-(2-Hydroxyethyl)adenosine*	Nucleotides and derivatives
pme2060	N6-Isopentenyladenine	Nucleotides and derivatives
MWS4354	N6-methyladenosine	Nucleotides and derivatives
pmb0197	N7-Methylguanosine	Nucleotides and derivatives
pme2651	NADP (Nicotinamide adenine dinucleotide phosphate)	Nucleotides and derivatives
pmb0530	Nicotinic acid adenine dinucleotide	Nucleotides and derivatives
pme2746	Riboflavin 5′-Adenosine Diphosphate	Nucleotides and derivatives
pmf0289	Riboprine	Nucleotides and derivatives
pmc0281	Ribosyladenosine	Nucleotides and derivatives
pme3337	Succinyladenosine	Nucleotides and derivatives
mws0251	Thymine	Nucleotides and derivatives
pme0257	Uracil	Nucleotides and derivatives
mws0248	Uridine	Nucleotides and derivatives
pme3007	Uridine 5′-diphosphate	Nucleotides and derivatives
pmb2922	Uridine 5′-diphospho-d-glucose	Nucleotides and derivatives
pme3188	Uridine 5′-monophosphate	Nucleotides and derivatives
Zmfn000481	Uridine-5’-Diphosphate-D-Xylose	Nucleotides and derivatives
Zmjp000966	Vidarabine	Nucleotides and derivatives
pme0256	Xanthine	Nucleotides and derivatives
mws0668	Xanthosine	Nucleotides and derivatives
mws0675	β-Nicotinamide mononucleotide	Nucleotides and derivatives
pme3154	Mevalonic acid	Organic acids
mws0277	Quinic Acid	Organic acids
mws4052	1-Aminocyclopropane-1-carboxylic acid*	Organic acids
MWS4396	1-Hydroxy-2-Naphthoic Acid	Organic acids
Rfmb320	1-Methylpiperidine-2-carboxylic acid*	Organic acids
Lmgn004461	2-(1,3-Dihydroxy-but-2-enylidene)-6-methyl-3-oxo-heptanoic acid	Organic acids
Lcsn006884	2,2′-(3-methylcyclohexane-1,1-diyl)diacetic acid	Organic acids
Wmzn000227	2,2-Dimethylsuccinic acid	Organic acids
Zmgn000503	2,3-Dihydroxy-3-Methylbutanoic Acid	Organic acids
pme0278	2,6-Diaminooimelic acid	Organic acids
Wccp000476	2-[(1*R*)-1-carboxyethoxy]propanoic acid	Organic acids
Lmbn001609	2-Acetyl-2-Hydroxybutanoic Acid	Organic acids
Zbqp000579	2-amino-3-(1H-pyrazol-1-yl)propanoic acid	Organic acids
mws0236	2-Aminoethanesulfonic acid	Organic acids
pme3017	2-Aminoisobutyric acid*	Organic acids
Lmbn001288	2-Hydroxy-2-methyl-3-oxobutanoic acid	Organic acids
Lmrn002746	2-Hydroxy-4-methylpentanoic acid	Organic acids
Zmgn000216	2-Hydroxyethylphosphonic acid	Organic acids
Zmyn000247	2-Hydroxyglutaric Acid*	Organic acids
Lmmn003323	2-Hydroxyhexadecanoic acid*	Organic acids
mws0341	2-Hydroxyisocaproic acid	Organic acids
Lcsn006335	2-Hydroxymyristic acid	Organic acids
Zmyn002323	2-Hydroxyphenylacetic acid	Organic acids
pmb3101	2-Isopropylmalic Acid	Organic acids
Lmgn002091	2-Methyl-3-oxoadipic acid	Organic acids
Lmgn000224	2-Methyl-3-oxosuccinic acid	Organic acids
mws0924	2-Methylglutaric acid*	Organic acids
mws0473	2-Methylsuccinic acid*	Organic acids
pme2589	2-Oxoadipic acid	Organic acids
mws2124	2-Phosphoglycolate	Organic acids
pme1216	2-Picolinic acid	Organic acids
Zmgn001448	2-Propylmalic Acid*	Organic acids
Lmbn002072	2-Propylsuccinic acid*	Organic acids
MWS5147	3-(Methylthio)Propionic Acid	Organic acids
ML10176345	3-Dehydroshikimic acid	Organic acids
MWSmce362	3-Ethoxy-3-oxopropanoic acid	Organic acids
Hmhn002738	3-Furoic acid	Organic acids
Zmpn000638	3-Guanidinopropionic acid	Organic acids
Lmbn001676	3-Hydroxy-3-Methyl-2-Oxopentanoic Acid*	Organic acids
mws0576	3-Hydroxybutyric acid	Organic acids
MWS3036	3-Hydroxyglutaric acid*	Organic acids
MWS2417	3-Hydroxymandelate	Organic acids
pme2601	3-Hydroxypropanoic acid	Organic acids
Lmbn001754	3-Isopropylmalic Acid*	Organic acids
Lmbn001718	3-Methyl-2-oxopentanoic acid	Organic acids
Lmbn000216	3-Methylmalic acid*	Organic acids
Lmgn000242	4,5,6-Trihydroxy-2-oxohexanoic acid	Organic acids
Zmtn001464	4,8-Dihydroxyquinoline-2-carboxylic acid	Organic acids
pme0295	4-Acetamidobutyric acid	Organic acids
mws0567	4-Guanidinobutyric acid	Organic acids
mws0373	4-Methyl-2-oxovalerate	Organic acids
MWS5238	4-Phenylbutyric acid	Organic acids
Lmbn001467	5-Acetamidopentanoic Acid	Organic acids
MWSslk038	6-Acetamidohexanoic acid	Organic acids
pme0274	6-Aminocaproic acid	Organic acids
mws0972	6-Hydroxyhexanoic acid	Organic acids
Lmtn004049	Abscisic acid	Organic acids
mws0208	Adipic Acid*	Organic acids
pme3096	Aminomalonic acid	Organic acids
Lmyp003934	Anacardic acid	Organic acids
mws0237	Azelaic acid	Organic acids
mws0489	Benzoylformic acid	Organic acids
mws0425	Citraconic acid	Organic acids
mws0281	Citric Acid	Organic acids
WaYn000716	Citric Acid diglucoside	Organic acids
ML10197929	Creatine	Organic acids
pme1936	Creatinine	Organic acids
pme1730	D-Erythronolactone	Organic acids
Lmmn000806	Dimethylmalonic acid*	Organic acids
MWS0274	DL-3-Phenyllactic acid*	Organic acids
mws0267	DL-Glyceric Acid	Organic acids
MWS1709	D-Malic acid*	Organic acids
MWSmce183	D-Mandelic acid	Organic acids
pme3034	Ethylmalonic acid*	Organic acids
mws0376	Fumaric acid*	Organic acids
Wasn001627	Glucosyl 2,3-Dihydroxy-2-Methylbutanoic Acid	Organic acids
Wasn003258	Glucosyl 2-Hydroxy-4-Methylpentanoic Acid	Organic acids
pme0243	Glutaric acid*	Organic acids
Lmhn007953	Hexylitaconic acid	Organic acids
Lcsn002137	Homovanillic acid sulfate	Organic acids
ML10172161	Hydroxypyruvic acid*	Organic acids
MWS1882	Iminodiacetic acid*	Organic acids
Zmyn000453	Isocitric Acid	Organic acids
Zmgn000217	Itaconic acid	Organic acids
Zmjp003163	Jasmonic acid	Organic acids
Lcsn000415	Keto-Deoxy-Nonulonic acid	Organic acids
mws0275	L-Malic acid*	Organic acids
MWS0811	L-Pipecolic Acid	Organic acids
mws0262	L-Tartaric acid	Organic acids
pme0271	Maleic acid*	Organic acids
Wayn000504	Malic acid-1-O-diglucoside	Organic acids
pme1975	Malonic acid	Organic acids
Lmyn002403	Mandelic acid-β-glucoside	Organic acids
MWS2040	Methanesulfonic acid	Organic acids
MWSmce536	Methyl 2-furoate	Organic acids
pme0220	Methyl jasmonate	Organic acids
mws0470	Methylmalonic acid*	Organic acids
MWS5136	Mono-Methyl Glutarate*	Organic acids
Lmmn002164	Monomethyl succinate*	Organic acids
Lmyn000160	Mucic acid Dimethyl Ester	Organic acids
mws1167	Oxalacetic acid	Organic acids
pmf0096	Oxalic acid	Organic acids
mws0159	Phenylpyruvic acid	Organic acids
mws2125	Phosphoenolpyruvate	Organic acids
Lmsn015919	Phytic acid	Organic acids
Zmjn001813	Pimelic acid*	Organic acids
mws0601	Pyrrole-2-carboxylic acid	Organic acids
mws0154	Shikimic acid	Organic acids
mws0242	Suberic Acid	Organic acids
mws0192	Succinic acid*	Organic acids
Lmgn000219	Succinic semialdehyde	Organic acids
Lmbn000193	Tartronate semialdehyde*	Organic acids
pme3009	trans-Aconitic acid	Organic acids
pme2380	α-Ketoglutaric acid	Organic acids
mws0147	β-Hydroxyisovaleric acid	Organic acids
pme3011	γ-Aminobutyric acid	Organic acids
Zmdp000917	δ-Guanidinovaleric acid	Organic acids
Lasp003143	1,4-Benzodioxin-6-propanol	Others
pmf0174	1-Decanol*	Others
Lmqp006559	2-Amino-1,3-eicosanediol	Others
pmf0175	2-Decanol*	Others
Jmzn006005	3,4-methylenedioxy cinnamyl alcohol	Others
pmf0256	3-Methyl-1-pentanol	Others
pmn001380	Eucommiol	Others
MWS0618	Pantothenol	Others
Hmjp000461	3-O-Glucoside-3-hydroxy-y-butyrolactone	Others
MWS2056	Delta-Hexalactone	Others
Cmyp007180	Dihydroactinidiolide	Others
pmb0128	δ-Tridecalactone	Others
Waln010743	1-(2,3-dihydroxypropoxy)-3-(((2-(dimethylamino)ethoxy)(hydroxy)phosphoryl)oxy)propan-2-yl (11Z,14Z)-octadeca-11,14-dienoate	Others
Waln009920	1-(2,3-dihydroxypropoxy)-3-(((2-(dimethylamino)ethoxy)(hydroxy)phosphoryl)oxy)propan-2-yl (8E,11Z,14Z)-octadeca-8,11,14-trienoate	Others
Waln010449	1-(2,3-dihydroxypropoxy)-3-(((2-(dimethylamino)ethoxy)(hydroxy)phosphoryl)oxy)propan-2-yl (*E*)-hexadec-9-enoate	Others
Waln011704	1-(2,3-dihydroxypropoxy)-3-(((2-(dimethylamino)ethoxy)(hydroxy)phosphoryl)oxy)propan-2-yl (Z)-14-Octadecenoic Acid	Others
WaYn011606	1-(2,3-dihydroxypropoxy)-3-(((2-(dimethylamino)ethoxy)(hydroxy)phosphoryl)oxy)propan-2-yl heptadecanoate	Others
Waln011524	1-(2,3-dihydroxypropoxy)-3-(((2-(dimethylamino)ethoxy)(hydroxy)phosphoryl)oxy)propan-2-yl palmitate*	Others
Wcdp006741	1-(9Z,12Z-Octadecadienoyl)-Sn-Glycero-3-Phosphocholine	Others
Walp007738	14-Amino-15-hydroxy-11-methylpentadecanoic acid	Others
Wcsn010224	14-hydroxy-2,6,10-trimethylpentadeca-2,5,10-trien-4-one*	Others
WaYn011395	2-(2,3-dihydroxypropoxy)-3-(((2-(dimethylamino)ethoxy)(hydroxy)phosphoryl)oxy)propan-2-yl (Z)-14-Octadecenoic Acid	Others
Waln011009	2-(2,3-dihydroxypropoxy)-3-(((2-(dimethylamino)ethoxy)(hydroxy)phosphoryl)oxy)propyl (11Z,14Z)-octadeca-11,14-dienoate	Others
Waln010192	2-(2,3-dihydroxypropoxy)-3-(((2-(dimethylamino)ethoxy)(hydroxy)phosphoryl)oxy)propyl (8E,11Z,14Z)-octadeca-8,11,14-trienoate	Others
Waln011222	2-(2,3-dihydroxypropoxy)-3-(((2-(dimethylamino)ethoxy)(hydroxy)phosphoryl)oxy)propyl palmitate*	Others
Lhyp111013	2(4H)-benzofuranone	Others
Zahp011689	2,2-dimethylchromene-6-carboxylic acid	Others
Hasp010605	2,3-dihydroxypropyl (9Z,12Z,15Z)-octadeca-9,12,15-trienoate	Others
Walp008255	2-aminodocoSane-1,5,7,21-tetraol	Others
Walp007192	2-aminodocoSane-1,6,19,20,21-pentaol	Others
Walp004995	2-Aminododecane-1,3,4-triol	Others
Walp004752	2-Aminododecane-1,4-diol	Others
pmb0302	2-Aminoethylphosphonate	Others
WaYp007721	2-Aminohexadecane-1,16,16-triol	Others
Walp007043	2-Aminohexadecane-1,4-diol*	Others
WaYp006462	2-Aminohexadecane-1,5,15-triol	Others
Walp007214	2-Aminohexadecane-1,5,6-triol	Others
Walp008158	2-AminoicoSane-1,5,19-triol	Others
Walp007190	2-AminoicoSane-1,5,7,19-tetraol	Others
Walp007137	2-AminoicoSane-1,6,18,19,20-pentaol	Others
WaYp007695	2-Aminooctadecane-1,16,18,18-tetraol	Others
Walp008102	2-Aminooctadecane-1,4-diol	Others
Walp007188	2-Aminooctadecane-1,5,7,17-tetraol	Others
WaYp007548	2-Aminotetradecan-1-ol	Others
Walp004906	2-Aminotetradecane-1,11,13-triol	Others
Walp006011	2-Aminotetradecane-1,4-diol*	Others
Walp006175	2-Aminotetradecane-1,5,11-triol	Others
WaYp005128	2-Aminotetradecane-1,5,13-triol	Others
Walp005087	2-Aminotetradecane-1,5,7,11-tetraol	Others
Wagp006039	2-Hexylphosphoric Acid	Others
Wagp002530	2-Methoxy-1-benzofuran-5-carbaldehyde	Others
Wcgp003250	2-Methyl-3-hydroxyindan-1-one	Others
Wagp002074	3-(1-hydroxyethyl)-4-methylpentane-1,4-diol O-Glucoside	Others
Wcgp006109	3-(2-hydroxyethyl)-5,7-dimethoxy-4-methyl-2H-1-benzopyran-2-one*	Others
Wdbn005328	3,3’-Bis(3,4-dihydro-4-hydroxy-6,8-dimethoxy-2H-1-benzopyran)	Others
Lskp211267	3-Ethyl-7-hydroxyphthalide	Others
Wchn004015	3-Hydroxy-1-(4′-hydroxy-3′-methoxyphenyl)-propan-1-one	Others
Wagp005869	3-Hydroxy-beta-ionol 3-(6″-Malonyl)Glucoside	Others
Wagp005297	3-Hydroxy-beta-ionol 3-Glucoside*	Others
Wcdp001930	3ξ-(1ξ-hydroxyethyl)-7-hydroxy-1-isobenzofuranone	Others
Saln003418	4-(Beta-D-Glucopyranosyloxy)-2,4′,6-Trihydroxybenzophenone	Others
Wagp001892	4-(Beta-D-Glucopyranosyloxy)-2-Pentanol	Others
Wcsn010254	4-[3-(4,8-dimethylnona-3,7-dienyl)-3-methyloxiran-2-yl]butan-2-one*	Others
Zmmp002106	4-methyl-1,5,2,3-dioxadiazinan-2-amine	Others
pmb0764	4-Methyl-5-thiazoleethanol	Others
zjgp122321	4-*O*-acetyl-3-O-caffeoyl-2-*C*-methyl-D-erythronate*	Others
Wcgp003276	5-(2-hydroxypropyl)-3H-2-benzofuran-1-one	Others
Zbzp007397	5,6,7,7a-tetrahydro-4,4,7a-trimethyl-2(4H)-benzofuranone	Others
Wcgp009361	5,7-diethoxy-3-(2-hydroxyethyl)-4-methyl-2H-1-benzopyran-2-one	Others
Lmln001856	5,7-Dihydroxy-4-oxo-2-(3,4,5-trihydroxyphenyl)-4H-chromen-3-yl-alloside	Others
Zahn007990	5-hydroxy-3,4-dimethyl-5-pentylfuran-2(5H)-one	Others
Lhmp122205	5-O-Methyllatifolin	Others
Wcsn010496	6,10,14-Trimethylpentadeca-5,9-Diene-2,13-Dione	Others
Hahn002084	6’-O-caffeoylcatalpol*	Others
Wcsn011125	Ethyl 15,16-dihydroxy-5,9-dimethyloctadeca-4,6,8,10,13-pentaenoate	Others
Lhhp120823	Eugenyl formate	Others
Wasn003817	Glucosyl 5,8-dihydroxy-2,6-dimethylocta-2,6-dienoic acid	Others
Wasn005621	Glucosyl 9-hydroxy-3-methyldec-2-enoic acid	Others
Wcdp004275	Lauramine oxide	Others
Wcdp006912	Lauryldiethanolamine*	Others
Lcsp012679	linolenoylethanolamine	Others
Lmhp001671	Noreugenin-7-O-glucoside*	Others
Wcdp010708	Octadeca-2,9,12,15-tetraen-1-ol	Others
Waln006120	Octyl 6-O-Alpha-L-Arabinopyranosyl-Beta-D-Glucopyranoside	Others
MWS20159	Phenyl-β-D-glucopyranoside	Others
zjgp122326	Pyridine-4-formyl-O-β-D-glucopyranoside*	Others
Zmsn110105	Rumexobtusifolius	Others
Wcdp005264	Tetradecasphinganine*	Others
MWSmce581	2,4-Dihydroxybenzaldehyde	Others
Lmbn001981	2,5-Dihydroxybenzaldehyde*	Others
Lmbn005172	2,6-Dimethoxybenzaldehyde*	Others
MWS1844	2-Pentyl-3-phenyl-2-propenal	Others
Wmlp000056	3,5-Dimethoxy-4-hydroxybenzaldehyde	Others
Lmbn002737	3-Methylbenzaldehyde	Others
MWSmce644	4-Acetoxy-3-Ethoxybenzaldehyde	Others
Zmdp000376	4-Guanidinobutanal	Others
mws0628	4-Hydroxybenzaldehyde	Others
MWSCX017	4-hydroxyphenyl acrylaldehyde*	Others
MWS1852	4-Methoxybenzaldehyde	Others
MWS20172	5-Hydroxymethylfurfural	Others
MWSmce040	Isovanillin	Others
Hmgn001653	Protocatechualdehyde*	Others
mws1350	Syringaldehyde; 4-Hydroxy-3,5-Dimethoxybenzaldehyde	Others
mws0458	Vanillin; 4-Hydroxy-3-Methoxybenzaldehyde*	Others
Hmyp002315	3,5,7,4’-Tetrahydroxy-Coumaronochromone	Others
Hmsp003093	3,7-Dihydroxychromen-4-one	Others
Layp002880	5,7-Dihydroxychromone glucoside	Others
MWSHY0141	7-Hydroxy-4-chromone	Others
Zmzp006646	Capillarisin	Others
MWSmce658	Noreugenin; 5,7-Dihydroxy-2-Methylchromone	Others
pmp000307	Sec-O-glucosylhamaudol	Others
Lmqn000432	1-(sn-Glycero-3-phospho)-1D-myo-inositol	Others
pme2529	1,5-Anhydro-D-glucitol	Others
MWS0559	1,6-anhydro-β-d-glucose	Others
Wafn004792	1-O-Acetyl-Glucopyranose 6-Decanoate	Others
Zmyn000268	2,3-Dihydroxypropanal	Others
Zmyn000230	2-Dehydro-3-deoxy-L-arabinonate*	Others
Lmbn000198	3-Dehydro-L-Threonic Acid*	Others
Lcsn000341	3’-Fucosyllactose	Others
Zmgn000447	3-Phospho-D-glyceric acid	Others
Wasn000977	4-O-galactopyranosylxylose	Others
Wasn000495	6-O-alpha-L-arabinopyranosyl-d-glucopyranose	Others
Jmbn003202	Butyl Beta-D-Fructopyranoside	Others
Hmqp001042	Butyl beta-D-glucoside	Others
Lmsn000954	Dambonitol	Others
ML10171848	D-Arabinono-1,4-lactone*	Others
MWSmce676	D-Arabinose*	Others
mws0437	D-Arabitol*	Others
MWSmce203	D-Cellobiose	Others
Zmzn000079	D-Erythrose-4-phosphate	Others
mws1164	d-Fructose*	Others
MWS2442	d-Fructose 6-Phosphate*	Others
mws1595	D-Fucose*	Others
Zmpn000199	D-Galactaric acid	Others
pmf0139	D-Galactose*	Others
mws1189	D-Galacturonic acid*	Others
MWSmce220	D-Glucono-1,5-lactone*	Others
pme3705	D-Glucoronic acid*	Others
pmb0786	D-Glucosamine	Others
Zmyn000110	D-Glucosamine 1-phosphate	Others
mws4170	d-Glucose*	Others
Zmyn000083	d-Glucose 1,6-bisphosphate	Others
mws0866	d-Glucose 6-phosphate*	Others
mws1090	d-Glucose-1-phosphate*	Others
mws4175	D-Glucurono-6,3-lactone	Others
Lmxn000380	Digalactosylglycerol	Others
Zmzn000078	Dihydroxyacetone phosphate	Others
MA10039641	d-Lactose*	Others
MWSmce682	DL-Xylose*	Others
Lmsn000381	D-Maltose*	Others
mws1593	D-Maltotetraose	Others
mws1155	d-Mannitol*	Others
pmf0138	D-Mannose*	Others
pme0500	D-Melezitose	Others
mws1589	D-Panose*	Others
mws2104	D-Pinitol	Others
Zmgn000173	d-Ribose	Others
Zmyn000108	D-Saccharic acid	Others
pme3163	D-Sedoheptuiose 7-phosphate	Others
mws0214	D-Sorbitol	Others
pme0519	D-Sucrose*	Others
pme2134	d-Threitol*	Others
mws0889	D-Threonic Acid	Others
pma0134	D-Threose	Others
mws0264	D-Trehalose*	Others
pme2237	Dulcitol*	Others
mws0344	D-Xylonic acid	Others
mws1080	Galactinol	Others
pmb3081	Glucaric acid-1-Phosphate	Others
pme0534	Gluconic acid	Others
MWSmce113	Guaifenesin	Others
Hmln000297	Inositol*	Others
mws5038	Isomaltulose*	Others
MWSmce165	L-Fucitol	Others
pme2435	L-Fucose*	Others
MWSmce576	l-Glucose*	Others
pme2253	L-Gulono-1,4-Lactone*	Others
MWSmce199	L-Xylose*	Others
MWS1983	Maltitol	Others
MWS0442	Maltotriose	Others
MWSmce486	Manninotriose	Others
mws1333	Melibiose	Others
pmf0283	Meso-Erythritol*	Others
mws2608	N-Acetyl-D-galactosamine	Others
pme2755	N-Acetyl-D-glucosamine	Others
pmb3079	N-Acetyl-D-glucosamine-1-phosphate	Others
mws4174	N-Acetyl-D-mannosamine	Others
mws4163	Nystose	Others
Hmqp000191	Planteose	Others
pme2125	Raffinose*	Others
mws0854	Rhamnose*	Others
mws0213	Ribitol*	Others
pma6455	Ribulose-5-phosphate	Others
Lmqn000351	Rutinose	Others
Hmcn000192	Sedoheptulose	Others
Lmmn000214	Solatriose	Others
Zmpn000095	Sorbitol-6-phosphate	Others
Lmqn000213	Stachyose	Others
mws1089	Sucrose-6-phosphate	Others
mws2523	Trehalose 6-phosphate	Others
Lmdn000248	Verbascose	Others
pme0513	Xylitol*	Others
MWSslk225	1-Indanone	Others
Zmgp004297	1-phenyl-7-(4-hydroxyphenyl)-4-ene-3-heptanone	Others
Lasp003640	2,4-Dihydroxy-6-methoxyacetophenone	Others
Lafp003256	3,4’-Dihydroxy-3′,5′-dimethoxypropiophenone	Others
pmp001119	3,5-Dihydroxy-2,4-dimethoxy-9H-fluoren-9-one	Others
Lmyp003951	3-Hydroxy-1-(4-hydroxy-3,5-dimethoxyphenyl)propan-1-one	Others
MWSslk095	4-Hydroxy-2,5-dimethyl-3(2H)furanone	Others
MWS20181	4’-Hydroxy-3′-methoxyacetophenone (Acetovanillone)	Others
MWSmce466	4-Hydroxyacetophenone	Others
MWSmce283	4’-Hydroxypropiophenone	Others
Wbtn006721	6,8-dihydroxy-3-methyl-9-oxoxanthene-1-carboxylic acid*	Others
MWSslk226	Benzylacetone	Others
Wbmp003302	Frambinone	Others
MWSHC20159	Roseoside	Others
Lmtp004146	Santalin	Others
MWS0434	Trans-dehydrorosinone	Others
Wasn000528	2-O-α-d-Glucopyranosyl-l-ascorbic acid	Others
pme2596	4-Pyridoxic acid	Others
pmb0801	4-Pyridoxic acid-O-glucoside	Others
pme2266	Biotin	Others
MA10039492	Dehydroascorbic acid	Others
mws1337	D-Pantothenic Acid	Others
MWSmce690	Erythorbic Acid; Isoascorbic Acid	Others
Wasn001007	Isoascorbic acid 2-O-glucoside	Others
MWSmce039	Isonicotinic acid	Others
pma1751	N-(beta-D-Glucosyl)nicotinate*	Others
mws0133	Nicotinamide	Others
pma3101	Nicotinate D-ribonucleoside	Others
pme0490	Nicotinic acid (Vitamin B3)	Others
pme2165	Orotic acid (Vitamin B13)	Others
pme3511	Orotidine	Others
MWSmce674	Phylloquinone (Vitamin K1)	Others
Zmjp000624	Pyridoxal	Others
mws0655	Pyridoxal-5′-phosphate	Others
pme1383	Pyridoxine	Others
pmb0790	Pyridoxine-5’-O-diglucoside	Others
pmb0789	Pyridoxine-5’-O-glucoside	Others
pme1306	Pyridoxine-5′-phosphate	Others
pme2289	Retinol (Vitamin A1)	Others
mws0232	Riboflavin (Vitamin B2)	Others
MWSmce489	2,3,5,4’-Tetrahydroxystilbene-2-O-glucoside	Others
Lmtp004915	3,5-Dihydroxy-3′,4′-diacetoxylstilbene-3-O-glucoside	Others
MWSmce484	Astringin	Others
Zmhn001970	Piceid (3,4′,5-Trihydroxystilbene-3-O-glucoside)	Others
mws0021	Resveratrol	Others
mws0178	Chlorogenic acid (3-O-Caffeoylquinic acid)*	Phenolic acids
mws2108	Cryptochlorogenic acid (4-O-Caffeoylquinic acid)*	Phenolic acids
pmn001382	Isochlorogenic acid A*	Phenolic acids
Li512115	Isochlorogenic acid B*	Phenolic acids
pmn001384	Isochlorogenic acid C*	Phenolic acids
MWSprf004	Neochlorogenic acid (5-O-Caffeoylquinic acid)*	Phenolic acids
mws1358	Pyrocatechol	Phenolic acids
Zbfn002690	1-(4-Hydroxybenzoyl)Glucose; 25,545–07-7	Phenolic acids
pmn001681	1-(4-Methoxyphenyl)-1-propanol	Phenolic acids
Lmfn001337	1,2,3-Tri-O-galloyl-β-d-glucose*	Phenolic acids
Zbyn004414	1,2,4,6-tetra-O-galloyl-β-d-glucose	Phenolic acids
Zmdn003203	1,2,6-Tri-O-galloyl-β-d-glucose*	Phenolic acids
Lmsn004534	1,2-Di-O-galloyl-6-O-Cinnamoyl-β-d-glucose	Phenolic acids
Lmfn001209	1,3,6-Tri-O-galloyl-β-d-glucose*	Phenolic acids
Cmbp005523	1,3-O-Dicaffeoylglycerol	Phenolic acids
mws1584	1,3-O-Dicaffeoylquinic Acid (Cynarin)	Phenolic acids
Lmsn003088	1,4,6-Tri-O-galloyl-β-d-glucose*	Phenolic acids
Wmhn001495	1,4-Di-O-Galloyl-d-glucose	Phenolic acids
Waxn004217	1,5-O-dicaffeoyl-3-O-glucoside-quinic acid	Phenolic acids
Lmsn004322	1,6-Di-O-caffeoyl-β-d-glucose*	Phenolic acids
Lmsn004659	1,6-Di-O-galloyl-2-O-Feruloyl-β-d-glucose	Phenolic acids
mws0748	1-Caffeoylquinic acid	Phenolic acids
pmp000086	1-Feruloyl-sn-glycerol	Phenolic acids
MWS2066	1-Naphthol*	Phenolic acids
Wasn002933	1-O-(3,4,5-Trimethoxybenzoyl)-B-D-Glucopyranoside	Phenolic acids
Lmtn000940	1-O-(3,4-Dihydroxy-5-methoxy-benzoyl)-glucoside	Phenolic acids
Lmnn002886	1’-O-(3,4-Dihydroxyphenethyl)-O-caffeoyl-glucoside	Phenolic acids
Lmsn002288	1-O-Caffeoyl-(6-O-glucosyl)-β-d-glucose	Phenolic acids
Lmsn004690	1-O-Caffeoyl-3-O-galloyl-4,6-(*S*)-HHDP-β-d-glucose	Phenolic acids
Lmsn002937	1-O-Caffeoyl-6-O-galloyl-β-d-glucose	Phenolic acids
Hmbp003234	1-O-Caffeoylglycerol	Phenolic acids
pmn001420	1-O-Caffeoyl-β-d-glucose*	Phenolic acids
Lmsn002887	1-O-Caffeoyl-β-D-xylose	Phenolic acids
Lmsp004521	1-O-Cinnamoyl-4,6-(*S*)-HHDP-β-d-glucose	Phenolic acids
pma3724	1-O-Feruloylquinic acid	Phenolic acids
Zmhn002422	1-O-Feruloyl-β-d-glucose	Phenolic acids
Lmsn004323	1-O-Galloyl-2-O-Feruloyl-β-d-glucose	Phenolic acids
Lmsn003644	1-O-Galloyl-6-O-Feruloyl-β-d-glucose	Phenolic acids
Lmsn001014	1-O-Galloyl-β-d-glucose*	Phenolic acids
pmb2871	1-O-Gentisoyl-β-D-glucoside*	Phenolic acids
Lmsn005321	1-O-p-Coumaroyl-2-O-galloyl-4,6-(*S*)-HHDP-β-d-glucose	Phenolic acids
pmb3068	1-O-p-Coumaroylquinic acid	Phenolic acids
pmn001419	1-O-p-Coumaroyl-β-d-glucose*	Phenolic acids
pmn001320	1-O-p-Cumaroylglycerol	Phenolic acids
Zbjn003642	1-O-rhamnose-3-O-Caffeoyl Quinic Acid	Phenolic acids
Lmsn002247	1-O-Salicyloyl-β-d-glucose*	Phenolic acids
HJN003	1-O-Sinapoyl-β-d-glucose	Phenolic acids
Lmtn002565	1-O-Vanilloyl-d-Glucose	Phenolic acids
Zbln003341	2-(2-(3,4-dihydroxyphenyl)-2-methoxyethoxy)-6-(hydroxymethyl)tetrahydro-2H-pyran-3,4,5-tri	Phenolic acids
Jmwn002117	2-(3,4-dihydroxyphenyl)ethanediol 1-O-β-D-glucopyranoside*	Phenolic acids
pme3083	2-(Formylamino)benzoic acid	Phenolic acids
MWSslk138	2,3,4-Trihydroxybenzoic acid	Phenolic acids
Lafp002342	2,3-Dihydroxy-1-(4-hydroxy-3,5-dimethoxyphenyl)propan-1-one	Phenolic acids
mws0639	2,3-Dihydroxybenzoic Acid*	Phenolic acids
MWSmce490	2′,4′,6′-Trihydroxyacetophenone	Phenolic acids
Xmbp001692	2,4,6-Trihydroxybenzoic acid	Phenolic acids
mws0885	2,4-Dihydroxybenzoic acid	Phenolic acids
Lmbn013410	2,4-Di-Tert-Butylphenol*	Phenolic acids
mws0180	2,5-Dihydroxybenzoic acid; Gentisic Acid*	Phenolic acids
Lmdp002901	2,6-Dihydroxy-4-isopropylphenyl-1-O-β-D-glucoside	Phenolic acids
MWSmce587	2,6-Dimethoxybenzoic acid	Phenolic acids
Wmyn000217	2,6-dimethoxy-hydroquinone-4-O-β-D-glucopyranoside	Phenolic acids
Lmln010063	2,6-Di-tert-butylphenol*	Phenolic acids
NK10253223	2-Amino-3-methoxybenzoic acid	Phenolic acids
MWSmce342	2-Caffeoyl-*L*-tartaric acid (Caftaric acid)	Phenolic acids
pmp000087	2-Feruloyl-sn-glycerol	Phenolic acids
Lmgp002452	2-Glucosyloxy-(4-hydroxyphenyl)acetic acid (Dhurrin acid)	Phenolic acids
Lmrn001951	2-Hydroxy-3-(4-Hydroxyphenyl)Propanoic Acid	Phenolic acids
MWS2001	2-Hydroxy-3,5-dinitrobenzoic acid	Phenolic acids
Lmrn003000	2-Hydroxy-3-phenylpropanoic acid	Phenolic acids
Lmbn001930	2-Hydroxybenzaldehyde (Salicylaldehyde)	Phenolic acids
Lmmn001643	2-Hydroxycinnamic acid*	Phenolic acids
Hmsn002948	2-Hydroxyphenol-1-O-glucosyl(6 → 1)rhamnoside	Phenolic acids
MWS2069	2-Methoxy-4-methylphenol	Phenolic acids
Zmyn002919	2-Methylbenzoic acid	Phenolic acids
MWS1875	2-Naphthol*	Phenolic acids
Zmzn002575	2-Nitrophenol	Phenolic acids
Zbfn003867	2-O-(3,4-dihydroxyphenylacetyl)-6-O-caffeoylglucoside	Phenolic acids
Zbfn004301	2-O-(4-carboxylic acid phenethyl)-6-O-caffeoyl glucoside	Phenolic acids
Wacn002584	2-O-Caffeoylhydroxycitric Acid	Phenolic acids
Lmhn102068	2-O-Caffeoylmalic acid	Phenolic acids
Hmln000873	2-O-Galloyl-d-glucose	Phenolic acids
Wacn003131	2-O-P-Coumaroylhydroxycitric Acid	Phenolic acids
HJN093	2-O-Salicyl-6-O-Galloyl-d-Glucose	Phenolic acids
Lakn001671	2-β-D-Glucopyranosyloxy-5-hydroxy-phenylacetic acid	Phenolic acids
Lakn003294	2-β-D-Glucopyranosyloxy-5-hydroxyphenylacetic acidmethylester*	Phenolic acids
HJAP051	3-(3,4,5-Trimethoxyphenyl)propan-1-ol	Phenolic acids
MWS3046	3-(3-Hydroxyphenyl)-3-hydroxypropanoic acid	Phenolic acids
mws0346	3-(3-Hydroxyphenyl)-propionic acid	Phenolic acids
MWSmce713	3-(4-Hydroxyphenyl)-1-propanol	Phenolic acids
mws0467	3-(4-Hydroxyphenyl)-propionic acid*	Phenolic acids
HJN102	3,4,5-Tricaffeoylquinic acid	Phenolic acids
pmn001517	3,4,5-Trimethoxyphenyl-1-O-Glucoside	Phenolic acids
Zmhn004909	3,4-Digalloylshikimic acid	Phenolic acids
Jmwp002208	3,4-dihydroxy phenylethanol	Phenolic acids
pme2598	3,4-Dihydroxybenzeneacetic acid*	Phenolic acids
mws0183	3,4-Dihydroxybenzoic acid (Protocatechuic acid)*	Phenolic acids
Jmwn002172	3,4-dihydroxyphenylethanol-β-D-glucopyranoside	Phenolic acids
Lckn000312	3,4-dihydroxy-β-phenylethoxy-O-α-L-rhamnopyranosyl(1 → 3)-6-O-caffeoyl(cis)-β-D-glucopyranoside*	Phenolic acids
MWSmce332	3,4-Dimethoxycinnamic acid	Phenolic acids
mws0612	3,4-Dimethoxyphenyl acetic acid	Phenolic acids
Zbjn003262	3,4-*O*-di-Caffeoyl-1-O-glucoside Quinic Acid	Phenolic acids
Wmzn002116	3,5-Dicaffeoylquinic acid	Phenolic acids
pmn001525	3,5-Digalloylshikimic acid	Phenolic acids
HJN037	3,5-Dihydroxy-4-methoxybenzoic acid; 4-O-Methylgallic Acid	Phenolic acids
MWSmce454	3,5-Dihydroxyacetophenone	Phenolic acids
MWS1944	3,5-Dihydroxytoluene	Phenolic acids
Lmsn004970	3,6-Di-O-caffeoyl glucose*	Phenolic acids
MA10107783	3-[(1-Carboxyvinyl)oxy]benzoic acid	Phenolic acids
Jmwn002494	3-[4-(β-D-glucopyranoside)-phenylacrylic]-acid	Phenolic acids
mws0444	3-Aminosalicylic acid	Phenolic acids
MWSHC20102	3-Hydroxy-1-(4-Hydroxy-3-Methoxyphenyl)Propan-1-One	Phenolic acids
pmn001690	3-Hydroxy-4-isopropylbenzylalcohol-3-O-glucoside	Phenolic acids
MWSslk066	3-Hydroxy-4-methoxybenzoic acid; Isovanillic Acid	Phenolic acids
pmn001521	3-Hydroxy-5-Methylphenol-1-O-(6’-Galloyl)Glucoside*	Phenolic acids
pmn001511	3-Hydroxy-5-Methylphenol-1-O-Glucoside	Phenolic acids
Lmgp002593	3-hydroxybenzaldehyde	Phenolic acids
Lmyn004439	3-Hydroxyl-5-methylphenol-1-O-β-D-(6′-galloyl)glucoside*	Phenolic acids
MWS4301	3-hydroxyphenylacetic acid*	Phenolic acids
MWS3149	3-Methoxybenzoic acid	Phenolic acids
Zmgn001989	3-Methylcatechol	Phenolic acids
MWS2058	3-Methylsalicylic Acid	Phenolic acids
WaYp004929	3-O-(p-coumaroyl) 3-Hydroxy-3-methylglutaric acid*	Phenolic acids
Zbln002999	3-O,6-*O*-bis(3-methoxy-4-hydroxy-trans-cinnamoyl)-β-D-fructofuranosyl2-O,6-*O*-diacetyl-α-D-glucopyranoside|1-O-Acetyl-3-O,6-*O*-bis[3-(4-hydroxy-3-methoxyphenyl)acryloyl]-β-D-fructofuranosyl1-O,2-O,6-*O*-triacetyl-α-D-glucopyranoside	Phenolic acids
Zaln004082	3-O-caffeoylshikimic acid*	Phenolic acids
pmb0752	3-O-Feruloylquinic acid	Phenolic acids
pmb2833	3-O-Feruloylquinic acid-O-glucoside*	Phenolic acids
Hmln000659	3-O-Galloyl-d-glucose*	Phenolic acids
MWSmce387	3-O-Methylgallic acid	Phenolic acids
Lmsp004151	3-O-p-Coumaroyl-4,6-(*S*)-HHDP-β-d-glucose	Phenolic acids
pmn001421	3-O-p-Coumaroylquinic acid*	Phenolic acids
pmb3064	3-O-p-Coumaroylquinic acid-O-glucoside	Phenolic acids
pmb3075	3-O-p-Coumaroylshikimic acid	Phenolic acids
pmb3072	3-O-p-Coumaroylshikimic acid-O-glucoside	Phenolic acids
Lmtn003598	3-Prenyl-4-O-glucosyloxy-4-hydroxybenzoic acid	Phenolic acids
Lmyn003835	4-(3,4,5-Trihydroxybenzoxy)benzoic acid	Phenolic acids
Walp002129	4-Acetoxy-3-Methoxybenzoic Acid glucoside	Phenolic acids
mws1336	4-Aminobenzoic acid	Phenolic acids
ML10177402	4-Aminosalicylic acid	Phenolic acids
Zaln004057	4-caffeoylshikimic acid*	Phenolic acids
Jmwn004308	4-Dihydroxyphenethoxy-8-O-[6-O-(4-O-glucosyl)feruloyl]glucoside	Phenolic acids
MWSslk155	4-Hydroxy-3-methoxymandelate	Phenolic acids
mws0749	4-Hydroxybenzoic acid	Phenolic acids
Wayn001856	4-Hydroxybenzoic acid 4-(6-O-sulfo)glucopyranoside	Phenolic acids
HJN025	4-Hydroxybenzyl Alcohol	Phenolic acids
MWSmce370	4-Hydroxyphenylacetic acid*	Phenolic acids
MWS5206	4-Hydroxyphenyllactic Acid*	Phenolic acids
pmb2795	4-Methoxycinnamic acid	Phenolic acids
Lmbn004847	4-Methoxyphenylpropionic acid	Phenolic acids
mws0566	4-Methylcatechol	Phenolic acids
Zmyn002321	4-Methylphenol	Phenolic acids
pme2828	4-Nitrophenol	Phenolic acids
Wafn002319	4-O-(3’-O-alpha-d-Glucopyranosyl)caffeoylquinic acid	Phenolic acids
Wafn002874	4-O-(4’-O-alpha-d-Glucopyranosyl)caffeoylquinic acid	Phenolic acids
Zmhn002643	4-O-(6’-O-Glucosyl-4″-hydroxybenzoyl)-4-hydroxybenzyl alcohol	Phenolic acids
Zbln003174	4”-O-Acetylverbascoside	Phenolic acids
Wdtp010604	4-Octylphenol-1-O-glucuronide	Phenolic acids
Lmqn000859	4-O-Glucosyl-2-hydroxy-6-methoxyacetophenone	Phenolic acids
Zmhn000892	4-O-Glucosyl-3,4-dihydroxybenzyl alcohol	Phenolic acids
Zmhn001358	4-O-Glucosyl-4-hydroxybenzoic acid*	Phenolic acids
Zmhn002227	4-O-Glucosyl-sinapate	Phenolic acids
pma6460	4-O-p-Coumaroylquinic acid	Phenolic acids
pma0110	4-O-Sinapoylquinic acid	Phenolic acids
Hmhn003518	4-O-β-D-glucopyranosylferulic acid	Phenolic acids
Hmtn001120	5-(2-Hydroxyethyl)-2-O-glucosylphenol	Phenolic acids
MWSmce001	5-Acetylsalicylic acid	Phenolic acids
pmn001516	5-Galloylshikimic acid	Phenolic acids
Lmmn003663	5-Glucosyloxy-2-Hydroxybenzoic acid methyl ester	Phenolic acids
Lmzn001582	5′-Glucosyloxyjasmanic acid	Phenolic acids
MWSHC20125	5-O-Caffeoylshikimic acid	Phenolic acids
pmb2554	5-O-Feruloyl quinic acid glucoside*	Phenolic acids
Lmgn003073	5-O-Feruloylquinic acid	Phenolic acids
Zmhn001703	5-O-Galloyl-D-hamamelose*	Phenolic acids
pmb3074	5-O-p-Coumaroylquinic acid*	Phenolic acids
Ymjm000116	6-O-Acetylarbutin	Phenolic acids
Lman003972	6-O-Caffeoylarbutin	Phenolic acids
Zmhn001793	6-O-Caffeoyl-d-glucose*	Phenolic acids
Hmbn002692	6’-O-Feruloyl-D-sucrose	Phenolic acids
Zmhn002334	6-O-Feruloyl-β-d-glucose	Phenolic acids
Lmfn000604	6-O-Galloyl-β-d-glucose*	Phenolic acids
Lmsn003628	6’-O-Sinapoylsucrose	Phenolic acids
Zmxn003874	7,8-Dihydro-Buddlenol B	Phenolic acids
Smcn001947	Acteoside; Verbascoside*	Phenolic acids
Lmqp002761	Alnusonol	Phenolic acids
Lmtn002233	Androsin	Phenolic acids
pmb2654	Anthranilate-1-O-Sophoroside	Phenolic acids
mws1078	Anthranilic Acid	Phenolic acids
MWSmce675	Arbutin	Phenolic acids
Labn003910	Benzyl B-Primeveroside*	Phenolic acids
mws1297	Benzyl glucoside	Phenolic acids
Hmln003529	Benzyl β-primeveroside	Phenolic acids
Lmtn002324	Benzyl-(2”-O-glucosyl)glucoside*	Phenolic acids
Cmjn004337	Benzyl-(2”-O-xylosyl)glucoside*	Phenolic acids
Lmyn003028	Benzyl-β-gentiobioside*	Phenolic acids
Lmwp011196	Bis(2-ethylhexyl)phthalate*	Phenolic acids
mws2120	Brevifolin carboxylic acid	Phenolic acids
MWSmce341	Butyl isobutyl phthalate*	Phenolic acids
mws2212	Caffeic acid	Phenolic acids
MWSCX015	Caffeic aldehyde	Phenolic acids
Zbfn002396	Caffeoyl-O-mannitol	Phenolic acids
Labn003679	Caffeyl alcohol 4-O-β-D-glucopyranoside	Phenolic acids
MWS0550	Cinnamaldehyde	Phenolic acids
mws2213	Cinnamic acid	Phenolic acids
mws0906	Coniferin	Phenolic acids
mws0093	Coniferyl alcohol	Phenolic acids
Hmsn002272	Demethyl coniferin	Phenolic acids
Lmlp012720	Dibutyl phthalate*	Phenolic acids
Lmgp003989	Dicaffeoylshikimic acid	Phenolic acids
pmn001513	Digallic Acid	Phenolic acids
Lmlp001436	Dihydrocaffeic acid	Phenolic acids
Lmmn000774	Dihydrocaffeoylglucose*	Phenolic acids
Lmxp011770	Diisobutyl phthalate*	Phenolic acids
Lmmp010562	Diisooctyl Phthalate*	Phenolic acids
MWSslk097	Dimethyl phthalate	Phenolic acids
Lmzn001983	D-Threo-guaiacylglycerol-7-O-β-D-glucoside	Phenolic acids
Lazn001573	Erythro-Guaiacylglycerol	Phenolic acids
mws2184	Ethyl caffeate	Phenolic acids
MWSslk006	Ethyl Vanillate	Phenolic acids
mws0014	Ferulic acid*	Phenolic acids
Hmmn002544	Ferulic acid-4-O-glucoside	Phenolic acids
pmb0108	Feruloyl syringic acid	Phenolic acids
Lmhn003074	Feruloylmalic acid	Phenolic acids
mws0024	Gallic acid	Phenolic acids
pmb2928	Gallic acid-4-O-glucoside	Phenolic acids
Hmtn001302	Glucosyloxybenzoic acid	Phenolic acids
MWSHC2022	Glucosyringic acid	Phenolic acids
Cmyp005063	Grandidentatin	Phenolic acids
pme1292	Homogentisic acid*	Phenolic acids
WaYn002313	Homosyringic Acid 4’-O-Glucoside	Phenolic acids
mws0117	Homovanillic acid; 4-Hydroxy-3-methoxyphenylacetic acid	Phenolic acids
pmb3056	Homovanilloylquinic acid	Phenolic acids
Lmyn002435	Hydrangeifolin I	Phenolic acids
MA10014775	Hydroquinone	Phenolic acids
Wafn003070	Hydroxyferulic acid glucoside	Phenolic acids
MWSmce264	Hydroxytyrosol	Phenolic acids
Hmgp001944	Isoacteoside	Phenolic acids
pme0422	Isoferulic Acid*	Phenolic acids
Lmmn001294	Koaburaside*	Phenolic acids
Li512113	Maleoyl-caffeoylquinic acid	Phenolic acids
MWS1776	m-Cresol	Phenolic acids
MWSmce680	Mequinol	Phenolic acids
Hmtn001288	Methyl 2,4-dihydroxyphenylacetate*	Phenolic acids
Lmbn004790	Methyl 3-(3-hydroxy-4-methoxyphenyl)propanoate*	Phenolic acids
Lmcn002982	Methyl 3-O-Methyl Gallate	Phenolic acids
Zmgn004894	Methyl 4-hydroxybenzoate*	Phenolic acids
Wafn002807	methyl 4-O-galloylchlorogenate	Phenolic acids
Hmdn001963	Methyl Brevifolincarboxylate	Phenolic acids
Lmdn003756	Methyl caffeate	Phenolic acids
MWSHC20168	Methyl cumalate*	Phenolic acids
MWSmce230	Methyl gallate	Phenolic acids
Wbsp004755	Methyl Hydroxycinnamate	Phenolic acids
Lmmn002179	Methylsalicylate-2-O-glucoside	Phenolic acids
mws0145	O-Anisic acid (2-Methoxybenzoic acid)*	Phenolic acids
pme1439	p-Coumaric acid*	Phenolic acids
MWSslk144	p-Coumaric acid ethyl ester	Phenolic acids
mws1195	p-Coumaric acid methyl ester	Phenolic acids
Zmhn002301	p-Coumaric acid-4-O-glucoside*	Phenolic acids
Lmhn002926	p-Coumaroylmalic acid	Phenolic acids
pmb3055	p-Coumaroylquinic acid-4’-O-glucuronide	Phenolic acids
MWS1846	Phenoxyacetic acid*	Phenolic acids
MWS1848	Phenyl acetate	Phenolic acids
Hmhn003067	Phenylpropionic acid-O-β-D-glucopyranoside	Phenolic acids
NK10264324	Phloroglucinol; 1,3,5-Benzenetriol	Phenolic acids
Hmhn000927	Phloroglucinol-1-O-β-D-glucopyranoside	Phenolic acids
pme0282	Phthalic acid	Phenolic acids
pmp001285	Phthalic anhydride	Phenolic acids
Lmln001195	Picein (4-Acetylphenyl-glucoside)	Phenolic acids
mws1506	Plantamajoside	Phenolic acids
MWS2070	Propyl 4-hydroxybenzoate	Phenolic acids
Wasn004683	Protocatechuic acid 1-O-(Glucosylvanilloyl)	Phenolic acids
Wasn005073	Protocatechuic acid 4-O-(2”-O-Vanilloyl)Glucoside	Phenolic acids
Lakn002165	Protocatechuic acid glucosyl xyloside	Phenolic acids
MWSmce501	Protocatechuic Acid Methyl Ester	Phenolic acids
pmn001367	Protocatechuic acid-4-O-glucoside*	Phenolic acids
mws0025	Pyrogallol	Phenolic acids
pmb3317	Quinacyl syringic acid	Phenolic acids
pmn001710	Rosmarinic acid-3’-O-glucoside	Phenolic acids
mws1521	Salicin	Phenolic acids
MWSmce274	Salicyl Alcohol	Phenolic acids
Lmgn001670	Salicylic acid	Phenolic acids
pmb3142	Salicylic acid-2-O-glucoside	Phenolic acids
mws2367	Salidroside	Phenolic acids
Hmsn002598	Salirepin	Phenolic acids
Lmbp002309	Sinapaldehyde-4-O-Glucoside	Phenolic acids
mws4085	Sinapic acid	Phenolic acids
pme3443	Sinapinaldehyde	Phenolic acids
pma0149	Sinapoyl malate	Phenolic acids
Lmgn004359	SinapoylcaffeoylQuinic acid O-glucose	Phenolic acids
mws0853	Sinapyl alcohol*	Phenolic acids
Zmln000899	Syringaldehyde-4-O-glucoside	Phenolic acids
mws0027	Syringic acid	Phenolic acids
pmb0824	Syringic acid-4-O-(6″-feruloyl)glucoside	Phenolic acids
mws0011	Syringin	Phenolic acids
Lmgn002253	Syringoylcaffeoylquinic acid-d-glucose	Phenolic acids
Hmqn000843	Tachioside	Phenolic acids
pme0281	Terephthalic acid	Phenolic acids
pmb0751	Trans-5-O-(p-Coumaroyl)shikimate	Phenolic acids
pmn001523	Trigallic acid	Phenolic acids
mws2368	Tyrosol; 4-Hydroxyphenylethanol	Phenolic acids
MWSslk092	Usnic acid	Phenolic acids
mws0028	Vanillic acid	Phenolic acids
Lmqp002627	Vanillic acid methyl ester	Phenolic acids
Zmhn001883	Vanillic acid-4-O-glucoside	Phenolic acids
Jmwn002620	Vanillic Acid-4-O-Glucuronide	Phenolic acids
MWSmce632	Vanillin acetate	Phenolic acids
Zmtn001661	Vanilloloside	Phenolic acids
Lmhn002321	Vnilloylcaffeoyltartaric acid	Phenolic acids
Lmhn002051	Vnilloylmalic acid	Phenolic acids
Lmhn001386	Vnilloyltartaric acid	Phenolic acids
Lmbn002648	α-Hydroxycinnamic Acid*	Phenolic acids
Zbln001290	β-Hydroxy-(3,4-dihydroxyphenylethanolyl)-glucoside	Phenolic acids
Hmln002996	β-Hydroxyacteoside	Phenolic acids
Hmgn002038	β-Oxoacteoside	Phenolic acids
MA10079134	1,2,4-Trihydroxyanthraquinone	Quinones
MWS20169	2,5-dihydroxy-1-methoxy-anthraquinone	Quinones
Zbn004863	3-Hydroxymorindone-6β-primeveroside	Quinones
Zbn006783	5,6-Dihydroxylucidin	Quinones
Zbn004415	5,6-Dihydroxylucidin-3β-primeveroside	Quinones
Zmdn006929	Aloe-Emodin-9-Anthrone	Quinones
Zdhn005610	Citreorosein	Quinones
Wcfn008271	Danthron; 1,8-Dihydroxyanthraquinone	Quinones
Zbqn006094	Pseudopurpurin(1,2,4-trihydroxy-3-carboxyanthraquinone)	Quinones
Wmcp000011	Rheic Acid	Quinones
pmn001338	Rhein-8-O-glucoside	Quinones
Zbln013951	1-(3′-methoxy-4′-hydroxybenzyl)-2,7-dihydroxy-4-methoxyphenanthrene	Quinones
Zbln007388	4,6-dimethoxy-9,10-dihydrophenanthrene-2,3,7-triol	Quinones
Lmqp003022	2,3-Dihydro-1,4-naphthoquinone	Quinones
Wmhp000055	2,5-Dimethoxybenzoquinone*	Quinones
Zmsp001834	2,6-Dimethoxy-1,4-benzoquinone*	Quinones
Wchn003984	1,4,8-Trihydroxynaphthalene-1-O-glucoside*	Quinones
pme0432	Procyanidin A2	Tannins
mws0836	Procyanidin B1	Tannins
MWSHY0171	Procyanidin B2	Tannins
pme0436	Procyanidin B3	Tannins
pmn001667	Procyanidin B4	Tannins
Lssp210081	Procyanidin B5	Tannins
pmn001646	Procyanidin C1	Tannins
pmn001647	Procyanidin C2	Tannins
Zmlp005013	3,3’-*O*-Dimethylellagic Acid	Tannins
Lmqn004838	3-O-Methylellagic acid	Tannins
Hmdn001667	Ellagic acid-4-O-glucoside	Tannins
Lmyn004187	Flavogallonic Acid Dilactone	Tannins
mws1629	Aucubin	Terpenoids
mws1565	Geniposide	Terpenoids
mws1574	Geniposidic acid*	Terpenoids
Lafp004533	(6*R*,9*R*)-3-Oxo-α-ionol-β-*D*-malonyl-glucoside	Terpenoids
Qmjp080407	14(15)-Bisnor-13-oxolabd-8(17),11(*E*)-dien-19-oic acid	Terpenoids
Qmjp080402	15,16-Bisnor-13-oxo-8(17),11-labdadien-19-ol	Terpenoids
Wdzp007419	1β-hydroxy-β-cyperone	Terpenoids
Wbmp003788	3,4-Dihydroxy-7,8-dihydro-β-ionone-4-O-β-D-glucoside	Terpenoids
Labp004808	3-Oxo-Alpha-Ionol diglucoside	Terpenoids
Wcfp005519	5-hydroxyindene-1,3-dione	Terpenoids
Lmqp003432	6,9-Dihydroxy-7-megastigmen-3-one	Terpenoids
Hmcn000773	6’-O-Glucosylaucubin	Terpenoids
Zjyp102922	Humula-3(12),7(13),9(E)-triene-2,6-diol	Terpenoids
MWSmce096	Nootkatone	Terpenoids
Hmlp013407	β-Eudesmol	Terpenoids
Cmbn004962	10-O-[(E)-p-Coumaroyl]-geniposidic acid	Terpenoids
Qmdp112605	1-Oxo-Eucommiol	Terpenoids
Lmdn003075	3,4-Dihydroverbenalin	Terpenoids
Cmbn003318	5-Hydroxy-10-O-(p-methoxycinnamoyl)adoxosidic acid	Terpenoids
Lmdn001560	6-DeoxyCatalpol	Terpenoids
Zbbn002910	6”-O-sinapoyl-7-O-caffeoyl-geniposide	Terpenoids
pmp001070	6”-O-Trans-Sinapoylgenipin gentiobioside	Terpenoids
Wbmn003453	6-O-Vanilloylajugol	Terpenoids
Hmmn003964	7-Deoxyloganic acid	Terpenoids
Sacn001940	8-Epiloganic acid	Terpenoids
MWSslk186	8-O-Acetylharpagide	Terpenoids
pmn001587	Asperulosidic acid	Terpenoids
pmp001045	Bartsioside	Terpenoids
Hmlp008487	Blumenol C	Terpenoids
mws1562	Catalpol	Terpenoids
MWS1962	cis-Citral	Terpenoids
pmn001586	Deacetylasperulosidic acid	Terpenoids
MWSHC20122	Dehydrovomifoliol	Terpenoids
MWSmce434	Gardenoside	Terpenoids
Lmdn001501	Gardoside*	Terpenoids
MWSmce115	Genipin	Terpenoids
pmp001059	Genipin-1-O-(2”-O-apiosyl)glucoside	Terpenoids
Lhjp111633	Glucosylasperuloside	Terpenoids
Wmbn000547	Harpagide	Terpenoids
pmp001048	Ixoroside	Terpenoids
pmp000691	Loganetin	Terpenoids
Zbyn002745	Loganic acid	Terpenoids
Lmjp004816	Loliolide	Terpenoids
pmn001585	Monotropein	Terpenoids
Cmrn001591	Mussaenosidic acid	Terpenoids
Lmyp004084	Perillyl alcohol	Terpenoids
Cmhp003670	Secologanin	Terpenoids
pmp001053	Shanzhiside	Terpenoids
mws1526	Sweroside	Terpenoids
Lmpn004676	Verminoside*	Terpenoids
Hmmp003004	Vogeloside	Terpenoids
mws1429	Vomifoliol (Blumenol A)	Terpenoids
Labn003976	Vomifoliol 9-[Xylosyl-(1 → 6)-Glucoside]	Terpenoids
mws1515	α-Ionone	Terpenoids
pmf0314	cis-Abienol	Terpenoids
Cmmn012461	Dehydroabietic acid	Terpenoids
MWSslk208	Kaurenoic Acid*	Terpenoids
Lmbn014696	Pimaric acid*	Terpenoids
Wbmn011269	Vitexilactone	Terpenoids
Llhp011701	11,12-epoxy-13-hydroxy-3-Oxooleanane-28-oic acid gamma-lactone (Liquidambaric Lactone)	Terpenoids
pmn001427	16,23:16,30-Diepoxydammar-24-ene-3,20-diol (Jujubogenin)*	Terpenoids
Lskp211493	2,19-Dihydroxy-3-oxo-24-norolean-12-en-28-oic acid	Terpenoids
pmn001426	2,3,19-trihydroxyurs-12-en-28-oic acid (Euscaphic acid)*	Terpenoids
MWSmce394	2,3,19-Trihydroxyurs-12-en-28-oic acid (Tormentic acid)*	Terpenoids
Smpn009230	2,3,23-Trihydroxyolean-12-en-28-oic acid*	Terpenoids
Lmqp008286	2,3,23-Trihydroxyolean-12-en-28-oic acid (Arjunolic acid)	Terpenoids
Hmjn003948	2,3,6-Trihydroxyurs-12-en-28-oic acid (Madasiatic acid)*	Terpenoids
Wbmn009545	2,3-Diacetoxy-18-hydroxyoleana-5,12-dien-28-oic acid	Terpenoids
Smpn011792	2,3-Dihydroxy-12-ursen-28-oic acid*	Terpenoids
Lmzn106284	2,3-Dihydroxylup-20(29)-en-28-oic acid (Alphitolic acid)*	Terpenoids
pmn001706	2,3-Dihydroxyolean-12-en-28-oic acid (2-Hydroxyoleanolic acid)*	Terpenoids
Hmjn008136	2,3-Dihydroxyoleana-11,13(18)-dien-28-oic acid (Camaldulenic acid)	Terpenoids
Lmsn012627	2,3-Dihydroxyurs-12,18-dien-28-oic acid	Terpenoids
Zmpn008194	2,3-Dihydroxyurs-12-en-28-oic acid (Corosolic acid)*	Terpenoids
Li512114	2,3-Dihydroxyurs-12-en-28-oic acid methyl ester (Corosolic acid methyl ester)	Terpenoids
mws1610	2,3-Dihydroxyurs-12-en-29-oic acid (Maslinic acid)*	Terpenoids
Lssp210085	3,11-dioxo-β-oleorene	Terpenoids
pmn001591	3,19,23-Trihydroxyurs-12-en-28-oic acid (Rutundic acid)	Terpenoids
Lmzn006169	3,19-Dihydroxyurs-12-en-28-oic acid (Pomolic acid)*	Terpenoids
Lhnp110101	3,20-Dihydroxyurs-21-en-28-oic acid (Oleanderic acid)	Terpenoids
Hmbn005207	3,23-Dihydroxyolean-12-en-28-oic acid (Hederagenin)*	Terpenoids
pmp000441	3-Hydroxy-11-oxours-12-en-28-oic acid (11-Keto-ursolic acid)	Terpenoids
MWSHC20176	3-Hydroxylup-20(29)-en-28-oic acid (Betulinic acid)	Terpenoids
mws1389	3-Hydroxyolean-12-en-28-oic acid (Oleanolic acid)*	Terpenoids
Lmsp102910	3-Hydroxyolean-12-ene-27,28-dioic acid (Cincholic acid)	Terpenoids
MWSmce052	3-Hydroxyurs-12-en-28-oic acid (3-Epiursolic acid)	Terpenoids
Lssp210084	3-Hydroxyurs-12-en-28-oic acid (Ursolic acid)	Terpenoids
Lmmn009550	3-Oxo-9,19-cyclolanost-24-en-26-oic acid (Mangiferonic acid)*	Terpenoids
MWSmce134	3-Oxolup-20(29)-en-28-oic acid (Betulonic acid)*	Terpenoids
Lmyn012771	3-Oxoolean-18-en-28-oic acid (Moronic acid)*	Terpenoids
Ymjm000099	3-Oxooleana-11,13(18)-dien-28-oic acid	Terpenoids
Lmzn006795	3-Oxours-12-en-28-oic acid (Ursonic acid)*	Terpenoids
Lmhn011365	Morolic acid*	Terpenoids
Hmcp005697	Olean-13(18)-en-3-one (δ-Amyrenone)	Terpenoids
Lmqn012798	Urs-12(13)-en-3-one-28-oic acid*	Terpenoids
Lmxn001694	6”-O-β-D-Glucosyl-8-*O*-acetylharpagide	Terpenoids
Lmxn003152	8-Epiloganin	Terpenoids
Zadn003162	Ajugoside	Terpenoids
Smhn002274	Blumenol C glucoside; Byzantionoside B*	Terpenoids
Zadp003164	Dehydrololiolide	Terpenoids
pmn001381	Eucommioside	Terpenoids
Zmdn003368	eucomoside A	Terpenoids
Zmdn004697	eucomoside B	Terpenoids
Zmdn004845	eucomoside C	Terpenoids
Lmlp002205	Isololiolide	Terpenoids

Based on the above results, a non-supervised PCA model was conducted to further examine QC and tested samples (Fig. 2D). The four groups of EUL were distinctly differentiated, with the QC samples located in the center of all tested samples. This result validated reliability and reproducibility of this experiment. Additionally, the result confirmed that non-volatile metabolites determined in the four groups of EUL were obviously distinct. The significant separation of BT in PC1 (40.9 %) is attributed to fermentation-specific metabolites such as esters and phenolic acids, while the differences between GT and HG in PC2 (16.53 %) stem from the impact of thermal processing on lipid metabolism.This result suggested that the BT was the most different from the other groups, followed by the GT and HG, while the metabolites were more similar within the other groups.

### 3.3 Differential metabolites in XY, HG, BT, GT

**Supplementary fig S1:**
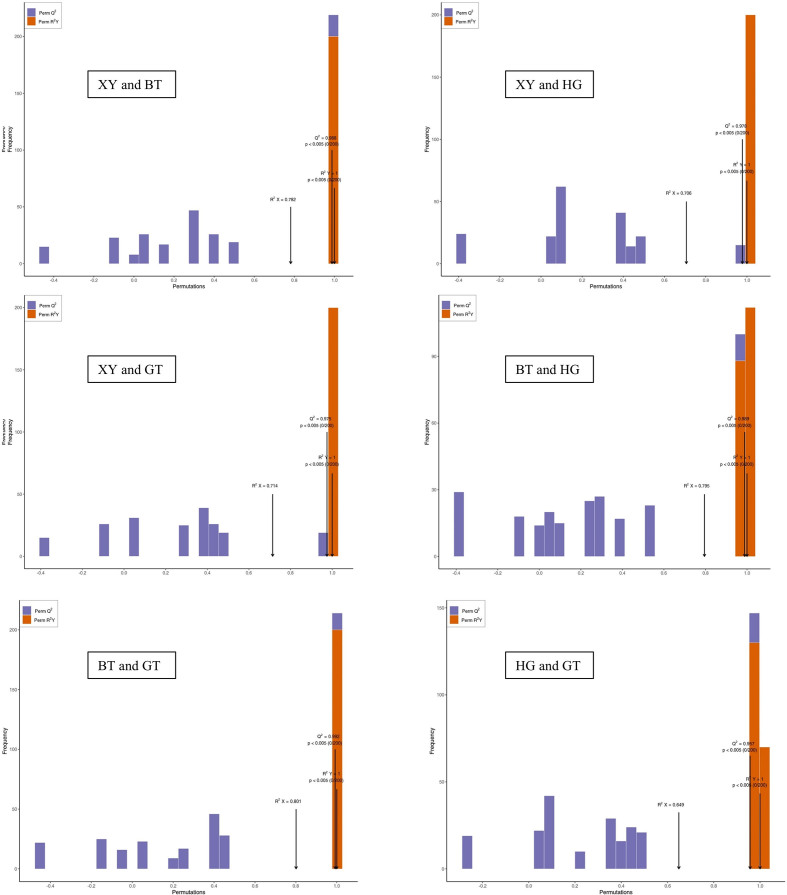
OPLS-DA model validation plot; Note: The horizontal coordinates represent the model R^2^Y，Q^2^ values, and the vertical coordinates are the frequency of the model classification effects in 200 random permutation experiments. In the figure, the orange color represents the random grouping model R^2^Y, the purple color represents the random grouping model Q^2^, and the values represented by the black arrows are the R^2^X, R^2^Y, and Q^2^ values of the original model.

The OPLS-DA analysis demonstrated statistically significant differences, filtering the four samples for substantial differences in nonvolatile metabolites based on conditions of VIP ≥ 1, FC ≥ 2(or ≤ 0.5) and *p* < 0.05. Prior to this further analysis, the OPLS-DA analysis needs to be validated. The Q^2^ values between XY and BT, XY and HG, XY and GT, BT and HG, BT and GT, and HG and GT were 0.988, 0.978, 0.975, 0.989, 0.992, and 0.957, respectively (Fig. S1). Q^2^ represents the predictive power, while R^2^Y and R^2^X reveal the explanatory power of the Y and X matrices, separately. All pairwise comparisons of R^2^Y and Q^2^ scores turned out to be higher than 0.9, which implies that the model is good. In addition, a total of 200 randomized permutation tests were also conducted to validate the OPLS-DA model, and the *p*-values were found to be below 0.005, indicating an excellent model. Therefore, the implemented OPLS-DA model is stable, meaningful and can be used for further screening of differential metabolites.

**Fig. 3:**
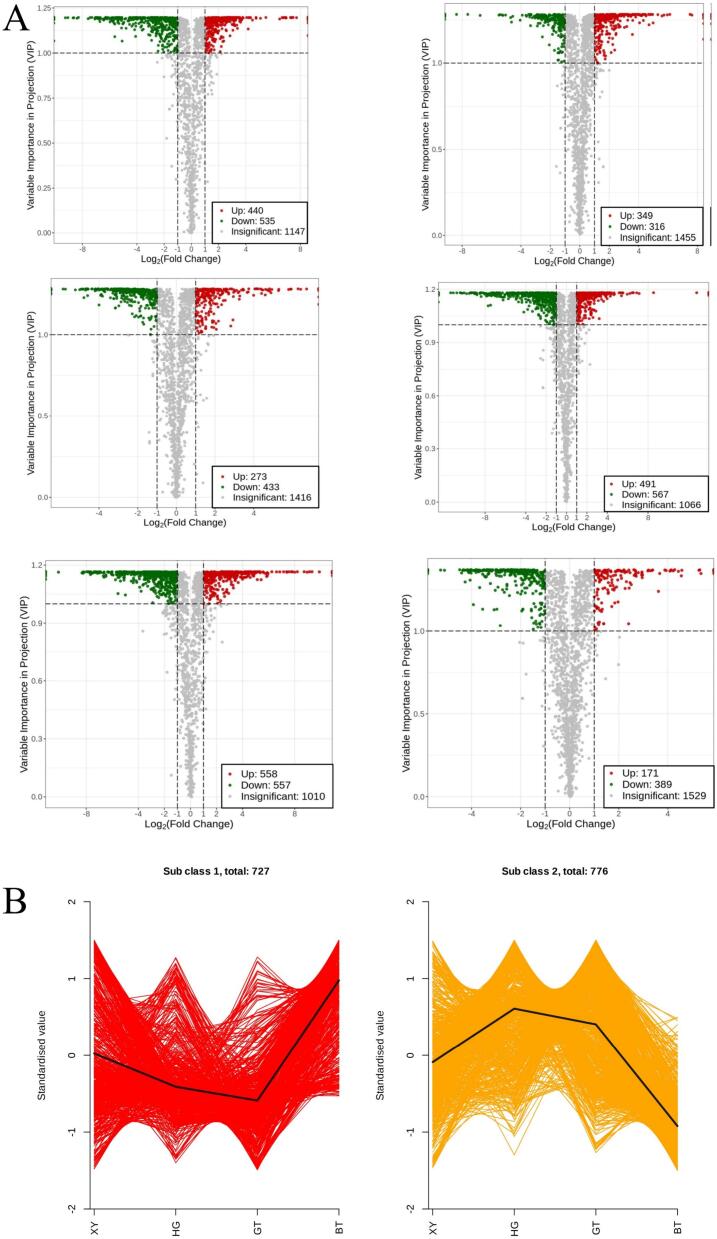
**A.** Volcano plots of nonvolatile metabolites in the two-by-two comparison groups, in order of BT vs. XY, HG vs. XY, GT vs. XY, BT vs. HG, BT vs.GT, HG vs.GT. Note: Each point in the volcano diagram represents a metabolite, where green points represent down-regulated differential metabolites, red points represent up-regulated differential metabolites, and gray points represent metabolites that were detected but the difference was not significant; the horizontal coordinate represents the logarithm of the multiplicity of the difference of the relative content of a metabolite between the two groups of samples (log_2_). FC), the larger the absolute value of the horizontal coordinate, the larger the difference in relative content of the substance between the two groups of samples. VIP + FC + *P*-value screening conditions: the vertical coordinate indicates the significance level of the difference (−log_10_ P-value), the size of the dot represents the VIP value. VIP + FC screening condition: the vertical coordinate represents the VIP value, the larger the value of the vertical coordinate, the more significant the difference is, and the more reliable the differential metabolite obtained by screening. **B.** Horizontal coordinates represent the grouping of samples, vertical coordinates represent the normalized relative metabolite content, Sub class represents the number of metabolite classes with the same trend, and total: represents the number of metabolites in the class.

The data of OPLS-DA results were combining the grouping of specific samples and visualizing the results through volcano plots (Fig.3A). A total of 975 (440 up-regulated) metabolites were found to be significantly different between BT and XY, 665 (349 up-regulated) between HG and XY, 706 (273 up-regulated) between GT and XY, 986 (419 up-regulated) between BT and HG, 1115 (558 up-regulated) between BT and GT, and 560 (171 up-regulated) between GT and HG. In addition, two different trends were found for the differential metabolites screened in the four samples of this study (Fig. 3B). For example, one part of the metabolites in BT showed higher levels than in XY, while the other half of the metabolites in BT showed lower levels than in XY.

**Fig. 4:**
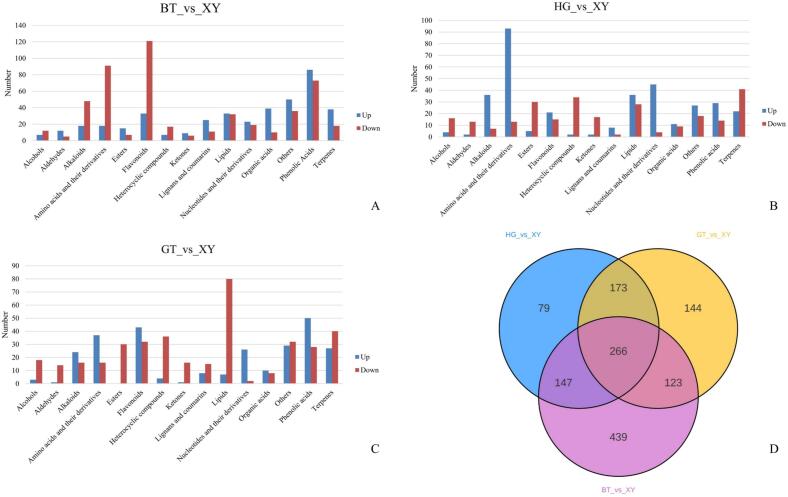
Variations of metabolites groups in different samples. A, B, C shows the up/down and number of adjustments bars for BT vs. XY, HG vs. XY, and GT vs XY., respectively. D: Venny for HG vs. XY and GT vs. XY and RT vs. XY.

**Supplementary fig S2:**
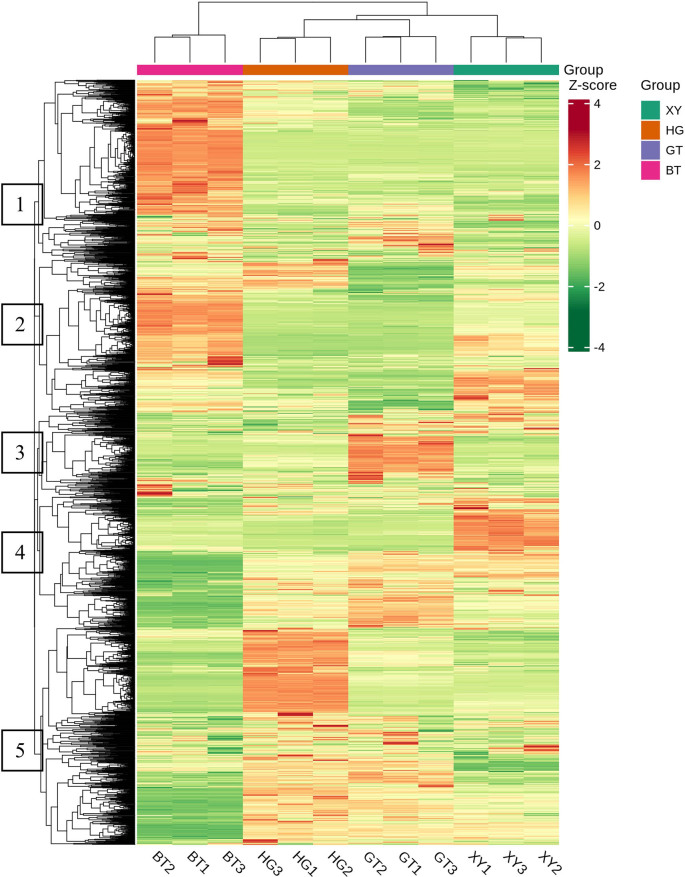
Heat map of cluster analysis of different processing processes of EUL；Note: Horizontal is the sample name, vertical is the metabolite information, Group is the grouping, and different colors are filled with different values obtained by normalizing different relative contents (red for high, green for low).

**Supplementary fig S3:**
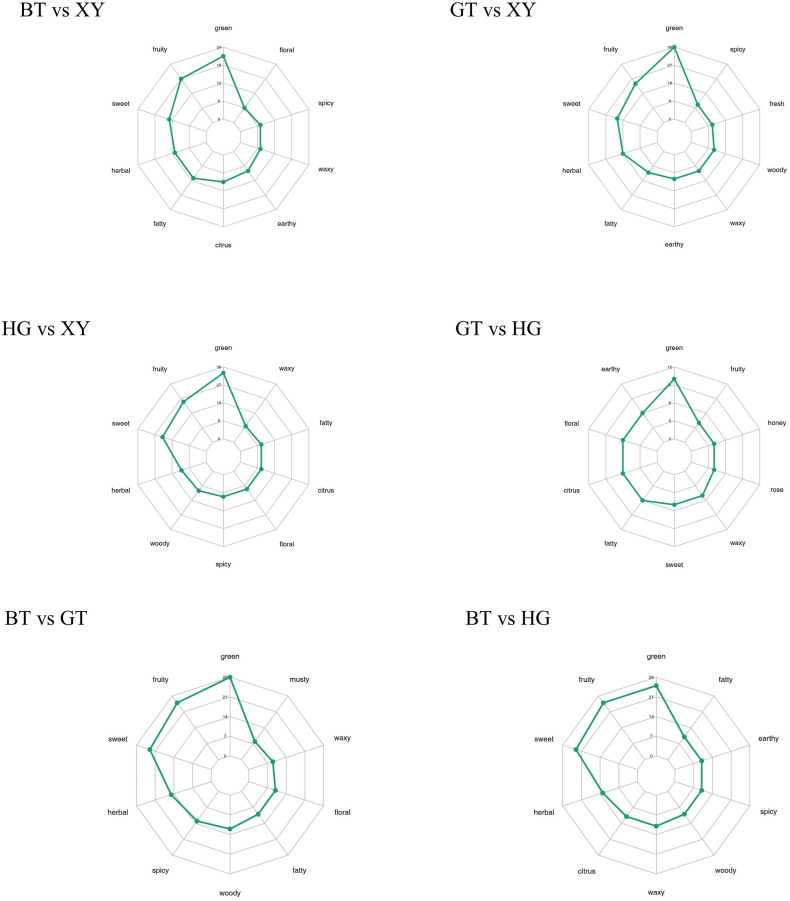
Thermograms of non-volatile metabolites of various classes of differently processed Cortex Eucommia leave. (A-K represent one class of substances each. A: Amino acids and their derivatives; B: Lipids; C: Flavonoids; D: Phenolic acids; E: Nucleotides and their derivatives; F: Alkaloids; G: Organic acids; H: Lignans: I: Tannins; J: Terpenoids; K: Other classes).

To better summarize and analyze the dynamics of metabolites between different treatments of EUL, a clustered heat map analysis was performed as in Supplemental Fig. S2 & Fig. S3 A-K. These differentially regulated nonvolatile metabolites were mainly focusing on flavonoids, phenolic acids, amino acids and their derivatives (Table 3). As shown in Fig. 4, the classification of significant differences in the comparison between BT vs. XY showed that alkaloids, amino acids and their derivatives, and flavonoids were significantly down-regulated, with the number of flavonoids down-regulated reaching more than 120. While phenolic acids, organic acids, and other classes existed significant up-regulation. The withering process during black tea processing can result in a highly significant positive correlation between free amino acids in the withered leaves and flavonoids in the finished black tea. This explains that amino acids and their derivatives are correlated with the decreasing trend of both flavonoids. The decrease in flavonoids is also consistent with previous experimental studies by Li et al. that the fermentation process leads to this result(Li et al., 2017). Furthermore, in the HG vs. XY, besides the significant increase in the number of up-regulated amino acids and their derivatives after the drying process, reaching more than 90 species, there was also a more pronounced up-regulation of lipids, nucleotides and their derivatives, and alkaloids. While the down-regulation of heterocyclic compounds and terpenoids increased in the number. This indicates that drying is very favorable for the production of amino acids and their derivatives, but not for terpenoids and heterocyclic compounds. In the GT vs. XY, the number of down-regulation of lipids was the most significant, reaching almost 80. While the number of up-regulation of phenolic acids, flavonoids, amino acids and their derivatives was significantly higher. It indicates that the GT processing leads to a decrease in the content of lipids, but an increase in the content of some phenolic acids and flavonoids.

**Table 3:** Table of major differences in composition between groups of non-volatile metabolites of different processed EUL.

Index	Compounds	Class	BT_vs_XY_Type	GT_vs_XY_Type	HG_vs_XY_Type
mws0178	Chlorogenic acid (3-O-Caffeoylquinic acid)*	Phenolic acids	down	–	–
mws2108	Cryptochlorogenic acid (4-O-Caffeoylquinic acid)*	Phenolic acids	down	–	–
pmn001382	Isochlorogenic acid A*	Phenolic acids	–	up	–
Li512115	Isochlorogenic acid B*	Phenolic acids	–	up	–
pmn001384	Isochlorogenic acid C*	Phenolic acids	–	up	–
MWSprf004	Neochlorogenic acid (5-O-Caffeoylquinic acid)*	Phenolic acids	down	–	–
mws1358	Pyrocatechol	Phenolic acids	–	up	–
mws1068	Kaempferol (3,5,7,4’-Tetrahydroxyflavone)	Flavonoids	up	down	down
MWSHY0136	Kaempferol-3-O-glucoside (Astragalin)*	Flavonoids	down	–	–
pme2954	Quercetin	Flavonoids	up	–	–
mws0091	Quercetin-3-O-glucoside (Isoquercitrin)	Flavonoids	down	–	–
mws0045	Quercetin-3-O-rhamnoside(Quercitrin)	Flavonoids	down	–	–
MWSHY0065	Catechin*	Flavonoids	down	up	–
Lajp002810	Procyanidin A4	Flavonoids	down	–	–
pmn001370	Pinoresinol-4,4’-O-diglucoside	Lignans and Coumarins	down	–	–
mws0836	Procyanidin B1	Tannins	down	–	–
MWSHY0171	Procyanidin B2	Tannins	down	–	–
pme0436	Procyanidin B3	Tannins	down	–	–
pmn001667	Procyanidin B4	Tannins	down	–	–
Lssp210081	Procyanidin B5	Tannins	down	–	–
pmn001646	Procyanidin C1	Tannins	down	–	–
pmn001647	Procyanidin C2	Tannins	down	–	–
mws1629	Aucubin	Terpenoids	down	–	–
mws1565	Geniposide	Terpenoids	–	–	–
mws1574	Geniposidic acid*	Terpenoids	down	–	–
MWS00330g	1-Amino-1-cyclobutane-carboxylic-acid*	Amino acids and derivatives	–	–	up
MWS0933	1-Methylhistidine*	Amino acids and derivatives	up	–	–
Zmmp001941	2-Amino-3,4-dihydroxybutanoic acid-3-O-arabinoside	Amino acids and derivatives	down	–	up
Zmdp000441	3-(Allylsulphinyl)-L-alanine	Amino acids and derivatives	–	–	up
MWS04491	3,4-Dehydro-DL-proline	Amino acids and derivatives	–	down	–
pme2914	3-Hydroxy-3-methylpentane-1,5-dioic acid	Amino acids and derivatives	up	–	–
MWS04559g	3-Hydroxy-*L*-phenylalanine*	Amino acids and derivatives	down	down	–
MWS2010	3-nitro-L-tyrosine	Amino acids and derivatives	down	–	–
Zbqp003444	4-amino-5-oxo-5-(pentylamino)pentanoic acid	Amino acids and derivatives	down	–	–
pme2758	4-Hydroxy-L-glutamic acid	Amino acids and derivatives	down	–	up
MWSmce190	4-Hydroxy-L-Isoleucine	Amino acids and derivatives	down	–	–
mws0263	5-Oxo-L-Proline*	Amino acids and derivatives	–	up	–
MWS0813	5-Oxoproline*	Amino acids and derivatives	–	up	–
MWS201384	Arg-Gly	Amino acids and derivatives	–	up	up
Zmdp000292	Arginine methyl ester*	Amino acids and derivatives	down	up	up
MWS201391	Asn-Ile	Amino acids and derivatives	down	–	up
MWS201449	Asn-Leu	Amino acids and derivatives	down	–	up
MWS201455	Asp-Lys*	Amino acids and derivatives	–	–	up
pmf0470	cis-4-Hydroxy-D-proline*	Amino acids and derivatives	down	–	up
MWStz261	Cyclo(D-Leu-L-Pro)*	Amino acids and derivatives	–	up	up
MWStz205	Cyclo(D-Phe-L-Pro)*	Amino acids and derivatives	–	up	–
MWStz083	Cyclo(D-Val-L-Pro)	Amino acids and derivatives	–	up	up
Zazp002547	cyclo-(Gly-Phe)	Amino acids and derivatives	down	–	up
MWStz091	Cyclo(L-Ala-L-Pro)	Amino acids and derivatives	–	up	–
MWStz170	Cyclo(L-Leu-trans-4-hydroxy-L-Pro)	Amino acids and derivatives	–	–	up
MWStz211	Cyclo(L-Phe-trans-4-hydroxy-L-Pro)	Amino acids and derivatives	–	–	up
Lmrj002793	Cyclo(Phe-Glu)	Amino acids and derivatives	down	up	–
Lmhp002764	Cyclo(Pro-Leu)*	Amino acids and derivatives	–	up	up
MWStz255	Cyclo(Pro-Phe)*	Amino acids and derivatives	–	up	–
Lmrj002244	Cyclo(Pro-Pro)	Amino acids and derivatives	–	up	–
Lmrj001341	Cyclo(Ser-Pro)	Amino acids and derivatives	down	up	up
ML10181668	Cycloleucine	Amino acids and derivatives	–	–	–
MWS0611	D-Alanyl-D-Alanine*	Amino acids and derivatives	down	–	–
MWS04555g	D-Allo-Isoleucine*	Amino acids and derivatives	down	–	up
MWS1926	DL-Leucine*	Amino acids and derivatives	down	–	up
mws0224	DL-Methionine	Amino acids and derivatives	down	–	up
MWSmce056	DL-Tryptophan*	Amino acids and derivatives	down	–	up
mws4532g	D-Ornithine	Amino acids and derivatives	–	–	–
MWS201465	Glu-Phe	Amino acids and derivatives	down	–	up
MWS20672	Glu-Phe-Ala	Amino acids and derivatives	down	–	up
MWS4296	Glycylphenylalanine*	Amino acids and derivatives	down	–	up
MWS4309	Glycyl-tryptophan	Amino acids and derivatives	–	up	up
MWS20645	Gly-Tyr*	Amino acids and derivatives	down	–	up
Zbqp002414	h-gamma-Glu-leu-oh	Amino acids and derivatives	down	–	–
MWS201405	His-Tyr	Amino acids and derivatives	down	–	up
MWS04447	Homoproline	Amino acids and derivatives	up	up	up
MWS201398	Hyp-Ser	Amino acids and derivatives	–	–	up
MWS201397	Hyp-Val	Amino acids and derivatives	–	down	down
MWS201424	Ile-Asn	Amino acids and derivatives	–	–	up
pme2074	Jasmonoyl-L-Isoleucine	Amino acids and derivatives	up	up	up
pme0128	L-Alanyl-L-Alanine*	Amino acids and derivatives	down	–	–
mws4176	L-Alanyl-L-Phenylalanine	Amino acids and derivatives	down	–	up
pme2679	L-Allo-isoleucine	Amino acids and derivatives	down	–	up
mws0260	L-Arginine	Amino acids and derivatives	down	–	up
mws0001	L-Asparagine	Amino acids and derivatives	–	–	up
mws0219	L-Aspartic Acid*	Amino acids and derivatives	down	up	down
mws0629	L-Aspartyl-L-Phenylalanine	Amino acids and derivatives	down	–	up
pme0116	L-Carnosine	Amino acids and derivatives	down	–	–
pme0008	L-Citrulline	Amino acids and derivatives	–	–	–
Hmsp000364	L-Cyclopentylglycine	Amino acids and derivatives	up	up	–
mws0875	L-Cysteinyl-L-glycine	Amino acids and derivatives	–	up	–
Lmbp001216	L-Dihomomethionine	Amino acids and derivatives	down	–	–
MWS201461	Leu-Arg	Amino acids and derivatives	–	–	up
MWS201437	Leu-Asp	Amino acids and derivatives	down	up	up
pme0014	L-Glutamic acid	Amino acids and derivatives	down	–	–
pme0193	l-Glutamine	Amino acids and derivatives	down	–	down
mws5041	L-Glycyl-L-isoleucine*	Amino acids and derivatives	down	up	up
mws5042	L-Glycyl-*L*-phenylalanine*	Amino acids and derivatives	down	–	up
pme0124	L-Glycyl-L-proline	Amino acids and derivatives	down	up	up
mws0254	L-Histidine	Amino acids and derivatives	down	up	up
pme0057	L-Homocysteine	Amino acids and derivatives	–	–	–
pme2890	L-Homocystine	Amino acids and derivatives	down	–	up
Lmbp000123	L-Homomethionine	Amino acids and derivatives	down	–	–
MWS00123g	*L*-Homophenylalanine	Amino acids and derivatives	up	–	–
mws0671	L-Homoserine*	Amino acids and derivatives	–	–	–
mws0258	L-Isoleucine*	Amino acids and derivatives	down	–	up
Lmrj002087	L-Isoleucyl-l-Aspartate	Amino acids and derivatives	down	–	up
MWS00327g	L-Isoserine	Amino acids and derivatives	–	down	down
mws0227	L-Leucine*	Amino acids and derivatives	down	–	up
Lmhp002031	L-Leucyl-L-Leucine	Amino acids and derivatives	down	–	up
mws5035	L-Leucyl-*L*-phenylalanine	Amino acids and derivatives	down	–	up
pme0026	l-Lysine	Amino acids and derivatives	down	–	down
pmb0962	l-Lysine-Butanoic Acid	Amino acids and derivatives	up	up	up
pme1210	L-Methionine	Amino acids and derivatives	down	–	up
pme1419	L-Methionine methyl ester	Amino acids and derivatives	down	down	down
pme2617	L-Methionine Sulfoxide	Amino acids and derivatives	–	–	up
mws1587	L-Norleucine*	Amino acids and derivatives	down	–	up
pme0021	L-Phenylalanine	Amino acids and derivatives	down	–	up
mws0636	L-Phenylalanyl-*L*-phenylalanine	Amino acids and derivatives	down	–	up
pme0006	L-Proline*	Amino acids and derivatives	–	–	up
Lmhp001461	L-Prolyl-L-Leucine	Amino acids and derivatives	down	–	up
Lmhp001732	L-Prolyl-L-Phenylalanine	Amino acids and derivatives	–	–	up
Zmzn000113	L-threo-3-Methylaspartate	Amino acids and derivatives	down	–	–
mws0230	L-Threonine*	Amino acids and derivatives	–	–	–
mws0282	L-Tryptophan	Amino acids and derivatives	down	up	up
mws0250	L-Tyrosine*	Amino acids and derivatives	down	down	–
MWS1771	L-Tyrosine methyl ester	Amino acids and derivatives	down	–	–
mws0256	L-Valine	Amino acids and derivatives	down	–	up
Wcdp000380	L-Valinol	Amino acids and derivatives	–	down	down
Lmhp001670	L-Valyl-L-Leucine	Amino acids and derivatives	down	–	up
Lmhp002001	L-Valyl-L-Phenylalanine	Amino acids and derivatives	down	–	up
MWS201412	Lys-Asp*	Amino acids and derivatives	–	up	up
MWS201471	Lys-Tyr	Amino acids and derivatives	–	up	up
pmb0034	L-α-Glutamyl-L-Glutamic Acid	Amino acids and derivatives	down	down	–
Zmdp002216	L-γ-Glutamyl-L-leucine	Amino acids and derivatives	down	down	–
MWS201470	Met-Arg	Amino acids and derivatives	up	up	up
MWS201399	Met-Asn	Amino acids and derivatives	down	–	up
MWSmce585	Methyl 3-aminopropanoate	Amino acids and derivatives	–	down	down
MWS201421	Met-Phe	Amino acids and derivatives	down	down	up
MWS201429	Met-Ser	Amino acids and derivatives	–	–	up
MWS201430	Met-Thr	Amino acids and derivatives	–	–	up
Wayn001257	N-(1-Deoxy-1-fructosyl)Leucine	Amino acids and derivatives	–	up	up
Wayp001024	N-(1-Deoxy-1-fructosyl)Valine	Amino acids and derivatives	up	up	up
MWS04412	N(6),N(6)-Dimethyl-l-lysine	Amino acids and derivatives	down	–	up
Zbqn004590	N-(acetyl)phenylalanine	Amino acids and derivatives	–	–	–
MWS5164	*N*,*N*′-Dimethylarginine;SDMA*	Amino acids and derivatives	down	–	up
pme3033	*N*,*N*-Dimethylglycine*	Amino acids and derivatives	–	down	down
MWS20987	N5-(1-Iminoethyl)-L-ornithine	Amino acids and derivatives	–	–	up
pme0122	N6-Acetyl-l-lysine	Amino acids and derivatives	down	–	–
MWSslk120	N-Acetyl-DL-phenylalanine	Amino acids and derivatives	up	–	up
pme0170	N-Acetyl-L-Arginine	Amino acids and derivatives	–	–	up
pme0137	N-Acetyl-l-Glutamine	Amino acids and derivatives	up	–	–
pme0253	N-Acetyl-L-leucine	Amino acids and derivatives	up	–	up
MWSslk140	N-Acetyl-L-Methionine	Amino acids and derivatives	–	–	up
pmb2591	N-Acetyl-L-Tryptophan	Amino acids and derivatives	–	down	down
mws0520	N-Acetyl-L-tyrosine	Amino acids and derivatives	up	–	–
Zbqn003921	N-carboxy-N-(2-oxo-2-phenylethyl)-L-alanine	Amino acids and derivatives	up	–	–
MWS00204g	N-Ethylglycine*	Amino acids and derivatives	–	down	down
NK10251888	NG,NG-Dimethyl-L-arginine*	Amino acids and derivatives	down	–	up
mws0736	N-Glycyl-L-leucine*	Amino acids and derivatives	down	up	up
Lmqp000427	N-Methyl-Trans-4-Hydroxy-L-Proline	Amino acids and derivatives	down	–	–
MWS5209	N-Methyl-α-aminoisobutyric acid	Amino acids and derivatives	–	up	–
Zmjp000182	*N*-Monomethyl-L-arginine*	Amino acids and derivatives	down	up	–
Zmyn000155	N-α-Acetyl-L-ornithine	Amino acids and derivatives	down	–	up
MWS201054	O-Acetyl-L-homoserine	Amino acids and derivatives	–	–	–
mws1050	O-Acetylserine	Amino acids and derivatives	down	down	down
MWS20633g	O-phosphate-L-tyrosine	Amino acids and derivatives	–	up	up
mws3133	Oxaceprol	Amino acids and derivatives	–	–	–
mws4134	Oxiglutatione	Amino acids and derivatives	down	–	–
MWS201458	Phe-Ile	Amino acids and derivatives	down	down	up
MWS201478	Phe-Ser	Amino acids and derivatives	down	–	up
MWS201442	Phe-Thr	Amino acids and derivatives	down	–	up
MWS201415	Pro-Asp	Amino acids and derivatives	down	–	–
mws4516	Pyroglutamic acid	Amino acids and derivatives	–	–	–
pme1286	S-(5′-Adenosy)-L-homocysteine	Amino acids and derivatives	down	–	up
MWStz103	S-(5′-Adenosyl)-L-methionine	Amino acids and derivatives	up	–	–
MWS201381	Ser-Trp	Amino acids and derivatives	down	up	up
Lcsp002417	Ser-Val-Leu	Amino acids and derivatives	down	–	up
Zmdp000972	S-Methyl-L-cysteine	Amino acids and derivatives	down	down	–
MWS20966	*S*-methyl-L-thiocitrulline	Amino acids and derivatives	–	–	–
MWS201477	Thr-Thr	Amino acids and derivatives	–	–	–
mws0216	Trans-4-Hydroxy-L-proline*	Amino acids and derivatives	down	–	up
pmp001257	Tridecanoylglycine	Amino acids and derivatives	down	–	down
Zmzp000145	Trimethyllysine	Amino acids and derivatives	down	up	–
MWS201187	Trp-His	Amino acids and derivatives	up	–	–
MWS201480	Tyr-Ala	Amino acids and derivatives	–	–	up
MWS201479	Tyr-Gly*	Amino acids and derivatives	down	–	up
MWS201400	Val-Trp	Amino acids and derivatives	down	–	up
Zbqp003189	Val-Val	Amino acids and derivatives	up	up	up
Zmdp001647	γ-Glutamyl-L-valine	Amino acids and derivatives	down	–	–
Zmdp001663	γ-glutamylmethionine	Amino acids and derivatives	–	–	–
Zmdp001857	γ-Glutamyltyrosine	Amino acids and derivatives	down	–	–
Zmmp003443	γ-Glu-Trp	Amino acids and derivatives	down	–	up
Zmdp001718	γ-L-Glutamyl-S-allyl-L-cysteine	Amino acids and derivatives	down	–	–
Zmdp000446	γ-L-Glutamyl-*S*-methyl-L-cysteine	Amino acids and derivatives	up	–	–
Zbfn002690	1-(4-Hydroxybenzoyl)Glucose; 25,545–07-7	Phenolic acids	up	–	–
Lmfn001337	1,2,3-Tri-O-galloyl-β-d-glucose*	Phenolic acids	–	up	–
Zmdn003203	1,2,6-Tri-O-galloyl-β-d-glucose*	Phenolic acids	–	up	–
Lmfn001209	1,3,6-Tri-O-galloyl-β-d-glucose*	Phenolic acids	–	up	–
mws1584	1,3-O-Dicaffeoylquinic Acid (Cynarin)	Phenolic acids	–	–	–
Lmsn003088	1,4,6-Tri-O-galloyl-β-d-glucose*	Phenolic acids	–	up	–
Wmhn001495	1,4-Di-O-Galloyl-d-glucose	Phenolic acids	up	up	–
Waxn004217	1,5-O-dicaffeoyl-3-O-glucoside-quinic acid	Phenolic acids	–	up	–
mws0748	1-Caffeoylquinic acid	Phenolic acids	down	–	–
pmp000086	1-Feruloyl-sn-glycerol	Phenolic acids	up	–	–
Wasn002933	1-O-(3,4,5-Trimethoxybenzoyl)-B-*D*-Glucopyranoside	Phenolic acids	down	–	–
Lmtn000940	1-O-(3,4-Dihydroxy-5-methoxy-benzoyl)-glucoside	Phenolic acids	down	–	–
Lmsn002288	1-O-Caffeoyl-(6-O-glucosyl)-β-d-glucose	Phenolic acids	down	up	–
Lmsn004690	1-O-Caffeoyl-3-O-galloyl-4,6-(*S*)-HHDP-β-d-glucose	Phenolic acids	–	–	–
Hmbp003234	1-O-Caffeoylglycerol	Phenolic acids	–	–	down
Zmhn002422	1-O-Feruloyl-β-d-glucose	Phenolic acids	down	–	–
Lmsn004323	1-O-Galloyl-2-O-Feruloyl-β-d-glucose	Phenolic acids	down	–	–
Lmsn001014	1-O-Galloyl-β-d-glucose*	Phenolic acids	down	–	–
pmb2871	1-O-Gentisoyl-β-D-glucoside*	Phenolic acids	down	–	–
pmb3068	1-O-p-Coumaroylquinic acid	Phenolic acids	up	–	–
pmn001320	1-O-p-Cumaroylglycerol	Phenolic acids	up	–	–
Lmsn002247	1-O-Salicyloyl-β-d-glucose*	Phenolic acids	down	up	–
HJN003	1-O-Sinapoyl-β-d-glucose	Phenolic acids	up	–	–
Lmtn002565	1-O-Vanilloyl-d-Glucose	Phenolic acids	down	up	–
Zbln003341	2-(2-(3,4-dihydroxyphenyl)-2-methoxyethoxy)-6-(hydroxymethyl)tetrahydro-2H-pyran-3,4,5-tri	Phenolic acids	–	up	–
Jmwn002117	2-(3,4-dihydroxyphenyl)ethanediol 1-O-β-D-glucopyranoside*	Phenolic acids	down	–	–
pme3083	2-(Formylamino)benzoic acid	Phenolic acids	up	–	–
MWSslk138	2,3,4-Trihydroxybenzoic acid	Phenolic acids	up	–	–
Lafp002342	2,3-Dihydroxy-1-(4-hydroxy-3,5-dimethoxyphenyl)propan-1-one	Phenolic acids	up	–	–
mws0639	2,3-Dihydroxybenzoic Acid*	Phenolic acids	up	–	–
MWSmce490	2′,4′,6′-Trihydroxyacetophenone	Phenolic acids	down	down	down
mws0885	2,4-Dihydroxybenzoic acid	Phenolic acids	up	–	–
mws0180	2,5-Dihydroxybenzoic acid; Gentisic Acid*	Phenolic acids	up	–	–
Lmdp002901	2,6-Dihydroxy-4-isopropylphenyl-1-O-β-D-glucoside	Phenolic acids	down	–	–
Wmyn000217	2,6-dimethoxy-hydroquinone-4-O-β-D-glucopyranoside	Phenolic acids	down	–	–
NK10253223	2-Amino-3-methoxybenzoic acid	Phenolic acids	–	down	–
pmp000087	2-Feruloyl-sn-glycerol	Phenolic acids	up	–	–
Lmgp002452	2-Glucosyloxy-(4-hydroxyphenyl)acetic acid (Dhurrin acid)	Phenolic acids	up	down	up
Lmbn001930	2-Hydroxybenzaldehyde (Salicylaldehyde)	Phenolic acids	up	–	–
Lmmn001643	2-Hydroxycinnamic acid*	Phenolic acids	up	–	up
Zmyn002919	2-Methylbenzoic acid	Phenolic acids	up	up	up
Zmzn002575	2-Nitrophenol	Phenolic acids	up	–	–
Lmhn102068	2-O-Caffeoylmalic acid	Phenolic acids	down	–	up
Hmln000873	2-O-Galloyl-d-glucose	Phenolic acids	down	–	–
Wacn003131	2-O-P-Coumaroylhydroxycitric Acid	Phenolic acids	down	–	–
Lakn001671	2-β-D-Glucopyranosyloxy-5-hydroxy-phenylacetic acid	Phenolic acids	–	–	–
MWS3046	3-(3-Hydroxyphenyl)-3-hydroxypropanoic acid	Phenolic acids	up	down	down
mws0346	3-(3-Hydroxyphenyl)-propionic acid	Phenolic acids	up	down	down
MWSmce713	3-(4-Hydroxyphenyl)-1-propanol	Phenolic acids	–	–	–
HJN102	3,4,5-Tricaffeoylquinic acid	Phenolic acids	–	up	up
pmn001517	3,4,5-Trimethoxyphenyl-1-O-Glucoside	Phenolic acids	down	–	–
pme2598	3,4-Dihydroxybenzeneacetic acid*	Phenolic acids	–	down	–
mws0183	3,4-Dihydroxybenzoic acid (Protocatechuic acid)*	Phenolic acids	up	–	–
mws0612	3,4-Dimethoxyphenyl acetic acid	Phenolic acids	up	down	–
Wmzn002116	3,5-Dicaffeoylquinic acid	Phenolic acids	–	–	–
pmn001525	3,5-Digalloylshikimic acid	Phenolic acids	up	up	–
HJN037	3,5-Dihydroxy-4-methoxybenzoic acid; 4-O-Methylgallic Acid	Phenolic acids	up	–	–
MWSmce454	3,5-Dihydroxyacetophenone	Phenolic acids	up	–	–
MWS1944	3,5-Dihydroxytoluene	Phenolic acids	up	up	–
mws0444	3-Aminosalicylic acid	Phenolic acids	–	down	–
MWSHC20102	3-Hydroxy-1-(4-Hydroxy-3-Methoxyphenyl)Propan-1-One	Phenolic acids	–	–	–
pmn001690	3-Hydroxy-4-isopropylbenzylalcohol-3-O-glucoside	Phenolic acids	down	–	up
MWSslk066	3-Hydroxy-4-methoxybenzoic acid; Isovanillic Acid	Phenolic acids	–	down	down
pmn001511	3-Hydroxy-5-Methylphenol-1-O-Glucoside	Phenolic acids	up	–	–
Lmgp002593	3-hydroxybenzaldehyde	Phenolic acids	up	–	–
Lmyn004439	3-Hydroxyl-5-methylphenol-1-O-β-D-(6′-galloyl)glucoside*	Phenolic acids	up	–	–
MWS4301	3-hydroxyphenylacetic acid*	Phenolic acids	up	down	–
MWS3149	3-Methoxybenzoic acid	Phenolic acids	up	up	up
Zmgn001989	3-Methylcatechol	Phenolic acids	up	–	–
MWS2058	3-Methylsalicylic Acid	Phenolic acids	up	up	up
Zaln004082	3-O-caffeoylshikimic acid*	Phenolic acids	down	–	–
pmb0752	3-O-Feruloylquinic acid	Phenolic acids	–	–	up
pmb2833	3-O-Feruloylquinic acid-O-glucoside*	Phenolic acids	down	–	–
Hmln000659	3-O-Galloyl-d-glucose*	Phenolic acids	down	–	–
MWSmce387	3-O-Methylgallic acid	Phenolic acids	up	–	–
pmb3064	3-O-p-Coumaroylquinic acid-O-glucoside	Phenolic acids	down	–	–
pmb3075	3-O-p-Coumaroylshikimic acid	Phenolic acids	up	up	–
Lmtn003598	3-Prenyl-4-O-glucosyloxy-4-hydroxybenzoic acid	Phenolic acids	–	up	–
Lmyn003835	4-(3,4,5-Trihydroxybenzoxy)benzoic acid	Phenolic acids	down	–	–
mws1336	4-Aminobenzoic acid	Phenolic acids	up	down	–
Zaln004057	4-caffeoylshikimic acid*	Phenolic acids	down	–	–
mws0749	4-Hydroxybenzoic acid	Phenolic acids	up	down	down
HJN025	4-Hydroxybenzyl Alcohol	Phenolic acids	–	up	–
MWSmce370	4-Hydroxyphenylacetic acid*	Phenolic acids	up	down	down
MWS5206	4-Hydroxyphenyllactic Acid*	Phenolic acids	up	down	down
pmb2795	4-Methoxycinnamic acid	Phenolic acids	up	–	–
Lmbn004847	4-Methoxyphenylpropionic acid	Phenolic acids	up	–	–
mws0566	4-Methylcatechol	Phenolic acids	–	up	–
Zmyn002321	4-Methylphenol	Phenolic acids	up	–	down
Wafn002874	4-O-(4’-O-alpha-d-Glucopyranosyl)caffeoylquinic acid	Phenolic acids	–	–	–
Zmhn000892	4-O-Glucosyl-3,4-dihydroxybenzyl alcohol	Phenolic acids	down	–	–
Zmhn001358	4-O-Glucosyl-4-hydroxybenzoic acid*	Phenolic acids	down	–	–
Zmhn002227	4-O-Glucosyl-sinapate	Phenolic acids	–	up	–
pma0110	4-O-Sinapoylquinic acid	Phenolic acids	–	down	–
Hmhn003518	4-O-β-D-glucopyranosylferulic acid	Phenolic acids	down	–	–
Hmtn001120	5-(2-Hydroxyethyl)-2-O-glucosylphenol	Phenolic acids	down	–	–
pmn001516	5-Galloylshikimic acid	Phenolic acids	up	–	–
Lmmn003663	5-Glucosyloxy-2-Hydroxybenzoic acid methyl ester	Phenolic acids	down	up	–
Lmzn001582	5′-Glucosyloxyjasmanic acid	Phenolic acids	up	–	–
MWSHC20125	5-O-Caffeoylshikimic acid	Phenolic acids	down	–	–
pmb2554	5-O-Feruloyl quinic acid glucoside*	Phenolic acids	down	–	–
Zmhn001703	5-O-Galloyl-D-hamamelose*	Phenolic acids	down	–	–
Ymjm000116	6-O-Acetylarbutin	Phenolic acids	–	–	up
Zmhn001793	6-O-Caffeoyl-d-glucose*	Phenolic acids	up	–	–
Hmbn002692	6’-O-Feruloyl-D-sucrose	Phenolic acids	down	–	–
Zmhn002334	6-O-Feruloyl-β-d-glucose	Phenolic acids	down	–	–
Lmfn000604	6-O-Galloyl-β-d-glucose*	Phenolic acids	down	–	–
Zmxn003874	7,8-Dihydro-Buddlenol B	Phenolic acids	up	down	–
Lmqp002761	Alnusonol	Phenolic acids	down	down	down
Lmtn002233	Androsin	Phenolic acids	down	–	–
pmb2654	Anthranilate-1-O-Sophoroside	Phenolic acids	down	–	–
MWSmce675	Arbutin	Phenolic acids	down	–	–
Labn003910	Benzyl B-Primeveroside*	Phenolic acids	–	–	–
mws1297	Benzyl glucoside	Phenolic acids	up	–	–
Lmtn002324	Benzyl-(2”-O-glucosyl)glucoside*	Phenolic acids	–	–	up
Cmjn004337	Benzyl-(2”-O-xylosyl)glucoside*	Phenolic acids	–	–	–
Lmyn003028	Benzyl-β-gentiobioside*	Phenolic acids	–	up	up
mws2120	Brevifolin carboxylic acid	Phenolic acids	down	–	–
mws2212	Caffeic acid	Phenolic acids	–	up	up
Zbfn002396	Caffeoyl-O-mannitol	Phenolic acids	down	–	–
Labn003679	Caffeyl alcohol 4-O-β-D-glucopyranoside	Phenolic acids	up	–	–
MWS0550	Cinnamaldehyde	Phenolic acids	down	down	down
mws2213	Cinnamic acid	Phenolic acids	up	–	up
mws0906	Coniferin	Phenolic acids	up	–	–
mws0093	Coniferyl alcohol	Phenolic acids	up	–	–
Hmsn002272	Demethyl coniferin	Phenolic acids	–	up	up
Lmgp003989	Dicaffeoylshikimic acid	Phenolic acids	up	up	–
pmn001513	Digallic Acid	Phenolic acids	–	up	–
Lmmn000774	Dihydrocaffeoylglucose*	Phenolic acids	–	–	–
Lazn001573	Erythro-Guaiacylglycerol	Phenolic acids	up	down	–
mws2184	Ethyl caffeate	Phenolic acids	up	up	up
MWSslk006	Ethyl Vanillate	Phenolic acids	up	–	–
mws0014	Ferulic acid*	Phenolic acids	up	down	–
Hmmn002544	Ferulic acid-4-O-glucoside	Phenolic acids	down	–	–
mws0024	Gallic acid	Phenolic acids	–	up	–
pmb2928	Gallic acid-4-O-glucoside	Phenolic acids	down	–	–
Hmtn001302	Glucosyloxybenzoic acid	Phenolic acids	–	up	up
MWSHC2022	Glucosyringic acid	Phenolic acids	–	–	up
Cmyp005063	Grandidentatin	Phenolic acids	–	down	up
pme1292	Homogentisic acid*	Phenolic acids	–	–	–
WaYn002313	Homosyringic Acid 4’-O-Glucoside	Phenolic acids	down	–	–
mws0117	Homovanillic acid; 4-Hydroxy-3-methoxyphenylacetic acid	Phenolic acids	up	–	–
pmb3056	Homovanilloylquinic acid	Phenolic acids	down	–	–
Lmyn002435	Hydrangeifolin I	Phenolic acids	–	up	up
MA10014775	Hydroquinone	Phenolic acids	down	–	up
Wafn003070	Hydroxyferulic acid glucoside	Phenolic acids	down	up	up
MWSmce264	Hydroxytyrosol	Phenolic acids	down	–	–
pme0422	Isoferulic Acid*	Phenolic acids	up	down	–
Lmmn001294	Koaburaside*	Phenolic acids	–	–	–
Li512113	Maleoyl-caffeoylquinic acid	Phenolic acids	up	–	–
MWS1776	m-Cresol	Phenolic acids	up	down	–
Hmtn001288	Methyl 2,4-dihydroxyphenylacetate*	Phenolic acids	–	down	down
Zmgn004894	Methyl 4-hydroxybenzoate*	Phenolic acids	up	up	up
Hmdn001963	Methyl Brevifolincarboxylate	Phenolic acids	up	up	up
Lmdn003756	Methyl caffeate	Phenolic acids	–	–	–
MWSHC20168	Methyl cumalate*	Phenolic acids	up	–	–
MWSmce230	Methyl gallate	Phenolic acids	up	up	–
Wbsp004755	Methyl Hydroxycinnamate	Phenolic acids	up	–	–
Lmmn002179	Methylsalicylate-2-O-glucoside	Phenolic acids	down	–	–
mws0145	O-Anisic acid (2-Methoxybenzoic acid)*	Phenolic acids	up	up	up
pme1439	p-Coumaric acid*	Phenolic acids	up	–	–
MWSslk144	p-Coumaric acid ethyl ester	Phenolic acids	up	down	down
Lmhn002926	p-Coumaroylmalic acid	Phenolic acids	up	up	–
pmb3055	p-Coumaroylquinic acid-4’-O-glucuronide	Phenolic acids	down	–	–
MWS1846	Phenoxyacetic acid*	Phenolic acids	up	–	–
MWS1848	Phenyl acetate	Phenolic acids	–	–	–
NK10264324	Phloroglucinol; 1,3,5-Benzenetriol	Phenolic acids	down	up	–
Hmhn000927	Phloroglucinol-1-O-β-D-glucopyranoside	Phenolic acids	down	up	–
pme0282	Phthalic acid	Phenolic acids	up	–	–
mws1506	Plantamajoside	Phenolic acids	down	–	–
Wasn004683	Protocatechuic acid 1-O-(Glucosylvanilloyl)	Phenolic acids	down	–	–
MWSmce501	Protocatechuic Acid Methyl Ester	Phenolic acids	up	up	–
pmn001367	Protocatechuic acid-4-O-glucoside*	Phenolic acids	down	–	–
pmb3317	Quinacyl syringic acid	Phenolic acids	down	–	–
pmn001710	Rosmarinic acid-3’-O-glucoside	Phenolic acids	down	–	–
mws1521	Salicin	Phenolic acids	up	–	–
MWSmce274	Salicyl Alcohol	Phenolic acids	up	down	–
Lmgn001670	Salicylic acid	Phenolic acids	up	up	up
pmb3142	Salicylic acid-2-O-glucoside	Phenolic acids	down	–	–
mws2367	Salidroside	Phenolic acids	down	–	–
Lmbp002309	Sinapaldehyde-4-O-Glucoside	Phenolic acids	down	–	–
mws4085	Sinapic acid	Phenolic acids	up	–	–
pme3443	Sinapinaldehyde	Phenolic acids	up	up	up
pma0149	Sinapoyl malate	Phenolic acids	down	–	–
Lmgn004359	SinapoylcaffeoylQuinic acid O-glucose	Phenolic acids	down	–	–
Zmln000899	Syringaldehyde-4-O-glucoside	Phenolic acids	–	–	–
mws0027	Syringic acid	Phenolic acids	up	–	–
pmb0824	Syringic acid-4-O-(6″-feruloyl)glucoside	Phenolic acids	down	–	–
mws0011	Syringin	Phenolic acids	down	–	–
Hmqn000843	Tachioside	Phenolic acids	–	up	–
pme0281	Terephthalic acid	Phenolic acids	up	–	–
pmn001523	Trigallic acid	Phenolic acids	down	up	–
mws2368	Tyrosol; 4-Hydroxyphenylethanol	Phenolic acids	up	up	up
MWSslk092	Usnic acid	Phenolic acids	up	up	–
mws0028	Vanillic acid	Phenolic acids	up	down	down
Lmqp002627	Vanillic acid methyl ester	Phenolic acids	–	–	–
Zmhn001883	Vanillic acid-4-O-glucoside	Phenolic acids	–	–	–
Jmwn002620	Vanillic Acid-4-O-Glucuronide	Phenolic acids	up	–	–
MWSmce632	Vanillin acetate	Phenolic acids	up	–	–
Zmtn001661	Vanilloloside	Phenolic acids	down	–	–
Lmhn002051	Vnilloylmalic acid	Phenolic acids	up	–	up
Lmbn002648	α-Hydroxycinnamic Acid*	Phenolic acids	up	down	down
Zbln001290	β-Hydroxy-(3,4-dihydroxyphenylethanolyl)-glucoside	Phenolic acids	down	–	–
Hmln002996	β-Hydroxyacteoside	Phenolic acids	down	–	–
Hmgn002038	β-Oxoacteoside	Phenolic acids	–	–	–
mws0663	1,7-Dimethylxanthine	Nucleotides and derivatives	up	up	–
MWSmce295	1-beta-D-Arabinofuranosyluracil	Nucleotides and derivatives	up	–	up
mws0847	1-Methyladenine	Nucleotides and derivatives	down	–	–
pme3967	2-(Dimethylamino)guanosine*	Nucleotides and derivatives	–	up	up
pmb0374	2-Aminopurine	Nucleotides and derivatives	down	–	up
pme3961	2’-Deoxyadenosine*	Nucleotides and derivatives	down	up	up
pme3184	2’-Deoxyadenosine-5′-monophosphate	Nucleotides and derivatives	down	–	–
pme1194	2’-Deoxycytidine	Nucleotides and derivatives	–	up	up
pme1184	2’-Deoxyguanosine	Nucleotides and derivatives	–	–	up
pmc0066	2’-Deoxyinosine-5′-monophosphate	Nucleotides and derivatives	up	–	–
pmb2507	2-Deoxyribose-1-phosphate	Nucleotides and derivatives	–	down	–
mws0863	2-Deoxyribose-5′-phosphate	Nucleotides and derivatives	–	–	–
Hmhp001812	2’-O-Methyladenosine	Nucleotides and derivatives	up	up	up
mws0874	3′-Adenylic Acid	Nucleotides and derivatives	down	up	up
pme1266	3-Methylxanthine	Nucleotides and derivatives	–	–	–
pme0152	5,6-Dihydro-5-methyluracil	Nucleotides and derivatives	down	–	–
pme0151	5,6-Dihydrouracil	Nucleotides and derivatives	–	–	up
Lmqp000329	5-Aminoimidazole ribonucleotide	Nucleotides and derivatives	down	–	–
pme1474	5’-Deoxy-5′-(methylthio)adenosine	Nucleotides and derivatives	up	up	up
MWSmce116	5’-Deoxyadenosine*	Nucleotides and derivatives	–	–	up
MWS2910	5-Methyl-2’-Deoxycytidine	Nucleotides and derivatives	down	–	up
mws0572	5-Methylcytosine	Nucleotides and derivatives	–	up	up
MWSslk102	6-Chloropurine	Nucleotides and derivatives	–	–	–
MWS4525	6-*O*-methylguanine	Nucleotides and derivatives	–	up	up
pme3968	7-Methylguanine	Nucleotides and derivatives	–	up	–
pme2801	7-Methylxanthine	Nucleotides and derivatives	–	–	up
MWS2984	8-Azaguanine	Nucleotides and derivatives	up	up	up
mws0724	8-Hydroxyguanosine	Nucleotides and derivatives	–	–	–
mws1060	9-(Arabinosyl)hypoxanthine	Nucleotides and derivatives	up	–	up
Wcjp001256	9-Alpha-Ribofuranosyladenine*	Nucleotides and derivatives	down	–	up
Zmsp001773	9-Arabinosyladenine*	Nucleotides and derivatives	down	–	up
MWSHC2054	Adenine	Nucleotides and derivatives	up	–	up
pme0230	Adenosine*	Nucleotides and derivatives	down	–	up
Zmjn001030	Adenosine 2’-Phosphate	Nucleotides and derivatives	–	–	up
pme2117	Adenosine 5′-diphosphate	Nucleotides and derivatives	down	–	down
pmb0981	Adenosine 5′-monophosphate	Nucleotides and derivatives	down	–	down
pme1173	Allopurinol	Nucleotides and derivatives	up	–	up
mws1715	Cordycepin (3’-Deoxyadenosine)*	Nucleotides and derivatives	down	up	up
MWSmce333	Crotonoside; 2-Hydroxyadenosine	Nucleotides and derivatives	–	–	up
mws0884	Cyclic 3′,5′-Adenylic acid	Nucleotides and derivatives	up	up	up
ML10180524	Cytarabine	Nucleotides and derivatives	up	up	up
pme3732	Cytidine	Nucleotides and derivatives	up	up	up
MWSslk257	Cytidine 5′-monophosphate(Cytidylic acid)	Nucleotides and derivatives	–	–	up
mws0255	Cytosine	Nucleotides and derivatives	up	up	up
MWS5083	Flavin Single Nucleotide(FMN)	Nucleotides and derivatives	–	–	down
pme1109	Guanine	Nucleotides and derivatives	up	up	up
pme1178	Guanosine	Nucleotides and derivatives	–	up	up
mws0609	Guanosine 3′,5′-cyclic monophosphate	Nucleotides and derivatives	up	up	up
pmb0998	Guanosine 5′-monophosphate	Nucleotides and derivatives	down	–	–
pme0033	Hypoxanthine	Nucleotides and derivatives	up	–	up
pmb0532	Inosine 5′-monophosphate	Nucleotides and derivatives	down	–	down
MWS5173	Isocytosine	Nucleotides and derivatives	–	–	up
pme0183	Isoguanine	Nucleotides and derivatives	up	up	up
Lmcp002302	N6-(2-Hydroxyethyl)adenosine*	Nucleotides and derivatives	–	up	up
pme2060	N6-Isopentenyladenine	Nucleotides and derivatives	up	–	–
MWS4354	N6-methyladenosine	Nucleotides and derivatives	up	up	up
pmb0197	N7-Methylguanosine	Nucleotides and derivatives	down	–	up
pmb0530	Nicotinic acid adenine dinucleotide	Nucleotides and derivatives	down	–	–
pme2746	Riboflavin 5′-Adenosine Diphosphate	Nucleotides and derivatives	–	up	–
pmf0289	Riboprine	Nucleotides and derivatives	up	–	up
pme3337	Succinyladenosine	Nucleotides and derivatives	–	–	up
mws0248	Uridine	Nucleotides and derivatives	up	–	up
pmb2922	Uridine 5′-diphospho-d-glucose	Nucleotides and derivatives	–	up	–
pme3188	Uridine 5′-monophosphate	Nucleotides and derivatives	–	up	up
Zmfn000481	Uridine-5’-Diphosphate-D-Xylose	Nucleotides and derivatives	–	up	–
Zmjp000966	Vidarabine	Nucleotides and derivatives	down	–	up
pme0256	Xanthine	Nucleotides and derivatives	up	–	–
mws0668	Xanthosine	Nucleotides and derivatives	up	–	up
mws0675	β-Nicotinamide mononucleotide	Nucleotides and derivatives	–	down	–
Lmsp004137	3,4,2′,4′,6’-Pentahydroxychalcone	Flavonoids	down	–	–
pmn001716	Carthamone*	Flavonoids	down	–	–
HJN055	Dihydrocharcone-4’-O-glucoside*	Flavonoids	down	up	–
Labn004865	Hydroxy isoliquiritigenin glucoside*	Flavonoids	down	–	–
Lmlp006175	Isosalipurposide (Phlorizin Chalcone)	Flavonoids	–	–	–
pme2960	Naringenin chalcone; 2′,4,4′,6’-Tetrahydroxychalcone*	Flavonoids	–	down	–
Cmxp005429	Okanin	Flavonoids	–	down	–
zjgp122004	Okanin-3’-O-β-D-glucoside*	Flavonoids	down	–	–
Cmxp003975	Okanin-4’-O-glucoside(Marein)*	Flavonoids	down	–	–
pme1201	Phloretin	Flavonoids	down	down	down
mws2118	Phloretin-2’-O-glucoside (Phlorizin)	Flavonoids	down	up	–
Hmmn003343	Sappanchalcone	Flavonoids	–	–	–
Hmpn005101	Sieboldin	Flavonoids	down	–	–
Zbbp005255	Aureusidin	Flavonoids	–	down	–
Cmxn006627	Maritimetin	Flavonoids	up	down	down
Jmgn005927	2-hydroxynaringenin	Flavonoids	down	down	–
Hmqn003268	5,7,3′,4′,5’-Pentahydroxydihydroflavone	Flavonoids	–	–	–
Jmgn004021	6-C-Glucosyl-2-Hydroxynaringenin	Flavonoids	down	–	–
Zbsp007084	Butin; 7,3′,4′-Trihydroxyflavanone*	Flavonoids	–	down	–
HJN090	Butin-7-O-glucoside*	Flavonoids	–	up	–
MWSslk252	Didymin (Isosakuranetin-7-O-rutinoside)*	Flavonoids	up	up	up
Hmhp005335	Dihydrobaicalein	Flavonoids	up	down	down
mws0064	Eriodictyol (5,7,3′,4’-Tetrahydroxyflavanone)*	Flavonoids	–	down	–
HJN086	Eriodictyol-3’-O-glucoside*	Flavonoids	–	–	–
MWS20145	Eriodictyol-7-O-glucoside*	Flavonoids	down	–	–
pmb3023	Eriodictyol-8-C-glucoside*	Flavonoids	down	–	–
Lmqn009304	Eucalyptin (5-Hydroxy-7,4′-dimethoxy-6,8-dimethylflavone)	Flavonoids	up	up	up
mws0463	Hesperetin	Flavonoids	up	down	down
pmb2970	Hesperetin-5,7-di-O-glucoside	Flavonoids	down	–	–
pme1598	Hesperetin-5-O-glucoside	Flavonoids	down	–	–
MWSHY0019	Hesperetin-7-O-neohesperidoside(Neohesperidin)*	Flavonoids	–	–	–
Lahp003599	Homoeriodictyol-7,4′-di-O-β-D-glucopyranoside	Flavonoids	–	up	–
Labp005025	Malonyl isoSakuranin	Flavonoids	down	–	–
MWSHY0017	Naringenin (5,7,4′-Trihydroxyflavanone)*	Flavonoids	–	down	–
HJN087	Naringenin-4’-O-glucoside*	Flavonoids	–	–	–
pma0724	Naringenin-6-C-Glucoside	Flavonoids	down	–	down
mws1179	Naringenin-7-O-glucoside (Prunin)*	Flavonoids	down	–	–
mws1454	Persicoside	Flavonoids	down	–	–
mws0789	Pinocembrin (Dihydrochrysin)	Flavonoids	up	down	–
mws0791	Poncirin (Isosakuranetin-7-O-neohesperidoside)*	Flavonoids	–	up	–
mws0914	3,5,7-Trihydroxyflavanone (Pinobanksin)	Flavonoids	–	down	–
mws1094	Aromadendrin (Dihydrokaempferol)	Flavonoids	–	down	–
Lmtn002796	Aromadendrin-7-O-glucoside*	Flavonoids	down	–	–
Lmmn004625	Dihydrokaempferol-7-O-glucoside*	Flavonoids	down	–	–
mws0044	Taxifolin(Dihydroquercetin)	Flavonoids	–	down	–
Zbxn003562	Taxifolin-2-O-glucoside	Flavonoids	–	up	–
Xmsn002700	Taxifolin-3’-O-glucoside	Flavonoids	down	–	–
Lmdp003110	2,6,7,4’-Tetrahydroxyisoflavanone	Flavonoids	–	–	–
Lmqn006260	2′,3′,4′,5,7-Pentahydroxyflavone*	Flavonoids	up	–	–
Lmtp003948	3′,5′,5,7-Tetrahydroxy-4′-methoxyflavanone-3’-O-glucoside	Flavonoids	down	–	–
Zbqn006065	3,5,7,2’-Tetrahydroxyflavone; Datiscetin*	Flavonoids	–	down	–
Zmjp004875	5,6,3′,4’-Tetrahydroxy-3,7-dimethoxyflavone	Flavonoids	up	–	–
Zmjp004852	5,6,3′,4’-Tetrahydroxy-3,7-dimethoxyflavone-6-O-glucoside*	Flavonoids	–	up	–
Zmhn003257	5,7,2′-Trihydroxy-8-methoxyflavone*	Flavonoids	–	down	–
Hmmp007513	5,7-Dihydroxy-6,3′,4′,5′-tetramethoxyflavone (Arteanoflavone)*	Flavonoids	down	down	down
Zmhp003514	6,7,8-Tetrahydroxy-5-methoxyflavone	Flavonoids	up	up	–
Zmyn003693	6-Hydroxy-2′-methoxyflavone	Flavonoids	–	up	up
Hmcn002875	6-Hydroxyluteolin	Flavonoids	up	–	–
Hmcn001884	6-Hydroxyluteolin 5-glucoside*	Flavonoids	down	–	–
MWS20151	Apigenin; 4′,5,7-Trihydroxyflavone	Flavonoids	–	down	–
MWS20148	Apigenin-4’-O-glucoside*	Flavonoids	down	–	–
mws1073	Apigenin-6,8-di-C-glucoside (Vicenin-2)	Flavonoids	–	–	–
Lmtp002474	Apigenin-6-C-(2″-glucosyl)arabinoside	Flavonoids	up	up	–
pmp000237	Apigenin-6-C-(2″-glucuronyl)xyloside	Flavonoids	down	–	–
MWSHY0021	Apigenin-7,4′-dimethyl ether	Flavonoids	up	up	up
pmp000585	Apigenin-7-O-(6″-malonyl)glucoside	Flavonoids	down	–	–
Lmpp003930	Apigenin-7-O-(6″-p-Coumaryl)glucoside	Flavonoids	down	down	down
MWSHY0189	Apigenin-7-O-glucoside(Cosmosiin)*	Flavonoids	up	up	up
pmb2991	Apigenin-7-O-glucoside-4’-O-rutinoside	Flavonoids	down	–	–
pme0368	Apigenin-7-O-rutinoside (Isorhoifolin)	Flavonoids	down	–	down
Lmtp002642	Apigenin-8-C-(2″-glucosyl)arabinoside	Flavonoids	up	up	–
Lakn002234	Catechin glucosyl glucoside	Flavonoids	down	–	–
Lmyp005841	Chrysin-7-O-glucoside	Flavonoids	down	–	–
Lazp002140	Chrysoeriol glucosyl xylosyl glucoside	Flavonoids	down	–	–
pmb2999	Chrysoeriol-5-O-glucoside	Flavonoids	–	–	–
pmb0587	Chrysoeriol-7-O-(2”-O-glucuronyl)glucoside	Flavonoids	–	up	–
Zbsn005918	Chrysoeriol-7-O-(6″-acetyl)glucoside*	Flavonoids	–	–	–
pmb3012	Chrysoeriol-7-O-glucoside	Flavonoids	–	–	–
pmb3002	Chrysoeriol-7-O-rutinoside	Flavonoids	up	–	–
Zmjp009119	Chrysosplenetin (5,4’-Dihydroxy-3,6,7,3′-tetramethoxyflavone)*	Flavonoids	down	down	down
mws0058	Diosmetin (5,7,3′-Trihydroxy-4′-methoxyflavone)*	Flavonoids	–	down	–
pmp000579	Diosmetin-7-O-galactoside*	Flavonoids	down	–	–
Lmyn006227	Galangin (3,5,7-Trihydroxyflavone)	Flavonoids	–	down	–
Lmlp005572	Galangin-7-O-glucoside	Flavonoids	down	–	–
MWSslk237	Hispidulin-7-O-glucoside(Homoplantaginin)*	Flavonoids	up	–	–
Zbzp003056	Isoorientin-7-O-glucoside*	Flavonoids	down	–	–
Lmlp002990	Isosaponarin(Isovitexin-4’-O-glucoside)	Flavonoids	up	up	up
Lmyp003837	Isovitexin-7-O-glucoside-2”-O-rhamnoside	Flavonoids	down	–	–
Lmfn001893	Leucocyanidin	Flavonoids	down	up	up
pme0088	Luteolin (5,7,3′,4’-Tetrahydroxyflavone)	Flavonoids	–	down	–
MWS20147	Luteolin-3’-O-glucoside	Flavonoids	down	–	–
Hmpp003270	Luteolin-4’-O-glucoside*	Flavonoids	down	–	–
MWSHY0016	Luteolin-6-C-glucoside (Isoorientin)	Flavonoids	down	–	–
Zmlp003063	Luteolin-7-O-(2”-O-rhamnosyl)rutinoside*	Flavonoids	down	–	–
Hmjn004446	Luteolin-7-O-(6″-caffeoyl)rhamnoside	Flavonoids	up	up	up
pmp000587	Luteolin-7-O-(6″-malonyl)glucoside*	Flavonoids	–	–	–
MWSHY0104	Luteolin-7-O-glucoside (Cynaroside)*	Flavonoids	down	–	–
MWSHY0121	Luteolin-7-O-glucuronide	Flavonoids	–	–	–
MWSHY0080	Luteolin-7-O-neohesperidoside (Lonicerin)*	Flavonoids	–	–	down
Hmlp003068	Meratin*	Flavonoids	down	–	–
Hmmn004152	Neoeriocitrin	Flavonoids	–	up	–
Hmgp002036	Nepetin-7-O-glucoside(Nepitrin)*	Flavonoids	down	–	–
Ybnn007052	Norartocarpetin	Flavonoids	up	down	down
Zmjp003031	Orientin-2”-O-galactoside*	Flavonoids	down	–	–
Zbjp003056	Orientin-7-O-glucoside*	Flavonoids	down	–	–
Lmsp004632	Scutellarein (5,6,7,4’-Tetrahydroxyflavone)	Flavonoids	–	–	–
mws0920	Tricetin (5,7,3′,4′,5’-Pentahydroxyflavone)	Flavonoids	up	down	–
Lmzp004885	Tricin (5,7,4′-Trihydroxy-3′,5′-dimethoxyflavone)	Flavonoids	up	up	–
pmb3045	Tricin-7-O-Glucuronide	Flavonoids	down	–	–
Lcyp000688	Verecundin(Pinocembrin-5-glucoside)*	Flavonoids	down	–	–
Zbsn003878	Vicenin-3	Flavonoids	up	up	–
pmp001111	Violanthin	Flavonoids	down	–	–
Zmjp003291	Vitexin-2”-O-galactoside	Flavonoids	–	–	–
pme3227	Vitexin-2”-O-rhamnoside	Flavonoids	down	–	–
Lmjp003655	6-C-MethylKaempferol-3-glucoside*	Flavonoids	down	–	–
Zmhp102730	6-Hydroxykaempferol-3,6-O-Diglucoside	Flavonoids	–	–	–
pmp001312	6-Hydroxykaempferol-3,7,6-O-triglycoside	Flavonoids	–	–	up
pmp001311	6-Hydroxykaempferol-6,7-O-Diglucoside	Flavonoids	down	–	–
pmp001309	6-Hydroxykaempferol-7-O-glucoside	Flavonoids	–	–	–
Lmjp003295	6-Methoxykaempferol-3-O-glucoside*	Flavonoids	–	–	–
MWSHY0077	Fisetin	Flavonoids	–	–	–
Lmmp002143	Gossypetin-3-O-(6″-malonyl)glucoside*	Flavonoids	down	up	–
Lmmp001947	Gossypetin-3-O-glucoside*	Flavonoids	down	–	–
Lmmp002904	Gossypetin-3-O-glucoside-8-O-xyloside	Flavonoids	–	up	–
Zmhp003784	Gossypetin-7-O-(3″-glucosyl)rhamnoside; Rhodioflavonoside	Flavonoids	–	up	–
pmb0645	Hesperetin-6-C-glucoside-7-O-glucoside	Flavonoids	–	–	up
pmb0618	Hesperetin-8-C-glucoside-3’-O-glucoside	Flavonoids	down	–	–
mws0066	Isorhamnetin; 3′-Methoxy-3,4′,5,7-Tetrahydroxyflavone	Flavonoids	up	–	–
Hmcp001578	Isorhamnetin-3,7-O-diglucoside	Flavonoids	down	–	–
Hmcp001658	Isorhamnetin-3-O-(6″-malonyl)glucoside-7-O-glucoside	Flavonoids	down	–	–
Lmgp005640	Isorhamnetin-3-O-Galactoside; Cacticin	Flavonoids	–	–	–
Zbpp001992	Isorhamnetin-3-O-Glucoside*	Flavonoids	–	–	–
MWSHY0064	Isorhamnetin-3-O-neohesperidoside*	Flavonoids	–	–	–
Zbpp001877	Isorhamnetin-3-O-rutinoside*	Flavonoids	down	–	–
Hmcp002207	Isorhamnetin-7-O-glucoside (Brassicin)*	Flavonoids	–	–	–
MWSHY0201	Kaempferol-3,7-O-dirhamnoside (Kaempferitrin)	Flavonoids	down	–	down
Lmdp004819	Kaempferol-3-O-(2″-sinapoyl)glucosyl-(1 → 2)-(6″-malonyl)glucoside	Flavonoids	down	down	–
Lmdp004892	Kaempferol-3-O-(6″-malonyl)galactoside*	Flavonoids	down	–	–
Lmmp003817	Kaempferol-3-O-(6″-malonyl)glucoside*	Flavonoids	–	–	–
Hmcp001629	Kaempferol-3-O-(6″-Malonyl)glucoside-7-O-Glucoside	Flavonoids	down	–	–
Lmmn003398	Kaempferol-3-O-(6”-*O*-acetyl)glucoside	Flavonoids	down	–	–
Xmyp004945	Kaempferol-3-O-(6”-Rhamnosyl-2″-Glucosyl)Glucoside (Camelliaside A)	Flavonoids	–	–	up
mws0913	Kaempferol-3-O-galactoside (Trifolin)*	Flavonoids	down	–	–
Lmsp004670	Kaempferol-3-O-glucoside-7-O-rhamnoside*	Flavonoids	–	–	down
mws1035	Kaempferol-3-O-robinoside-7-O-rhamnoside (Robinin)*	Flavonoids	down	–	–
Lmsp003161	Kaempferol-3-O-sophoroside-7-O-rhamnoside*	Flavonoids	down	–	–
Lmqp002170	Kaempferol-3-O-sophorotrioside	Flavonoids	down	–	–
Xmyp005654	Kaempferol-4’-O-glucoside*	Flavonoids	down	–	–
HJAP023	Kaempferol-6,8-di-C-glucoside-7-O-glucoside	Flavonoids	down	–	–
mws0089	Kaempferol-7-O-glucoside*	Flavonoids	down	–	–
pme3514	Morin*	Flavonoids	up	–	–
mws0032	Myricetin	Flavonoids	–	up	–
Lmtp004126	Myricetin-3-O-(6″-malony)glucoside*	Flavonoids	down	up	–
pmn001640	Myricetin-3-O-arabinoside	Flavonoids	down	up	up
Lmdp002969	Myricetin-3-O-galactoside*	Flavonoids	down	–	–
Lmpp003465	Myricetin-3-O-β-D-glucoside*	Flavonoids	down	–	–
Zahp003364	myricetin-3-O-β-d-xylopyranosyl-(1 → 2)-β-D-glucopyranoside	Flavonoids	down	up	–
Lakp003108	Myricetindiglucoside	Flavonoids	down	up	up
Hmgp001996	Quercetagetin; 3,3′,4′,5,6,7-Hexahydroxyflavone	Flavonoids	up	–	–
Lmfp003403	Quercetagetin-7-O-glucoside(Quercetagitrin)*	Flavonoids	down	up	–
Lmcp004369	Quercetin-3′,4′-dimethyl ether	Flavonoids	–	–	–
Zmcp002666	Quercetin-3,7-Di-O-glucoside*	Flavonoids	down	–	–
Zmcp003530	quercetin-3-hydroxyferuloyldiglucoside	Flavonoids	–	–	–
Lmyp003588	Quercetin-3-O-(2″,6”-O-digalloyl)-glucoside	Flavonoids	down	down	down
Lmdp004221	Quercetin-3-O-(2”-O-caffeoyl)glucoside-(1 → 2)-(6″-Malonyl)glucoside	Flavonoids	down	–	down
pmp000596	Quercetin-3-O-(2”-O-galactosyl)glucoside	Flavonoids	–	–	–
Lmfp005436	Quercetin-3-O-(2”-O-galloyl)Arabinoside	Flavonoids	up	down	down
MWSHY0142	Quercetin-3-O-(2”-O-galloyl)galactoside	Flavonoids	–	–	up
Lmmp003266	Quercetin-3-O-(2”-O-malonyl)glucoside-7-O-arabinoside	Flavonoids	down	–	–
Lmjp003360	Quercetin-3-O-(2”-O-malonyl)sophoroside-7-O-arabinoside	Flavonoids	–	–	up
Hmcp001618	Quercetin-3-O-(2”-O-Xylosyl)rutinoside	Flavonoids	–	–	up
Hmln002199	Quercetin-3-O-(6”-*O*-acetyl)galactoside	Flavonoids	–	–	–
Zmsp004363	Quercetin-3-O-(6”-*O*-acetyl)glucoside	Flavonoids	–	up	up
Hmln001682	Quercetin-3-O-(6”-*O*-acetyl)glucosyl-(1 → 3)-Galactoside	Flavonoids	down	–	–
Zbsp004136	Quercetin-3-O-(6”-O-arabinosyl)glucoside*	Flavonoids	–	–	–
MWSHY0199	Quercetin-3-O-(6”-O-galloyl)galactoside	Flavonoids	–	–	down
pmb0706	Quercetin-3-O-(6”-O-malonyl)glucosyl-5-O-glucoside	Flavonoids	down	–	–
Lmfp002421	Quercetin-3-O-[rhamnosyl(1 → 2)glucosyl]-5-O-glucoside*	Flavonoids	down	–	–
Lmdp003286	Quercetin-3-O-alloside; Isohyperoside*	Flavonoids	down	–	–
MWSHY0113	Quercetin-3-O-galactoside (Hyperin)*	Flavonoids	down	–	–
Hmcp001769	Quercetin-3-O-rhamnosyl(1 → 2)arabinoside	Flavonoids	down	–	–
Lnrp102163	Quercetin-3-O-rutinoside-7-O-rhamnoside	Flavonoids	–	–	up
Lmjp002596	Quercetin-3-O-sambubioside*	Flavonoids	–	–	–
Lmsp002982	Quercetin-3-O-sophoroside-7-O-rhamnoside*	Flavonoids	down	–	–
Lmdp003509	Quercetin-3-O-xyloside (Reynoutrin)*	Flavonoids	down	–	–
Lmmp003306	Quercetin-3-O-xylosyl(1 → 2)arabinoside	Flavonoids	–	–	–
Zmgp002857	Quercetin-3-O-α-rhamnosyl (1 → 2)-[α-rhamnosyl (1 → 6)]-β-glucoside	Flavonoids	–	up	–
mws0856	Quercetin-4’-O-glucoside (Spiraeoside)*	Flavonoids	down	–	–
Lmfn003760	Quercetin-4’-O-glucuronide	Flavonoids	–	up	–
Smgp004575	Quercetin-5-O-β-D-glucoside*	Flavonoids	–	–	–
pmp000589	Quercetin-7-O-(6″-malonyl)glucoside	Flavonoids	down	–	–
mws1329	Quercetin-7-O-glucoside	Flavonoids	down	–	–
Lmjp002906	Rhamnetin-3-O-Glucoside*	Flavonoids	down	–	–
Zmpp002571	Robinetin	Flavonoids	down	–	–
mws2627	Tamarixetin (3,3′,5,7-Tetrahydroxy-4′-Methoxyflavone)	Flavonoids	up	up	–
Lahp003408	2-(3,4-dihydroxyphenyl)-4 h-chromene-3,5,7-triol-glucoside	Flavonoids	down	–	–
Lmmp003109	3’-O-Methyl-epicatechin	Flavonoids	down	–	–
pme3285	Afzelechin (3,5,7,4’-Tetrahydroxyflavan)	Flavonoids	down	–	–
mws0355	Catechin gallate*	Flavonoids	up	up	up
pmn001416	Catechin-(7,8-bc)-4α-(3,4-dihydroxyphenyl)-dihydro-2-(3H)-one	Flavonoids	down	–	–
pmn001415	Catechin-(7,8-bc)-4β-(3,4-dihydroxyphenyl)-dihydro-2-(3H)-one	Flavonoids	down	–	–
Zajn002491	catechin-4-β-D-galactopyranoside*	Flavonoids	down	–	–
pmb2947	Catechin-catechin-catechin	Flavonoids	down	–	–
pme0460	Epicatechin*	Flavonoids	down	up	–
mws1397	Epicatechin gallate*	Flavonoids	up	up	up
HJN041	Epicatechin glucoside	Flavonoids	down	–	–
Zmdn002400	Epicatechin-3’-O-β-D-glucopyranoside*	Flavonoids	down	–	–
Zmdn002049	Epicatechin-4’-O-β-D-glucopyranoside*	Flavonoids	down	–	–
pmb3114	Epicatechin-epiafzelechin	Flavonoids	down	–	–
MWSHY0098	Epigallocatechin	Flavonoids	–	up	–
mws0034	Epigallocatechin-3-gallate*	Flavonoids	–	up	–
Lajp003377	Fisetinidol-(4α,6)-gallocatechin	Flavonoids	down	–	–
mws0049	Gallocatechin	Flavonoids	down	up	up
mws2220	Gallocatechin 3-O-gallate*	Flavonoids	–	up	–
Lajp002779	Gallocatechin-(4α- > 8)-Catechin-(4α- > 8)-Catechin	Flavonoids	down	–	–
Lmmp000897	Gallocatechin-(4α → 8)-gallocatechin	Flavonoids	down	–	–
Wbtp004753	1,2,3,7,8-pentahydroxy-6-methylanthracene-9,10-dione	Flavonoids	down	–	–
Wbtn006715	1,2,4,5,8-pentahydroxy-6-methylanthracene-9,10-dione*	Flavonoids	up	–	–
Wbtp004961	1,2,4,5-tetrahydroxy-7-(hydroxymethyl)anthracene-9,10-dione	Flavonoids	down	–	–
pmn001477	4-C-Glucose-1,3,6-trihydroxy-7-methoxyxanthone	Flavonoids	down	–	–
Satp005088	5-hydroxy-7-(2-hydroxypropyl)-2-(3-hydroxy-2-(4-hydroxy-3,5-dimethoxybenzyl)propyl)-chromone	Flavonoids	up	–	–
Wbtn006810	6,7-dihydroxy-1,3-dimethoxyxanthen-9-one*	Flavonoids	–	down	–
Lcsn003955	Maesopsin	Flavonoids	–	down	–
MA10079134	1,2,4-Trihydroxyanthraquinone	Quinones	–	–	–
MWS20169	2,5-dihydroxy-1-methoxy-anthraquinone	Quinones	up	up	–
Zbn006783	5,6-Dihydroxylucidin	Quinones	up	–	–
Zmdn006929	Aloe-Emodin-9-Anthrone	Quinones	up	down	–
Zdhn005610	Citreorosein	Quinones	up	down	down
Zbqn006094	Pseudopurpurin(1,2,4-trihydroxy-3-carboxyanthraquinone)	Quinones	up	–	–
Wmcp000011	Rheic Acid	Quinones	up	–	–
pmn001338	Rhein-8-O-glucoside	Quinones	down	–	–
Zbln013951	1-(3′-methoxy-4′-hydroxybenzyl)-2,7-dihydroxy-4-methoxyphenanthrene	Quinones	down	down	down
Zbln007388	4,6-dimethoxy-9,10-dihydrophenanthrene-2,3,7-triol	Quinones	up	down	down
Wmhp000055	2,5-Dimethoxybenzoquinone*	Quinones	up	–	–
Zmsp001834	2,6-Dimethoxy-1,4-benzoquinone*	Quinones	up	–	–
pmn001369	1-Hydroxypineolin Diglucoside	Lignans and Coumarins	–	up	–
pmn001375	1-Hydroxypinoresinol-1-O-Glucoside	Lignans and Coumarins	down	–	–
Zmcn003729	1-Hydroxypinoresinol-4’-O-Glucoside	Lignans and Coumarins	–	–	–
WaYn005928	Buddlenol B*	Lignans and Coumarins	up	down	–
WaYn006596	Buddlenol C	Lignans and Coumarins	up	–	–
WaYn006188	Buddlenol E*	Lignans and Coumarins	up	–	–
Lazn006616	Buddlenol F	Lignans and Coumarins	up	–	–
Lmsp004450	Dehydrodiconiferyl alcohol	Lignans and Coumarins	up	down	–
Cmsp003083	Dehydrodiconiferyl alcohol-4-O-glucoside*	Lignans and Coumarins	–	–	–
Lmsp003655	Dehydrodiconiferyl alcohol-gamma’-O-glucoside*	Lignans and Coumarins	–	–	–
Lmtn002596	Dihydrodehydrodiconiferyl alcohol-4-O-glucoside*	Lignans and Coumarins	down	–	–
Lskp211385	dihydrosesamin	Lignans and Coumarins	up	down	–
MWSHC20189	Epipinoresinol*	Lignans and Coumarins	–	down	–
Lazn003893	Erythro-Guaiacylglycerol-β-Coniferyl Ether	Lignans and Coumarins	up	down	–
Lazn006735	Erythro-Guaiacylglycerol-β-O-4′-dehydrodisinapyl Ether	Lignans and Coumarins	up	down	–
Lazn005262	Erythro-Guaiacylglycerol-β-Sinapyl Ether	Lignans and Coumarins	up	–	–
WaYn003112	Erythro-Guaiacylglycerol-β-threo-syringylglycerol Ether	Lignans and Coumarins	up	–	–
Lhhp102922	Fargesin	Lignans and Coumarins	–	–	–
Lazn002951	Guaiacylglycerol-β-Guaiacyl Ether	Lignans and Coumarins	up	–	–
Hmgn004139	Isohydroxymatairesinol	Lignans and Coumarins	–	up	–
Lmmn003748	Isolariciresinol	Lignans and Coumarins	up	down	–
Lmmn002274	Isolariciresinol-9’-O-glucoside*	Lignans and Coumarins	down	–	–
HJN083	Lariciresinol-4’-O-glucoside	Lignans and Coumarins	–	–	–
Hmcn002743	Lirioresinol A	Lignans and Coumarins	up	–	–
Lmmn003020	Lyoniresinol	Lignans and Coumarins	–	down	–
Cmmn005231	Massoniresinol; Vladinol A	Lignans and Coumarins	–	–	–
Lssp210087	Matairesinol	Lignans and Coumarins	up	down	–
Lmmn003875	Medioresinol	Lignans and Coumarins	–	down	down
Rfmb056	Medioresinol-4’-O-(6″‘-acetyl)glucoside	Lignans and Coumarins	up	–	–
pmn001376	Olivil	Lignans and Coumarins	up	down	–
mws0097	Pinoresinol*	Lignans and Coumarins	–	down	–
pmn001378	Pinoresinol-4-O-glucoside	Lignans and Coumarins	up	up	up
Lskp211262	Secoisolariciresinol	Lignans and Coumarins	–	–	up
MWSmce499	Secoisolariciresinol diglucoside	Lignans and Coumarins	–	–	–
Cmsn002480	Secoisolariciresinol-9’-O-glucoside	Lignans and Coumarins	up	–	–
MWS20152	Syringaresinol	Lignans and Coumarins	up	up	–
MWSHC2047	Syringaresinol-4’-O-glucoside; Acanthoside B	Lignans and Coumarins	up	–	–
Hmqn002118	7-Hydroxycoumarin;Umbelliferone	Lignans and Coumarins	up	down	–
mws1075	7-Methoxycoumarin	Lignans and Coumarins	–	–	up
Lhkp101525	Apiosylskimmin (Adicardin)	Lignans and Coumarins	down	–	–
pmn001492	Ayapin	Lignans and Coumarins	up	–	–
MWSmce301	Coumarin-3-carboxylic Acid	Lignans and Coumarins	up	–	up
mws1074	Daphnetin	Lignans and Coumarins	–	down	–
Cmyn001328	Daphnin*	Lignans and Coumarins	down	up	up
mws1013	Esculetin (6,7-Dihydroxycoumarin)	Lignans and Coumarins	–	down	–
Lmbn001162	Esculetin-7-O-glucoside*	Lignans and Coumarins	down	up	up
pmb3093	Esculetin-7-O-quinic acid	Lignans and Coumarins	down	–	–
mws1015	Esculin (6,7-Dihydroxycoumarin-6-O-glucoside)*	Lignans and Coumarins	down	up	up
MWSmce025	Fraxetin-8-O-glucoside (Fraxin)	Lignans and Coumarins	–	up	up
Lcyp000676	Isoscopoletin-β-D-glucoside*	Lignans and Coumarins	down	–	–
Lmwp102713	Peucedanol*	Lignans and Coumarins	up	–	–
MWSCX014	Scopoletin (7-Hydroxy-6-methoxycoumarin)*	Lignans and Coumarins	up	–	–
Lhqp101805	Skimmin (7-Hydroxycoumarin-7-O-glucoside)	Lignans and Coumarins	–	–	–
Lmjp002764	Umckalin (7-hydroxy-5,6-dimethoxycoumarin)	Lignans and Coumarins	–	–	down
Qmzp101901	Zanthoxyloside*	Lignans and Coumarins	down	–	–
Lasp003143	1,4-Benzodioxin-6-propanol	Others	–	–	–
Jmzn006005	3,4-methylenedioxy cinnamyl alcohol	Others	–	up	–
pmn001380	Eucommiol	Others	up	–	–
Cmyp007180	Dihydroactinidiolide	Others	up	–	–
pmb0128	δ-Tridecalactone	Others	up	–	–
Waln010743	1-(2,3-dihydroxypropoxy)-3-(((2-(dimethylamino)ethoxy)(hydroxy)phosphoryl)oxy)propan-2-yl (11Z,14Z)-octadeca-11,14-dienoate	Others	–	down	–
Waln009920	1-(2,3-dihydroxypropoxy)-3-(((2-(dimethylamino)ethoxy)(hydroxy)phosphoryl)oxy)propan-2-yl (8E,11Z,14Z)-octadeca-8,11,14-trienoate	Others	–	down	–
Waln010449	1-(2,3-dihydroxypropoxy)-3-(((2-(dimethylamino)ethoxy)(hydroxy)phosphoryl)oxy)propan-2-yl (E)-hexadec-9-enoate	Others	–	down	up
Waln011704	1-(2,3-dihydroxypropoxy)-3-(((2-(dimethylamino)ethoxy)(hydroxy)phosphoryl)oxy)propan-2-yl (Z)-14-Octadecenoic Acid	Others	–	down	–
WaYn011606	1-(2,3-dihydroxypropoxy)-3-(((2-(dimethylamino)ethoxy)(hydroxy)phosphoryl)oxy)propan-2-yl heptadecanoate	Others	–	down	up
Waln011524	1-(2,3-dihydroxypropoxy)-3-(((2-(dimethylamino)ethoxy)(hydroxy)phosphoryl)oxy)propan-2-yl palmitate*	Others	–	down	–
Wcdp006741	1-(9Z,12Z-Octadecadienoyl)-Sn-Glycero-3-Phosphocholine	Others	–	down	–
Wcsn010224	14-hydroxy-2,6,10-trimethylpentadeca-2,5,10-trien-4-one*	Others	–	down	–
WaYn011395	2-(2,3-dihydroxypropoxy)-3-(((2-(dimethylamino)ethoxy)(hydroxy)phosphoryl)oxy)propan-2-yl (Z)-14-Octadecenoic Acid	Others	–	down	–
Waln011009	2-(2,3-dihydroxypropoxy)-3-(((2-(dimethylamino)ethoxy)(hydroxy)phosphoryl)oxy)propyl (11Z,14Z)-octadeca-11,14-dienoate	Others	–	down	up
Waln010192	2-(2,3-dihydroxypropoxy)-3-(((2-(dimethylamino)ethoxy)(hydroxy)phosphoryl)oxy)propyl (8E,11Z,14Z)-octadeca-8,11,14-trienoate	Others	–	down	–
Waln011222	2-(2,3-dihydroxypropoxy)-3-(((2-(dimethylamino)ethoxy)(hydroxy)phosphoryl)oxy)propyl palmitate*	Others	–	down	up
Lhyp111013	2(4H)-benzofuranone	Others	down	–	–
Hasp010605	2,3-dihydroxypropyl (9Z,12Z,15Z)-octadeca-9,12,15-trienoate	Others	down	down	–
pmb0302	2-Aminoethylphosphonate	Others	–	up	up
Wagp002074	3-(1-hydroxyethyl)-4-methylpentane-1,4-diol O-Glucoside	Others	down	–	–
Wcgp006109	3-(2-hydroxyethyl)-5,7-dimethoxy-4-methyl-2H-1-benzopyran-2-one*	Others	up	–	–
Wdbn005328	3,3’-Bis(3,4-dihydro-4-hydroxy-6,8-dimethoxy-2H-1-benzopyran)	Others	up	–	–
Lskp211267	3-Ethyl-7-hydroxyphthalide	Others	–	–	–
Wagp005297	3-Hydroxy-beta-ionol 3-Glucoside*	Others	down	–	–
Wcdp001930	3ξ-(1ξ-hydroxyethyl)-7-hydroxy-1-isobenzofuranone	Others	down	–	–
Wagp001892	4-(Beta-D-Glucopyranosyloxy)-2-Pentanol	Others	down	–	–
Wcsn010254	4-[3-(4,8-dimethylnona-3,7-dienyl)-3-methyloxiran-2-yl]butan-2-one*	Others	–	down	–
Zmmp002106	4-methyl-1,5,2,3-dioxadiazinan-2-amine	Others	down	–	up
pmb0764	4-Methyl-5-thiazoleethanol	Others	up	up	up
zjgp122321	4-*O*-acetyl-3-O-caffeoyl-2-*C*-methyl-D-erythronate*	Others	down	–	–
Zbzp007397	5,6,7,7a-tetrahydro-4,4,7a-trimethyl-2(4H)-benzofuranone	Others	up	up	–
Wcgp009361	5,7-diethoxy-3-(2-hydroxyethyl)-4-methyl-2H-1-benzopyran-2-one	Others	up	–	–
Lmln001856	5,7-Dihydroxy-4-oxo-2-(3,4,5-trihydroxyphenyl)-4H-chromen-3-yl-alloside	Others	down	up	–
Zahn007990	5-hydroxy-3,4-dimethyl-5-pentylfuran-2(5H)-one	Others	–	up	up
Lhmp122205	5-O-Methyllatifolin	Others	–	up	–
Wcsn010496	6,10,14-Trimethylpentadeca-5,9-Diene-2,13-Dione	Others	–	down	–
Hahn002084	6’-O-caffeoylcatalpol*	Others	up	–	–
Wcsn011125	Ethyl 15,16-dihydroxy-5,9-dimethyloctadeca-4,6,8,10,13-pentaenoate	Others	–	down	–
Lhhp120823	Eugenyl formate	Others	–	up	–
Wasn005621	Glucosyl 9-hydroxy-3-methyldec-2-enoic acid	Others	up	–	–
Lcsp012679	linolenoylethanolamine	Others	–	–	down
Lmhp001671	Noreugenin-7-O-glucoside*	Others	down	–	–
MWSmce581	2,4-Dihydroxybenzaldehyde	Others	up	up	–
Lmbn001981	2,5-Dihydroxybenzaldehyde*	Others	up	down	down
Wmlp000056	3,5-Dimethoxy-4-hydroxybenzaldehyde	Others	up	down	down
Lmbn002737	3-Methylbenzaldehyde	Others	–	–	–
MWSmce644	4-Acetoxy-3-Ethoxybenzaldehyde	Others	up	up	up
Zmdp000376	4-Guanidinobutanal	Others	down	–	–
mws0628	4-Hydroxybenzaldehyde	Others	up	–	up
MWSCX017	4-hydroxyphenyl acrylaldehyde*	Others	up	down	down
MWS1852	4-Methoxybenzaldehyde	Others	up	down	–
MWS20172	5-Hydroxymethylfurfural	Others	–	up	–
Hmgn001653	Protocatechualdehyde*	Others	up	down	down
mws1350	Syringaldehyde; 4-Hydroxy-3,5-Dimethoxybenzaldehyde	Others	up	–	–
mws0458	Vanillin; 4-Hydroxy-3-Methoxybenzaldehyde*	Others	up	up	up
Hmyp002315	3,5,7,4’-Tetrahydroxy-Coumaronochromone	Others	down	–	–
Layp002880	5,7-Dihydroxychromone glucoside	Others	down	up	up
Zmzp006646	Capillarisin	Others	up	up	–
MWSmce658	Noreugenin; 5,7-Dihydroxy-2-Methylchromone	Others	–	down	–
MWS0559	1,6-anhydro-β-d-glucose	Others	up	–	up
Wafn004792	1-O-Acetyl-Glucopyranose 6-Decanoate	Others	up	–	–
Lcsn000341	3’-Fucosyllactose	Others	down	–	–
Zmgn000447	3-Phospho-D-glyceric acid	Others	down	–	down
ML10171848	D-Arabinono-1,4-lactone*	Others	–	–	–
Zmzn000079	D-Erythrose-4-phosphate	Others	down	–	down
MWS2442	d-Fructose 6-Phosphate*	Others	–	–	–
MWSmce220	D-Glucono-1,5-lactone*	Others	up	up	–
Zmyn000110	D-Glucosamine 1-phosphate	Others	down	–	–
mws0866	d-Glucose 6-phosphate*	Others	–	up	–
mws1090	d-Glucose-1-phosphate*	Others	–	up	–
mws4175	D-Glucurono-6,3-lactone	Others	–	up	–
Lmxn000380	Digalactosylglycerol	Others	–	down	–
MA10039641	d-Lactose*	Others	down	–	–
Lmsn000381	D-Maltose*	Others	–	–	–
mws1593	D-Maltotetraose	Others	up	–	up
pme0500	D-Melezitose	Others	up	–	up
mws1589	D-Panose*	Others	up	–	–
pme3163	D-Sedoheptuiose 7-phosphate	Others	down	down	down
pme0519	D-Sucrose*	Others	–	–	–
mws0889	D-Threonic Acid	Others	up	–	–
pma0134	D-Threose	Others	–	up	–
mws0264	D-Trehalose*	Others	–	–	–
mws1080	Galactinol	Others	–	–	–
pmb3081	Glucaric acid-1-Phosphate	Others	–	down	down
MWSmce113	Guaifenesin	Others	up	up	up
pme2253	L-Gulono-1,4-Lactone*	Others	up	up	up
MWS1983	Maltitol	Others	–	–	–
MWS0442	Maltotriose	Others	up	–	–
mws1333	Melibiose	Others	down	–	–
mws2608	N-Acetyl-D-galactosamine	Others	up	–	–
pmb3079	N-Acetyl-D-glucosamine-1-phosphate	Others	–	down	up
mws4174	N-Acetyl-D-mannosamine	Others	up	–	–
mws4163	Nystose	Others	up	–	–
pme2125	Raffinose*	Others	up	–	–
Lmmn000214	Solatriose	Others	down	down	down
Lmqn000213	Stachyose	Others	up	–	–
mws1089	Sucrose-6-phosphate	Others	–	up	up
mws2523	Trehalose 6-phosphate	Others	–	up	up
MWSslk225	1-Indanone	Others	–	down	–
Lafp003256	3,4’-Dihydroxy-3′,5′-dimethoxypropiophenone	Others	up	–	–
Lmyp003951	3-Hydroxy-1-(4-hydroxy-3,5-dimethoxyphenyl)propan-1-one	Others	–	–	down
MWSslk095	4-Hydroxy-2,5-dimethyl-3(2H)furanone	Others	down	–	–
MWSmce466	4-Hydroxyacetophenone	Others	up	–	–
MWSmce283	4’-Hydroxypropiophenone	Others	up	–	–
Wbtn006721	6,8-dihydroxy-3-methyl-9-oxoxanthene-1-carboxylic acid*	Others	–	down	–
MWSslk226	Benzylacetone	Others	–	down	down
Wbmp003302	Frambinone	Others	down	up	up
MWSHC20159	Roseoside	Others	–	–	–
Lmtp004146	Santalin	Others	down	–	–
MWS0434	Trans-dehydrorosinone	Others	up	up	up
Wasn000528	2-O-α-d-Glucopyranosyl-l-ascorbic acid	Others	down	–	–
pmb0801	4-Pyridoxic acid-O-glucoside	Others	down	–	–
MA10039492	Dehydroascorbic acid	Others	down	–	down
MWSmce690	Erythorbic Acid; Isoascorbic Acid	Others	–	up	–
Wasn001007	Isoascorbic acid 2-O-glucoside	Others	down	–	–
MWSmce039	Isonicotinic acid	Others	up	–	up
mws0133	Nicotinamide	Others	up	up	up
pma3101	Nicotinate D-ribonucleoside	Others	up	–	up
pme0490	Nicotinic acid (Vitamin B3)	Others	up	–	–
pme3511	Orotidine	Others	up	–	up
MWSmce674	Phylloquinone (Vitamin K1)	Others	down	–	down
Zmjp000624	Pyridoxal	Others	down	–	down
mws0655	Pyridoxal-5′-phosphate	Others	down	–	down
pme1383	Pyridoxine	Others	up	down	–
pmb0790	Pyridoxine-5’-O-diglucoside	Others	down	–	–
pmb0789	Pyridoxine-5’-O-glucoside	Others	down	–	–
pme1306	Pyridoxine-5′-phosphate	Others	up	–	–
pme2289	Retinol (Vitamin A1)	Others	down	–	–
mws0232	Riboflavin (Vitamin B2)	Others	up	up	up
MWSmce489	2,3,5,4’-Tetrahydroxystilbene-2-O-glucoside	Others	down	–	down
Lmtp004915	3,5-Dihydroxy-3′,4′-diacetoxylstilbene-3-O-glucoside	Others	–	up	–
MWSmce484	Astringin	Others	up	–	–
mws0021	Resveratrol	Others	up	–	–
Zmlp005013	3,3’-*O*-Dimethylellagic Acid	Tannins	up	–	–
Lmyn004187	Flavogallonic Acid Dilactone	Tannins	down	–	–
pme1738	3-Carbamyl-1-methylpyridinium;(1-Methylnicotinamide)	Alkaloids	up	up	–
pma6298	3-Hydroxypyridine	Alkaloids	–	up	–
Zblp001009	3-pyridine-methanol-O-β-d-glucopyranosyl	Alkaloids	down	–	–
pmf0291	4-Hydroxypyridine	Alkaloids	–	up	–
MWS3270	Quinolinic Acid	Alkaloids	up	–	–
Yacp000453	3-hydroxy-1-methylpyrrolidin-2-one*	Alkaloids	–	–	up
Zmsp000878	4-Hydroxy-5-(2-oxo-1-pyrrolidinyl)benzoic acid*	Alkaloids	down	–	–
MWStz081	Piperlotine C; 1-(3,4,5-Trimethoxycinnamoyl)pyrrolidine	Alkaloids	–	–	–
MWS1860	Pyrrolidin	Alkaloids	down	–	up
MWSmce157	Stachydrine	Alkaloids	up	–	–
MWSslk106	2-Phenylethylamine	Alkaloids	down	–	–
MWS2076	2-Aminophenol	Alkaloids	–	up	–
MWSmce462	4-Hydroxybenzylamine	Alkaloids	–	down	–
Lmgp000796	4-Hydroxymandelonitrile	Alkaloids	down	–	up
MWS0435	Acetaminophen	Alkaloids	up	–	–
pma0101	Caffeoylagmatine	Alkaloids	–	up	up
pmp001244	Caffeoylcholine	Alkaloids	–	up	–
Hmcp009963	Candicine	Alkaloids	down	down	down
MWSmce521	Dobutamine	Alkaloids	down	down	down
MWStz070	N-(2-Hydroxy-4-methoxyphenyl)acetamide	Alkaloids	down	down	–
Wagp001741	N-(gamma-L-glutamyl)tyramine O-glucoside	Alkaloids	down	–	–
pmb0492	N′,N″,N″‘-p-Coumaroyl-cinnamoyl-caffeoyl spermidine	Alkaloids	down	–	up
Zdcp003644	N-Caffeoylputrescine	Alkaloids	down	–	–
pmb0496	N-Feruloylagmatine	Alkaloids	down	up	up
Lmhp003013	N-Feruloyl-Cadaverine	Alkaloids	–	–	down
MWSmce098	Nonivamide	Alkaloids	–	up	–
pmb0490	p-Coumaroylputrescine	Alkaloids	–	–	–
Lmqp002784	Salicylamide	Alkaloids	down	–	–
Lahp002608	3,5-Dihydro-2H-Furo[3,2-*C*]Quinolin-4-One*	Alkaloids	down	–	up
Ladp002935	3-quinolinecarboxylic acid	Alkaloids	–	–	up
Lmgp001898	4,6-Dihydroxyquinoline	Alkaloids	–	–	–
Ladp002110	4-hydroxy-2-oxo-1,2-dihydroquinoline-3-carboxylic acid	Alkaloids	down	–	–
Wcjp002598	8-hydroxyquinoline	Alkaloids	down	–	up
Wasn002329	Xanthurenic Acid 8-O-Glucoside	Alkaloids	down	–	–
MWStz063	2-Ethyl-2,6,6-trimethylpiperidin-4-one	Alkaloids	up	up	up
pmp001198	6-Deoxyfagomine	Alkaloids	down	–	up
pmb0782	Piperidine	Alkaloids	down	–	up
MWStz152	2-(Acetylamino)-3-phenyl-2-propenoic acid*	Alkaloids	–	–	–
MWSmce645	2-(Aminooxy)Acetic Acid	Alkaloids	up	up	up
MWSslk118	2,2’-Cyclouridine	Alkaloids	–	up	–
MWS2063	2,6-Dimethylaniline	Alkaloids	down	down	–
MWSmce557	2-Acetyl-3-ethylpyrazine	Alkaloids	–	down	–
MWS1936	2-Methyl-5-nitroimidazole-1-ethanol	Alkaloids	–	–	down
Hmgp002327	3-amino-2-naphthoic acid*	Alkaloids	down	–	up
MWS1777	3-Chloroaniline	Alkaloids	–	up	up
MWSmce128	4(3H)-Quinazolinone	Alkaloids	–	up	–
Hmtp000776	4,5,6-Trihydroxy-2-cyclohexen-1-ylideneacetonitrile	Alkaloids	–	–	up
Lamp000484	4-Methylazetidine-2-Carboxylic acid*	Alkaloids	–	–	up
pma3649	5-Aminolevulinic Acid*	Alkaloids	down	–	up
MWS2072	5-Nitrobenzimidazole	Alkaloids	–	up	–
Wbjp004368	8-Hydroxy-harmine	Alkaloids	down	–	–
MWSmce207	Acetylpyrazine	Alkaloids	–	–	–
pmb0501	Agmatine	Alkaloids	–	up	up
MWS3105	Aniline	Alkaloids	–	–	–
MWSmce331	Azetidine-2-carboxylic acid*	Alkaloids	down	–	–
MWSmce548	Betaine	Alkaloids	up	up	–
mws2218	Caffeine	Alkaloids	up	up	up
pmb0484	Choline	Alkaloids	–	down	down
MWS1784	Cyclohexylamine	Alkaloids	down	–	–
mws1346	DL-2-Aminoadipic acid*	Alkaloids	up	–	–
MWSmce448	Imidazol-1-yl-acetic acid*	Alkaloids	up	–	–
MWSmce461	L-Azetidine-2-carboxylic acid*	Alkaloids	down	–	–
pme1002	L-Tyramine	Alkaloids	down	–	–
Zasp102439	*m*-Aminophenylacetylene	Alkaloids	down	–	up
Zajp000573	N-(4-oxopentyl)-acetamide*	Alkaloids	up	up	up
pme2693	N-Acetylputrescine	Alkaloids	–	up	up
Smcp000882	N-benzoyl-2-aminoethyl-β-*D*-glucopyranoside	Alkaloids	–	up	up
pmp001287	*N*-Benzylmethylene isomethylamine	Alkaloids	down	–	up
MWS0700	Neopterin	Alkaloids	up	down	–
MWSmce571	N-Methylbenzylamine	Alkaloids	down	–	–
Qmdp090606	N-Methyltetrahydropalmatine	Alkaloids	down	down	–
mws0983	N-Oleoylethanolamine	Alkaloids	up	–	–
Zmpp000906	Norepinephrine	Alkaloids	up	down	–
Wbjp001169	o-Carboxy-5-hydroxytryptamine	Alkaloids	down	–	–
pmb1754	O-Phosphocholine	Alkaloids	down	–	–
pma0948	Phenylethanolamine	Alkaloids	up	–	up
MWS1919	Thiazole	Alkaloids	down	–	–
Wbmp002283	Α-hydroxyquinoline	Alkaloids	down	–	up
Cmyp003522	3’-Hydroxy-*N*-methylcoclaurine	Alkaloids	up	down	–
MWSmce709	Isoquinoline	Alkaloids	down	–	up
MWStz108	1-Ethoxycarbonyl-β-Carboline	Alkaloids	–	down	down
Zmbp002538	1-Methoxy-indole-3-acetamide*	Alkaloids	down	–	up
MWStz282	3-Hydroxy-3-acetonyloxindole*	Alkaloids	up	–	–
pmb0819	3-Indoleacetonitrile	Alkaloids	down	–	up
Hmmp001310	3-Indoleacrylic acid*	Alkaloids	down	–	up
pme2244	3-Indolepropionic acid	Alkaloids	up	up	up
pmc0682	4-Aminoindole	Alkaloids	down	–	–
mws0597	5-Hydroxyindole-3-acetic acid	Alkaloids	down	up	–
pme2836	5-Hydroxytryptophol	Alkaloids	–	down	–
mws0333	5-Methoxytryptamine	Alkaloids	down	down	down
Lmyn002540	Dioxindole-3-acetyl-3-O-glucoside	Alkaloids	down	–	–
pmb1096	Indole	Alkaloids	down	–	up
pme1651	Indole-3-acetic acid (IAA)	Alkaloids	–	up	up
mws0103	Indole-3-carboxaldehyde	Alkaloids	up	up	–
mws1417	Indole-3-carboxylic acid*	Alkaloids	up	down	–
mws0102	Indole-5-carboxylic acid*	Alkaloids	up	down	–
pmb0818	Methoxyindoleacetic acid	Alkaloids	down	–	up
Hmyp002656	Methyl dioxindole-3-acetate	Alkaloids	down	–	–
mws0677	N-Acetyl-5-hydroxytryptamine	Alkaloids	down	–	–
mws0620	N-Methyltryptamine	Alkaloids	down	–	–
mws0005	Tryptamine	Alkaloids	–	–	up
Qmjp080407	14(15)-Bisnor-13-oxolabd-8(17),11(*E*)-dien-19-oic acid	Terpenoids	up	down	–
Qmjp080402	15,16-Bisnor-13-oxo-8(17),11-labdadien-19-ol	Terpenoids	–	down	down
Wdzp007419	1β-hydroxy-β-cyperone	Terpenoids	up	down	down
Hmcn000773	6’-O-Glucosylaucubin	Terpenoids	–	–	–
Zjyp102922	Humula-3(12),7(13),9(E)-triene-2,6-diol	Terpenoids	up	up	up
MWSmce096	Nootkatone	Terpenoids	–	–	down
Zbbn002910	6”-O-sinapoyl-7-O-caffeoyl-geniposide	Terpenoids	up	–	–
pmp001070	6”-O-Trans-Sinapoylgenipin gentiobioside	Terpenoids	–	–	–
Wbmn003453	6-O-Vanilloylajugol	Terpenoids	–	–	–
Hmmn003964	7-Deoxyloganic acid	Terpenoids	down	–	–
Sacn001940	8-Epiloganic acid	Terpenoids	down	–	–
pmn001587	Asperulosidic acid	Terpenoids	–	–	up
mws1562	Catalpol	Terpenoids	up	up	up
pmn001586	Deacetylasperulosidic acid	Terpenoids	down	–	–
MWSmce434	Gardenoside	Terpenoids	–	up	up
Lmdn001501	Gardoside*	Terpenoids	down	–	–
MWSmce115	Genipin	Terpenoids	up	down	down
Lhjp111633	Glucosylasperuloside	Terpenoids	–	up	–
pmp000691	Loganetin	Terpenoids	–	–	–
Zbyn002745	Loganic acid	Terpenoids	down	–	–
Lmjp004816	Loliolide	Terpenoids	–	–	–
pmn001585	Monotropein	Terpenoids	down	–	–
Cmrn001591	Mussaenosidic acid	Terpenoids	down	–	–
Cmhp003670	Secologanin	Terpenoids	–	up	–
pmp001053	Shanzhiside	Terpenoids	down	–	–
mws1526	Sweroside	Terpenoids	down	up	up
Lmpn004676	Verminoside*	Terpenoids	up	–	–
mws1429	Vomifoliol (Blumenol A)	Terpenoids	up	–	–
Labn003976	Vomifoliol 9-[Xylosyl-(1 → 6)-Glucoside]	Terpenoids	up	–	–
mws1515	α-Ionone	Terpenoids	up	–	–
Cmmn012461	Dehydroabietic acid	Terpenoids	down	down	down
MWSslk208	Kaurenoic Acid*	Terpenoids	up	–	–
Wbmn011269	Vitexilactone	Terpenoids	–	down	down
Llhp011701	11,12-epoxy-13-hydroxy-3-Oxooleanane-28-oic acid gamma-lactone (Liquidambaric Lactone)	Terpenoids	up	–	up
pmn001427	16,23:16,30-Diepoxydammar-24-ene-3,20-diol (Jujubogenin)*	Terpenoids	–	up	–
Lskp211493	2,19-Dihydroxy-3-oxo-24-norolean-12-en-28-oic acid	Terpenoids	up	–	–
pmn001426	2,3,19-trihydroxyurs-12-en-28-oic acid (Euscaphic acid)*	Terpenoids	up	up	–
MWSmce394	2,3,19-Trihydroxyurs-12-en-28-oic acid (Tormentic acid)*	Terpenoids	–	up	up
Smpn009230	2,3,23-Trihydroxyolean-12-en-28-oic acid*	Terpenoids	up	up	up
Lmqp008286	2,3,23-Trihydroxyolean-12-en-28-oic acid (Arjunolic acid)	Terpenoids	up	–	–
Hmjn003948	2,3,6-Trihydroxyurs-12-en-28-oic acid (Madasiatic acid)*	Terpenoids	up	up	up
Lmzn106284	2,3-Dihydroxylup-20(29)-en-28-oic acid (Alphitolic acid)*	Terpenoids	–	up	up
pmn001706	2,3-Dihydroxyolean-12-en-28-oic acid (2-Hydroxyoleanolic acid)*	Terpenoids	–	up	up
Hmjn008136	2,3-Dihydroxyoleana-11,13(18)-dien-28-oic acid (Camaldulenic acid)	Terpenoids	up	up	–
Lmsn012627	2,3-Dihydroxyurs-12,18-dien-28-oic acid	Terpenoids	up	up	–
Zmpn008194	2,3-Dihydroxyurs-12-en-28-oic acid (Corosolic acid)*	Terpenoids	–	up	up
Li512114	2,3-Dihydroxyurs-12-en-28-oic acid methyl ester (Corosolic acid methyl ester)	Terpenoids	–	–	up
mws1610	2,3-Dihydroxyurs-12-en-29-oic acid (Maslinic acid)*	Terpenoids	–	up	up
Lssp210085	3,11-dioxo-β-oleorene	Terpenoids	–	up	up
pmn001591	3,19,23-Trihydroxyurs-12-en-28-oic acid (Rutundic acid)	Terpenoids	–	up	–
Lhnp110101	3,20-Dihydroxyurs-21-en-28-oic acid (Oleanderic acid)	Terpenoids	–	up	up
Hmbn005207	3,23-Dihydroxyolean-12-en-28-oic acid (Hederagenin)*	Terpenoids	–	–	up
MWSHC20176	3-Hydroxylup-20(29)-en-28-oic acid (Betulinic acid)	Terpenoids	–	up	–
mws1389	3-Hydroxyolean-12-en-28-oic acid (Oleanolic acid)*	Terpenoids	–	up	up
Lmsp102910	3-Hydroxyolean-12-ene-27,28-dioic acid (Cincholic acid)	Terpenoids	up	–	–
Lmmn009550	3-Oxo-9,19-cyclolanost-24-en-26-oic acid (Mangiferonic acid)*	Terpenoids	up	–	–
Ymjm000099	3-Oxooleana-11,13(18)-dien-28-oic acid	Terpenoids	up	up	up
Lmhn011365	Morolic acid*	Terpenoids	up	up	up
Lmqn012798	Urs-12(13)-en-3-one-28-oic acid*	Terpenoids	up	up	up
Lmxn001694	6”-O-β-D-Glucosyl-8-*O*-acetylharpagide	Terpenoids	up	–	up
Zadn003162	Ajugoside	Terpenoids	–	–	up
Smhn002274	Blumenol C glucoside; Byzantionoside B*	Terpenoids	down	–	–
Zadp003164	Dehydrololiolide	Terpenoids	–	–	–
pmn001381	Eucommioside	Terpenoids	up	–	–
Zmdn003368	eucomoside A	Terpenoids	–	up	up
Zmdn004697	eucomoside B	Terpenoids	up	–	–
Zmdn004845	eucomoside C	Terpenoids	up	up	–
Lmlp002205	Isololiolide	Terpenoids	–	–	–
Rfmb320	1-Methylpiperidine-2-carboxylic acid*	Organic acids	up	up	up
Lcsn006884	2,2′-(3-methylcyclohexane-1,1-diyl)diacetic acid	Organic acids	–	–	–
Wmzn000227	2,2-Dimethylsuccinic acid	Organic acids	down	down	down
pme0278	2,6-Diaminooimelic acid	Organic acids	down	–	–
Wccp000476	2-[(1*R*)-1-carboxyethoxy]propanoic acid	Organic acids	–	–	–
Lmbn001609	2-Acetyl-2-Hydroxybutanoic Acid	Organic acids	up	–	–
Zbqp000579	2-amino-3-(1H-pyrazol-1-yl)propanoic acid	Organic acids	down	up	up
mws0236	2-Aminoethanesulfonic acid	Organic acids	–	up	–
pme3017	2-Aminoisobutyric acid*	Organic acids	–	down	down
Lmbn001288	2-Hydroxy-2-methyl-3-oxobutanoic acid	Organic acids	up	–	–
Lmrn002746	2-Hydroxy-4-methylpentanoic acid	Organic acids	–	–	–
Lmmn003323	2-Hydroxyhexadecanoic acid*	Organic acids	up	–	–
mws0341	2-Hydroxyisocaproic acid	Organic acids	up	–	–
Lcsn006335	2-Hydroxymyristic acid	Organic acids	up	–	–
Zmyn002323	2-Hydroxyphenylacetic acid	Organic acids	up	–	–
mws0924	2-Methylglutaric acid*	Organic acids	up	–	–
mws0473	2-Methylsuccinic acid*	Organic acids	–	–	–
pme1216	2-Picolinic acid	Organic acids	up	–	up
Lmbn002072	2-Propylsuccinic acid*	Organic acids	up	–	–
MWS5147	3-(Methylthio)Propionic Acid	Organic acids	–	–	down
MWSmce362	3-Ethoxy-3-oxopropanoic acid	Organic acids	down	–	–
Hmhn002738	3-Furoic acid	Organic acids	down	–	–
Lmbn001676	3-Hydroxy-3-Methyl-2-Oxopentanoic Acid*	Organic acids	up	up	up
Lmbn000216	3-Methylmalic acid*	Organic acids	down	–	–
Zmtn001464	4,8-Dihydroxyquinoline-2-carboxylic acid	Organic acids	up	–	–
pme0295	4-Acetamidobutyric acid	Organic acids	up	–	–
mws0373	4-Methyl-2-oxovalerate	Organic acids	up	–	–
Lmbn001467	5-Acetamidopentanoic Acid	Organic acids	up	–	–
MWSslk038	6-Acetamidohexanoic acid	Organic acids	down	down	–
pme0274	6-Aminocaproic acid	Organic acids	down	–	up
mws0972	6-Hydroxyhexanoic acid	Organic acids	up	down	down
Lmtn004049	Abscisic acid	Organic acids	up	–	up
mws0208	Adipic Acid*	Organic acids	up	–	–
pme3096	Aminomalonic acid	Organic acids	up	–	–
Lmyp003934	Anacardic acid	Organic acids	up	down	up
mws0237	Azelaic acid	Organic acids	up	–	–
mws0489	Benzoylformic acid	Organic acids	down	down	down
mws0425	Citraconic acid	Organic acids	–	up	–
WaYn000716	Citric Acid diglucoside	Organic acids	down	–	–
Lmmn000806	Dimethylmalonic acid*	Organic acids	–	–	–
MWS0274	DL-3-Phenyllactic acid*	Organic acids	up	up	up
mws0267	DL-Glyceric Acid	Organic acids	–	–	–
MWSmce183	D-Mandelic acid	Organic acids	up	down	down
Wasn001627	Glucosyl 2,3-Dihydroxy-2-Methylbutanoic Acid	Organic acids	–	–	down
Wasn003258	Glucosyl 2-Hydroxy-4-Methylpentanoic Acid	Organic acids	up	–	–
MWS1882	Iminodiacetic acid*	Organic acids	down	up	–
Zmjp003163	Jasmonic acid	Organic acids	up	down	down
MWS0811	L-Pipecolic Acid	Organic acids	–	–	–
Wayn000504	Malic acid-1-O-diglucoside	Organic acids	up	up	up
Lmyn002403	Mandelic acid-β-glucoside	Organic acids	up	–	–
MWS2040	Methanesulfonic acid	Organic acids	up	–	–
MWSmce536	Methyl 2-furoate	Organic acids	–	–	–
pme0220	Methyl jasmonate	Organic acids	up	–	–
mws0470	Methylmalonic acid*	Organic acids	up	–	–
MWS5136	Mono-Methyl Glutarate*	Organic acids	up	up	up
Lmmn002164	Monomethyl succinate*	Organic acids	–	–	–
Lmyn000160	Mucic acid Dimethyl Ester	Organic acids	–	–	–
mws1167	Oxalacetic acid	Organic acids	up	–	up
mws0159	Phenylpyruvic acid	Organic acids	up	–	–
mws2125	Phosphoenolpyruvate	Organic acids	–	up	down
Zmjn001813	Pimelic acid*	Organic acids	up	–	–
mws0242	Suberic Acid	Organic acids	up	–	–
mws0192	Succinic acid*	Organic acids	up	–	–
Lmgn000219	Succinic semialdehyde	Organic acids	up	–	–
pme2380	α-Ketoglutaric acid	Organic acids	up	–	–
mws0147	β-Hydroxyisovaleric acid	Organic acids	up	–	–
Wcdp010162	1-Monolinolenoyl-Rac-Glycerol*	Lipids	–	down	down
pmb0296	1-Oleoyl-Sn-Glycerol	Lipids	–	down	–
Zbfn008434	1-O-Linoleoyl-3-O-galactopyranosyl-L-glycerol	Lipids	down	down	–
Wagn011658	1-Palmitoyl-Sn-Glycerol 3-O-Diglucoside	Lipids	down	down	–
pmb1562	1-Stearidonoyl-Glycerol	Lipids	down	down	–
Lmhp011562	1-α-Linolenoyl-glycerol*	Lipids	–	down	down
Lmhp009773	1-α-Linolenoyl-glycerol-3-O-glucoside*	Lipids	–	–	–
Wagn012030	2-Palmitoyl-Sn-Glycerol 3-O-Diglucoside	Lipids	–	down	–
Lmhp011388	2-α-Linolenoyl-glycerol*	Lipids	–	down	down
Lmhp008513	2-α-Linolenoyl-glycerol-1,3-di-O-glucoside	Lipids	down	down	–
Lmhp009526	2-α-Linolenoyl-glycerol-1-O-glucoside*	Lipids	–	–	–
Lmfp009701	Glycerol 9(E),11(Z),13(*E*)-octadecatrienoyl ester*	Lipids	–	down	down
Walp013004	Glycidyl Linoleate	Lipids	–	down	down
WaYn012231	LPG(18:2(9Z,12Z)/0:0)	Lipids	down	down	down
Hmyn007168	LysoPG 16:0	Lipids	down	down	down
Sazp010264	Monogalactosyldiacylglycerol	Lipids	–	down	down
Lmsp010763	Monolinolenin*	Lipids	–	down	down
Hmyp007792	PE(oxo-11:0/16:0)	Lipids	down	up	up
Lmqn008024	PS(18:2)	Lipids	down	down	–
mws0120	Choline Alfoscerate	Lipids	down	–	down
pmb2221	4-Hydroxysphinganine; Phytosphingosine	Lipids	down	up	up
pmd0130	LysoPC 14:0	Lipids	–	down	up
pmb2319	LysoPC 15:0*	Lipids	up	down	–
Lmhp009129	LysoPC 15:0(2n isomer)*	Lipids	–	down	–
pmb2260	LysoPC 15:1	Lipids	up	down	–
pmb0855	LysoPC 16:0	Lipids	–	down	–
pmd0132	LysoPC 16:0(2n isomer)	Lipids	–	down	–
pmp001270	LysoPC 16:1*	Lipids	up	down	up
Lmhp008833	LysoPC 16:1(2n isomer)*	Lipids	–	down	–
pmb0863	LysoPC 16:2(2n isomer)	Lipids	–	down	up
pmb2406	LysoPC 17:0*	Lipids	–	down	–
Lmhp010515	LysoPC 17:0(2n isomer)*	Lipids	up	down	up
Lmhp009590	LysoPC 17:1	Lipids	–	down	up
Lmhp008718	LysoPC 17:2	Lipids	up	down	up
mws0126	LysoPC 18:0	Lipids	–	down	–
pmd0136	LysoPC 18:0(2n isomer)	Lipids	–	down	–
Lmhp010190	LysoPC 18:1(2n isomer)	Lipids	–	down	–
pmp001251	LysoPC 18:2(2n isomer)	Lipids	–	down	up
pmb0865	LysoPC 18:3(2n isomer)	Lipids	–	down	–
Hmqp006235	LysoPC 18:4	Lipids	–	down	up
pmb2228	LysoPC 19:0	Lipids	–	down	–
Lmhp010908	LysoPC 19:1	Lipids	–	down	–
Lmhp007598	LysoPC 19:2(2n isomer)	Lipids	up	down	–
Lmhp011549	LysoPC 20:1	Lipids	–	down	–
pmd0147	LysoPC 20:2*	Lipids	–	down	–
pmd0146	LysoPC 20:2(2n isomer)*	Lipids	–	down	–
Lmhp009890	LysoPC 20:3	Lipids	–	down	–
pmb0864	LysoPE 14:0*	Lipids	down	–	up
Lmhp008337	LysoPE 14:0(2n isomer)*	Lipids	down	–	up
Lmhp009187	LysoPE 15:0	Lipids	down	down	up
Lmhp008885	LysoPE 15:0(2n isomer)	Lipids	down	–	up
Lmhp008440	LysoPE 15:1	Lipids	down	–	up
pmb0876	LysoPE 16:0	Lipids	down	down	up
pmd0160	LysoPE 16:0(2n isomer)	Lipids	down	down	up
Lmhp009034	LysoPE 16:1*	Lipids	down	down	–
Lmhp008763	LysoPE 16:1(2n isomer)*	Lipids	down	down	–
Lmhp010162	LysoPE 17:0	Lipids	down	–	up
Lmhp009769	LysoPE 17:1*	Lipids	down	down	up
Lmhp009464	LysoPE 17:1(2n isomer)*	Lipids	down	down	up
pmb0883	LysoPE 18:0	Lipids	down	down	up
mws0289	LysoPE 18:1*	Lipids	down	–	up
pmb0856	LysoPE 18:1(2n isomer)*	Lipids	down	down	up
pmb0881	LysoPE 18:2	Lipids	down	–	up
pmb0874	LysoPE 18:2(2n isomer)	Lipids	down	–	up
Lmhp008801	LysoPE 18:3	Lipids	down	–	up
Lmhp008589	LysoPE 18:3(2n isomer)	Lipids	down	–	up
Lmhp008233	LysoPE 18:4	Lipids	down	–	up
Lmhp010040	LysoPE 20:3*	Lipids	down	down	up
Lmhp009802	LysoPE 20:3(2n isomer)*	Lipids	down	–	up
pmn001686	10,16-Dihydroxypalmitic acid	Lipids	up	up	up
mws2623	11-Octadecanoic acid(Vaccenic acid)*	Lipids	–	down	down
Zmyn004548	12-Oxo-phytodienoic acid	Lipids	–	down	down
Lmbn005369	13(*S*)-HODE;13(*S*)-Hydroxyoctadeca-9Z,11E-dienoic acid*	Lipids	–	down	down
Zmzn003953	13(s)-hydroperoxy-(9z,11e,15z)-octadecatrienoic acid	Lipids	up	up	up
MWS80007	13-Hydroperoxy-9Z,11E-octadecadienoic acid*	Lipids	up	up	up
Lmbn005443	13-KODE; (9Z,11E)-13-Oxooctadeca-9,11-dienoic acid*	Lipids	up	down	–
MWS2430	13-methylmyristic acid	Lipids	up	up	up
pmb2804	13S-Hydroperoxy-9Z,11E-octadecadienoic acid	Lipids	–	down	down
Zmyn004676	17-Hydroxylinolenic acid	Lipids	up	down	down
Zmyn005384	2R-Hydroxyoctadecanoic Acid*	Lipids	up	–	–
Zmyn005252	3-Hydroxy-palmitic acid methyl ester	Lipids	up	–	–
Wcdp006929	4-Oxo-9,11,13,15-Octadecatetraenoic Acid	Lipids	up	down	–
pmb0885	4-Oxo-9Z,11Z,13E,15E-Octadecatetraenoic Acid	Lipids	up	down	–
MWS2673	5,6-DiHETrE[(±)5,6-dihydroxy-8Z,11Z,14Z-eicosatrienoic acid]	Lipids	–	down	–
Lmbn005287	7S,8S-DiHODE; (9Z,12Z)-(7*S*,8*S*)-Dihydroxyoctadeca-9,12-dienoic acid*	Lipids	up	up	up
Lmbn005662	9(10)-EpOME;(9*R*,10*S*)-(12Z)-9,10-Epoxyoctadecenoic acid	Lipids	up	down	down
pmn001694	9,10,13-Trihydroxy-11-Octadecenoic Acid	Lipids	up	down	–
Lmbn004240	9,10-Dihydroxy-12,13-epoxyoctadecanoic acid	Lipids	up	–	up
Lmbn003970	9,12,13-TriHOME; 9(*S*),12(*S*),13(*S*)-Trihydroxy-10(E)-octadecenoic acid	Lipids	up	down	–
pmn001691	9,12,13-Trihydroxy-10,15-octadecadienoic acid	Lipids	up	down	–
pmb2791	9-Hydroperoxy-10E,12,15Z-octadecatrienoic acid	Lipids	up	down	down
pmb2786	9-Hydroxy-10,12,15-octadecatrienoic acid*	Lipids	up	down	–
Zmyn004449	9-Hydroxy-12-oxo-10(E),15(Z)-octadecadienoic acid	Lipids	–	down	down
pmn001689	9-Hydroxy-12-oxo-15(Z)-octadecenoic acid*	Lipids	up	down	–
Rfmb087	9-Hydroxy-13-oxo-10-octadecenoic Acid	Lipids	up	–	–
WaYn010298	9-Hydroxyoctadeca-6,10,12,15-Tetraenoic Acid	Lipids	up	–	down
Wcdp007645	9-Oxo-10,12-Octadecadienoic Acid	Lipids	up	down	down
pmb2787	9-Oxo-10E,12Z-octadecadienoic acid	Lipids	up	down	down
Zmjn004133	9S-Hydroperoxy-10E,12Z-octadecadienoic acid	Lipids	–	–	–
Rfmb091	9S-Hydroxy-10E,12Z-octadecadienoic acid*	Lipids	–	down	down
Wafn011571	alpha-Hydroxylinoleic acid*	Lipids	–	down	down
Zbfn013528	Beta-Hydroxypalmitic Acid*	Lipids	up	–	–
Lmbn005923	Crepenynic acid	Lipids	–	down	–
MWS4295	DL-2-hydroxystearic acid*	Lipids	up	–	–
mws0396	Elaidic Acid*	Lipids	–	down	down
MWSslk132	Hexadecanedioic acid	Lipids	up	–	–
Zadn009075	Hydroperoxylinoleic acid*	Lipids	up	down	–
Yalp008047	Methyl 12-phenyldodecanoate	Lipids	–	down	down
Lmyn012331	Petroselinic acid*	Lipids	–	down	down
mws0367	α-Linolenic Acid*	Lipids	–	down	down
mws0366	γ-Linolenic Acid*	Lipids	–	down	down

XY as the core group and comparing it one-to-one with the other three samples, their nonvolatile metabolites formed a Venn diagram (Fig. 4D), with both common and unique metabolites between the different control groups. The result revealed those were 266 overlapping significantly differential metabolites among the three pairwise comparisons. These 266 metabolites are considered key metabolites that might be differently regulated during each processing treatment. Compared to the other two groups, there were 79 metabolites specific to HG vs. XY. And 144 metabolites specific to the GT vs. XY group, and a high number of 439 metabolites specific to BT vs. XY. It is illustrated again that the BT treatment makes a great impact on the non-volatile metabolites in EUL.

#### 3.3.1 Amino acids and their derivatives

Amino acids are often associated with aroma, flavor and freshness. Methionine, valine, lysine and threonine, etc. are necessary for human body. Moreover, many amino acids are the flavor enhancers.

Among the processing methods, the HG is most conducive to producing the amino acid content beneficial for the human body in EUL leaves, with the GT process ranking in second. In the heatmap the amino acid and their derivatives (Fig. S3A), the relative content varies from red to green, with the HG group exhibiting a concentration of red, while the BT group shows a concentration of dark green.

Based on data from Table S3, the BT still caused a substantial decrease in the amino acid content of fresh EUL. Most of the down-regulated amino acids were related to bitterness, which may indicate that BT processing can reduce bitterness and astringency. The reason might be that amino acids undergo deamination, decarboxylation, and Maillard reaction during withering to produce various alcohols and aldehydes as aroma substances(Jin-jie et al., 2013). In addition, during fermentation, most of the amino acids provide nitrogen and carbon sources for microorganisms, and combine with tea polyphenols and sugars to form quinones, aldehydes, acids, alcohols, pigments and other substances under enzymatic action, thus the amino acids are down-regulated(Shao et al., 2022).

In GT, there were more up-regulated metabolites than down-regulated amino acid and derivatives, and mostly small-molecule peptides in the up-regulated substances. Especially, cyclosmall peptides with proline, which is heat stable, should be the marker for GT. This association could be attributed to the diminished activity of hydrolytic enzymes during the green tea processing (Yiding et al., 2014). This results in a decrease in the ability to convert substances such as proteins and polypeptides into free amino acids, ultimately resulting in an increased formation of small molecule peptides.

In HG, the random degraded peptides with 2 to 3 amino acid residues are the main up-regulated metabolites, and the fructosyl amino acid which is probably produced by heat stress should be recognized as the marker, for N-(1-deoxy-1-fructosyl) leucine and N-(1-deoxy-1-fructosyl) valine upregulated for over 60 folds. This can indicate that EUL HG process than the GT process makes them show different variation obviously. Furthermore, some important amino acids including L-tryptophan, L-leucine, *L*-phenylalanine, L-arginine, and L-methionine showed different levels of content increase after HG compared to XY. L-tryptophan, *L*-phenylalanine and L-tyrosine are precursors for natural products such as pigments, indole compounds, alkaloids, phenylpropanoids and hormones(Maeda & Dudareva, 2012).

Conversely, the concentration of glutamic acid and lysine, recognized as flavor enhancers, is substantially higher in XY compared to other samples. In contrast, aspartic acid, another flavor enhanced amino acid, is mainly increased in the BT process. This indicate that BT processed EUL should possess different flavor in contrast to XY significantly.

#### 3.3.2 Lipids

Both glycerides and glycerophospholipids are major lipids for energy substrate accumulation in plant leaves (Kimura et al., 2019). They can be hydrolyzed and oxidized in enzymatic and nonenzymatic way, also can be hydroxylated in enzymatic way. In this research, 144 nonvolatile lipid metabolites were detected belonging to the following five categories: free fatty acids, glycerides, lysoglycerophospholipid, hydroxylated fatty acid and oxidized fatty acid. In BT, almost all of the up-regulated lipids were hydroxylated and oxidized fatty acid, it should contribute to that the fermentative microorganism use lipid as oxidative substrates to obtain energy. In HG, nearly all of the up-regulated lipids were lysoglycerophospholipid, especially lysophosphatidylethanolamine (LPE) and lysophosphatidylcholin (LPC). It should contribute to free fatty acid loss in the thermos pyrolysis of glycerides. In GT, longer/shorter chain fatty acids such as arachidic acid(C20), docosenoic acid(C22), myristic acid(C14) and random unsaturated octadecanoic(C18) were upregulated, it should be contributed to a rapid thermal oxidation.

From the Heat map of lipid classes (Fig. S3B), α-linolenic acid and γ-linolenic acid showed downward adjustments in both the GT process and HG process, and there was no significant change in BT processing. The reason is that each process has different water distribution speed, black tea after withering for a long time. And room temperature water loss is not as fast as removing and drying. Reducing water causes the concentration of lipoxygenase (LPO) to rise, therefore lipids like linolenic acid are converted to volatiles by the enzyme(Dongqing, 2001).

Furthermore, glyceride metabolites were down-regulated after different processes. May be attributed to autoxidation, photo-oxidation and enzymatic oxidation during processing (Shahidi & Hossain, 2022).

#### 3.3.3 Flavonoids

Flavonoids are generally present in EUL in the form of glycosides or carbon sugar groups, but also in free form. Flavonoids are also important coloring substances in tea, for example, quercetin gives tea a green color(Wang et al., 2021).

In this study, the number of significant up- and down-regulation of the three comparison groups, BT vs. XY, GT vs. XY, and HG vs. XY, were 33, 43, 21, and 121, 32, 18, respectively. It is observed that the metabolite content of flavonoids was decreased after the BT process, while the HG treatment had a somewhat lesser effect on the total quantity of flavonoids.

In BT, plenty of dissociated flavonoids and a few flavonoid glycosides increased rapidly, for example, the contents of isorhamnetin, tamarixetin, quercetin, morin, and kaempferol increased by 11.80-, 11.01-, 9.88-, 7.51-, and 6.44-fold, respectively. That is to say, the fermentation microorganisms hydrolyzed a great many of flavonoid glycosides by glycosidase in BT process. Pick quercetin and its glycosides as an example, those associated with dicarbohydrate, rare or modified monosaccharides such as quercetin −3-O-rhamnosyl(1 → 2) arabinoside, quercetin-7-O-rutinoside etc. decreased distinctly, but flavonoids monomer, such as quercetin and naringin, increased evidently in BT process. It might be because fermentation allows glycosides to be degraded by the action of enzymes (Jingyi, M., et al., 2023). Reducing compounds such as catechins and proanthocyanidins A4 are consumed in microbial processes. The fermentation process in turn leads to further conversion of catechins (Dongqing, 2001).

In GT, the apparent change is flavonoid gallate and rare flavonoid glycosides increased obviously. Catechin, epicatechin, gallocatechin, epigallocatechin etc. were all existed in the form associated with gallate. And didymin, poncirin, myricetin, apigenin-7-O-glucoside and isovitexin-4’-O-glucoside, which were not present in XY, increased significantly after processing. The other significant increase is in catechins, the major components of freshness, bitterness and astringency in green tea. After the GT processing, there was a partial down-regulation of chalcone analogs, which are important precursor substances of catechins. This may be the reason for the up-regulation of catechins. The GT processing can also enhance part of the flavonoid content, to play its medicinal properties are still beneficial, but the bitterness and astringency cannot be less like the BT processing.

In HG, there was also a tendency for some catechins and homologs to be significantly up-regulated. However, the multiplicity was not as high as after the GT processing and the existed form of catechins were polymer or modified glycosides, for example, gallocatechin-(4α → 8)-catechin and 3’-O-Methyl-epicatechin. But in general,

Overall, HG is the lest different process from fresh leaves. And the BT treatments cause the flavonoids to drop even more, where the withering treatment is crucial for reducing the bitter and astringent flavonoids in the tea. Also, after prolonged withering and fermentation, the ester catechins will decrease and the enzyme activity will be similarly inhibited(Shao et al., 2022).

#### 3.3.4 Phenolic acids

**Supplementary fig S4:**
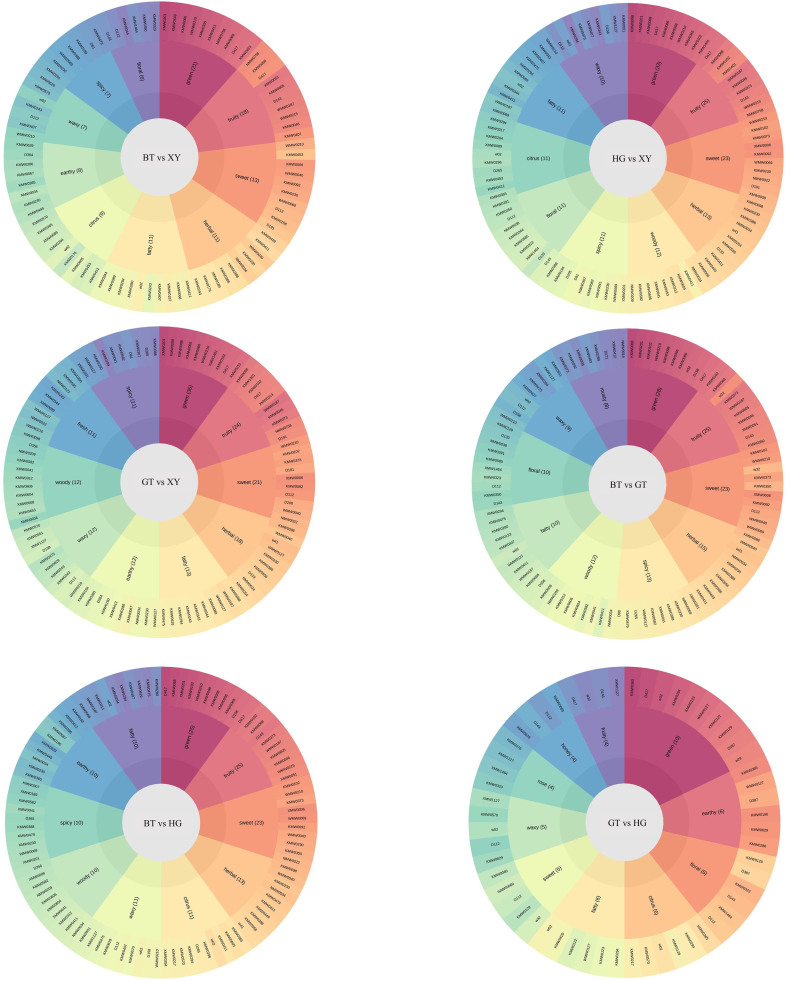
Radar plot of sensory flavor characterization of differential metabolites across comparison groups; Note: The outermost circle name indicates the organoleptic flavor profile, and the number corresponding to the green dot indicates the number of occurrences of the corresponding organoleptic flavor profile, i.e., the number of differential metabolites annotated to that organoleptic flavor profile.

The phenolic acid active substances in EUL are mainly composed of chlorogenic acid and their related metabolites (isochlorogenic acid, chlorogenic acid A/B/C, 1-caffeoyl- quinic acid, etc.) and gallic acid (GA) and its associated compounds, caffeic acid and its homologs and derivatives (caffeic aldehyde, etc.) and quinic acid derivatives. (hydroquinone, Koaburaside etc.). In the heat map of phenolic acids(Fig.S4D) in this study, both dark red and dark green colors are mostly concentrated in the BT, indicating significant up- and down-regulation changes. and is significantly different from the other three samples.

Chlorogenic acid, cryptochlorogenic acid and neochlorogenic acid were significantly down-regulated by 0.36-, 0.43- and 0.23- fold only after the BT processing, while isochlorogenic acid A/B/C was up-regulated by about 2-fold only after the GT processing. Phenolic compounds originate from the biosynthetic pathways of shikimic acid, phenylpropanoid, and flavonoids(Tohge et al., 2017). Chlorogenic acid was down-regulated after the BT processing, while chlorogenic acid did not change much after the GT and HG processing. This is similar to previous studies because of the fermentation stage of black tea processing and the gradual reduction of enzymes(Sha et al., 2008). In another words, in BT processing microorganism utilized the reducing force of chlorogenic acid and promote the biosynthesis of other pre-chlorogenic acid metabolites. And HG/GT processing maintain the concentration of chlorogenic acid, but the higher temperature of GT processing promotes the production of isochlorogenic acid by additioning of another caffeic acid to the 1′,3′ or 5′ hydroxyl of quinic acid.

Gallic acid (GA) and its associated compounds should also be markers of GT processing EUL. Gallic acid, digallic acid, trigallic acid and other gallate associated carbohadrate or modified by methyl were all increased uniquely in GT than other processing EUL. So, GT processing would bring more astringency for this feature.

Caffeic acid and its homologs and derivatives are tightly related to GT processing. And caffeic acid has good hepatoprotective, anti-diabetic and anti-tumor effects (Mirzaei et al., 2021). Almost all the simple caffeol modified compounds were increased in GT, such as caffeic acid, 1-caffeoylquinic acid, 3-O-caffeoylshikimic acid, dihydrocaffeoylglucose etc.

Quinic acid derivatives widely existed in XY, GT and HG, but only a few derivatives existed in BT. But, it is interesting that the etherifying quinic acid glucoside which is improved to be a potential antiviral and anti-inflammatory activity were determined so high in BT processing, and hydroquinone increased obviously in GT and HG which maybe cauesd by high temperature stimulation on quinic acid.

In BT, metabolites increased also include O-anisic acid, gentisic acid, ferulic acid, isoferulic acid, p-coumaric acid, hydroxycinnamic acid, and sinapyl alcohol. Those are all functional substances which should contribute to fermentation microrganism.

In HG, cinnamic acid, desmethylpinobanksin, 2-methylbenzoic acid, and 3-methylsalicylic acid were all up-regulated by 5-folds or more. Cinnamic acid improves glucose tolerance in the body and stimulates insulin secretion outside the body to achieve anti-diabetic effect (Hafizur et al., 2015). Cinnamaldehyde has the potential to be converted into cinnamyl alcohol and methyl cinnamate and cinnamic acid in the body, from the heatmap (Fig.S4D) this is the reason why the fresh leaves contain more cinnamyl alcohol than other processed samples.

#### 3.3.5 Nucleotides and derivatives

A total of 69 significantly differentiated metabolites of nucleotides and their derivatives were screened. According to the three comparative groups of BT vs. XY, GT vs. XY and HG vs. XY, the highest number of up-regulation was found in HG vs. XY, followed by GT vs. XY and BT vs. XY. The highest number of down-regulation was found in the group of BT vs. XY. It can be seen that the HG treatment improves the content of nucleotides and their derivatives better.

Common nucleotide and those compounds can be used as reducing force such as NAD and NADP were all found to increase in fresh EUL. HG processing boosted the production of most nucleotides and derivatives include the common nucleotides, such as adenosine(A), guanosine(G), thymine(T), cytidine(C) and their modified molecules. These should contribute to the thermo-decomposition of nuclear acid. BT processing promoted the production of 1,7-dimethylxanthine, 3-methylxanthine, which are the precursor of caffeine. And also, their xanthine, a purine degradation intermediate, should be picked as the typic metabolite of BT processing. Then, GT processing promoted the production of methyl modification nucleotides and especially stimulated the accumulation of inosine 5′-monophosphate (IMP), a flavor enhancer which would endow the flavor of GT auxiliary.

#### 3.3.6 Alkaloids

Alkaloids, as a natural ingredient, can achieves significant anti-hepatocellular carcinogenic effects. It is reached using a variety of mechanisms to inhibit proliferation, such as metastasis and angiogenesis, to promote apoptosis and autophagy, and to regulate various cancer-related genes and pathways, etc.(Liu et al., 2019). From the comparison of the number of significant up- and down-regulations in these three groups (BT vs. XY, GT vs. XY, and HG vs. XY), HG vs. XY was the most up-regulated and BT vs. XY was the most down-regulated. The variated alkaloids in EUL were mainly composed of amines, amides, quinoline and derivatives, indoles, quinolinic acid and nuclear bases derivatives. Based on the heatmap of the alkaloid classes (Fig.S3F), there was less red color lumps in the XY sample, and there were significant red color lumps in BT, GT, and HG. This suggests any one of the treatments would have a significant effect on the content of the different alkaloids.

BT processing promoted the production of caffeine, quinine, indole carboxylic acid and Octadec-2-enamide. The content of metabolites such as indole ketone, quinine, and quinolinic acid in BT showed a significant increasing trend (Fig.S3F), whereas salicylamide, benzamide, and cyclohexylamine showed a significant decreasing trend. Notably, caffeine was up-regulated 120-fold after BT/GT processing. This indicates that the BT processing stimulated the increase in caffeine content more than the other two processes. Quinolinic acid was up-regulated in black tea processing only and not in the other two processes.

The HG processing promoted the idolization, hydroxylation and carbonitridation, more than 20 metabolites such as indole, 8-hydroxyquinoline, isoquinoline, and 3-Indoleacetonitrile, 4-Hydroxymandelonitrile were significantly more abundant than other samples.

GT processing should be marked by the production of active polyamines (PAs). Spermine, spermidine, agmatine, caffeoylagmatine, stearamide and dopamine etc. were increased only in GT processing EUL. PAs are not only one of the plant growth regulators, but also the essential metabolites to tumor cell cycle. Recent data indicate that polyamines can play a major role in regulating the anti-tumor immune response, thus likely contributing to the existence of immunologically ‘cold’ tumors that do not respond to immune checkpoint blockade (Holbert et al., 2022).

#### 3.3.7 Organic acids

Organic acids are not only key intermediates for decomposing carbon compounds, but also coordinate flavors, providing acidity and fruit flavors. Since the rankings of the number of upward and downward adjustments in the organic acids category were BT vs. XY, HG vs. XY and GT vs. XY. In addition, from the organic acids heatmap (Fig. S3G), it could be seen that the red color lumps concentrated in the BT sample, meaning that BT processing still favors the enhancement of organic acids.

Firstly, methyl jasmonate was up-regulated up to 89-fold in the BT group and was investigated as a substance with a floral aroma. It has also been shown to be a phytohormone. (Besson et al., 2018).

Secondly, in GT, the up-regulation multiplier is not as high as the up-regulation multiplier of organic acids after the BT processing. Finally, from the organic acids heatmap (Fig. S3G), it could be seen that the red color lumps concentrated in the BT sample, meaning that the BT processing favors the production of organic acids.

#### 3.3.8 Lignans

Lignans are one of the most researched components of EUL. The active ingredients in *Eucommia* lignans are mainly pinosylvin diglucoside and butyrophilin diglucoside. However, neither of these two substances were among the significantly different metabolites in the BT vs. XY, GT vs. XY, and HG vs. XY groups. Among the three groups, the highest number of upregulations was found in BT vs. XY; and the lowest number of downregulations was found in GT vs. XY. Finally, judging from the lignin Heatmap (Fig. S3H), the red color is concentrated in the BT sample, so it is clear that the EUL leaves after the BT processing are the highest in lignin content.

Pinoresinol, epipinoresinol, eucommin A, and buddlenol B/E/F, which amounts were enhanced just in the processing of BT, should be identified as the marker of lignans of EUL. Pinoresinol and epipinoresinol is the residue of pinosylvin diglucoside, and had been proved to be the effective antihypertensive drug by recent research. Otherwise, pinoresinol-4,4’-O-diglucoside and secoisolariciresinol diglucoside existed in GT processing, which had been identified to anti hypertension.

#### 3.3.9 Tannin

There were 12 non-volatile metabolites tannins in the four samples tested, 9 (8 down-regulated) of them were significantly different metabolites. And the differential metabolites were only present in the BT vs. XY group. It contains proanthocyanidins, which are condensed tannins and are the end products of the flavonoid biosynthesis pathway. It is recognized for its strong antioxidant activity, in addition to anticancer, anti-glycemic and neuroprotective effects(Rauf et al., 2019). In addition, the heatmap (Fig. S3I) showed that the proanthocyanidins in fresh EUL were relatively abundant, especially in GT processing and it would further decompose after BT processing. The proanthocyanidins content of GT only raised a bit higher than XY and was differed from that of HG obviously, but all the three samples contained similar content of tannin.

#### 3.3.10 Terpenes

Terpenes are generally derived from mevalonate, and come into being the hot area in *E. ulmoides*, many terpenes, especially 5 new iridoid molecules were identified and analyzed in recent years (Lv et al., 2023; Takamura et al., 2007).

105 terpene nonvolatile metabolites were detected in this study. Among them, 71 were significantly different in the three comparison groups of BT vs. XY, GT vs. XY, and HG vs. XY. With BT vs. XY being the group with the highest number of up- and down-regulations. In general, BT, GT and HG treatments raised the terpene content. In the Heatmap of terpenes (Fig. S3J), it can be seen that the red color of the BT samples is more concentrated, further illustrating the benefits of the BT processing on the rise of terpene content.

It is clear that BT processing has a beneficial effect on some free terpene rise. EUL leaves have important terpene actives, especially iridoid and its derivatives, such as geniposide, geniposidic acid, etc. Almost all of them got increase in BT processing, while verminoside, kaurenoic acid were also raised. But it is worth to point out that the aucubin, geniposide, genipenic acid and gardoside down-regulated obviously in BT processing. These should contribute to glycoside shedding caused by fermentation microrganis. Conversely, GT and HG processing enhance the accumulation of aucubin, geniposide, genipenic acid and gardoside.

#### 3.3.11 Sugar (carbohydrates)

BT processing also increased the acidification of monocarbohydrate and some nonreduced carbohydrate, for example, concentration of D-glucoronic acid, D-galacturonic acid, D-xylonic acid raised obviously and raffinose, fucose, xylitol, fructose and rhamnose were much higher than XY and other treatments. That is to say, BT EUL would possess a special sweet taste.

GT processing raised the concentration of sucrose, manitol, dulcitol etc. and promoted the carbohydrate phosphorylation, for instance, contents of d-fructose 6-phosphate, d-glucose 1,6-bisphosphate, d-glucose 6-phosphate, d-glucose 1-phosphate were all higher than other processing.

### 3.4 Volatile metabolite profiles and dynamics of XY, HG, RT, GT

**Fig. 5:**
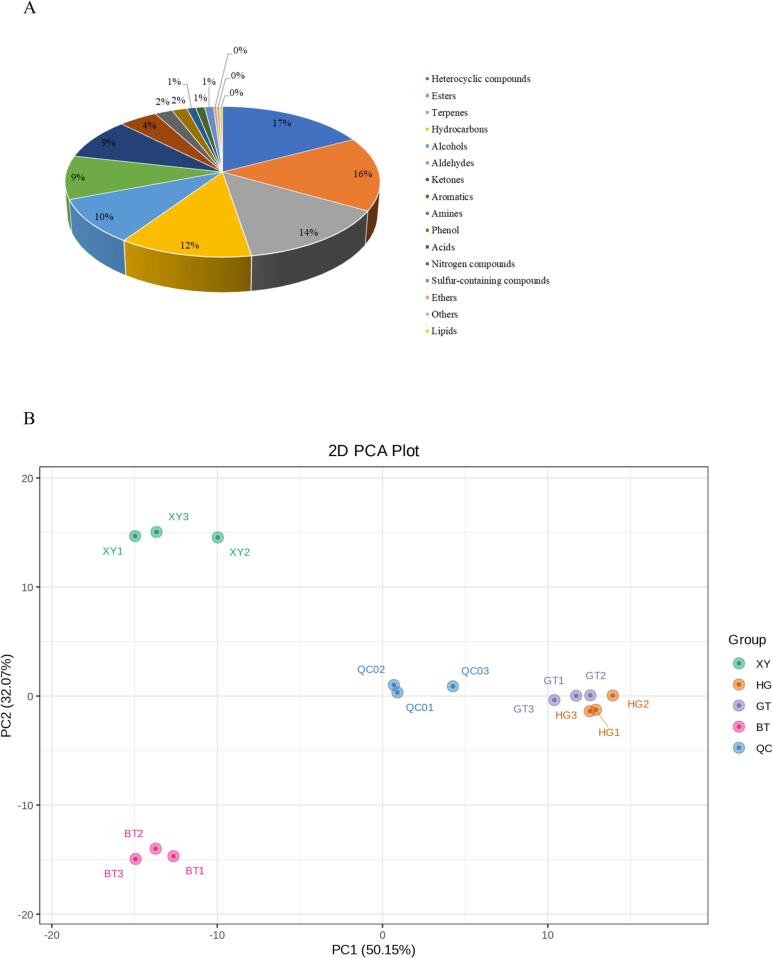
Pie chart of volatile metabolites(PC1 = 50.15 %,PC2 = 32.07 %); 5B PCA score of volatile metabolites.

A total of 289 (Table 4) volatile metabolites belonging to 16 classes were found in XY, HG, RT, and GT. According to Fig. 5A, the heterocyclic class had the largest proportion (50), followed by esters (46), terpenoids (41), hydrocarbons (35), alcohols (28), aldehydes (27), ketones (26), aromatics (13), amines (6), phenols (5), acids (3), nitrogen-containing compounds (3), sulfur-containing compounds (3), ethers (1), lipids (1), and other volatile metabolites (1), and other volatile metabolites (1), and other volatile metabolites (1), lipids (1) and other volatile metabolites (1).

**Table 4:** Summary table of volatile metabolite composition, classification and relative content of substances in different processed EUL.

Index	Compounds	Class	Odor
NMW0057	Glutarimide	Amine	–
XMW0711	Formamide, *N*-phenyl-	Amine	–
XMW1471	1-ButanAmine, *N*-methyl-N-2-propenyl-	Amine	–
NMW0013	Benzylamine	Amine	–
WMW0092	1-OctanAmine,*N*-methyl-	Amine	–
XMW0545	Benzenemethanamine, N-(1-methylethyl)-	Amine	–
XMW0735	Benzenemethanol, .alpha.-2-propenyl-	Alcohol	–
WMW0187	2-Hexanol	Alcohol	chemical, winey, fruity, fatty, terpenic, cauliflower
D373	2,4-Heptadien-1-ol, (E,E)-	Alcohol	green, fruity, nutty, cheese
KMW0102	1-Hexanol	Alcohol	ethereal, fusel, oily, fruity, alcoholic, sweet, green
w02	2,4-Decadien-1-ol	Alcohol	fatty, waxy, citrus, melon
KMW0092	2-Furanmethanol	Alcohol	alcoholic, chemical, musty, sweet, caramel, bread, coffee
KMW0323	Benzyl Alcohol	Alcohol	floral, rose, phenol, balsamic
WMW0127	3-Octen-1-ol, (Z)-	Alcohol	fresh, fatty, grassy, melon, green, cortex, herbal, earthy, fusel, spicy
KMW0194	2-Octanol	Alcohol	fresh, spicy, green, woody, herbal, earthy
KMW0355	trans,cis-2,6-Nonadien-1-ol	Alcohol	green, cucumber, oily, violet, leafy
XMW0424	2,6-Dimethyl-1-nonen-3-yn-5-ol	Alcohol	–
KMW0629	1-Dodecanol	Alcohol	earthy, soapy, waxy, fatty, honey, coconut
KMW0344	1-Nonanol	Alcohol	fresh, clean, fatty, floral, rose, orange, dusty, wet, oily
XMW1072	(*E*)-2,6-Dimethylocta-5,7-dien-2-ol	Alcohol	–
KMW0098	3-Hexen-1-ol, (Z)-	Alcohol	fresh, green, grass, foliage, vegetable, herbal, oily
XMW1307	Cyclohexanol, 1-methyl-	Alcohol	–
XMW0642	Cyclohexanol, 3,5-dimethyl-	Alcohol	–
XMW0434	4-Hexen-1-ol, acetate	Alcohol	–
WMW0103	2-Cyclopentylethanol	Alcohol	–
D387	1-Hexanol, 3,5,5-trimethyl-	Alcohol	grassy, green, weedy, floral, earthy, aldehydic, hay, straw, leafy
NMW0022	Benzenemethanol, .alpha.-methyl-	Alcohol	fresh, sweet, gardenia, hyacinth
KMW0276	1-Octanol	Alcohol	intense citrus, rose
XMW1233	3-Cyclopentyl-1-propanol	Alcohol	–
XMW0410	1-Nonen-4-ol	Alcohol	–
KMW0294	2-Octen-1-ol, (E)-	Alcohol	green, citrus, vegetable, fatty
NMW0038	Benzenemethanol, 4-methyl-	Alcohol	mild, floral
KMW0376	2-Nonanol	Alcohol	rose
KMW0588	3-Cyclohexene-1-ethanol, .beta.,4-dimethyl-	Alcohol	fruity, herbal
w08	Naphthalene, 1-methyl-	Aromatics	naphthyl, chemical, medicinal, camphor
XMW0916	(5-bromopentyl)-Benzene	Aromatics	–
KMW0070	Benzene, 1,3-dimethyl-	Aromatics	plastic
XMW0659	Benzene, n-butyl-	Aromatics	–
KMW0060	Toluene	Aromatics	sweet
KMW0404	Naphthalene	Aromatics	pungent, dry, tarry
XMW0790	6,7-Dimethyl-1,2,3,5,8,8a-hexahydronaphthalene	Aromatics	–
WMW0227	Benzene, 1,4-dichloro-	Aromatics	–
KMW0115	Styrene	Aromatics	penetrating, balsamic, gasoline
KMW0273	Benzene, 1,2,4,5-tetramethyl-	Aromatics	rancid, sweet
KMW0307	Benzene, 1-methyl-4-(1-methylethenyl)-	Aromatics	phenol, spicy, clove, guaiacol
XMW0539	3,4-Dimethoxytoluene	Aromatics	–
XMW0041	Benzene, 1,2-dimethoxy-4-(1-propenyl)-	Aromatics	spicy, clove, blossom, carnation, woody
XMW0615	Phenol, m-tert-butyl-	Phenol	–
D356	3-Methoxy-5-methylphenol	Phenol	oakmoss, fruity, iodine, woody, hay
KMW0175	Phenol	Phenol	phenol, medicinal
XMW0791	Phenol, 4-methyl-2-nitro-	Phenol	–
NMW0112	Phenol, 2-(1,1-dimethylethyl)-	Phenol	–
D265	Dodecanenitrile	Nitrogen compounds	citrus, orange, peel, metallic, spicy
D355	2,6-Octadienenitrile, 3,7-dimethyl-, (Z)-	Nitrogen compounds	citral, lemon, aldehydic, metallic
XMW0762	Imidodicarbonic diamide	Nitrogen compounds	–
D94	Benzenemethanethiol	Sulfur compounds	sharp, alliaceous, onion, sulfury, garlic, horseradish, minty, coffee
KMW0162	Dimethyl triSulfur compounds	Sulfur compounds	sulfury, cooked onion, savory, meaty
D98	Benzene, (methylthio)-	Sulfur compounds	toluene, solvent, spicy, woody, sawdust
XMW1464	Benzene, (butoxymethyl)-	Ether	floral, rose
XMW0269	2-Methylbutanoic anhydride	Others	–
KMW0435	2-Decenal, (Z)-	Aldehyde	tallow
KMW0570	2-Dodecenal, (E)-	Aldehyde	citrus, metallic, mandarin, orange, waxy, aldehydic
KMW0068	3-Hexenal, (Z)-	Aldehyde	green, fatty, grassy, weedy, fruity, apple
KMW0066	Hexanal	Aldehyde	aldehyde, grassy, green, leafy, vinegar
XMW1118	CyclohexanecarboxAldehyde	Aldehyde	–
XMW0294	10-Undecenal	Aldehyde	waxy, aldehydic, rose, mandarin, citrus, soapy, fatty
XMW0255	BenzAldehyde, 2,4-dimethyl-	Aldehyde	naphthyl, cherry, almond, spice, vanilla
XMW0089	4-Decenal, (E)-	Aldehyde	fresh, aldehydic, citrus, orange, mandarin, tangerine, green, fatty
KMW0088	2-Hexenal, (E)-	Aldehyde	green, grassy
WMW0069	2-Propenal, 3-phenyl-	Aldehyde	sweet, spicy, aldehydic, aromatic, balsamic, cinnamyl, resinous, honey, powdery
XMW0246	OxiranecarboxAldehyde, 3-methyl-3-(4-methyl-3-pentenyl)-	Aldehyde	–
KMW0243	(E)-2-Octenal	Aldehyde	fresh, cucumber, fatty, green, herbal, banana, waxy, leafy
D401	2-Isopropyl-5-methylhex-2-enal	Aldehyde	herbal, lavender, woody, green, blueberry, tomato
XMW0170	BenzAldehyde, 3-ethyl-	Aldehyde	–
KMW0407	2,4-Nonadienal, (E,E)-	Aldehyde	fatty, melon, waxy, green, violet, leafy, cucumber, tropical, fruity, chicken
XMW0526	AcetAldehyde, (3,3-dimethylcyclohexylidene)-, (Z)-	Aldehyde	–
KMW0123	4-Heptenal, (Z)-	Aldehyde	oily, fatty, green, dairy, milky, creamy
KMW0122	Heptanal	Aldehyde	fresh, aldehydic, fatty, green, herbal, wine, ozonous
KMW0129	2,4-Hexadienal, (E,E)-	Aldehyde	sweet, green, spicy, floral, citrus
KMW0158	BenzAldehyde	Aldehyde	sweet, bitter, almond, cherry
KMW0343	2-Nonenal	Aldehyde	fatty, green, waxy, cucumber, melon
XMW0679	AcetAldehyde, tetramer	Aldehyde	–
KMW0361	BenzAldehyde, 4-ethyl-	Aldehyde	bitter, almond, sweet, anisic
XMW0675	2,6,6-Trimethylcyclohexa-1,4-dienecarbAldehyde	Aldehyde	–
XMW0217	cis-7-Decen-1-al	Aldehyde	citrus, aldehydic, cucumber
XMW0189	3-Cyclohexene-1-acetAldehyde, .alpha.,4-dimethyl-	Aldehyde	spicy, herbal
KMW0475	(2E,4Z)-2,4-Decadienal	Aldehyde	fried, fatty, geranium, green, waxy
XMW1237	3-Pentenoic Acid, 2,2-dimethyl-	Acid	–
NMW0209	Undecylenic Acid	Acid	sweet, woody
KMW0096	Butanoic Acid, 3-methyl-	Acid	sour, stinky, feet, sweaty, cheese, tropical
KMW0386	cis-Dihydrocarvone	Terpenoids	herbal, warm
KMW0556	Naphthalene, decahydro-4a-methyl-1-methylene-7-(1-methylethenyl)-, [4aR-(4a.alpha.,7.alpha.,8a.beta.)]-	Terpenoids	herbal
KMW0296	Cyclohexene, 1-methyl-4-(1-methylethylidene)-	Terpenoids	citrus, pine
NMW0093	D-Verbenone	Terpenoids	–
KMW0606	.alpha.-Muurolene	Terpenoids	woody
XMW1138	6-Octen-1-ol, 7-methyl-3-methylene-	Terpenoids	–
NMW0101	Ascaridole	Terpenoids	–
KMW0217	d-Limonene	Terpenoids	citrus
XMW0006	(+)-3-Carene	Terpenoids	sweet
NMW0041	(*E*)-4,8-Dimethylnona-1,3,7-triene	Terpenoids	–
KMW0595	1,3-Cyclohexadiene, 5-(1,5-dimethyl-4-hexenyl)-2-methyl-, [S-(R*,S*)]-	Terpenoids	spice, fresh, sharp
KMW0604	(1S,2E,6E,10R)-3,7,11,11-Tetramethylbicyclo[8.1.0]undeca-2,6-diene	Terpenoids	green, woody, weedy
D197	3-Oxatricyclo[4.1.1.0(2,4)]octane, 2,7,7-trimethyl-	Terpenoids	green
NMW0104	2,6-Octadien-1-ol, 3,7-dimethyl-	Terpenoids	–
WMW0040	trans-.beta.-Ocimene	Terpenoids	sweet, herbal
NMW0034	Fenchone	Terpenoids	herbal, cedar leaf, bitter, thuja, camphor, earthy, woody
XMW0913	cis-Chrysanthenol	Terpenoids	–
KMW0453	Linalyl acetate	Terpenoids	sweet, green, citrus, bergamot, lavender, woody
KMW0526	2-Buten-1-one, 1-(2,6,6-trimethyl-1,3-cyclohexadien-1-yl)-, (E)-	Terpenoids	apple, rose, honey, tobacco, sweet
KMW0582	.beta.-Guaiene	Terpenoids	sweet, woody, dry, guaiacwood, spicy, powdery
KMW0199	.beta.-Myrcene	Terpenoids	musty, balsamic, spice
NMW0075	Cyclohexanol, 1-methyl-4-(1-methylethylidene)-	Terpenoids	terpineol, lilac
NMW0113	6-Octen-1-ol, 3,7-dimethyl-, formate	Terpenoids	bergamot, cucumber, rose, apricot, peach, plum
KMW0254	1,5-Heptadien-4-one, 3,3,6-trimethyl-	Terpenoids	herbal, honey, minty, berry
KMW0479	1,3-Cyclohexadiene-1-carboxaldehyde, 2,6,6-trimethyl-	Terpenoids	fresh, herbal, phenol, metallic, rosemary, tobacco, spicy
WMW0194	Thujone	Terpenoids	cedar leaf
NMW0087	2-Oxabicyclo[2.2.2]octan-6-one, 1,3,3-trimethyl-	Terpenoids	–
WMW0215	.beta.-Ocimene	Terpenoids	apple, pear, fruity
D407	7-Octen-4-ol, 2-methyl-6-methylene-, (*S*)-	Terpenoids	–
KMW0291	Linalool	Terpenoids	floral, green
KMW0312	Furan, 3-(4-methyl-3-pentenyl)-	Terpenoids	woody
XMW0780	2,6-Dimethyl-1,3,5,7-octatetraene, E,E-	Terpenoids	–
XMW0542	1-Cyclohexene-1-carboxaldehyde, 4-(1-methylethyl)-	Terpenoids	–
KMW0388	Carveol	Terpenoids	minty, spearmint, cool, green, herbal, caraway, spicy
XMW0766	Cyclohexanone, 5-methyl-2-(1-methylethylidene)-	Terpenoids	minty
WMW0003	Eremophilene	Terpenoids	–
XMW0548	Bicyclo[7.2.0]undecane, 10,10-dimethyl-2,6-bis(methylene)-, [1S-(1R*,9S*)]-	Terpenoids	–
WMW0007	[1S-(1.alpha.,7.alpha.,8a.beta.)]-1,2,3,5,6,7,8,8a-octahydro-1,4-dimethyl-7-(1-methylethenyl)-Azulene	Terpenoids	–
XMW0817	(1*S*,5*S*,6*R*)-6-Methyl-2-methylene-6-(4-methylpent-3-en-1-yl)bicyclo[3.1.1]heptane	Terpenoids	–
D328	(5*R*,10*R*)-10-Methyl-6-methylene-2-(propan-2-ylidene)spiro[4.5]dec-7-ene	Terpenoids	–
XMW0157	(2*R*,3*R*,6*S*)-6-Isopropyl-3-methyl-2-(prop-1-en-2-yl)-3-vinylcyclohexanone	Terpenoids	–
XMW0422	3,5-Dimethyldodecane	Hydrocarbons	–
NMW0010	Dicyclopentadiene	Hydrocarbons	–
D266	1-Tridecene	Hydrocarbons	–
KMW0079	Octane	Hydrocarbons	gasoline
XMW0771	1,5-Cyclooctadiene, 3,4-dimethyl-	Hydrocarbons	–
XMW0279	E-1-Methoxy-4-hexene	Hydrocarbons	–
XMW0121	1-Nonene, 4,6,8-trimethyl-	Hydrocarbons	–
WMW0126	1,3-Hexadiene, 3-ethyl-2-methyl-	Hydrocarbons	nutty
XMW0159	2,6,10-Trimethyltridecane	Hydrocarbons	–
XMW0099	Decane, 2,3,5-trimethyl-	Hydrocarbons	–
XMW0265	Tetradecane, 2,6,10-trimethyl-	Hydrocarbons	–
WMW0213	Heptane, 2,4-dimethyl-	Hydrocarbons	–
XMW0403	Nonane, 2,5-dimethyl-	Hydrocarbons	–
KMW0137	Nonane	Hydrocarbons	linseed, oily, oily, sweaty
KMW0306	Undecane	Hydrocarbons	alkane
KMW0551	Tetradecane	Hydrocarbons	mild, waxy
KMW0208	Decane	Hydrocarbons	alkane
XMW0420	Undecane, 2,9-dimethyl-	Hydrocarbons	–
XMW0253	Undecane, 5-methyl-	Hydrocarbons	–
XMW0373	Undecane, 2-methyl-	Hydrocarbons	–
XMW0367	Undecane, 2,4-dimethyl-	Hydrocarbons	–
XMW1065	Octane, 2,6,6-trimethyl-	Hydrocarbons	–
XMW0023	4-methyl-1-(1-methylethyl)-Bicyclo[3.1.0]hex-2-ene	Hydrocarbons	–
XMW0884	5-Hexenal, 4-methylene-	Hydrocarbons	–
XMW0202	cis-2,6-Dimethyl-2,6-octadiene	Hydrocarbons	–
XMW0649	Nonane, 2,6-dimethyl-	Hydrocarbons	–
XMW0196	Decane, 4-methyl-	Hydrocarbons	pungent
WMW0082	1,10-Undecadiene	Hydrocarbons	–
XMW0499	Undecane, 3,4-dimethyl-	Hydrocarbons	–
XMW0243	Undecane, 2,5-dimethyl-	Hydrocarbons	–
WMW0145	Undecane, 4,6-dimethyl-	Hydrocarbons	–
XMW0110	Dodecane, 4,6-dimethyl-	Hydrocarbons	–
KMW0488	Tridecane	Hydrocarbons	alkane
w07	1H-Indene, 1-ethylidene-	Hydrocarbons	–
XMW0097	1,5-Cycloundecadiene, 8,8-dimethyl-9-methylene-	Hydrocarbons	–
KMW0046	2-Hexanone	Ketone	fruity, fungal, meaty, buttery
KMW0067	3-Penten-2-one, 4-methyl-	Ketone	pungent, earthy, vegetable, acrylate
XMW0162	3-Penten-2-one, 4-(acetyloxy)-, (*Z*)-	Ketone	–
XMW0900	3-Cyclohexen-1-one, 3,5,5-trimethyl-	Ketone	–
XMW0637	Cyclobutanone, 2,2,3-trimethyl-	Ketone	–
KMW0176	5-Hepten-2-one, 6-methyl-	Ketone	herbal, green, citrus, musty, lemon grass
D110	Bicyclo[3.1.1]heptan-3-one, 2-hydroxy-2,6,6-trimethyl-	Ketone	–
KMW0280	3,5-Octadien-2-one	Ketone	fruity, fatty, mushroom
XMW0648	3-Hexanone, 2,2-dimethyl-	Ketone	–
XMW0201	4-Hexen-3-one	Ketone	pungent, ethereal, spicy, green, tropical, metallic
D340	3-Hexanone, 1-phenyl-	Ketone	–
XMW1378	4,6-Octadiyn-3-one, 2-methyl-	Ketone	–
WMW0205	2,5-Octanedione	Ketone	–
NMW0773	Cyclohexanone	Ketone	minty, acetone
D206	1,2-Cyclohexanedione	Ketone	sweet, acorn, nut skin, maple, caramel, brothy
XMW0486	Cyclohexanone, 2,2,6-trimethyl-	Ketone	pungent, thujone, labdanum, honey, cistus
D413	4-Methyl-5-nonanone	Ketone	–
XMW0008	Isophorone	Ketone	cool, woody, sweet, green, camphor, fruity, musty, cedarwood, tobacco, leathery
NMW0070	1,2-Propanedione, 1-phenyl-	Ketone	plastic, buttery, honey
XMW0181	5-Hepten-2-one, 4,6-dimethyl-	Ketone	–
XMW0177	Ethanone, 1-(2-methyl-1-cyclopenten-1-yl)-	Ketone	–
w41	3-Octen-2-one, (*E*)-	Ketone	herbal, mushroom
KMW0230	3-Octen-2-one	Ketone	earthy, spicy, herbal, sweet, mushroom, hay, blueberry
XMW0909	2,6,6-Trimethylbicyclo[3.2.0]hept-2-en-7-one	Ketone	–
NMW0040	Benzyl methyl ketone	Ketone	almond
XMW0583	Ethanone, 1-(3-hydroxyphenyl)-	Ketone	–
KMW0182	Furan, 2-pentyl-	Heterocyclic compound	fruity, green, earthy, beany, vegetable, metallic
KMW0190	Pyrazine, trimethyl-	Heterocyclic compound	nut skin, earthy, powdery, cocoa, baked, potato, roasted, peanut, hazelnut, musty
WMW0081	cis-2-(2-Pentenyl)furan	Heterocyclic compound	–
XMW0115	5-Methyloxazolidine	Heterocyclic compound	–
D81	2-Furanpropanoic acid, ethyl ester	Heterocyclic compound	pineapple, fruity, spicy, jammy
NMW0201	Phenmetrazine	Heterocyclic compound	–
XMW0914	Ethanone, 1-(1H-pyrazol-4-yl)-	Heterocyclic compound	–
D188	Pyrazinamide	Heterocyclic compound	–
XMW0484	Furan, 2-propyl-	Heterocyclic compound	–
NMW0215	*N*,*N*-Dimethyl-1,2,3-trithian-5-Amine	Heterocyclic compound	–
XMW0132	Pyridine, 2,3,4,5-tetrahydro-	Heterocyclic compound	–
D191	2-Hexanoylfuran	Heterocyclic compound	sweet, fruity, ketonic, green, apricot, peach
XMW0714	3-Acetyl-1H-pyrroline	Heterocyclic compound	roasted
XMW0533	3-Methylbenzothiophene	Heterocyclic compound	–
NMW0217	1H-Pyrrolizine-7-methanol, 2,3,5,7a-tetrahydro-1-hydroxy-, (1S-cis)-	Heterocyclic compound	–
XMW0483	1H-Pyrrole-2-carboxAldehyde	Heterocyclic compound	musty, beefy, coffee
XMW1208	1H-Tetrazole	Heterocyclic compound	–
XMW0532	1H-Pyrazole-1-carboximidamide, 3,5-dimethyl-	Heterocyclic compound	–
KMW0425	Benzothiazole	Heterocyclic compound	meaty, vegetable, brown, cooked, beefy, coffee
KMW0286	Pyrazine, 2-methoxy-3-(1-methylethyl)-	Heterocyclic compound	beany, pea, earthy, chocolate, nutty
KMW0365	2-((3,3-Dimethyloxiran-2-yl)methyl)-3-methylfuran	Heterocyclic compound	green, earthy, citrus
KMW0126	Pyrazine, 2,6-dimethyl-	Heterocyclic compound	ethereal, cocoa, nutty, roasted, roasted, meaty, beefy, brown, coffee, buttermilky
XMW0412	3-Acetyl-2,5-dimethyl furan	Heterocyclic compound	sweet, musty, nutty, earthy, cocoa, corn, leathery
KMW0266	2-Thiophenemethanethiol	Heterocyclic compound	roasted, coffee, fishy
KMW0264	Pyrazine, 3-ethyl-2,5-dimethyl-	Heterocyclic compound	potato, cocoa, roasted, nutty
XMW0445	6-Ethyl-5,6-dihydro-2H-pyran-2-one	Heterocyclic compound	–
KMW0097	2-Methyl-3-furanthiol	Heterocyclic compound	sulfury, meaty, fishy, metallic
XMW0517	Thiophene, 3-ethyl-	Heterocyclic compound	styrene
XMW0444	1,2,4,5-Tetrazin-3-Amine	Heterocyclic compound	–
KMW0132	Pyrazine, 2,3-dimethyl-	Heterocyclic compound	nutty, nut skin, cocoa, peanut, buttery, coffee, walnut, caramel, roasted
XMW0065	Fomepizole	Heterocyclic compound	–
XMW0289	2-Acetyl-5-methylfuran	Heterocyclic compound	strong, musty, nutty, hay, coconut, coumarin, milky
XMW0051	4-Pyridinecarboxaldehyde	Heterocyclic compound	fruity
NMW0011	Benzofuran	Heterocyclic compound	aromatic
XMW0042	3H-1,2,4-Triazol-3-one, 1,2-dihydro-	Heterocyclic compound	–
XMW0267	2-Ethylpiperidine	Heterocyclic compound	–
XMW0076	1H-Pyrrole-3‑carbonitrile	Heterocyclic compound	–
KMW0240	Ethanone, 1-(1H-pyrrol-2-yl)-	Heterocyclic compound	musty, nut skin, maraschino, cherry, coumarin, licorice, walnut, bread
XMW1203	2,5-Furandione, dihydro-3-methyl-	Heterocyclic compound	–
D364	2-Ethoxy-3-methylpyrazine	Heterocyclic compound	hazelnut, roasted, almond, pineapple, earthy
XMW0168	3-Butylthiophene	Heterocyclic compound	–
XMW1429	1-(2-Hydroxyethyl)-1,2,4-triazole	Heterocyclic compound	–
XMW0644	1H-Imidazole, 2-propyl-	Heterocyclic compound	–
NMW0059	4-Aminopyridine	Heterocyclic compound	–
KMW0380	Pyrazine, 2-methoxy-3-(2-methylpropyl)-	Heterocyclic compound	green bell pepper, pea, galbanum
XMW1317	8-Azabicyclo[3.2.1]octane, 3-chloro-8-methyl-	Heterocyclic compound	–
NMW0110	Picolinamide	Heterocyclic compound	–
KMW0539	2(5H)-Furanone, 5-ethyl-	Heterocyclic compound	spice
D274	2H-1-Benzopyran-2-one, 3-methyl-	Heterocyclic compound	–
NMW0210	Furo[3,4-*c*]pyridin-1(3H)-one, 7-hydroxy-6-methyl-	Heterocyclic compound	–
mws0366	γ-Linolenic Acid*	Lipids	–
NMW0758	1-Butanol, 3-methyl-, formate	Ester	plum, black currant, ethereal, vinegar, dry earthy, fruity, green
NMW0016	Acetic acid, cyclohexyl ester	Ester	fruity, sweet, musty, ethereal
KMW0568	2H-Pyran-2-one, tetrahydro-6-pentyl-	Ester	creamy, coconut, fruity
XMW1401	n-Valeric acid cis-3-hexenyl ester	Ester	green, fruity, apple, pear, kiwi, unripe, banana, tropical
XMW1240	Hexanoic acid, 2-methylbutyl ester	Ester	ethereal
KMW0557	Benzoic acid, 2-hydroxy-, ethyl ester	Ester	caramel, pepperminty
KMW0373	Benzoic acid, ethyl ester	Ester	fruity, dry, musty, sweet, wintergreen
KMW0605	Pentanoic acid, 1-ethenyl-1,5-dimethyl-4-hexenyl ester	Ester	fruity, lavender, apricot, citrus
XMW0881	2-Butenoic acid, 3-methyl-, methyl ester	Ester	–
XMW1310	Isobutyl isovalerate	Ester	sweet, fruity, apple, raspberry, green, banana
w32	Acetic acid, heptyl ester	Ester	fruity, green, sweet
XMW0803	Iso-3-thujyl acetate	Ester	–
KMW0449	Bicyclo[2.2.1]heptan-2-ol, 1,7,7-trimethyl-, formate, endo-	Ester	green, earthy, herbal, balsamic, pine
XMW0933	Bicyclo[3.1.1]hept-2-en-6-ol, 2,7,7-trimethyl-, acetate, [1S-(1.alpha.,5.alpha.,6.beta.)]-	Ester	–
XMW0180	2-Propenoic acid, 2-methoxyethyl ester	Ester	–
D112	Benzeneacetic acid, methyl ester	Ester	floral, honey, spice, waxy, sweet
WMW0087	Butanoic Acid, 3-methylbutyl ester	Ester	fruity, green, apricot, pear, banana
NMW0122	Geranyl formate	Ester	fresh, rose, neroli, tea, rose, green
XMW1308	1-Octyl trifluoroacetate	Ester	–
D240	Propanoic acid, 2,2-dimethyl-, pentyl ester	Ester	–
XMW0073	3-Methylheptyl acetate	Ester	–
D143	Benzenepropanoic acid, methyl ester	Ester	honey, fruity, wine, balsamic, floral
w43	2-Oxepanone	Ester	–
NMW0037	Butanoic acid, 3-methyl-, 3-methylbutyl ester	Ester	sweet, fruity, green, ripe apple, jammy, tropical
D271	Propanoic acid, hexyl ester	Ester	pear, green, fruity, musty, rotten
D357	2-Propanol, 1-(dimethylamino)-, acetate (ester)	Ester	–
w29	4-Pentenyl Acetate	Ester	green, plastic, weedy, acrylate, vegetable, metallic, cooked meat, sulfury
XMW0299	Butanoic acid, butyl ester	Ester	fruity, banana, pineapple, green, cherry, tropical fruit, ripe fruit, juicy fruity
D417	3-Hexen-1-ol, acetate, (*E*)-	Ester	sharp, fruity, green, cortex, hyacinth, narcissus, rummy, unripe banana, pear
WMW0210	2-Hexen-1-ol, acetate, (E)-	Ester	sweet, privet, green, fresh, apple skin, banana, peel, waxy, apple
XMW1221	Acetic acid, (propylthio)-, methyl ester	Ester	–
D133	4-Heptanol, 2,6-dimethyl-, acetate	Ester	herbal, rhubarb, floral, lilac, banana
D305	Butanoic acid, 2-methyl-, 3-methylbutyl ester	Ester	sweet, fruity, citrus, cherry, blueberry, apple
XMW1127	Formic acid, octyl ester	Ester	fruity, rose, orange, waxy, cucumber
XMW1071	Butanoic acid, cyclopentyl ester	Ester	–
KMW0350	Acetic acid, phenylmethyl ester	Ester	sweet, floral, fruity, jasmin, fresh
XMW1079	Butanoic acid, 1-methylhexyl ester	Ester	fruity, green, vegetable, cheese, walnut
XMW0213	Isopentyl hexanoate	Ester	fruity, banana, apple, pineapple, green
KMW0411	1,6-Octadien-3-ol, 3,7-dimethyl-, formate	Ester	citrus, herbal, bergamot, lavender, soapy, fatty, green, woody
KMW0421	Methyl salicylate	Ester	caramel, pepperminty
KMW0441	Benzeneacetic acid, ethyl ester	Ester	minty
XMW0937	(1*R*,5*S*,6*R*)-2,7,7-Trimethylbicyclo[3.1.1]hept-2-en-6-yl acetate	Ester	–
D346	Methyl 6,6-dimethylbicyclo[3.1.1]hept-2-ene-2-carboxylate	Ester	–
KMW0585	Citronellyl isobutyrate	Ester	sweet, fruity, floral, geranium, tropical
D158	2-Phenoxyethyl isobutyrate	Ester	green, fruity, waxy, apple, nuances
KMW0610	Bornyl isovalerate	Ester	valerian, camphor, tropical

The PCA score plot of volatile metabolites showed (Fig. 5B), BT showed a significant separation from the other groups, suggesting that the BT processing has a significant effect on the volatiles of EUL. Additionally, HG was below the QC sample and also showed a tendency to separate from the other groups, indicating that the volatile metabolites were not as close to the GT group as the nonvolatile metabolites, and these two groups still had differences in volatile metabolites. However, GT and XY are very close to each other, this suggests that the the GT process does not have much effect on the volatiles.

Then, according to the table of significant volatile metabolites (Table 5) of the two-by-two comparison groups (BT vs. XY, GT vs. XY, HG vs. XY), it can be found that there are 12 categories of the main significant volatiles, mainly esters, aldehydes, alcohols, ketones and so on. After the BT processing, there were 14 volatile metabolites with up-regulation multiples of 100 times or more, mainly focusing on esters and aldehydes. 1-ethenyl-1,5-dimethyl-4-hexenyl ester, is fruity, lavender, apricot, citrusy. Methyl salicylate in it is the aroma component of black tea in general. It is obtained by enzymatic hydrolysis of glycoside aroma precursors in the leaf tissue after twisting. Similarly, there is up-regulation of benzoic acid as well as pyrazines and pyridines, which are aroma components contained in black tea processing. Both the GT process and HG caused a decrease in methyl salicylate. Thermal cleavage and esterification reactions of the GT processing resulted in the up-regulation of 11 substances, mainly heterocyclic compounds. There are 19 substances with significant upward adjustments for the HG, mainly esters and alcohols. Secondary metabolites alcohols, aldehydes, etc. may be produced as a result of the degradation of lipids which then undergo further oxidation (Shahidi & Hossain, 2022). It is speculated that the enzyme activity affects the rise of volatile components because the GT process is killed and the enzyme is inactivated due to high temperature. While the other two processes have the participation of enzymes, the main mechanism has to be further investigated.

**Table 5:** Table of major differences in composition between groups of volatile metabolites of different processed EUL.

Index	Compounds	Class I	Odor	BT_vs_XY_Type	GT_vs_XY_Type	HG_vs_XY_Type
KMW0182	Furan, 2-pentyl-	Heterocyclic compound	fruity, green, earthy, beany, vegetable, metallic	–	–	–
KMW0386	cis-Dihydrocarvone	Terpenoids	herbal, warm	down	down	down
NMW0057	Glutarimide	Amine	–	–	–	down
KMW0190	Pyrazine, trimethyl-	Heterocyclic compound	nut skin, earthy, powdery, cocoa, baked, potato, roasted, peanut, hazelnut, musty	–	down	down
WMW0081	cis-2-(2-Pentenyl)furan	Heterocyclic compound	–	up	down	down
NMW0758	1-Butanol, 3-methyl-, formate	Ester	plum, black currant, ethereal, vinegar, dry earthy, fruity, green	down	down	down
KMW0556	Naphthalene, decahydro-4a-methyl-1-methylene-7-(1-methylethenyl)-, [4aR-(4a.alpha.,7.alpha.,8a.beta.)]-	Terpenoids	herbal	–	down	down
KMW0296	Cyclohexene, 1-methyl-4-(1-methylethylidene)-	Terpenoids	citrus, pine	–	down	down
XMW0735	Benzenemethanol, .alpha.-2-propenyl-	Alcohol	–	down	down	down
KMW0046	2-Hexanone	Ketone	fruity, fungal, meaty, buttery	down	down	down
w08	Naphthalene, 1-methyl-	Aromatics	naphthyl, chemical, medicinal, camphor	–	–	down
NMW0016	Acetic acid, cyclohexyl ester	Ester	fruity, sweet, musty, ethereal	–	down	down
NMW0093	*D*-Verbenone	Terpenoids	–	up	down	down
KMW0606	.alpha.-Muurolene	Terpenoids	woody	–	down	down
XMW0916	(5-bromopentyl)-Benzene	Aromatics	–	–	down	down
XMW0422	3,5-Dimethyldodecane	Hydrocarbons	–	–	down	–
KMW0568	2H-Pyran-2-one, tetrahydro-6-pentyl-	Ester	creamy, coconut, fruity	–	down	down
XMW0115	5-Methyloxazolidine	Heterocyclic compound	–	down	down	down
KMW0067	3-Penten-2-one, 4-methyl-	Ketone	pungent, earthy, vegetable, acrylate	down	down	down
XMW0162	3-Penten-2-one, 4-(acetyloxy)-, (*Z*)-	Ketone	–	–	–	up
XMW0900	3-Cyclohexen-1-one, 3,5,5-trimethyl-	Ketone	–	down	down	down
XMW1138	6-Octen-1-ol, 7-methyl-3-methylene-	Terpenoids	–	down	down	down
D81	2-Furanpropanoic acid, ethyl ester	Heterocyclic compound	pineapple, fruity, spicy, jammy	down	down	down
XMW1401	n-Valeric acid cis-3-hexenyl ester	Ester	green, fruity, apple, pear, kiwi, unripe, banana, tropical	down	down	down
NMW0101	Ascaridole	Terpenoids	–	down	down	down
XMW1240	Hexanoic acid, 2-methylbutyl ester	Ester	ethereal	down	down	down
KMW0435	2-Decenal, (Z)-	Aldehyde	tallow	–	–	up
D266	1-Tridecene	Hydrocarbons	–	–	down	down
XMW0615	Phenol, m-tert-butyl-	Phenol	–	–	down	down
KMW0557	Benzoic acid, 2-hydroxy-, ethyl ester	Ester	caramel, pepperminty	up	down	down
KMW0570	2-Dodecenal, (*E*)-	Aldehyde	citrus, metallic, mandarin, orange, waxy, aldehydic	up	up	–
NMW0201	Phenmetrazine	Heterocyclic compound	–	up	up	–
D265	Dodecanenitrile	Nitrogen compounds	citrus, orange, peel, metallic, spicy	–	down	down
KMW0068	3-Hexenal, (Z)-	Aldehyde	green, fatty, grassy, weedy, fruity, apple	down	down	down
KMW0079	Octane	Hydrocarbons	gasoline	down	down	down
KMW0066	Hexanal	Aldehyde	aldehyde, grassy, green, leafy, vinegar	down	down	down
KMW0217	d-Limonene	Terpenoids	citrus	–	–	down
XMW0771	1,5-Cyclooctadiene, 3,4-dimethyl-	Hydrocarbons	–	up	down	down
XMW0914	Ethanone, 1-(1H-pyrazol-4-yl)-	Heterocyclic compound	–	down	down	down
KMW0373	Benzoic acid, ethyl ester	Ester	fruity, dry, musty, sweet, wintergreen	up	down	down
D188	Pyrazinamide	Heterocyclic compound	–	down	down	down
KMW0605	Pentanoic acid, 1-ethenyl-1,5-dimethyl-4-hexenyl ester	Ester	fruity, lavender, apricot, citrus	up	–	–
XMW0637	Cyclobutanone, 2,2,3-trimethyl-	Ketone	–	down	down	down
WMW0187	2-Hexanol	Alcohol	chemical, winey, fruity, fatty, terpenic, cauliflower	down	down	down
XMW0279	*E*-1-Methoxy-4-hexene	Hydrocarbons	–	down	down	down
XMW0484	Furan, 2-propyl-	Heterocyclic compound	–	down	down	down
XMW0881	2-Butenoic acid, 3-methyl-, methyl ester	Ester	–	down	down	down
XMW1118	CyclohexanecarboxAldehyde	Aldehyde	–	up	–	–
KMW0176	5-Hepten-2-one, 6-methyl-	Ketone	herbal, green, citrus, musty, lemon grass	up	up	–
XMW0006	(+)-3-Carene	Terpenoids	sweet	down	down	down
XMW0121	1-Nonene, 4,6,8-trimethyl-	Hydrocarbons	–	–	down	down
WMW0126	1,3-Hexadiene, 3-ethyl-2-methyl-	Hydrocarbons	nutty	–	–	–
w32	Acetic acid, heptyl ester	Ester	fruity, green, sweet	–	down	up
XMW0803	Iso-3-thujyl acetate	Ester	–	–	down	down
NMW0215	*N*,*N*-Dimethyl-1,2,3-trithian-5-Amine	Heterocyclic compound	–	–	down	down
XMW0294	10-Undecenal	Aldehyde	waxy, aldehydic, rose, mandarin, citrus, soapy, fatty	–	down	down
XMW0132	Pyridine, 2,3,4,5-tetrahydro-	Heterocyclic compound	–	–	–	up
D373	2,4-Heptadien-1-ol, (E,E)-	Alcohol	green, fruity, nutty, cheese	up	up	up
NMW0041	(E)-4,8-Dimethylnona-1,3,7-triene	Terpenoids	–	up	–	–
XMW0255	BenzAldehyde, 2,4-dimethyl-	Aldehyde	naphthyl, cherry, almond, spice, vanilla	up	–	–
D191	2-Hexanoylfuran	Heterocyclic compound	sweet, fruity, ketonic, green, apricot, peach	down	down	down
D110	Bicyclo[3.1.1]heptan-3-one, 2-hydroxy-2,6,6-trimethyl-	Ketone	–	–	–	–
KMW0449	Bicyclo[2.2.1]heptan-2-ol, 1,7,7-trimethyl-, formate, endo-	Ester	green, earthy, herbal, balsamic, pine	up	down	down
XMW0159	2,6,10-Trimethyltridecane	Hydrocarbons	–	up	up	–
KMW0595	1,3-Cyclohexadiene, 5-(1,5-dimethyl-4-hexenyl)-2-methyl-, [S-(R*,S*)]-	Terpenoids	spice, fresh, sharp	–	down	down
KMW0604	(1S,2E,6E,10R)-3,7,11,11-Tetramethylbicyclo[8.1.0]undeca-2,6-diene	Terpenoids	green, woody, weedy	–	down	down
XMW0714	3-Acetyl-1H-pyrroline	Heterocyclic compound	roasted	–	down	down
D197	3-Oxatricyclo[4.1.1.0(2,4)]octane, 2,7,7-trimethyl-	Terpenoids	green	–	down	down
KMW0280	3,5-Octadien-2-one	Ketone	fruity, fatty, mushroom	up	–	–
XMW0933	Bicyclo[3.1.1]hept-2-en-6-ol, 2,7,7-trimethyl-, acetate, [1S-(1.alpha.,5.alpha.,6.beta.)]-	Ester	–	up	–	down
XMW0533	3-Methylbenzothiophene	Heterocyclic compound	–	down	down	–
NMW0104	2,6-Octadien-1-ol, 3,7-dimethyl-	Terpenoids	–	–	–	–
D356	3-Methoxy-5-methylphenol	Phenol	oakmoss, fruity, iodine, woody, hay	–	down	down
NMW0217	1H-Pyrrolizine-7-methanol, 2,3,5,7a-tetrahydro-1-hydroxy-, (1S-cis)-	Heterocyclic compound	–	–	down	down
XMW0180	2-Propenoic acid, 2-methoxyethyl ester	Ester	–	down	down	down
KMW0102	1-Hexanol	Alcohol	ethereal, fusel, oily, fruity, alcoholic, sweet, green	–	down	down
XMW0648	3-Hexanone, 2,2-dimethyl-	Ketone	–	up	down	down
XMW0483	1H-Pyrrole-2-carboxAldehyde	Heterocyclic compound	musty, beefy, coffee	down	down	down
XMW1208	1H-Tetrazole	Heterocyclic compound	–	down	down	down
WMW0040	trans-.beta.-Ocimene	Terpenoids	sweet, herbal	up	down	–
XMW0659	Benzene, n-butyl-	Aromatics	–	up	down	up
NMW0034	Fenchone	Terpenoids	herbal, cedar leaf, bitter, thuja, camphor, earthy, woody	down	down	down
XMW0532	1H-Pyrazole-1-carboximidamide, 3,5-dimethyl-	Heterocyclic compound	–	down	down	down
XMW0099	Decane, 2,3,5-trimethyl-	Hydrocarbons	–	–	down	down
XMW0913	cis-Chrysanthenol	Terpenoids	–	–	–	down
D112	Benzeneacetic acid, methyl ester	Ester	floral, honey, spice, waxy, sweet	up	down	down
XMW0089	4-Decenal, (E)-	Aldehyde	fresh, aldehydic, citrus, orange, mandarin, tangerine, green, fatty	down	down	down
D355	2,6-Octadienenitrile, 3,7-dimethyl-, (Z)-	Nitrogen compounds	citral, lemon, aldehydic, metallic	up	down	down
KMW0425	Benzothiazole	Heterocyclic compound	meaty, vegetable, brown, cooked, beefy, coffee	–	down	down
KMW0453	Linalyl acetate	Terpenoids	sweet, green, citrus, bergamot, lavender, woody	down	down	down
w02	2,4-Decadien-1-ol	Alcohol	fatty, waxy, citrus, melon	up	–	down
KMW0526	2-Buten-1-one, 1-(2,6,6-trimethyl-1,3-cyclohexadien-1-yl)-, (*E*)-	Terpenoids	apple, rose, honey, tobacco, sweet	–	down	down
XMW0265	Tetradecane, 2,6,10-trimethyl-	Hydrocarbons	–	–	down	down
KMW0060	Toluene	Aromatics	sweet	–	–	up
WMW0213	Heptane, 2,4-dimethyl-	Hydrocarbons	–	up	up	–
KMW0092	2-Furanmethanol	Alcohol	alcoholic, chemical, musty, sweet, caramel, bread, coffee	down	down	down
KMW0088	2-Hexenal, (*E*)-	Aldehyde	green, grassy	–	down	down
XMW0201	4-Hexen-3-one	Ketone	pungent, ethereal, spicy, green, tropical, metallic	down	down	down
KMW0175	Phenol	Phenol	phenol, medicinal	–	–	down
KMW0323	Benzyl Alcohol	Alcohol	floral, rose, phenol, balsamic	up	down	down
WMW0127	3-Octen-1-ol, (Z)-	Alcohol	fresh, fatty, grassy, melon, green, cortex, herbal, earthy, fusel, spicy	–	down	–
KMW0286	Pyrazine, 2-methoxy-3-(1-methylethyl)-	Heterocyclic compound	beany, pea, earthy, chocolate, nutty	down	down	down
KMW0365	2-((3,3-Dimethyloxiran-2-yl)methyl)-3-methylfuran	Heterocyclic compound	green, earthy, citrus	down	down	down
KMW0404	Naphthalene	Aromatics	pungent, dry, tarry	down	down	down
XMW0790	6,7-Dimethyl-1,2,3,5,8,8a-hexahydronaphthalene	Aromatics	–	up	down	down
WMW0069	2-Propenal, 3-phenyl-	Aldehyde	sweet, spicy, aldehydic, aromatic, balsamic, cinnamyl, resinous, honey, powdery	up	–	down
NMW0122	Geranyl formate	Ester	fresh, rose, neroli, tea, rose, green	–	down	down
D340	3-Hexanone, 1-phenyl-	Ketone	–	–	–	down
KMW0582	.beta.-Guaiene	Terpenoids	sweet, woody, dry, guaiacwood, spicy, powdery	–	down	down
WMW0227	Benzene, 1,4-dichloro-	Aromatics	–	down	down	down
XMW1378	4,6-Octadiyn-3-one, 2-methyl-	Ketone	–	down	down	down
D240	Propanoic acid, 2,2-dimethyl-, pentyl ester	Ester	–	–	–	up
XMW0073	3-Methylheptyl acetate	Ester	–	up	–	up
XMW0269	2-Methylbutanoic anhydride	Others	–	down	down	down
NMW0075	Cyclohexanol, 1-methyl-4-(1-methylethylidene)-	Terpenoids	terpineol, lilac	up	down	down
XMW0246	OxiranecarboxAldehyde, 3-methyl-3-(4-methyl-3-pentenyl)-	Aldehyde	–	up	down	down
NMW0113	6-Octen-1-ol, 3,7-dimethyl-, formate	Terpenoids	bergamot, cucumber, rose, apricot, peach, plum	down	down	down
D143	Benzenepropanoic acid, methyl ester	Ester	honey, fruity, wine, balsamic, floral	up	–	down
NMW0209	Undecylenic Acid	Acid	sweet, woody	–	down	down
WMW0205	2,5-Octanedione	Ketone	–	up	–	–
NMW0773	Cyclohexanone	Ketone	minty, acetone	–	–	–
KMW0137	Nonane	Hydrocarbons	linseed, oily, oily, sweaty	–	–	up
KMW0126	Pyrazine, 2,6-dimethyl-	Heterocyclic compound	ethereal, cocoa, nutty, roasted, roasted, meaty, beefy, brown, coffee, buttermilky	–	up	–
KMW0194	2-Octanol	Alcohol	fresh, spicy, green, woody, herbal, earthy	–	up	up
KMW0243	(E)-2-Octenal	Aldehyde	fresh, cucumber, fatty, green, herbal, banana, waxy, leafy	down	down	–
D206	1,2-Cyclohexanedione	Ketone	sweet, acorn, nut skin, maple, caramel, brothy	–	down	down
KMW0254	1,5-Heptadien-4-one, 3,3,6-trimethyl-	Terpenoids	herbal, honey, minty, berry	–	down	down
XMW0412	3-Acetyl-2,5-dimethyl furan	Heterocyclic compound	sweet, musty, nutty, earthy, cocoa, corn, leathery	–	down	down
KMW0306	Undecane	Hydrocarbons	alkane	–	down	down
KMW0355	trans,cis-2,6-Nonadien-1-ol	Alcohol	green, cucumber, oily, violet, leafy	down	down	down
XMW0711	Formamide, *N*-phenyl-	Amine	–	up	down	down
XMW0424	2,6-Dimethyl-1-nonen-3-yn-5-ol	Alcohol	–	–	down	down
KMW0479	1,3-Cyclohexadiene-1-carboxaldehyde, 2,6,6-trimethyl-	Terpenoids	fresh, herbal, phenol, metallic, rosemary, tobacco, spicy	up	–	–
KMW0551	Tetradecane	Hydrocarbons	mild, waxy	–	down	down
KMW0629	1-Dodecanol	Alcohol	earthy, soapy, waxy, fatty, honey, coconut	up	up	–
XMW0420	Undecane, 2,9-dimethyl-	Hydrocarbons	–	down	down	down
w43	2-Oxepanone	Ester	–	–	down	–
KMW0266	2-Thiophenemethanethiol	Heterocyclic compound	roasted, coffee, fishy	–	–	–
KMW0264	Pyrazine, 3-ethyl-2,5-dimethyl-	Heterocyclic compound	potato, cocoa, roasted, nutty	up	up	–
XMW0445	6-Ethyl-5,6-dihydro-2H-pyran-2-one	Heterocyclic compound	–	down	down	down
XMW0486	Cyclohexanone, 2,2,6-trimethyl-	Ketone	pungent, thujone, labdanum, honey, cistus	up	down	down
D413	4-Methyl-5-nonanone	Ketone	–	–	–	up
WMW0194	Thujone	Terpenoids	cedar leaf	–	down	down
NMW0037	Butanoic acid, 3-methyl-, 3-methylbutyl ester	Ester	sweet, fruity, green, ripe apple, jammy, tropical	–	–	–
D94	Benzenemethanethiol	Sulfur compounds	sharp, alliaceous, onion, sulfury, garlic, horseradish, minty, coffee	–	down	down
D401	2-Isopropyl-5-methylhex-2-enal	Aldehyde	herbal, lavender, woody, green, blueberry, tomato	–	–	–
D271	Propanoic acid, hexyl ester	Ester	pear, green, fruity, musty, rotten	–	–	–
XMW0008	Isophorone	Ketone	cool, woody, sweet, green, camphor, fruity, musty, cedarwood, tobacco, leathery	–	down	down
XMW0253	Undecane, 5-methyl-	Hydrocarbons	–	down	down	down
XMW0373	Undecane, 2-methyl-	Hydrocarbons	–	–	down	down
KMW0344	1-Nonanol	Alcohol	fresh, clean, fatty, floral, rose, orange, dusty, wet, oily	down	down	down
XMW0170	BenzAldehyde, 3-ethyl-	Aldehyde	–	up	down	down
XMW1072	(*E*)-2,6-Dimethylocta-5,7-dien-2-ol	Alcohol	–	down	down	down
NMW0070	1,2-Propanedione, 1-phenyl-	Ketone	plastic, buttery, honey	up	down	down
KMW0407	2,4-Nonadienal, (E,E)-	Aldehyde	fatty, melon, waxy, green, violet, leafy, cucumber, tropical, fruity, chicken	up	down	down
NMW0087	2-Oxabicyclo[2.2.2]octan-6-one, 1,3,3-trimethyl-	Terpenoids	–	up	down	down
XMW0526	AcetAldehyde, (3,3-dimethylcyclohexylidene)-, (*Z*)-	Aldehyde	–	up	down	down
KMW0123	4-Heptenal, (Z)-	Aldehyde	oily, fatty, green, dairy, milky, creamy	–	–	–
KMW0122	Heptanal	Aldehyde	fresh, aldehydic, fatty, green, herbal, wine, ozonous	–	–	–
XMW1471	1-ButanAmine, *N*-methyl-N-2-propenyl-	Amine	–	down	down	down
XMW0517	Thiophene, 3-ethyl-	Heterocyclic compound	styrene	down	down	down
KMW0098	3-Hexen-1-ol, (Z)-	Alcohol	fresh, green, grass, foliage, vegetable, herbal, oily	–	down	down
D357	2-Propanol, 1-(dimethylamino)-, acetate (ester)	Ester	–	up	down	down
KMW0096	Butanoic Acid, 3-methyl-	Acid	sour, stinky, feet, sweaty, cheese, tropical	down	down	down
XMW0023	4-methyl-1-(1-methylethyl)-Bicyclo[3.1.0]hex-2-ene	Hydrocarbons	–	up	–	–
XMW0444	1,2,4,5-Tetrazin-3-Amine	Heterocyclic compound	–	–	–	–
KMW0132	Pyrazine, 2,3-dimethyl-	Heterocyclic compound	nutty, nut skin, cocoa, peanut, buttery, coffee, walnut, caramel, roasted	–	down	down
KMW0115	Styrene	Aromatics	penetrating, balsamic, gasoline	down	down	down
w29	4-Pentenyl Acetate	Ester	green, plastic, weedy, acrylate, vegetable, metallic, cooked meat, sulfury	–	down	–
XMW0884	5-Hexenal, 4-methylene-	Hydrocarbons	–	–	down	–
XMW1307	Cyclohexanol, 1-methyl-	Alcohol	–	–	–	–
KMW0129	2,4-Hexadienal, (E,E)-	Aldehyde	sweet, green, spicy, floral, citrus	–	–	up
XMW0065	Fomepizole	Heterocyclic compound	–	down	down	down
KMW0158	BenzAldehyde	Aldehyde	sweet, bitter, almond, cherry	up	–	–
XMW0289	2-Acetyl-5-methylfuran	Heterocyclic compound	strong, musty, nutty, hay, coconut, coumarin, milky	–	down	down
XMW0181	5-Hepten-2-one, 4,6-dimethyl-	Ketone	–	up	down	down
XMW0642	Cyclohexanol, 3,5-dimethyl-	Alcohol	–	down	down	–
KMW0162	Dimethyl triSulfur compounds	Sulfur compounds	sulfury, cooked onion, savory, meaty	down	–	–
XMW0051	4-Pyridinecarboxaldehyde	Heterocyclic compound	fruity	up	–	–
XMW0299	Butanoic acid, butyl ester	Ester	fruity, banana, pineapple, green, cherry, tropical fruit, ripe fruit, juicy fruity	–	–	–
XMW0202	cis-2,6-Dimethyl-2,6-octadiene	Hydrocarbons	–	–	down	down
XMW0434	4-Hexen-1-ol, acetate	Alcohol	–	down	down	down
XMW0177	Ethanone, 1-(2-methyl-1-cyclopenten-1-yl)-	Ketone	–	–	down	down
WMW0103	2-Cyclopentylethanol	Alcohol	–	down	down	down
NMW0011	Benzofuran	Heterocyclic compound	aromatic	–	up	–
D417	3-Hexen-1-ol, acetate, (E)-	Ester	sharp, fruity, green, cortex, hyacinth, narcissus, rummy, unripe banana, pear	down	down	down
WMW0210	2-Hexen-1-ol, acetate, (E)-	Ester	sweet, privet, green, fresh, apple skin, banana, peel, waxy, apple	up	down	down
NMW0013	Benzylamine	Amine	–	down	down	down
w41	3-Octen-2-one, (E)-	Ketone	herbal, mushroom	–	down	down
XMW1221	Acetic acid, (propylthio)-, methyl ester	Ester	–	up	down	down
WMW0215	.beta.-Ocimene	Terpenoids	apple, pear, fruity	up	down	down
KMW0230	3-Octen-2-one	Ketone	earthy, spicy, herbal, sweet, mushroom, hay, blueberry	up	down	down
XMW0076	1H-Pyrrole-3‑carbonitrile	Heterocyclic compound	–	up	down	up
KMW0240	Ethanone, 1-(1H-pyrrol-2-yl)-	Heterocyclic compound	musty, nut skin, maraschino, cherry, coumarin, licorice, walnut, bread	down	down	down
D387	1-Hexanol, 3,5,5-trimethyl-	Alcohol	grassy, green, weedy, floral, earthy, aldehydic, hay, straw, leafy	–	–	–
XMW0196	Decane, 4-methyl-	Hydrocarbons	pungent	up	–	up
NMW0022	Benzenemethanol, .alpha.-methyl-	Alcohol	fresh, sweet, gardenia, hyacinth	–	down	down
D364	2-Ethoxy-3-methylpyrazine	Heterocyclic compound	hazelnut, roasted, almond, pineapple, earthy	down	down	down
KMW0276	1-Octanol	Alcohol	intense citrus, rose	down	down	up
WMW0082	1,10-Undecadiene	Hydrocarbons	–	–	down	down
WMW0092	1-OctanAmine,*N*-methyl-	Amine	–	–	down	down
D133	4-Heptanol, 2,6-dimethyl-, acetate	Ester	herbal, rhubarb, floral, lilac, banana	–	down	down
XMW0168	3-Butylthiophene	Heterocyclic compound	–	–	down	down
XMW1429	1-(2-Hydroxyethyl)-1,2,4-triazole	Heterocyclic compound	–	–	down	down
XMW0644	1H-Imidazole, 2-propyl-	Heterocyclic compound	–	–	down	down
D407	7-Octen-4-ol, 2-methyl-6-methylene-, (*S*)-	Terpenoids	–	–	down	down
KMW0291	Linalool	Terpenoids	floral, green	–	down	down
D305	Butanoic acid, 2-methyl-, 3-methylbutyl ester	Ester	sweet, fruity, citrus, cherry, blueberry, apple	–	–	–
XMW1233	3-Cyclopentyl-1-propanol	Alcohol	–	up	–	–
XMW0410	1-Nonen-4-ol	Alcohol	–	up	–	–
KMW0294	2-Octen-1-ol, (*E*)-	Alcohol	green, citrus, vegetable, fatty	down	down	up
NMW0038	Benzenemethanol, 4-methyl-	Alcohol	mild, floral	–	down	down
D98	Benzene, (methylthio)-	Sulfur compounds	toluene, solvent, spicy, woody, sawdust	–	–	–
XMW0909	2,6,6-Trimethylbicyclo[3.2.0]hept-2-en-7-one	Ketone	–	up	–	–
XMW1127	Formic acid, octyl ester	Ester	fruity, rose, orange, waxy, cucumber	–	down	up
KMW0307	Benzene, 1-methyl-4-(1-methylethenyl)-	Aromatics	phenol, spicy, clove, guaiacol	–	down	down
KMW0312	Furan, 3-(4-methyl-3-pentenyl)-	Terpenoids	woody	–	down	down
XMW1071	Butanoic acid, cyclopentyl ester	Ester	–	–	–	up
XMW0780	2,6-Dimethyl-1,3,5,7-octatetraene, E,E-	Terpenoids	–	–	down	down
KMW0343	2-Nonenal	Aldehyde	fatty, green, waxy, cucumber, melon	–	down	down
NMW0059	4-Aminopyridine	Heterocyclic compound	–	down	down	down
XMW0679	AcetAldehyde, tetramer	Aldehyde	–	down	–	down
KMW0350	Acetic acid, phenylmethyl ester	Ester	sweet, floral, fruity, jasmin, fresh	up	down	down
KMW0361	BenzAldehyde, 4-ethyl-	Aldehyde	bitter, almond, sweet, anisic	up	–	–
XMW0539	3,4-Dimethoxytoluene	Aromatics	–	up	down	down
XMW0542	1-Cyclohexene-1-carboxaldehyde, 4-(1-methylethyl)-	Terpenoids	–	up	down	down
XMW0499	Undecane, 3,4-dimethyl-	Hydrocarbons	–	down	down	down
XMW0675	2,6,6-Trimethylcyclohexa-1,4-dienecarbAldehyde	Aldehyde	–	up	down	down
KMW0380	Pyrazine, 2-methoxy-3-(2-methylpropyl)-	Heterocyclic compound	green bell pepper, pea, galbanum	down	down	down
KMW0376	2-Nonanol	Alcohol	rose	up	–	–
WMW0145	Undecane, 4,6-dimethyl-	Hydrocarbons	–	down	down	down
KMW0388	Carveol	Terpenoids	minty, spearmint, cool, green, herbal, caraway, spicy	up	down	down
XMW0766	Cyclohexanone, 5-methyl-2-(1-methylethylidene)-	Terpenoids	minty	–	down	down
XMW0213	Isopentyl hexanoate	Ester	fruity, banana, apple, pineapple, green	down	down	down
XMW1317	8-Azabicyclo[3.2.1]octane, 3-chloro-8-methyl-	Heterocyclic compound	–	up	down	down
KMW0411	1,6-Octadien-3-ol, 3,7-dimethyl-, formate	Ester	citrus, herbal, bergamot, lavender, soapy, fatty, green, woody	up	down	down
XMW0545	Benzenemethanamine, N-(1-methylethyl)-	Amine	–	up	down	down
XMW0189	3-Cyclohexene-1-acetAldehyde, .alpha.,4-dimethyl-	Aldehyde	spicy, herbal	up	down	–
KMW0421	Methyl salicylate	Ester	caramel, pepperminty	up	down	down
XMW0762	Imidodicarbonic diamide	Nitrogen compounds	–	down	down	down
XMW1464	Benzene, (butoxymethyl)-	Ether	floral, rose	up	–	down
XMW0583	Ethanone, 1-(3-hydroxyphenyl)-	Ketone	–	up	down	down
XMW0791	Phenol, 4-methyl-2-nitro-	Phenol	–	down	down	down
KMW0441	Benzeneacetic acid, ethyl ester	Ester	minty	–	–	down
XMW0937	(1*R*,5*S*,6*R*)-2,7,7-Trimethylbicyclo[3.1.1]hept-2-en-6-yl acetate	Ester	–	up	–	down
NMW0110	Picolinamide	Heterocyclic compound	–	up	down	down
NMW0112	Phenol, 2-(1,1-dimethylethyl)-	Phenol	–	up	–	down
XMW0110	Dodecane, 4,6-dimethyl-	Hydrocarbons	–	–	down	down
KMW0475	(2E,4*Z*)-2,4-Decadienal	Aldehyde	fried, fatty, geranium, green, waxy	–	down	down
KMW0488	Tridecane	Hydrocarbons	alkane	–	down	down
D346	Methyl 6,6-dimethylbicyclo[3.1.1]hept-2-ene-2-carboxylate	Ester	–	–	down	down
w07	1H-Indene, 1-ethylidene-	Hydrocarbons	–	–	–	down
XMW0041	Benzene, 1,2-dimethoxy-4-(1-propenyl)-	Aromatics	spicy, clove, blossom, carnation, woody	–	down	down
KMW0539	2(5H)-Furanone, 5-ethyl-	Heterocyclic compound	spice	–	–	–
D274	2H-1-Benzopyran-2-one, 3-methyl-	Heterocyclic compound	–	–	down	down
WMW0003	Eremophilene	Terpenoids	–	–	down	down
NMW0210	Furo[3,4-*c*]pyridin-1(3H)-one, 7-hydroxy-6-methyl-	Heterocyclic compound	–	–	down	down
XMW0097	1,5-Cycloundecadiene, 8,8-dimethyl-9-methylene-	Hydrocarbons	–	–	down	down
KMW0585	Citronellyl isobutyrate	Ester	sweet, fruity, floral, geranium, tropical	–	down	down
KMW0588	3-Cyclohexene-1-ethanol, .beta.,4-dimethyl-	Alcohol	fruity, herbal	–	down	down
D158	2-Phenoxyethyl isobutyrate	Ester	green, fruity, waxy, apple, nuances	–	down	down
XMW0548	Bicyclo[7.2.0]undecane, 10,10-dimethyl-2,6-bis(methylene)-, [1S-(1R*,9S*)]-	Terpenoids	–	–	down	down
WMW0007	[1S-(1.alpha.,7.alpha.,8a.beta.)]-1,2,3,5,6,7,8,8a-octahydro-1,4-dimethyl-7-(1-methylethenyl)-Azulene	Terpenoids	–	up	–	–
XMW0817	(1*S*,5*S*,6*R*)-6-Methyl-2-methylene-6-(4-methylpent-3-en-1-yl)bicyclo[3.1.1]heptane	Terpenoids	–	–	down	down
D328	(5*R*,10*R*)-10-Methyl-6-methylene-2-(propan-2-ylidene)spiro[4.5]dec-7-ene	Terpenoids	–	–	down	down
KMW0610	Bornyl isovalerate	Ester	valerian, camphor, tropical	–	down	down
XMW0157	(2*R*,3*R*,6*S*)-6-Isopropyl-3-methyl-2-(prop-1-en-2-yl)-3-vinylcyclohexanone	Terpenoids	–	–	down	down

### 3.5 Flavoromics analysis

Radar plotting (Fig. S4) for flavor characterization of differential metabolites using one-to-one comparisons of the detected volatile fractions. The top 10 sensory flavors with the highest number of annotations were selected for plotting, with green, fruity, and sweet being the three standout flavors. This is consistent with the results of this experiment tasted by the panelists, where 4 samples tasted of a strong grassy flavor.

**Supplementary fig S5:**
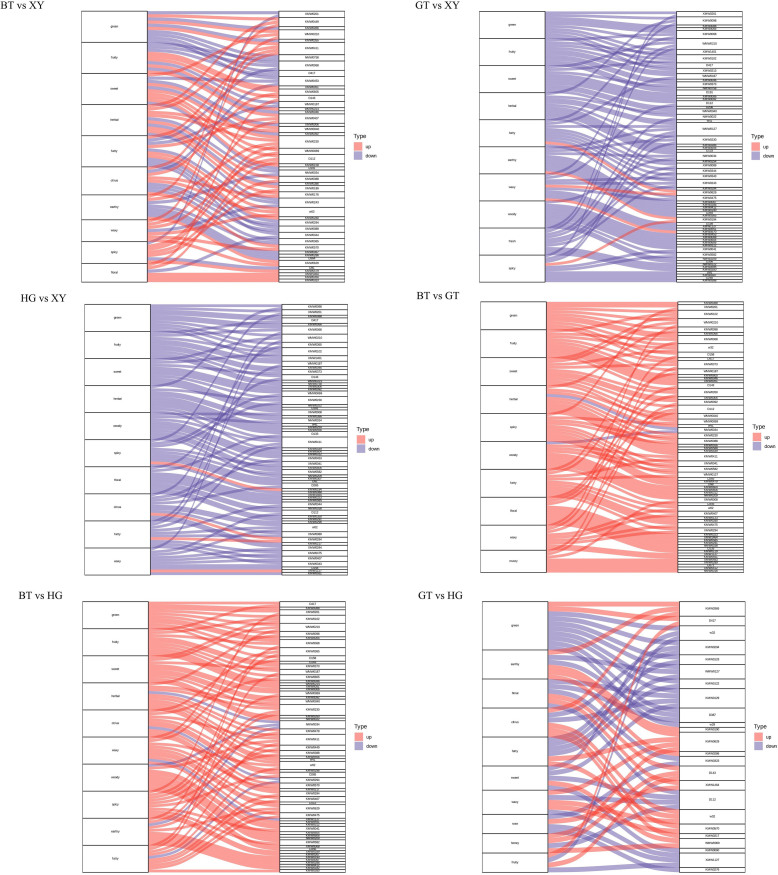
Differential metabolite flavor wheel plots for each comparison group; Note: the innermost circle is the differential comparison group, the second circle is the top 10 sensory flavor profiles with the highest number of differential metabolites annotated to the comparison group, the number in parentheses indicates the number of differential metabolites annotated to the sensory flavor profile, and the outermost circle indicates the differential metabolites, if the number of differential metabolites annotated to a sensory flavor profile If the number of differential metabolites annotated to an organoleptic flavor profile exceeds 10, the top 10 differential metabolites with the largest VIP values are displayed.

Further combining the differential metabolite flavor wheels for each comparison group (Fig.S5), there were 21 major differential metabolites for green flavor in BT vs. XY, 36 in GT vs. XY, and 33 in HG vs. XY. Hexanal dominated the grassy flavor in GT, while BT exhibited higher methyl salicylate, a marker for floral notes. This aligns with Aspergillus spp.-mediated esterification in dark tea fermentation. It shows that the BT processing is able to relatively reduce the generation of green flavor, and the GT process has the strongest green flavor. The study by Hu et al. (2020) showed that hexanal is capable of producing grassy and green odor at a low threshold, which is one of the volatiles responsible for the green odor in EUL. The next comparison was for fruity flavors, with 18 fruity key differentiators for BT vs. XY; 24 for GT vs. XY; and 25 for HG vs. XY. This suggests that the BT process does not add fruity flavors well, while the HG/GT treatments are more capable. Lastly, the sweetness comparison shows that the HG treatment is not very different from the GT treatment in terms of the level of sweetness, while the BT treatment will be a little less sweet.

The top 10 sensory flavors with the highest number of annotations were selected for Sankey map (Fig. S6) based on screening criteria for each of the difference comparison groups. The differential volatile metabolites of the two groups, GT vs. XY and HG vs. XY, showed a significant downward trend. Whereas the BT vs. XY groups showed a partial upward trend, proving once again that the the BT process is capable of stimulating the production of flavor volatiles. The BT vs. GT and BT vs. HG showed a trend of significant metabolite up-regulation, while GT vs. HG showed partial up-regulation.

### 3.6 KEGG enrichment analysis of differential metabolites from EUL with different processing methods

**Fig. 6:**
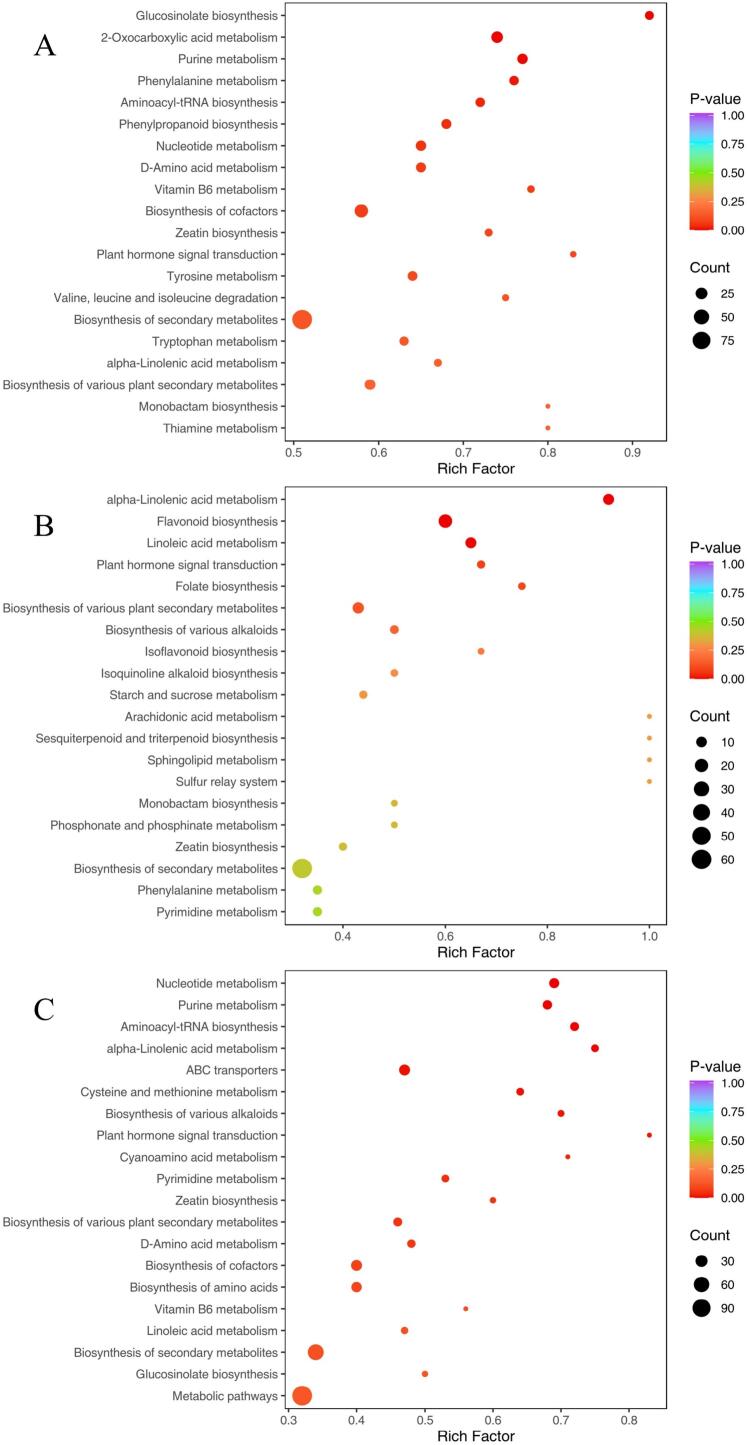
Bubble diagram for KEGG pathway enrichment analysis (A: BT vs. XY; B: GT vs. XY; C: HG vs. XY).

After enrichment analysis of differential metabolites in comparative groups of EUL with different processing methods, the KEGG database was further used to characterize the partitioning of these compounds in different pathways (Fig. 6). As Fig. 6, the differential metabolites of EUL under different processing methods were mainly involved in 43 different metabolic pathways, and the common pathways involved were, zeatin biosynthesis, plant hormone signal transduction, and α-linolenic acid metabolism. The differences in metabolic responses between the different treatment groups were mainly in the pathways related to amino acid metabolism, further confirming the substantial effects of different processing on the individual components of EUL. In the spectrum of metabolic pathways. The differences in metabolic responses between BT vs XY are mainly in glucosinolate biosynthesis, 2-0xocarboxylic acid metabolism, purine metabolism, phenylalanine metabolism, aminoacyl- tRNA biosynthesis and other related pathways. The metabolic pathways expressed in GT vs XY were: alpha-linolenic acid metabolism, flavonoid biosynthesis, linoleic acid metabolism, plant hormone signal transduction, folate biosynthesis. The main metabolic pathways enriched in HG vs XY were nucleotide metabolism, purine metabolism, aminoacyl-tRNA biosynthesis, alpha-Linolenic acid metabolism, and ABC. Overall, KEGG enrichment analysis revealed that different processing methods significantly affected pathways such as amino acid metabolism (ko00270), flavonoid biosynthesis (ko00941), and lipid oxidation (ko00591) (Fig. 6A).The HG process (low-temperature drying) significantly upregulated essential amino acids (e.g., phenylalanine, histidine) (Table 3), likely due to enhanced proteolytic enzyme activity during drying (Maeda & Dudareva, 2012). In contrast, amino acid reduction in BT (black tea processing) may result from microbial consumption during fermentation to generate volatiles (e.g., methyl salicylate) (LUO, et al., 2021. GT (green tea processing) increased catechins but degraded antioxidant flavonoids (e.g., quercetin) due to high-temperature inactivation of oxidative enzymes (Rigling et al., 2021). The widespread downregulation of flavonoids in BT (Fig. 4A) correlates with PPO-mediated oxidation and polymerization (Dongqing, 2001). Hydroxylated fatty acids in BT (Table 3) may arise from microbial β-oxidation during fermentation, while lysophospholipid enrichment in HG could stem from thermal degradation of triglycerides (Maeda & Dudareva, 2012).the different metabolic pathways mainly control the metabolism of bioactive compounds (e.g., amino acids, phenolic acids, flavonoids, nucleotides, lipids, monosaccharides, carbohydrates, and alkaloids), in agreement with the observation of differential compounds.

## 4 Conclusion

A broadly targeted metabolomics approach was used to identify and analyze metabolites from fresh EUL (XY), traditionally dried EUL (HG), green tea processed EUL (GT), and black tea processed EUL (BT). A total of 1839 non-volatile metabolites (including 12 subclasses) and 289 volatile metabolites (including 16 subclasses) were identified and detected. A total of 1503 differential compounds, consisting mainly of amino acids, lipids, phenolic acids, flavonoids, nucleotides, terpenoids and alkaloids, were observed in the different comparison groups. BT processing significantly reduced the content of amino acids, but enriched the content of various secondary metabolites (e.g. phenolic acids, organic acids, flavonoids, monosaccharides, and alkaloids) at the EUL level. GT processing, on the other hand, is significantly downregulated in the presence of lipids, but the high levels are concentrated in substances such as flavonoids, phenolic acids, and tannins. HG processing makes amino acids, lipids, etc. more upregulated and also retains some of the original substances in XY very well. Overall, different processing methods for EUL functionality, value and other aspects can each have their own advantages, to improve the quality of EUL products have a positive significance. In addition, BT processing can also be considered as the optimal processing method for EUL to produce the respective secondary metabolites, and the other two methods also have some processing significance and advantages. The processing of EUL should be based on the selection of suitable methods according to the processing objectives required in actual production.The results of this study provide insights into the mechanism of changes in some metabolites caused by different processing. It also provides health activity evaluation of metabolites produced by different processes of *Eucommia ulmoides* tea, which can be used as a functional reference for *Eucommia ulmoides* tea processing. In subsequent studies, in-depth investigation of the evolutionary trajectory of volatile and non-volatile products in the middle of EUL using different processing techniques can be explored, as well as further investigation of whether the flavor profile can be further improved.

The following are the supplementary data related to this article.

Supplementary material 1 Clustering heatmap of amino acids and their derivatives metabolites from differently processed *Eucommia ulmoides* leaves Supplementary material 1

Supplementary material 2 Clustering heatmap of lipid metabolites from differently processed *Eucommia ulmoides* leaves Supplementary material 2

Supplementary material 3 Clustering heatmap of flavonoid metabolites from differently processed *Eucommia ulmoides* leaves Supplementary material 3

Supplementary material 4 Clustering heatmap of phenolic acid metabolites from differently processed *Eucommia ulmoides* leaves Supplementary material 4

Supplementary material 5 Clustering heatmap of nucleotide and their derivatives metabolites from differently processed *Eucommia ulmoides* leaves Supplementary material 5

Supplementary material 6 Clustering heatmap of alkaloid metabolites from differently processed *Eucommia ulmoides* leaves Supplementary material 6

Supplementary material 7 Clustering heatmap of organic acid metabolites from differently processed *Eucommia ulmoides* leaves Supplementary material 7

Supplementary material 8 Clustering heatmap of lignin metabolites  from differently processed *Eucommia ulmoides* leaves Supplementary material 8

Supplementary material 9 Clustering heatmap of tannin metabolites from differently processed *Eucommia ulmoides* leaves Supplementary material 9

Supplementary material 10 Clustering heatmap of terpenoid metabolites from differently processed *Eucommia ulmoides* leaves Supplementary material 10

Supplementary material 11 Clustering heatmap of other metabolites from differently processed *Eucommia ulmoides* leaves Supplementary material 11

Supplementary material 12 Names of graphs and tables used in the article and related descriptions Supplementary material 12

## CRediT authorship contribution statement

**Yiyun Lin:** Writing – original draft, Investigation, Formal analysis, Data curation. **Hui Ouyang:** Writing – review & editing, Writing – original draft, Supervision, Project administration, Funding acquisition, Conceptualization. **Ruobing Li:** Methodology, Investigation. **Yunzhe Shao:** Methodology, Investigation. **Xiangrong Tian:** Writing – review & editing, Investigation. **Yongkang Zhang:** Supervision, Resources.

## Declaration of competing interest

The authors declare that they have no known competing financial interests or personal relationships that could have appeared to influence the work reported in this paper.

## Data Availability

Data will be made available on request.
