# Supplementary figures and images for: Integrated widely targeted metabolomics and flavoromics reveal processing-driven dynamic changes in functional metabolites of Eucommia ulmoides leaf tea
Source: Food Chem X. 2025 Apr 15;27:102434. doi: 10.1016/j.fochx.2025.102434 (PMC12059405; doi:10.1016/j.fochx.2025.102434)

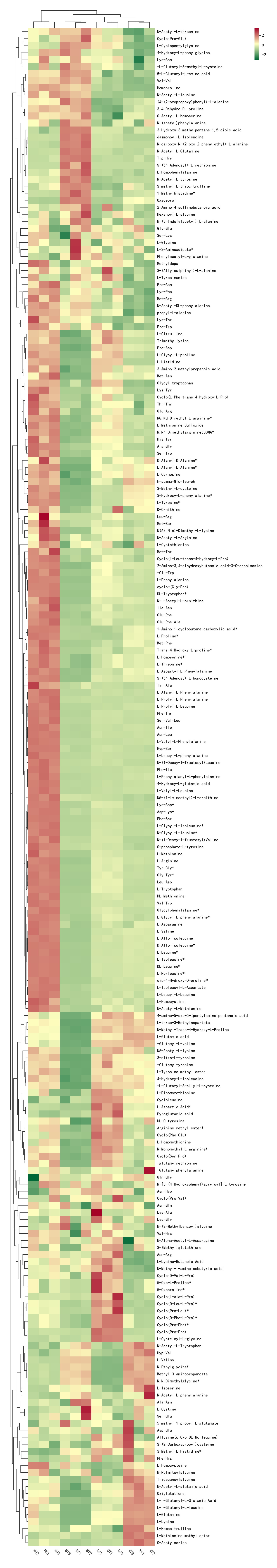

Supplement: Supplementary material 1 — Clustering heatmap of amino acids and their derivatives metabolites from differently processed Eucommia ulmoides leaves [file mmc6.pdf]

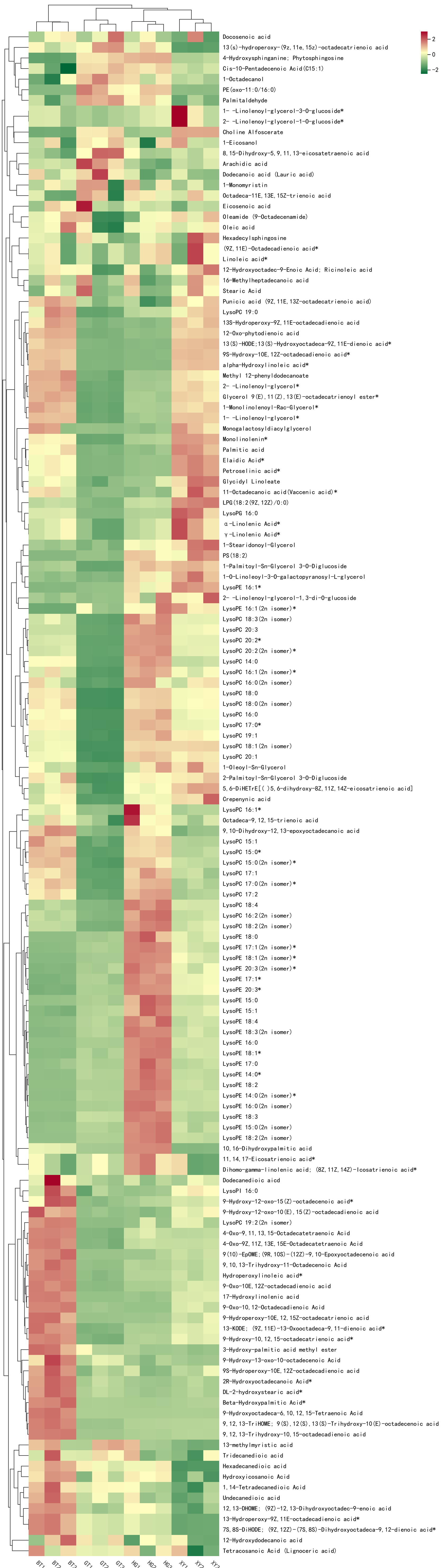

Supplement: Supplementary material 2 — Clustering heatmap of lipid metabolites from differently processed Eucommia ulmoides leaves [file mmc7.pdf]

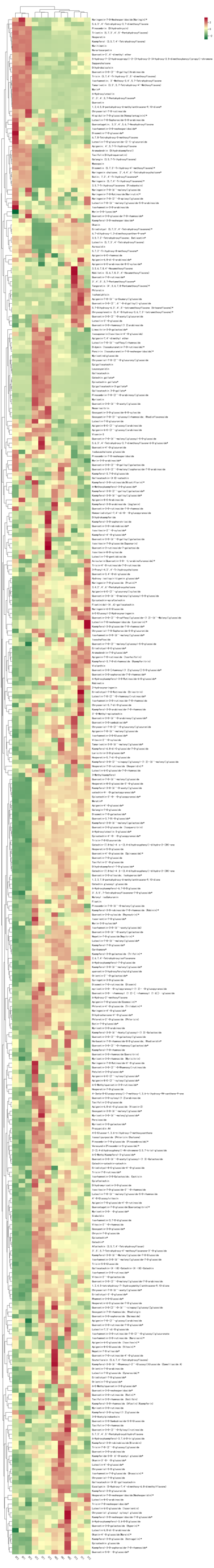

Supplement: Supplementary material 3 — Clustering heatmap of flavonoid metabolites from differently processed Eucommia ulmoides leaves [file mmc8.pdf]

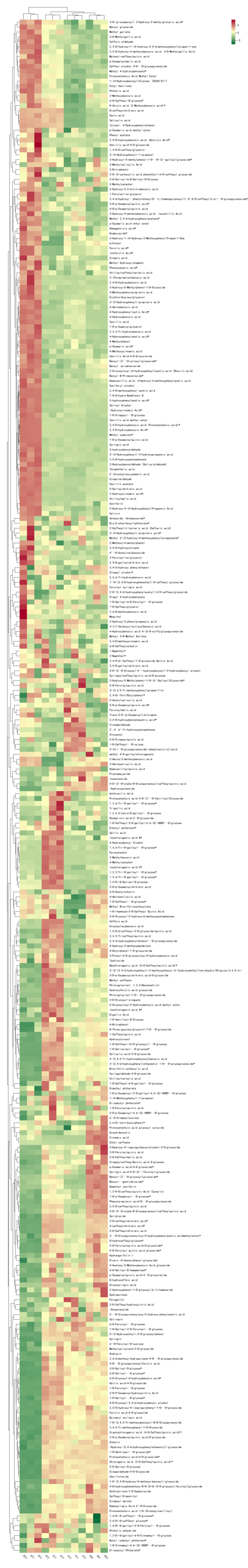

Supplement: Supplementary material 4 — Clustering heatmap of phenolic acid metabolites from differently processed Eucommia ulmoides leaves [file mmc9.pdf]

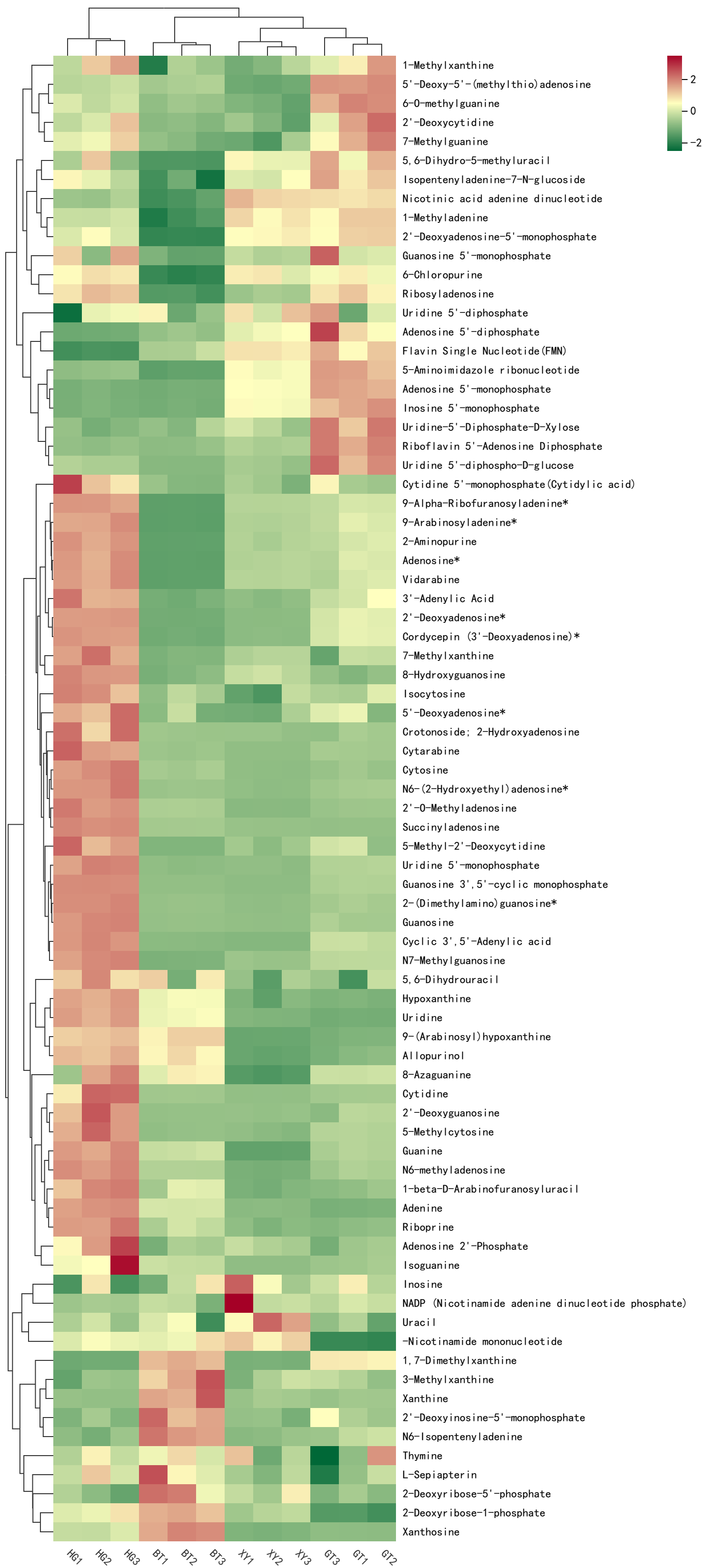

Supplement: Supplementary material 5 — Clustering heatmap of nucleotide and their derivatives metabolites from differently processed Eucommia ulmoides leaves [file mmc10.pdf]

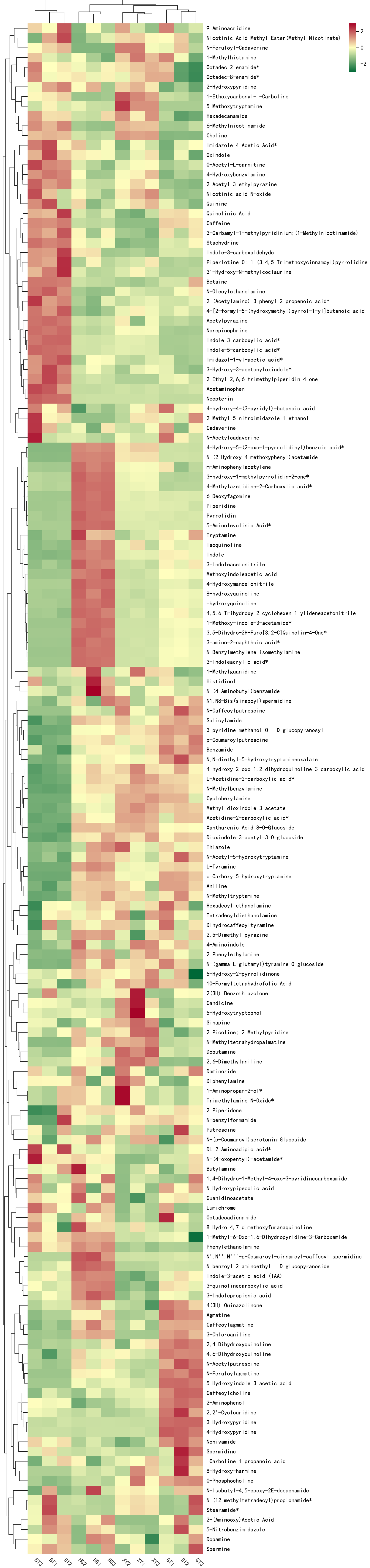

Supplement: Supplementary material 6 — Clustering heatmap of alkaloid metabolites from differently processed Eucommia ulmoides leaves [file mmc11.pdf]

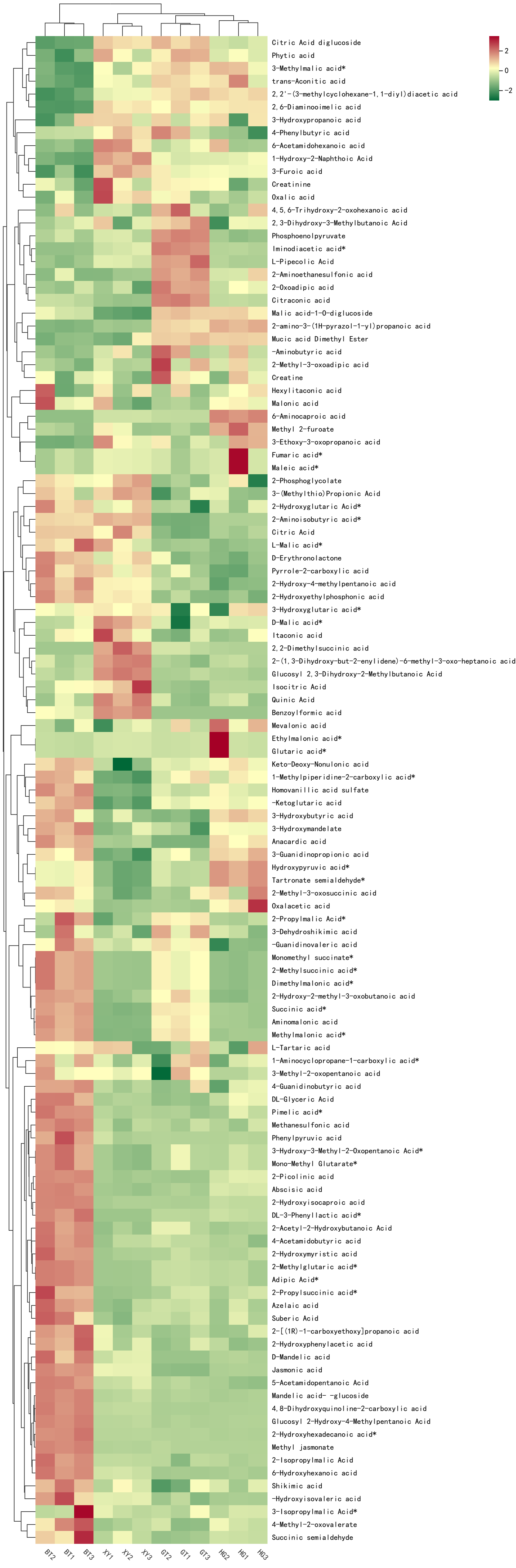

Supplement: Supplementary material 7 — Clustering heatmap of organic acid metabolites from differently processed Eucommia ulmoides leaves [file mmc12.pdf]

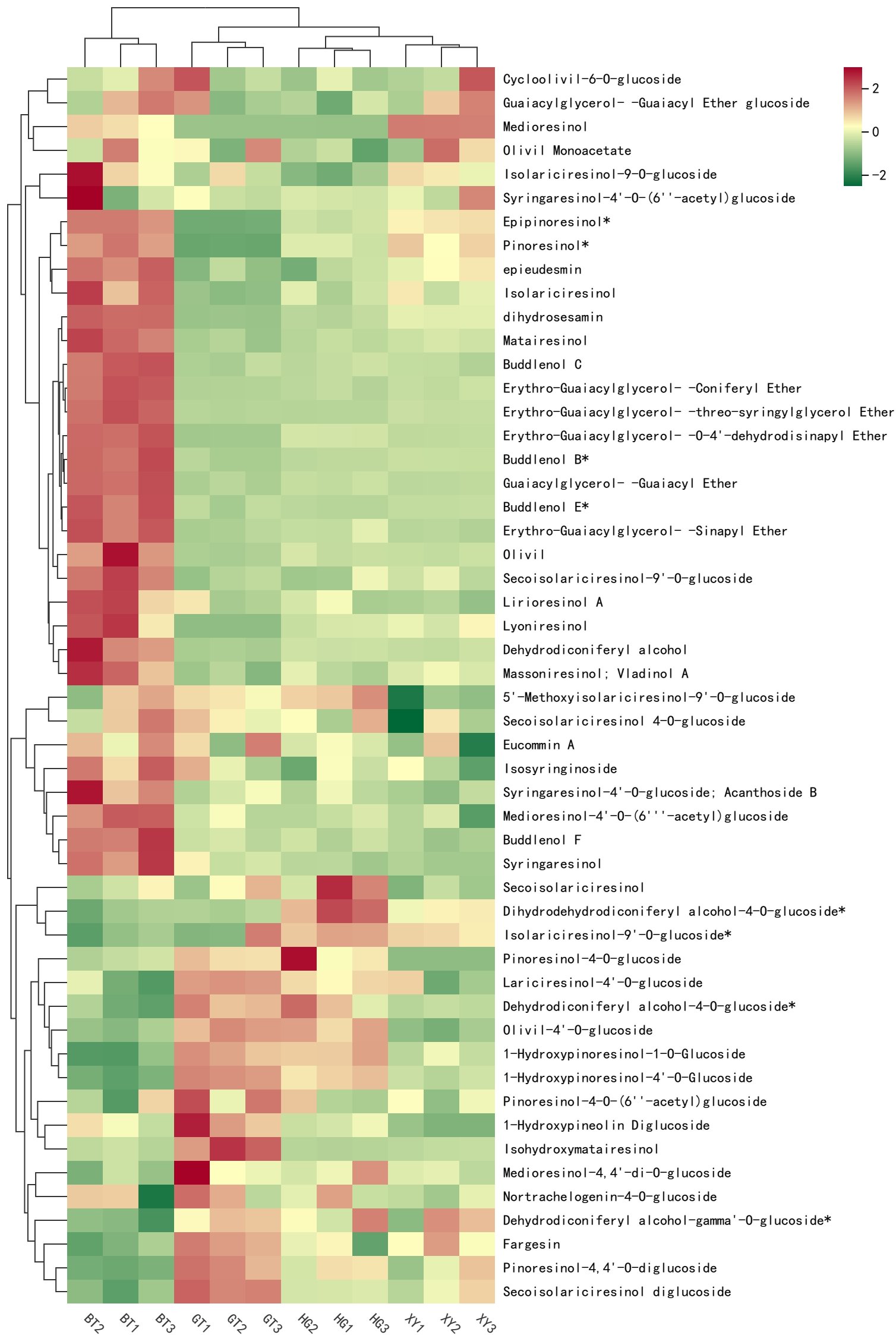

Supplement: Supplementary material 8 — Clustering heatmap of lignin metabolites from differently processed Eucommia ulmoides leaves [file mmc13.pdf]

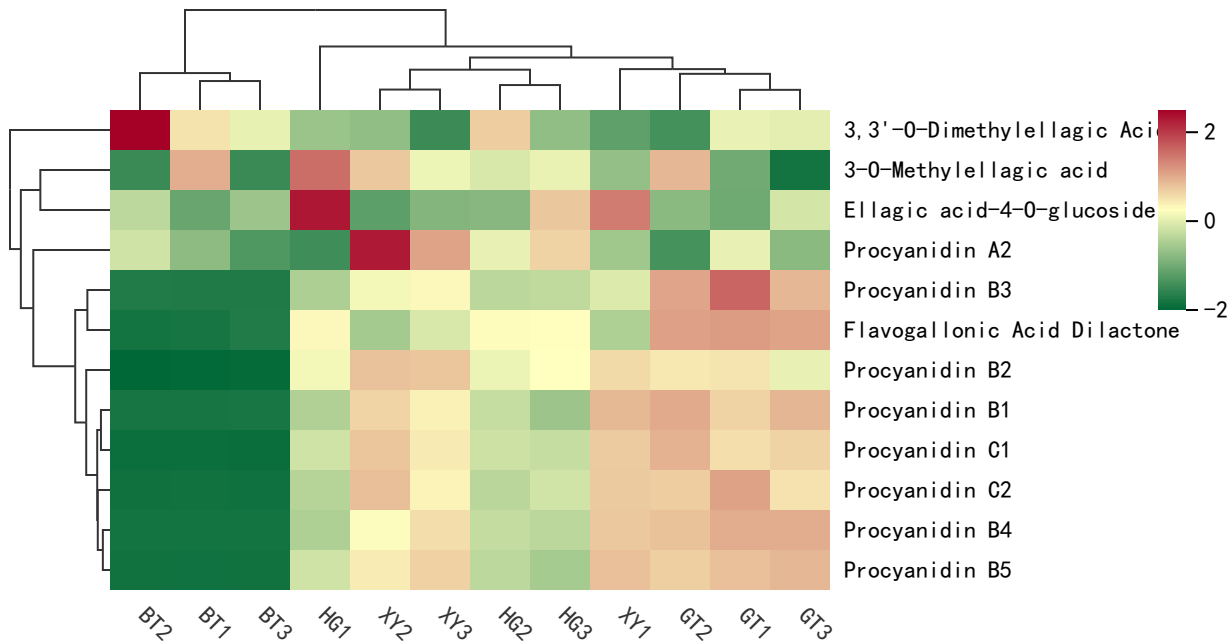

Supplement: Supplementary material 9 — Clustering heatmap of tannin metabolites from differently processed Eucommia ulmoides leaves [file mmc14.pdf]

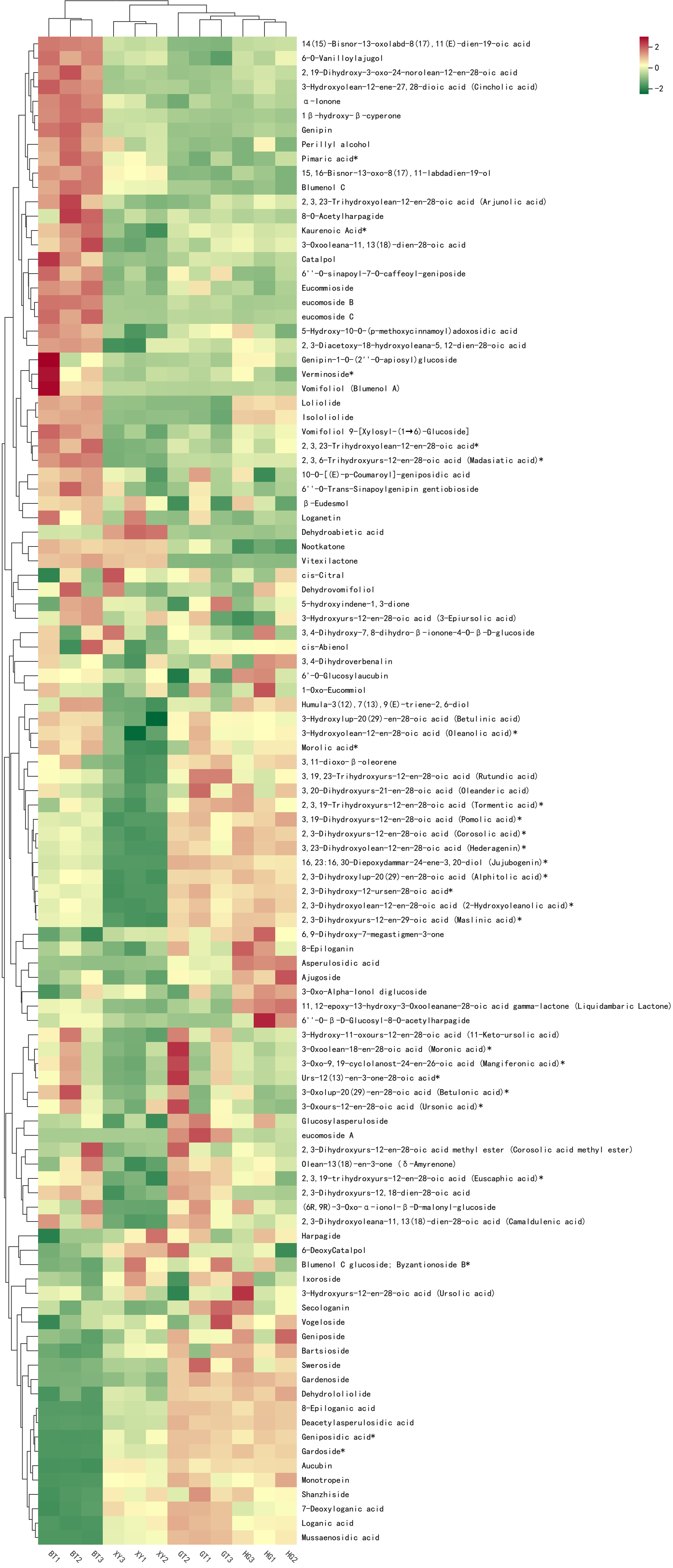

Supplement: Supplementary material 10 — Clustering heatmap of terpenoid metabolites from differently processed Eucommia ulmoides leaves [file mmc15.pdf]

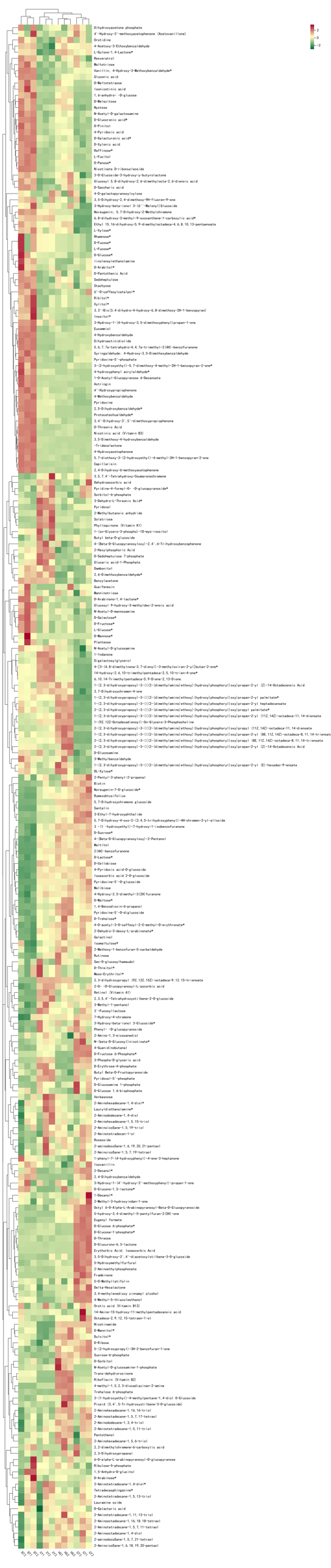

Supplement: Supplementary material 11 — Clustering heatmap of other metabolites from differently processed Eucommia ulmoides leaves [file mmc16.pdf]
