# Supplementary material for: Integrated widely targeted metabolomics and flavoromics reveal processing-driven dynamic changes in functional metabolites of Eucommia ulmoides leaf tea
Source: Food Chem X. 2025 Apr 15;27:102434. doi: 10.1016/j.fochx.2025.102434 (PMC12059405; doi:10.1016/j.fochx.2025.102434)
Supplement: Supplementary material 12 — Names of graphs and tables used in the article and related descriptions [file mmc17.docx]

**Supplementary documentation**

**Figure notes to the main text map**

**Fig. 1**. A Flow chart of different process preparation of EUL; B Brewing soup color of different samples

**Fig. 2**. A show a pie chart of the distribution of nonvolatile metabolites; B and C show the superimposed analysis of the total ion current maps of the QC samples in positive and negative ion modes; D shows the PCA scores of the nonvolatile.

**Fig. 3**. A. Volcano plots of nonvolatile metabolites in the two-by-two comparison groups, in order of BT vs. XY, HG vs. XY, GT vs. XY, BT vs. HG, BT vs.GT, HG vs.GT. Note: Each point in the volcano diagram represents a metabolite, where green points represent down-regulated differential metabolites, red points represent up-regulated differential metabolites, and gray points represent metabolites that were detected but the difference was not significant; the horizontal coordinate represents the logarithm of the multiplicity of the difference of the relative content of a metabolite between the two groups of samples (log2). FC), the larger the absolute value of the horizontal coordinate, the larger the difference in relative content of the substance between the two groups of samples. VIP + FC + P-value screening conditions: the vertical coordinate indicates the significance level of the difference (-log10 P-value), the size of the dot represents the VIP value. VIP + FC screening condition: the vertical coordinate represents the VIP value, the larger the value of the vertical coordinate, the more significant the difference is, and the more reliable the differential metabolite obtained by screening. B. Horizontal coordinates represent the grouping of samples, vertical coordinates represent the normalized relative metabolite content, Sub class represents the number of metabolite classes with the same trend, and total: represents the number of metabolites in the class.

**Fig. 4**. Variations of metabolites groups in different samples. A, B, C shows the up/down and number of adjustments bars for BT vs. XY, HG vs. XY, and GT vs XY., respectively. D: Venny for HG vs. XY and GT vs. XY and RT vs. XY.

**Fig. 5**. Pie chart of volatile metabolites（PC1=50.15%,PC2=32.07%）; 5B PCA score of volatile metabolites

F**ig. 6.** Bubble diagram for KEGG pathway enrichment analysis (A: BT vs. XY; B: GT vs. XY; C: HG vs. XY)

**Figure notes for supplementary figures**

**Fig. S1**. OPLS-DA model validation plot; Note: The horizontal coordinates represent the model R^2^Y，Q^2^ values, and the vertical coordinates are the frequency of the model classification effects in 200 random permutation experiments. In the figure, the orange color represents the random grouping model R^2^Y, the purple color represents the random grouping model Q^2^, and the values represented by the black arrows are the R^2^X, R^2^Y, and Q^2^ values of the original model.

Fig. S2.Heat map of cluster analysis of different processing processes of EUL；Note: Horizontal is the sample name, vertical is the metabolite information, Group is the grouping, and different colors are filled with different values obtained by normalizing different relative contents (red for high, green for low).

**Fig.S3**. Thermograms of non-volatile metabolites of various classes of differently processed Cortex Eucommia leave. (A-K represent one class of substances each. A: Amino acids and their derivatives; B: Lipids; C: Flavonoids; D: Phenolic acids; E: Nucleotides and their derivatives; F: Alkaloids; G: Organic acids; H: Lignans: I: Tannins; J: Terpenoids; K: Other classes)

**Fig. S4**. Radar plot of sensory flavor characterization of differential metabolites across comparison groups; Note: The outermost circle name indicates the organoleptic flavor profile, and the number corresponding to the green dot indicates the number of occurrences of the corresponding organoleptic flavor profile, i.e., the number of differential metabolites annotated to that organoleptic flavor profile.

**Fig. S5**. Differential metabolite flavor wheel plots for each comparison group; Note: the innermost circle is the differential comparison group, the second circle is the top 10 sensory flavor profiles with the highest number of differential metabolites annotated to the comparison group, the number in parentheses indicates the number of differential metabolites annotated to the sensory flavor profile, and the outermost circle indicates the differential metabolites, if the number of differential metabolites annotated to a sensory flavor profile If the number of differential metabolites annotated to an organoleptic flavor profile exceeds 10, the top 10 differential metabolites with the largest VIP values are displayed.

**Fig. S6**. Flavoromics Sankey diagrams for each comparison group；Note: The left column shows the sensory flavor profile, the right column shows the differential metabolites, and the red flow line shows the flow of up-regulated (up) differential metabolites and the blue flow line shows the flow of down-regulated (down) differential metabolites. The height of the box in the left column depends on the number of differential metabolites displayed in the right column, with the higher the number, the higher the box. When the sensory flavor profile corresponds to more than 10 differential metabolites, the top 10 differential metabolites with the highest VIP values are displayed.

**Name of the form and indication**

**Table. 1**. Total score table of different processed *Eucommia ulmoides leaves* (EUL), (including Contour, Liquor color, Aroma, Taste, Infused leaf, Total points, Ranking).

**Table. 2**. Summary table of non-volatile metabolite compositions, classifications and relative content of substances in different processed EUL.

**Table. 3** Table of major differences in composition between groups of non-volatile metabolites of different processed EUL. Note: VIP: variable importance projection, P-value: significance test P-value, Fold_Change: difference multiple (Fold_Change=experimental group average/control group average, when the control group average is 0, Fold_Change is infinite Inf), Log2FC: difference multiple is taken as the logarithm of 2 (when Fold_Change is infinite, Log2FC value is also infinite Inf); When Fold_Change is 0, Log2FC value is negative infinity Inf), Type: metabolite upregulation type.

**Table. 4**. Summary table of volatile metabolite composition, classification and relative content of substances in different processed EUL.

**Table. 5**. Table of major differences in composition between groups of volatile metabolites of different processed EUL. Note: VIP: variable importance projection, P-value: significance test P-value, Fold_Change: difference multiple (Fold_Change=experimental group average/control group average, when the control group average is 0, Fold_Change is infinite Inf), Log2FC: difference multiple is taken as the logarithm of 2 (when Fold_Change is infinite, Log2FC value is also infinite Inf); When Fold_Change is 0, Log2FC value is negative infinity Inf), Type: metabolite upregulation type.
